# Combining In Vitro, In Vivo, and Network Pharmacology Assays to Identify Targets and Molecular Mechanisms of Spirulina-Derived Biomolecules against Breast Cancer

**DOI:** 10.3390/md22070328

**Published:** 2024-07-22

**Authors:** Soha Osama Hassanin, Amany Mohammed Mohmmed Hegab, Reham Hassan Mekky, Mohamed Adel Said, Mona G. Khalil, Alaaeldin Ahmed Hamza, Amr Amin

**Affiliations:** 1 Biochemistry Department, Faculty of Pharmacy, Modern University for Technology and Information, Cairo 11585, Egypt; soha.hassanin@pharm.mti.edu.eg; 2 Egyptian Drug Authority (EDA), Formerly National Organization of Drug Control and Research, Developmental Pharmacology and Acute Toxicity Department, Giza 12611, Egypt; manalhegab2630@gmail.com; 3 Department of Pharmacognosy, Faculty of Pharmacy, Egyptian Russian University, Badr City, Cairo-Suez Road, Cairo 11829, Egypt; reham-mekky@eru.edu.eg; 4 Department of Pharmaceutical Chemistry, Faculty of Pharmacy, Egyptian Russian University, Badr City, Cairo 11829, Egypt; 5 Pharmacology and Toxicology Department, Faculty of Pharmacy, Modern University for Technology and Information, Cairo 11829, Egypt; 6 Biology Department, Egyptian Drug Authority (EDA), Formerly National Organization of Drug Control and Research (NODCAR), Giza 12611, Egypt; 7 Medical Research Council, Academy of Scientific Research and Technology, Cairo 11334, Egypt; 8 Basic Medical Sciences, College of Medicine, University of Sharjah, Sharjah 27272, United Arab Emirates

**Keywords:** spirulina, bioactive metabolites, BC, network pharmacology, in vivo, MCF-7 cell

## Abstract

The current research employed an animal model of 7,12-dimethylbenz(a)anthracene (DMBA)-induced mammary gland carcinogenesis. The estrogen receptor-positive human breast adenocarcinoma cell line (MCF-7) was used for in vitro analysis. This was combined with a network pharmacology-based approach to assess the anticancer properties of Spirulina (SP) extract and understand its molecular mechanisms. The results showed that the administration of 1 g/kg of SP increased the antioxidant activity by raising levels of catalase (CAT) and superoxide dismutase (SOD), while decreasing the levels of malonaldehyde (MDA) and protein carbonyl. A histological examination revealed reduced tumor occurrence, decreased estrogen receptor expression, suppressed cell proliferation, and promoted apoptosis in SP protected animals. In addition, SP disrupted the G2/M phase of the MCF-7 cell cycle, inducing apoptosis and reactive oxygen species (ROS) accumulation. It also enhanced intrinsic apoptosis in MCF-7 cells by upregulating cytochrome c, Bax, caspase-8, caspase-9, and caspase-7 proteins, while downregulating Bcl-2 production. The main compounds identified in the LC-MS/MS study of SP were 7-hydroxycoumarin derivatives of cinnamic acid, hinokinin, valeric acid, and α-linolenic acid. These substances specifically targeted three important proteins: ERK1/2 MAPK, PI3K-protein kinase B (AKT), and the epidermal growth factor receptor (EGFR). Network analysis and molecular docking indicated a significant binding affinity between SP and these proteins. This was verified by Western blot analysis that revealed decreased protein levels of p-EGFR, p-ERK1/2, and p-AKT following SP administration. SP was finally reported to suppress MCF-7 cell growth and induce apoptosis by modulating the PI3K/AKT/EGFR and MAPK signaling pathways suggesting EGFR as a potential target of SP in breast cancer (BC) treatment.

## 1. Introduction

Breast cancer (BC) still affects millions of women every year and is a serious public health issue [1]. Approximately two million new cases were reported worldwide in 2022 [2]. BC is the most frequent disease among women, accounting for 11.7 percent of all cancer diagnoses, according to Chhikara and Parang [2]. Unfortunately, it was the main reason why African women died from cancer and was responsible for 20% of all cancer-related fatalities on the continent [1]. Hereditary factors and aging are two risk factors for BC. However, environments with motor fuel, industrial activity, and coal burning are more likely to include polycyclic aromatic hydrocarbons (PAH) [3]. Research on the PAH 7,12-dimethylbenz(a)anthracene (DMBA) has been heavily pursued because of its association with breast tissue cancer. In this work, DMBA was used to generate mammary gland cancer. Remarkably, the histological changes and hyperplastic growth shown in the chemical model of DMBA-induced rat BC are strikingly similar to the development of human breast cancers that originate from the terminal ductal lobular unit [3,4].

The tissues of the liver and breasts are where the carcinogenic chemical DMBA is activated. This activation leads to the development of DMBA-3,4-diol-1,2-epoxide, which promotes oxidative stress, by upsetting the tissue’s redox balance. Within the cell, reactive oxygen species (ROS) can harm proteins or DNA, which can lead to dysregulated cell division and proliferation [4]. Cancer cells can multiply when there is estrogen in the bloodstream since the majority of breast cancers include estrogen hormone receptors (ERs) [3]. Patients with BC benefit from medications that prevent DNA damage, decrease cell cycle progression and division, or trigger apoptosis. Additionally, these therapies inhibit particular pathways that result in the production of abnormal cells [3,5]. Synthetic drugs are less likely to induce side effects than plant-based anticancer treatments, like vinblastine, vincristine, and paclitaxel (derived from Taxus brevifolia). Furthermore, chemoprevention has been suggested as a practical way to decrease environmental carcinogens and genotoxins’ detrimental effects [6,7]. According to Iqbal et al. [5] and Laskar et al. [3], these chemopreventive drugs, among other cancer-prevention techniques, decrease cell growth, estrogenic activity, oxidative stress, the activation of a detoxifying enzyme, and induce apoptosis.

Certain medications, including herbal remedies, may be helpful in the treatment and prevention of BC if they have the ability to change these signaling cascades [8,9]. A well-known food supplement, Spirulina (SP), consists of filamentous cyanobacteria species such as Spirulina maxima, Spirulina fusiformis, and Spirulina platensis [10]. Spirulina’s richness in vitamins, minerals, amino acids, fatty acids and polysaccharides has led to numerous studies showing the benefits of Spirulina as food supplements [10,11,12,13,14,15] includes nutrients found in SP, such as phycocyanin, cyanocobalamin, riboflavin, B-carotene, and phenolic acids. The main source of SP’s noteworthy anti-inflammatory, antioxidant, anticancer, and apoptotic properties [16,17,18,19,20] is the richness of its active biological components. In addition, there is growing interest in SP among cancer researchers. Studies have shown different types of SP preparations in preventing the growth of cancer cells in different types including human liver cancer [21,22], colorectal cancer [23,24], breast cancer [25,26,27,28], lung cancer [29], pancreatic cancer [30], and bone marrow cancer [31]. SP has been investigated for its chemopreventive potential in the prevention of liver cancer [14,32,33], invasive endometrial [34], colon cancer [35], and breast cancer [36]. A study found that SP extract, purified phycocyanin extract, and purified allophycocyanin can prevent endometrial cancer cells from migrating and spreading to the peritoneum in nude mice via regulating the TGFβ/SMAD4 signaling pathway [34].

Although SP extracts have anticancer properties, it is still unknown how they function, and the intrinsic mechanisms of breast cancer action are not fully understood. Furthermore, the active bioactive components’ targets and pharmacological processes are unclear, creating a barrier to the creation of herbal treatments. Understanding the mechanisms and pharmaceutical targets of active biological components are additional impediments to the development of herbal remedies. The efficacy and mechanisms of SP extract were investigated in order to evaluate its potential as an anticancer agent using MCF-7 BC cells and DMBA-induced mammary gland carcinogenesis. The impact of SP on proliferation was assessed with a focus on ER functions and ROS in both in vitro and in vivo contexts. The ability to predict the main targets and pathways of SP components in BC therapy was made possible by the use of target fishing and network pharmacology. The disruptions of the targets and pathways were verified by molecular docking. Finally, by verifying SP’s main targets and mechanisms against BC in vitro, the pharmacological effect was validated. The findings indicate that SP constituents induce apoptosis and inhibit cell proliferation by blocking the EGFR/Akt and ERK1/2 MAPK signaling pathways. Therefore, the development of small molecule inhibitors targeting these pathways for BC prevention and therapy requires a rigorous validation of active components and their corresponding targets.

## 2. Results

### 2.1. SP Reduces Tumor Histopathological Alterations

**Figure 1:**
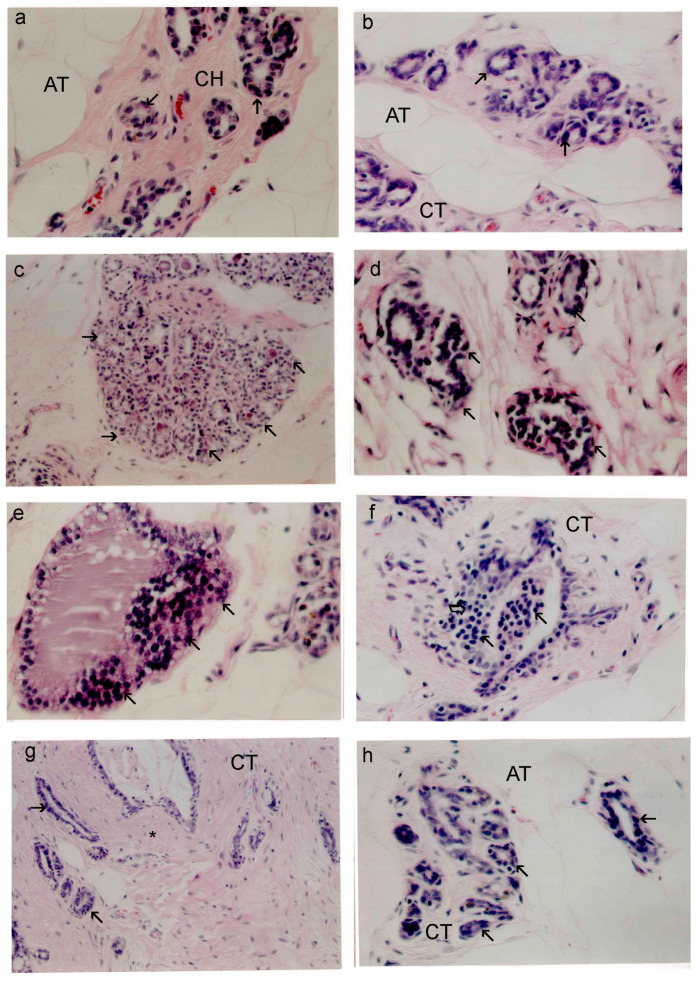
Standard mammary gland structure in histological sections (×200) of rat mammary tissues from the nontreated control (**a**) and treated SP (**b**) groups. Small mammary ducts are in the glands, partly surrounded by fibrous connective tissue (CT) and adipose tissues (ATs). Sections from the DMBA group (**c**–**f**) exhibit diverse histological alterations. (**c**) Dysplastic mammary gland (×200): the arrow indicates additional ducts and uneven cell division. Focal patches of dysplastic cells may be detected in these moderately dilated breast ductal tubules. (**d**) Fibroadenoma (×400): the arrow indicates a localized region of considerable ductal and epithelial hyperplasia, which inducts fibrosis (*). Invasive ductal carcinoma displays the proliferation of intraductal neoplastic epithelial cells with remarkable variations in cellular and nuclear sizes and shapes, which invaded the neighbor stroma, as seen in (**e**) (×400). The arrow represents a small lobular proliferation and localized epithelial hyperplasia with hyperchromatic enlarged nuclei. (**f**) (×400) The intraductal papillary carcinoma in situ: the arrow represents the micropapillae of neoplastic cells. (**g**,**h**) DMBA + SP group fibroadenoma (**g**) (×200) and stage of mammary gland cell death (**h**) (×400). (*) Fibrosis and CT connective tissues. H&E staining was used on all sections.

Figure 1 shows the normal structure of breast tissues as well as the abnormal changes brought on by DMBA use. Tiny ducts can be seen in the mammary cells of rats treated with SP and control rats, respectively, in Figure 1a,b. Adipose tissue (AT) and fibrous connective tissue (CT) in abundance, as well as a single layer of cuboidal epithelial cells, encircle these ducts. According to the current study, DMBA encouraged the growth of terminal ductal breast tissue in rats, which led to the development of hyperplastic lesions that were both premalignant and malignant and closely resembled human breast cancer. As a prevalent marker of mammary gland cancer, ductal epithelial dysplasia (shown in Figure 1c) is seen in 25% of all mammary glands evaluated after DMBA treatment. It is characterized by abnormal cellular extensions into the inner space, an increase in microscopic ducts, and uneven cell division. Nuclear shape varies and polarity is absent in dysplastic cells. Atypical ductal hyperplasia, which is defined by abnormal cell proliferation in the mammary glands’ interior and duct lining, is shown in Figure 1d. The size of neoplastic cells was significantly greater than that of the normal cells. These changes were seen in 55% of DMBA-treated mammary glands. It was found that some or all of the ducts were blocked by abnormal proliferative cells, also referred to as “carcinoma in situ”. Eight percent of the sections under examination have localized ductal cancer. An intraductal papillary carcinoma is depicted in Figure 1e, where the ductal structures are dilated and the lining epithelium develops into epithelial papillae. The invasive ductal carcinoma depicted in Figure 1f is characterized by the proliferation of intraductal neoplastic epithelial cells that penetrate the neighboring stroma. These cells exhibit notable differences in their cellular and nuclear sizes and shapes. When compared to healthy cells, the neoplastic cells were noticeably bigger. Rats treated with both SP and DMBA showed a reduction in all cancer types, with the exception of some fibroadenomas and proliferating neoplastic cells seen in particular ductal walls (Table 1 and Figure 1h,g).

### 2.2. SP Reduces Oxidative Stress Breast Tissues of DMBA-Treated Rats

In this study, it was discovered that SP lowers oxidative stress in rats that have mammary tumors caused by DMBA. Table 2 displays the impact of SP on the concentrations of oxidative stress indicators in rats that had breast cancer. Rats administered DMBA had significantly higher levels of MDA and P carbonyl in their breast tissues. Compared to the data of the control group, there was a discernible decrease in TAC content, CAT, and SOD activity after the increases. Nevertheless, the administration of SP to the DMBA-treated group prevented declines in CAT, SOD, and TAC levels as well as increases in MDA and P. carbonyl levels when compared to DMBA alone. It is interesting to note that SP treatment by itself had no discernible effect on these oxidative stress markers when compared to the control group (Table 2). 

### 2.3. SP Decreases the Expression of PCNA and ER-α in the Breast Tissues of DMBA-Treated Rats

**Figure 2:**
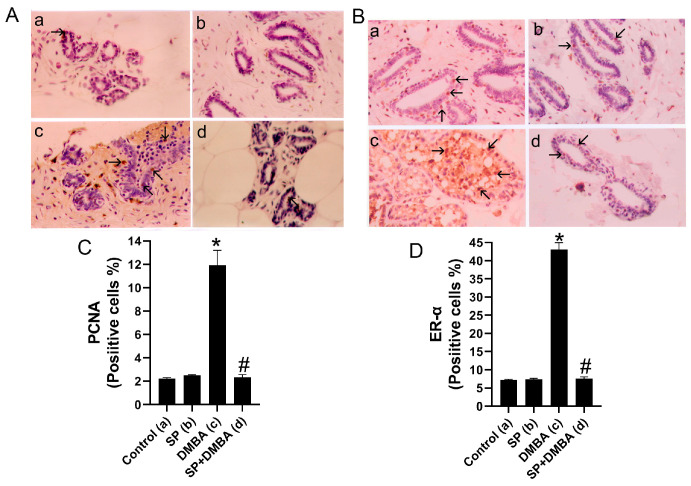
Figure displays immunohistochemical staining for PCNA and ER-α in breast sections, labeled as (**A**,**B**). Rats labeled as A and B were subjected to several treatments: (**a**) vehicle (as a control), SP (**b**), DMBA (**c**), and DMBA + SP (**d**) exhibiting PCNA- and ER-α-positive cell expressions in breast tissues. Photomicrographs and quantitative analysis (**C**,**D**) demonstrate the quantity of PCNA- and ER-α-positive cells The quantification of PCNA and ER-cells in each slice was conducted by enumerating the number of cells exhibiting brown staining positivity out of a total of 1000 cells seen at a magnification of 400×. Arrows indicate the presence of PCNA and ER-α-positive cells (H counter stained, 400×). Values expressed as mean ± SEM for six animals in each group. Significance was determined by one-way analysis of variance followed by a post hoc Dunnett’s test. * *p* < 0.05 vs. control group; # *p* < 0.05 vs. DMBA group.

The effects of SP alone or in combination with DMBA treatment on the expression of PCNA and ER-α in the breast tissues of control, SP, and DMBA-treated rats were investigated using immunohistochemistry. Figure 2C,D demonstrates a notable rise in the quantity of PCNA- and ER-α-positive cells in the breast tissues of rats subjected to DMBA treatment, in comparison to the control group. The observation is derived from the study of immunohistochemical staining for PCNA and ER-α, as depicted in Figure 2A,B. In contrast, the administration of SP to rats treated with DMBA greatly reduced the proliferation of PCNA- and ER-α-positive cells in the mammary gland, as seen in Figure 2. This impact was apparent when comparing the group treated with SP to both the control group and the group treated with DMBA. However, compared to the control group, administration of SP alone did not significantly increase the proportion of PCNA- and ER-α-positive cells.

### 2.4. Effect of SP on TUNEL-Positive Cells in the Mammary Tissues of DMBA-Treated Rats 

**Figure 3:**
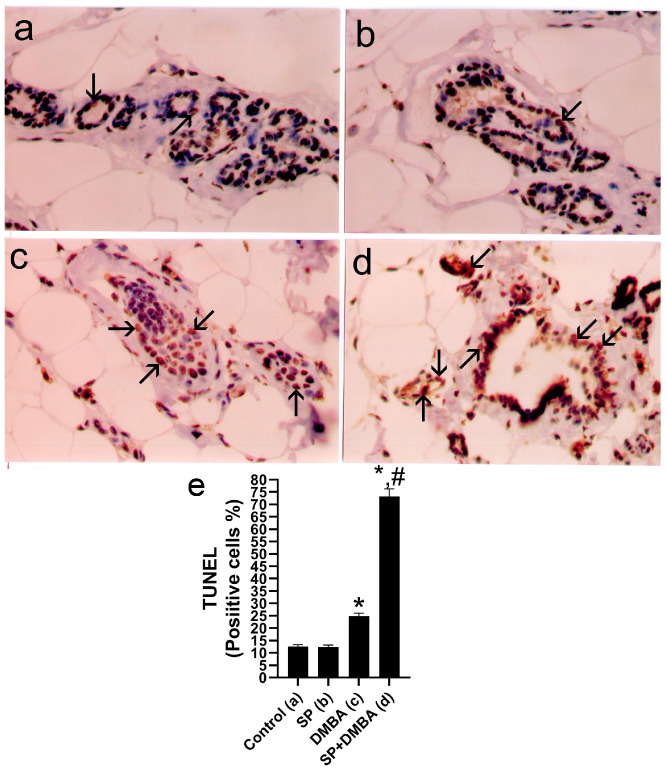
Figure displays the presence of TUNEL-positive cells in the mammary tissues of rats subjected to several treatments: (**a**) vehicle (as a control), SP (**b**), DMBA (**c**), and DMBA + SP (**d**). The semi-quantitative analysis (**e**) and the photomicrographs reveal the percentage of TUNEL-positive cells in various experimental groups. The percentage of TUNEL-positive cells in each slice was determined by quantifying the number of cells exhibiting brown staining using a 400× magnification, out of a total of 1000 cells. Arrows show TUNEL-positive cells (400×, H counterstained). Values expressed as mean ± SEM for six animals in each group. Significance was determined by one-way analysis of variance followed by a post hoc Dunnett’s test. * *p* < 0.05 vs. control group; # *p* < 0.05 vs. DMBA group.

The TUNEL test was utilized to evaluate apoptosis in the breast tissues of treated rats. Figure 3 illustrates the impact of DMBA and SP on TUNEL labeling on breast tissues. The use of immunohistochemical labeling with FUNEL demonstrated a notable increase in the quantity of TUNEL-positive cells in the breast tissues of rats treated with DMBA, in comparison to the control group (Figure 3e). In contrast, rats who received SP + DMBA treatment showed a notably elevated apoptotic index in their mammary glands when compared to the control and DMBA-only groups (Figure 3). However, SP therapy alone did not have a significant impact on the number of TUNEL-positive cells as compared to the control group.

### 2.5. Total Antioxidant Capacity and Phenolic Content of SP

The total capacity of antioxidants and the concentration of phenolic compounds were assessed. The FRAP method is a dependable and uncomplicated technique for quantifying the overall quantity of antioxidants. The current study demonstrates that dehydrated SP exhibits a significant FRAP value and total capacity of antioxidants of 9.56 mol of ascorbic acid equivalent per gram of SP. Furthermore, the overall polyphenolic content of SP is significant, quantified at 5.82 mg of gallic acid equivalent per gram of SP.

### 2.6. SP and DOX Effects on the Viability of BC Cells

**Figure 4:**
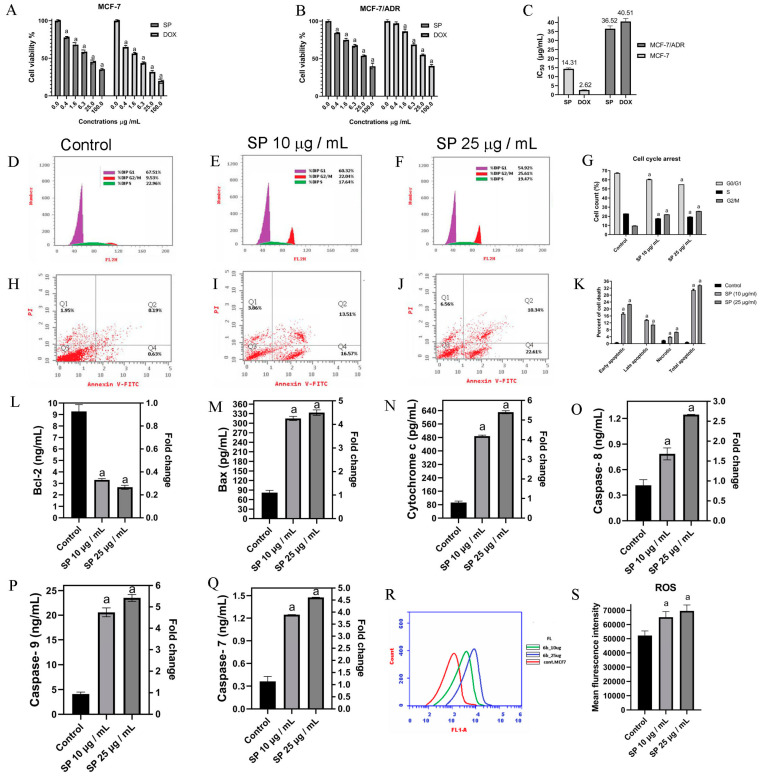
(**A**–**C**) Breast cancer cell growth decreased by SP. (**A**–**C**) illustrates how SP and DOX significantly reduce the viability of breast cancer cells. MCF-7 and MCF-7/ADR cells were treated with varying concentrations of SP and DOX, and 24 h later, their viability was evaluated. (**D**–**G**) demonstrate that SP produced cell cycle arrest using flow cytometry, a method by which the amount of cellular DNA was assessed following PI staining. (**H**–**J**) percentage of cells in the S, G1, G2, and M stages. In every instance, untreated cells in their growth media were used as controls. SP-treatment histograms for MCF-7 cells at zero (**D**), 10 µg/mL (**E**), and 25 µg/mL (**F**). (**G**) Three studies were used to calculate the average proportion of cells in each cell cycle phase. Cell apoptosis was observed using flow cytometry and an Annexin V/PI apoptosis detection kit (**H**–**J**). The dual parametric dot plots that incorporated PI fluorescence and Annexin V-FITC analysis reveal that early apoptotic cells are located in the bottom-right quadrant (Q4), late apoptotic cells in the top-right quadrant (Q2), and the viable cell population in the bottom-left quadrant (Q3). (**K**) demonstrates the percentage of cell necrosis and apoptosis. The reason for MCF-7 cell death is SP, which requires mitochondria. (**L**–**Q**) The expression of extrinsic and intrinsic apoptosis-related proteins (Bax, Bcl-2, cytochrome c, caspase 8, caspase 9, and caspase 7) was measured using microplate readers and ELISA kits following MCF-7 cells treated with suitable amounts of SP for 24 h. (**R**) The amount of ROS was measured using flow cytometry. The mean fluorescence density of the ROS level was calibrated using (**S**). All the data (n = 3) are displayed as mean ± SEM. One-way ANOVA was utilized first, and then Tukey’s post hoc analysis was carried out. ^a^ *p* < 0.05 was obtained when compared to control cells.

The initial assessment involved evaluating the toxic effects of SP and DOX on both MCF-7 and drug-resistant MCF-7/ADR cells. The MTT assay evaluated the viability of these cells following treatment with varied doses of SP and DOX (0.4, 1.6, 6.3, 25, and 100 μg/mL) for 24 h (Figure 4A–C). The results show that both SP and DOX exhibit cytotoxic properties against MCF-7 and MCF-7/ADR cells, causing a reduction in cell viability that is dependent on the dosage. The vitality of these cells is considerably suppressed by SP, as shown in Figure 4A. The IC50 values of MCF-7 and MCF-7/ADR cells were 14.31 ± 0.56 μg/mL and 36.52 ± 1.54 μg/mL, respectively (Figure 4C), indicating the potent cytotoxic impact of SP on MCF-7 cells. Treatment with DOX (used as a positive control) significantly induced cytotoxicity in both MCF-7 and MCF-7/ADR cells, exerting a pronounced growth-inhibitory effect on both cell lines. The IC_50_ values for MCF-7 cells were 2.62 ± 0.14 μg/mL and 40.51 ± 1.71 μg/mL for MCF-7/ADR cells. Figure 4C shows that the IC50 values of SP and DOX were substantially higher in drug-resistant cells compared to chemosensitive cells. The IC50 value of DOX was significantly higher in MCF-7/ADR cells compared to MCF-7 cells, with an approximate 15-fold difference. Similarly, the IC_50_ value of SP in MCF-7/ADR cells was about 2.5-times higher than in MCF-7 cells. Based on these findings and the IC_50_ values of SP, MCF-7 cells were selected for further experimentation. 

### 2.7. SP Causes Cell Cycle Arrest at G2/M and Promotes Apoptosis

Flow cytometry was used to analyze the effect of SP on cell cycle distribution (Figure 4D–G). At dosages of 10 and 25 µg/mL of SP, the G0/G1 phase ratio in our data declined with increasing the dose, shifting from 67.51% to 60.32% and 54.92%, respectively. The ratio of S phase cells similarly decreased dose-dependently following SP therapy, from 22.96% to 17.64% and 19.47%. However, the G2/M phase ratio significantly increased in response to SP treatments, shifting from 9.53% to 22.04% and 25.61%. The Annexin V binding assay, a dependable technique for identifying apoptosis, offers crucial insights into how SP affects cellular death, as demonstrated in Figure 4H–K. Our study’s findings demonstrate that SP exposure significantly increased the number of apoptotic cells. Apoptosis was particularly seen in 30.08% and 32.95% of cells, respectively, during the course of a 24 h incubation period at doses of 10 and 25 µg/mL, in contrast to only 0.82% in the control group. Furthermore, rather than late apoptosis, our data indicate that early apoptosis was the primary mediator of SP-induced apoptosis (Figure 4K). After being treated with SP, the cells showed a dose-dependent increase in apoptosis, which resulted in cell cycle arrest. 

The intrinsic apoptotic pathway and its reliance on caspase activation were investigated in order to gain a better understanding of the molecular mechanism by which SP extracts induce apoptosis. ELISA assays were used to evaluate the expression levels of caspases 7, 8, and 9, as well as Bcl-2, BAX and cytochrome c (Figure 4L–Q). Contrary to pro-apoptotic proteins BAX, cytochrome c, caspase 7, caspase 8, and caspase 9, anti-apoptotic protein Bcl-2 expression reduced in response to increasing the dose. The administration of SP resulted in a considerable rise in the levels of caspase-9 and cytochrome c when compared to the control group. The increases were as much as 5.77- and 5.22-fold at dosages of 10 and 25 g/mL, respectively. Following SP treatment, caspase-8 levels also dramatically rose; 1.89- and 2.99-fold increases were seen (Figure 4O). Furthermore, caspase-7 levels rose sharply by 3.44- and 4.07-fold after receiving SP therapy (Figure 4Q). The mitochondrial-mediated system that SP primarily activates is the caspase-9-dependent intrinsic mechanism that causes MCF-7 cells to undergo programmed cell death, according to recent studies.

Using flow cytometry and DCFH-DA in an intracellular accumulation technique, rogue oxygen species (ROS) were measured intracellularly in cultivated MCF-7 cells. The dose-dependent increase in ROS buildup brought on by SP treatment can be seen in Figure 4 R-S. Based on these results, the buildup of ROS has a significant effect on the SP-induced apoptosis *p*.

### 2.8. Characterization by LC-MS and MS^2^ of Spirulina

**Figure 5:**
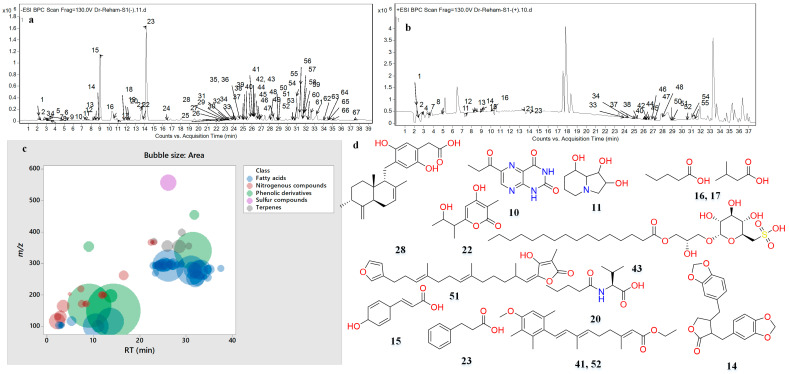
Base peak chromatograms (BPCs) of Spirulina extract in (**a**) the negative and (**b**) positive ionization modes; (**c**) bubble plot of the observed masses vs. the retention time in relation to metabolite classes; and (**d**) structures of the major compounds.

The hydroalcoholic extract of SP (*Arthrospira platensis*) was metabolically profiled and was found to contain 67 compounds. Figure 5 illustrates SP’s base peak chromatograms (BPC) in both negative and positive ionization modes. The annotated phytochemicals were categorized as phenolic derivatives (6), nitrogenous compounds (14), fatty acids (39), sulfur compounds (1), and terpenes (5). Molecular ion peaks (*m*/*z*), retention times (RTs), neutral losses, generated molecular formula, double-bond equivalence (DBE), and relative abundance were recorded for each annotated metabolite (Figure 5, Table 3 and Table S1A). To characterize the compounds, their generated chemical formulae and fragmentation patterns were compared to those found in the Reaxys database and relevant literature (Table S1A).

New technologies, such as HPLC-QTOF-MS and MS/MS, have facilitated the study of metabolites previously undiscovered [37,38]. These metabolites may be responsible for SP’s anticancer properties. Among phenolic derivatives, which constituted 43.84% of the relative abundance of all metabolites found, neutral losses of CO (28 Da), CO_2_ (44 Da), and water (18 Da) were observed [39,40]. They were subdivided into lignans, dihydrocinnamic acids, and other phenols. Hinokinin with *m*/*z* 353.1 and consistent fragments has been previously reported [41]. Dihydrocinnamic acid (*m*/*z* 149.06, relative abundance 19.4%) and dihydrocoumaric acid (*m*/*z* 165.06, relative abundance 12.96%) were the most abundant molecules among the identified metabolites. Additionally, 2,2′-Bis(4-methyl6-tert-butylphenol) methane (*m*/*z* 339.26) was detected in SP [42]. The chromane germicidin M (*m*/*z* 197.08) was also found in SP, previously described in *Streptomyces* sp. OUCMDZ-3436 from the marine green algae *Enteromorpha prolifera* [43]. Another quinone, chromequinolide (*m*/*z* 453.23), was found and first described in the marine brown algae *Sargassum sagamianum* [44] (Table 3 and Table S1). As for the nitrogenous compounds, the annotated alkaloids were identified for the first time in *A. platensis* and characterized using previously published data. They accounted for 0.65% of the relative abundance. Furthermore, the identified amino acids, responsible for 4.2% of the relative abundance were characterized using previous research [38,45,46].

Additionally, seven fatty acid amino acid conjugates were discovered for the first time in A. platensis, accounting for 1.64% of the relative abundance (Table 3 and Table S1A). In summary, two forms of N-butyryl-leucine/isoleucine I and II were identified, with the butyryl part removed, leaving behind leucine/isoleucine ions (*m*/*z* 130.09). The presence of valine and valeric acid ions at *m*/*z* 116.07 and 102.06, respectively, indicates the presence of N-valeryl-valine. Furthermore, N-caproylphenylalanine (*m*/*z* 262.15) exhibited a phenylalanine ion (Table 3 and Table S1A).

The most abundant metabolite class in SP was found to be fatty acids, both qualitatively (39 derivatives) and quantitatively (40.46%). Previous research has elucidated the neutral loss of CO (28 Da), CO_2_ (44 Da), and H_2_O (18 Da) moieties in fatty acids [47,48,49]. Short-chain fatty acids, such as hydroxybutyric acid I-III, valeric/isovaleric acid I-II, hydroxy valeric acid, and butyl acetate, were identified. Additionally, long-chain fatty acids like palmitic acid (C16:0) I–II, along with their oxo- and unsaturated derivatives, oxoplamitic acid and palmitoleic acid I–II (C16:1), were observed. Stearic acid (C18:0) and its monounsaturated form, oleic acid I–II (C18:1), were also detected along with their hydroxylated forms, hydroxyoleic acid I–IV. Furthermore, di-unsaturated stearic acid, viz., hydroxylinoleic acid I–II (C18:2) and its hydroxylated forms, hydroxylinoleic acid I–VI, were identified. Tri-unsaturated fatty acids, such as linolenic acid I–IV (C18:3), alongside its hydroxylated form, hydroxylinolenic acid I–VIII, were also noted (Table 3 and Table S1A). It is noteworthy that the short-chain fatty acids of A. platensis were studied for the first time. 

Furthermore, 1-O-palmitoyl-3-O-(6-sulfo-6-deoxy-α-D-glucopyranosyl)-L-glycerolwas identified as a sulfur component in SP (Table 1 and Table S1), exhibiting dehydration and decarboxylation before revealing the sulfodeoxy glucosyl part [50]. Moreover, SP contained five terpenoid derivatives: peyssonoic acid B (sesquiterpene hydroquinone), variabilin (sesterterpene), and etretinate and dihydroetretinate I–II (retinoids) (Table 3 and Table S1). Terpenoid compounds like peyssonoic acid B and variabilin have been previously discovered in marine sponges from the Irciniidae family (Balansa et al., 2010) and the red macroalga *Peyssonnelia* sp. [51].

### 2.9. Identification of Active Metabolites and Correlated Biological Target Proteins

The twenty-five most significant active metabolites of SP were selected. Table S2 presents their PubChem IDs, SMILES, and 2D structure files (SDFs). The Binding DB service was employed to predict the biological targets of these metabolites. Furthermore, the metabolites were screened using ADMET Lab 2.0 based on their drug-likeness (QED > 0.3) and oral bioavailability (Lipinski’s criterion) (Table S3). A total of 24 components and 452 targets were found after these screening procedures.

#### 2.9.1. Identification of BC-Related Genes

**Figure 6:**
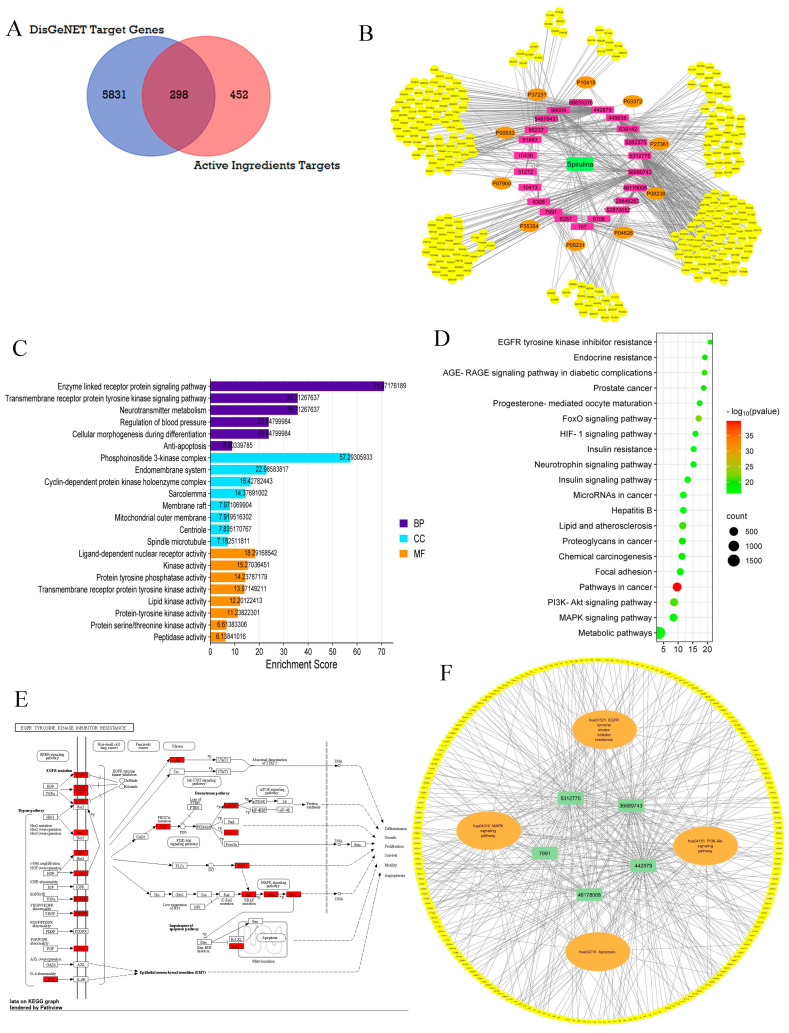
Prediction of SP by network pharmacology for BC treatment. (**A**) Venn diagram of component target and disease target. (**B**) SP-ingredients target network: the green rectangles represent SP, the purple rectangles represent ingredients, the orange circles represent the top 10 targets correlated to BC by the PPI network, and the yellow circles represent the other targets. (**C**) GO enrichment analysis of results for BC treatment of SP. (**D**) KEGG pathway enrichment analysis of results for BC treatment of SP. (**E**) EGFR tyrosine kinase resistance pathway. (**F**) The component–target pathway network.

Following merging with the 5831 BC targets collected from the DisGeNET database by FunRich 3.1.3 software’s Venn diagram intersection, 298 overlapping targets were identified as candidate targets, as depicted in (Figure 6A) (Table S6). The findings underscore the significance of 298 common genes deemed promising targets for cancer treatment and potentially modulated by SP ingredients. Ultimately, twenty-three SP ingredients were found to be associated with these intersected targets as active ingredients.

#### 2.9.2. PPI Network Building and Evaluation 

Using the String database, the species “human” was chosen to create a network graph with 296 nodes and 3380 edges (Supplementary Figure S2). The top 10 genes were found (Figure 6B) (Table S7) using the TSV file downloaded from this website and the cytoHubba plug-in cytoscape. These genes included IL6, EGFR, BCL2, PPARG, HSP90AA1, ESR1, MAPK3(ERK), PTGS2, HSP90AB1, and ERBB2. Three essential core genes—EGFR, BCL2, and MAPK3—in the PPI network of breast cancer-related targets of SP were among the top ten genes. These genes interact and cooperate together to prevent the growth of breast cancer. As the central genes governed by the network, they are crucial for the management of breast cancer. 

#### 2.9.3. GO and KEGG Pathway Analysis of Candidate Targets

Funrich and Shiny GO 0.77 were used to perform GO enrichment and KEGG enrichment analysis on 298 potential targets. Table S7 and Figure 6B illustrate the three components of GO enrichment analysis: molecular function (MF), cellular component (CC), and biological process (BP). As shown in Figure 6B, key BPs, CCs, and MFs were selected for each aspect of analysis based on enrichment scores of more than 5 and *p*-values less than 0.05.

BP analysis unveiled the involvement of targets in various signaling pathways, including those associated with the enzyme-linked receptor protein signaling pathway, transmembrane receptor protein tyrosine kinase signaling pathway, cellular morphogenesis during differentiation, apoptosis, and others. Furthermore, CC analysis revealed associations with the PI3K complex, endomembrane system, and cyclin-dependent protein kinase holoenzyme complex, among others. According to the MF data, the targets were predominantly engaged in ligand-dependent nuclear receptor activity, kinase activity, and protein tyrosine phosphatase activity. Furthermore, the KEGG enrichment analysis identified the top 20 pathways ranked by *p*-value (*p* < 0.05), depicted in a bubble diagram in (Figure 6C) (Table S9). The PI3K-Akt signaling pathway, MAPK signaling pathway, pathways in cancer, HIF-1 signaling, and EGFR tyrosine kinase inhibitor resistance stand out as the most relevant among the various breast cancer-related signaling pathways covered by these pathways. The PI3K-Akt signaling system largely controls protein synthesis, glycolysis, and gluconeogenesis throughout metabolism, cell cycle, and apoptosis, as seen in Supplementary Figure S3 obtained from KEGG. The EGFR tyrosine kinase resistance pathway, another crucial pathway identified, plays a significant role in differentiation, growth, proliferation, survival, motility, and angiogenesis, as shown in Figure 6E. 

It was noted that the coleader PI3K of the PI3K-Akt signaling pathway, along with the three critical core genes identified through PPI analysis (EGFR, MAPK3(ERK), and BcL-2), were related to the EGFR tyrosine kinase resistance pathway. This highlights the biological processes and molecular functions associated with these pathways, as shown in Figure 6E.

#### 2.9.4. Component–Target Pathway Network

Target protein interactions with important breast cancer-related pathways were further explored and visualized by the creation of the component–target pathway network. The participation level of the top five compounds (Table S10) was taken into consideration while employing the prior KEGG enrichment analysis (Table S10). Compounds, targets, and pathways were connected to create the component–target pathway network. This network highlighted the significance of four pathways, EGFR tyrosine kinase inhibitor resistance (hsa01521), the PI3K-Akt signaling pathway (hsa04151), MAPK signaling pathway (hsa04010), and apoptosis (hsa04210) in BC, in correlation with the highly ranked compounds, including hinokinin, 442879, hydroxylinolenic acid, 5312775, N-valeryl-valine, 36689743, peyssonoic acid B 46178008, and valeric acid, 7991 (Figure 6F).

#### 2.9.5. Molecular Docking Analysis 

**Figure 7:**
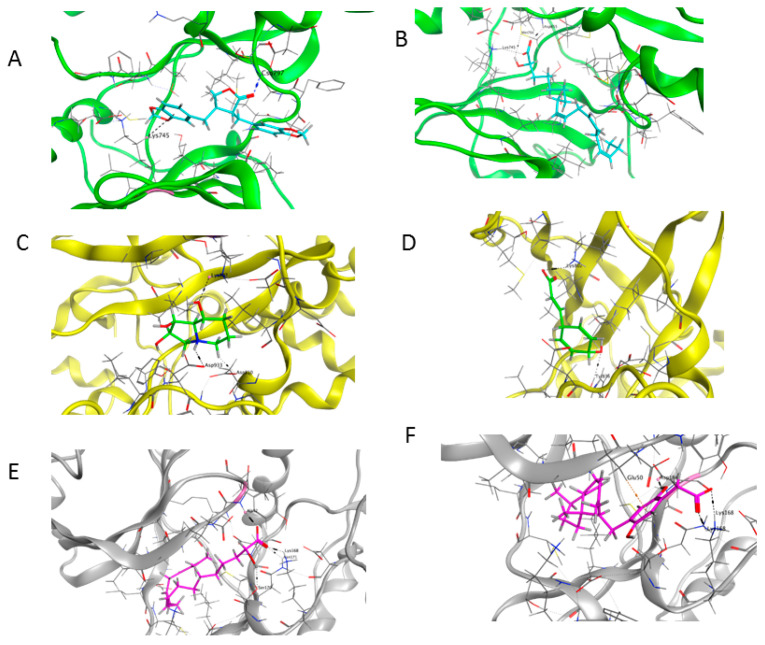
Three-dimensional representation of the most potent compounds against target enzymes: (**A**) hinokinin, 442879/EGFR (Green), (**B**) hydroxylinoleic acid II, 5312775/EGFR (Green), (**C**) swainsonine, 51683/PI3K (Yellow), (**D**) p-dihydrocoumaric acid, 129846263/PI3K (Yellow), (**E**) hydroxylinoleic acid II, 5312775/MAPK(ERK) (Gray), and (**F**) peyssonoic acid B, 46178008/MAPK(ERK) (Gray).

Three core targets (EGFR, PI3K, and MAPK3(ERK)) closely linked to the occurrence and development of breast cancer were chosen for a virtual screening docking simulation study against twenty-four active ingredients of SP in order to further validate the active ingredients and their potential targets and mechanisms in the treatment of BC from SP. This selection was based on the PPI network, KEGG pathways, GO enrichment, and component–target pathway network analyses using MOE software. The results reveal that nine active ingredients exhibit interactions with the core targets, including the previously mentioned top five compounds, hinokinin, 442879, hydroxylinoleic acid II, 5312775, N-valeryl-valine, 36689743, peyssonoic acid B, 46178008, and valeric acid, 7991, as well as four promising candidates [swainsonine, 51683), p-dihydrocoumaric acid, 129846263, dihydrocinammic acid, 107, and chromequinolide, 162870052 (Table S11). Among these, the strongly bound active components of EGFR were hinokinin, 442879 and hydroxylinoleic acid II, 5312775 with binding scores of −6.2373 kcal/mol and −6.1899 kcal/mol, respectively. Both compounds exhibited substantial interactions with important amino acid residues, as illustrated in Table 4 and Figure 7A and Figure 6B. Hinokinin (442879) demonstrated a significant hydrogen bonding interaction with the CSO797 residue, which represents the EGFR mutation that causes resistance to standard therapy routes (Table 4) (Figure 7A). Regarding PI3K activity, compounds swainsonine (51683) and p-dihydrocoumaric acid (129846263) showed the greatest binding scores of −5.3383 kcal/mol and −4.8623 kcal/mol, respectively. Swainsonine (51683) was bound to the key amino acid residues Lys802, Asp933, and Asp810, with four bonds, including three hydrogen bonds and a single ionic bond, while p-dihydrocoumaric acid (129846263) displayed three interacting bonds with the key residues Lys802 and Tyr836, comprising two hydrogen bonds and one ionic bond (Table 4) (Figure 7C,D). Finally, an examination of the activity against MAPK3 (ERK) revealed that hydroxylinoleic acid II (5312775) and peyssonoic acid B (46178008) exhibited the best interactions, with binding scores of −4.9854 kcal/mol and −4.8738 kcal/mol, respectively. Hydroxylinoleic acid II (5312775) formed four hydrogen bonds with the active site residues Ser170, Asn171, Lys168, and Ala52, while peyssonoic acid B (46178008) connected with Asp184, Lys168, and Glu50 through three hydrogen bonds and one hydrophobic contact, as revealed in Table 1 (Figure 7E,F).

### 2.10. Effect of SP on Phosphorylated and Total EGFR, AKT, and ERK1/2 

**Figure 8:**
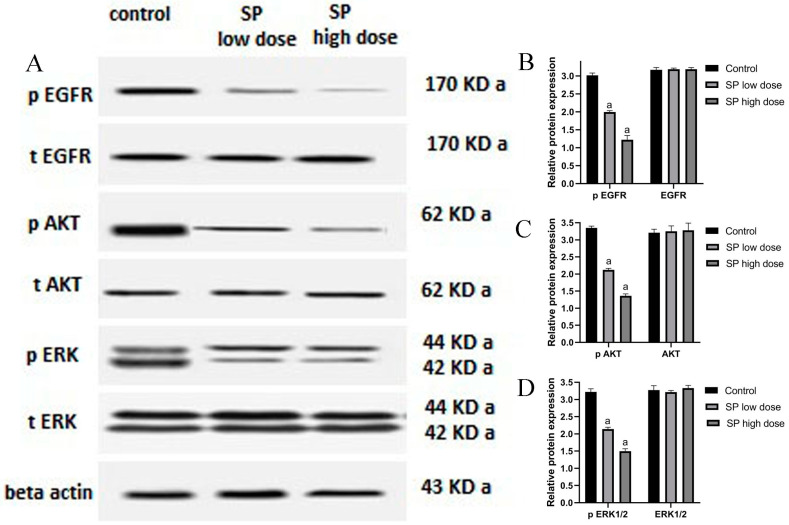
SP modulates MAPK and PI3K/Akt/EGFR signaling pathways in MCF-7 cells. In MCF-7 cells treated with low (10 μg/mL) and high (25 μg/mL) doses of SP for 24 h, the expression levels of p-EGFR, EGFR, p-AKT, AKT, p-ERK1/2, and ERK1/2 proteins are shown in the Western blot image in (**A**). Remarkably, the overall protein concentrations of EGFR, AKT, and ERK1/2 did not change with therapy. As a function of SP concentration, the relative protein expression levels of p-EGFR, EGFR, p-AKT, AKT, p-ERK1/2, and ERK1/2 are shown in (**B**–**D**). The control group consisted of untreated cells grown in their growth medium. The presentation of all data is as mean ± SEM (n = 3). After one-way ANOVA, Tukey’s post hoc analysis was carried out. ^a^ *p* < 0.05 was obtained when compared to control cells.

To confirm the results of the bioinformatic research, Western blot analysis was performed (Figure 8). MCF-7 cells were exposed to concentrations of 10 and 25 μg/mL of SP for 24 h. Western blot analyses revealed that, whereas the total protein levels of EGFR, AKT, and ERK1/2 did not change after treatment with SP, the levels of phosphorylated EGFR, AKT, and ERK1/2 did. Remarkably, cells subjected to a 25 μg/mL dose of SP showed much higher expression levels than cells treated to a lower concentration of SP.

## 3. Discussion

The study comprehensively investigated the anticancer properties of SP extracts utilizing a network pharmacology approach, DMBA-induced mammary gland carcinogenesis, and the MCF-7 cell line, representing human breast adenocarcinoma expressing the estrogen receptor. The use of DMBA-induced mammary carcinoma in rats serves as a well-established preclinical animal model to assess the efficacy of chemopreventive strategies for BC. DMBA is commonly employed to induce mammary gland cancers in animals [3,52]. BC observed in rats closely resembles hormone-dependent BC in humans, making it a relevant model for disease investigation [3]. Moreover, the histopathological changes and abnormal proliferation observed in the DMBA-induced mammary carcinoma model closely mimic the progression of pre-cancerous and malignant abnormalities observed in clinical cases of BC [4,53]. The main goal of this study was to examine early histopathological alterations in BC progression by administering DMBA to female rats. Studies suggest that DMBA induces lesions in the terminal ducts, leading to an abnormal proliferation of ductal epithelial cells. Excessive growth can give rise to the development of abnormally hyperplastic cells, which may progress into ductal carcinoma [4,54]. Rats exposed to DMBA had their breast glands examined, and both malignant and benign tumors developed in addition to many precancerous stages. Remarkably, the administration of SP significantly attenuated the incidence of tumor alterations compared to the DMBA-treated group, highlighting its potential as a therapeutic intervention for BC.

The pivotal role of oxidative stress in driving cellular changes and damage in BC has been extensively documented [55,56]. The metabolism of DMBA in breast tissue leads to the generation of free radicals. These radicals damage DNA and other biomolecules in the mammary gland and decrease the body’s natural antioxidant defenses. Consequently, this cascade initiates the development of BC [57]. The presence of the carcinogen DMBA elicited several markers of oxidative stress in the breast tissues of the experimental animals. Specifically, indicators measuring the oxidative degradation of proteins and lipids, namely P. carbonyl and MDA, were utilized. These markers have been previously investigated in a DMBA model by Karnam et al. [58], Kumar et al. [59], and Hamza et al. [56]. Consistent with prior research utilizing the DMBA model, it was demonstrated that rats treated with DMBA exhibited a significant decrease in the levels of endogenous antioxidants, including SOD, CAT, and TAC, in their mammary tissues. These findings indicate a deficiency in antioxidant levels in DMBA-treated rats. However, administering SP to DMBA-treated rats effectively restored the activity of TAC, CAT, and SOD in breast tissue, thereby mitigating the oxidative breakdown of proteins and lipids (P. carbonyl and MDA). These results suggest a strong correlation between the anticancer efficacy of SP and its antioxidant properties. Furthermore, the FRAP experiment conducted in this study provided evidence of SP’s substantial ability to undergo a reduction, thus confirming its antioxidant properties. The antioxidant properties of the SP suspension can be attributed to phenolic constituents, which constituted 43.84% of the suspension. Various phenolic compounds, including *p*-dihydroxycinnamic acid, *p*-dihydrocoumaric acid, hinokinin, 6-propionyllumazine, and peyssonoic acid B, contribute to the antioxidant activity of SP. Moreover, recent research has demonstrated that SP can alleviate liver oxidative stress in a mouse model of hepatocellular carcinoma, further highlighting its potential as an anticancer therapy [33].

After rats were administered DMBA, which caused oxidative stress and the production of ROS, the rate of cell proliferation in the rats’ breast tissues increased dramatically. The number of PCNA-positive cells in the breast tissues of rats administered DMBA was greater, suggesting a higher rate of cell division. DNA polymerase’s essential cofactor PCNA has been linked to both the advancement of BC and an increase in the proliferation of malignant cells [60]. According to studies by Laskar et al. [3] and Maru et al. [61], the lack of apoptotic mechanisms in cancer cells causes increasing growth and increased cell proliferation. As an antioxidant, SP significantly reduced the proportion of PCNA-positive cells in rats treated with DMBA, suggesting a potent inhibitory effect on cell division. Furthermore, a connection between the start of apoptosis and SP-induced cell growth suppression is shown by the rise in TUNEL-positive cells, which are indicative of apoptosis. These findings are consistent with the research that demonstrates MCF-7 cell cycle arrest and apoptotic induction. Rats treated with DMBA showed increased TUNEL-positive cell apoptosis in their mammary glands as compared to untreated rats. According to Ehemann et al. [62], regions with low blood perfusion may be responsible for the observed apoptotic effect. On the other hand, PCNA levels declined as the frequency of TUNEL-positive cells increased in rats administered both SP and DMBA, suggesting cellular death. These findings imply that SP affects rat mammary glands in two ways: it both prevents cell division and triggers programmed cell death. Given the concurrent rise in TUNEL-positive cells and drop in PCNA levels, SP may induce apoptosis and modify the cell cycle. This could reduce the number of potentially cancerous cells and stop the growth of numerous tumors by controlling cell division and promoting cell death. 

Rats administered DMBA treatment showed increased PCNA production, ER-α expression, and cell proliferation in their mammary glandular tissues. These results imply that the rate of cell division may be affected by estrogen. Numerous studies on people and animals have demonstrated the importance of ER-α for the formation of BC and the rapid expansion of cancer cells [63,64]. Accordingly, the estrogen receptor (ER) is the specific target of contemporary BC medications [3,63]. The results of this investigation show that rats administered SP and DMBA express significantly less PCNA and ER-α. These results are consistent with a study by Ouhtit et al. [36], which found that the cell proliferation markers Ki-67 and ER-α were considerably reduced when SP was added to breast tumors formed by DMBA. The primary benefits of employing SP in the fight against cancer lie in its ability to reduce oxidative stress, inhibit cell proliferation, promote apoptosis, and obstruct ER-α activity.

The current study indicates that the ethanolic extract of SP has potent cytotoxicity against breast cancer. The suppression of MCF-7 and MCF-7/ADR cell growth are dose-dependent, with the degree of repression increasing. According to Ouhit et al. [36], Jiang et al. [26], Fayad et al. [27], Najem et al. [65], and other studies, the findings of this investigation are compatible with those of earlier investigations. These findings demonstrate that exposure to active components present in SP extracts induces apoptosis in BC cells, such as MCF-7 or MDA-MB 231 cells. The analysis using flow cytometry demonstrates that SP efficiently halts the cell cycle and promotes apoptosis in MCF-7 cells, hence halting cell growth at the G2/M phase. These findings suggest that apoptosis is the reason asbestos inhibits MCF-7 cell growth.

The delivery of SP set off apoptosis, which correlates with the expected increase in ROS. In the mitochondria-mediated apoptotic pathway, ROS are important regulators of the pro-apoptotic protein Bax and the anti-apoptotic protein Bcl-2. Bax promotes the disruption of mitochondrial membranes, facilitating apoptosis [66,67], while Bcl-2 protects mitochondrial integrity, inhibiting apoptosis [68,69]. Additionally, Bax aids in the release of cytochrome c, which triggers caspase-9 activation and the intrinsic pathway’s start of apoptosis [68]. Following SP delivery, our research showed a substantial drop in Bcl-2 levels and an increase in cytochrome c, caspase-8, caspase-9, caspase-7, and Bax levels. These findings imply that the mitochondrial apoptotic pathway is the mechanism by which SP administration causes MCF-7 cell apoptosis. Prior studies have shown that SP extracts can induce the death of BC cells through mitochondrial-mediated apoptosis. This effect may be associated with the generation of ROS by the extracts, as reported by previous research [26,27,36,65].

The metabolic profile of the hydroalcoholic extract of SP led to the identification of 67 components. These phytochemicals were categorized as phenolic derivatives, nitrogenous compounds, fatty acids, sulfur compounds, and terpenes, with detailed annotations. By synthesizing data from previous research and publicly accessible sources, we explored the interactions between SP compounds and potential protein targets in BC. Our analysis also uncovered additional signaling pathways and networks, where SP metabolites interact with targets effective against BC. Utilizing network pharmacology analysis, 23 specific compounds with potential anti-breast cancer properties were identified. These compounds encompass alkaloids (6-propionyllumazine and swainsonine), phenolic derivatives (dihydrocinammic acid, *p*-dihydrocoumaric acid, hinokinin, chromequinolide, and 2,2′-Bis(4-methyl6-tert-butylphenol) methane), and fatty acids (Hydroxybutanoic acid I, valeric acid, isovaleric acid, hydroxyvaleric acid, and hydroxylinoleic acid). Additionally, active ingredients like dihydrocoumarin [70], peyssonoic acid B [51], hinokinin [71], cinnamic acid derivatives [72], valeric acid [73], and α-linolenic acid [74] have demonstrated an effective inhibition of BC cell growth by influencing epigenetic modifications, triggering apoptosis, and inducing cell cycle arrest. Among these compounds, our extract contains swainsonine, an indolizidine alkaloid known for its anti-tumor properties. Swainsonine has been demonstrated to inhibit the PI3K/AKT/mTOR signaling pathway, leading to a reduction in the proliferation and increase in the death of human cancer cells [75,76]. Through meticulous selection, a specific group of genes directly associated with BC as prospective targets for SP treatment was identified. Consistent with prior research, our KEGG pathway enrichment analysis highlighted the significance of the EGFR, PI3K-AKT, and MAPK signaling pathways. These pathways serve as potential mechanisms through which SP influences various cellular activities, including cell division, proliferation, differentiation, inflammation, metabolism, apoptosis, and cellular stress response. Our research identified eight proteins crucial for SP compounds’ activity in BC, including ERBB2, IL6, EGFR, BCL2, PPARG, HSP90AA1, ESR1, MAPK3, PTGS2, and HSP90AB1.

Through the construction of a protein–protein interaction (PPI) network, several key molecules in SP, including hinokinin, hydroxylinolenic acid, valeryl-valine, peyssonoic acid B, valeric acid, swainsonine, *p*-dihydrocoumaric acid, dihydrocinammic acid, and chromequinolide, were identified This network integrates various components, targets, and pathways, showcasing their interconnectedness. These compounds exhibit selective inhibitory effects on four crucial pathways: EGFR, PI3K-AKT, MAPK, and apoptosis. Utilizing the PPI network, KEGG pathways, GO enrichment, component–target pathway network, and literature research, MAPK3, EGFR, and PI3K as the primary targets for molecular docking studies were identified. Subsequently, molecular docking analyses were conducted to elucidate the binding modes, providing insights into the interactions between the main components of SP and their respective targets. The results reveal significant binding activity between nearly all primary constituents and their respective targets, highlighting their potential efficacy in modulating the targeted pathways involved in BC. 

Subsequent in vitro investigations revealed a significant reduction in the expression levels of p-EGFR, p-AKT, and p-ERK1/2 proteins, underscoring the pivotal functions of EGFR, PI3K/Akt, and MAPK signaling pathways in SP’s anti-breast cancer properties. This finding aligns with previous research demonstrating that SP inhibits Akt phosphorylation to prevent A549 cell death [29]. Furthermore, by reducing p-ERK1 levels in MDA-MB-231 cells, C-Phycocyanin, the active ingredient in SP, has shown anticancer effects [26]. BC cells’ motility, proliferation, adhesion, and capacity for invasion are all influenced by the interaction between ERs and the EGFR signaling pathway [77]. Upregulated EGFR expression in BC patients is associated with increased tumor development and resistance to apoptosis, indicating a more aggressive disease phenotype [77]. The three primary MAPK pathway subtypes are p38, JNK/SAPK, and ERK1/2. Each of these subtypes has a distinct role in a different cellular response [78,79]. Important BC features have been connected to the MAPK signaling system, which is implicated in tumor growth, differentiation, and programmed cell death [80]. According to studies by Lucas et al. [81], blocking the ERK signaling pathway causes a rise in cellular mortality and a fall in proliferation. The PI3K/Akt/mTOR pathway is an essential signaling cascade that regulates several cellular processes, including migration, autophagy, apoptosis, and proliferation [82,83]. Studies by Tewari et al. [83] and Yu et al. [84], which highlight the use of inhibition as a strategy to minimize metastasis, particularly in triple-negative and ER-positive BCs, demonstrate the significance of this pathway in BC. Gil [85] claims that the overactivation of the PI3K/Akt/mTOR pathway can promote tumor growth and result in resistance to treatment. This study provides compelling evidence that SP suppresses MCF-7 growth and increases apoptosis by targeting the EGFR, PI3K/Akt, and MAPK signaling pathways.

## 4. Materials and Methods

### 4.1. Chemicals 

The Spirulina powder, which comes from Spirulina platensis, was made by spray-drying. This excellent dark-blue–green powder is called 100 Organic Spirulina Powder, obtained from Vegan Superfood, Lot No. G1710120, China. This Spirulina powder is a nutrient-dense superfood because it is 100% organic. The components of the Spirulina used in our study were proteins (61%), carbs (24%), lipids (12%), fibers (3.7%), vitamins A (11%), A (16%), and B6 (20%), calcium (12%), and magnesium (48%). Fetal bovine serum (FBS), streptomycin, Dulbecco’s Modified Eagle Medium, 2-[4-(2-hydroxyethyl) piperazin-1-yl] ethane sulfonic acid (HEPES), glutamine, and penicillin were supplied by Gibco/Invitrogen (Karlsruhe, Germany). The 3-(4,5-dimethylthiazol-2-yl)-2,5-diphenyltetrazolium bromide (MTT) used in the MTT test kit for in vitro toxicity was provided by Sigma (Sigma, M-5655). The Apoptosis Detection Kit was used for Annexin V-FITC (K101-25). This kit was created by BioVision Research Products, 980 Linda Vista Avenue, Mountain View, CA 94043, USA. The experiment’s materials, which included DMBA, epinephrine, superoxide dismutase (SOD) enzyme, H_2_O_2_, thiobarbituric acid, and bovine albumin, were supplied by the St. Louis, MO, USA, Sigma Chemical Co. The other chemicals were obtained from local commercial providers.

### 4.2. Preparation and Purification of Extracts from SP 

The extraction process, which was adapted with slight modifications from a previous work, included blending and vigorously mixing each 100 g sample of powder with a 70:30 *v*/*v* ethanol:water mixture. The resulting combination was sonicated using a Branson (Danbury, CT, USA) B3510 sonicator for thirty min. The sample was sonicated and then allowed to sit at room temperature for 60 min while being agitated with a magnetic stirrer. The sample was then centrifuged, and the residual materials were extracted once more utilizing the technique that was previously described. The extracts were collected and then evaporated using a Rotavapor R-200 (Büchi Labor Technik, Flawil, Switzerland) at 38 °C. For use in animal experiments, the dried leftovers were dissolved in water, and for LC-MS analysis, in methanol. After that, the samples were kept at −20 °C until they could be examined further.

### 4.3. In Vivo Study 

#### 4.3.1. Animals

Female Wistar albino rats, 35–40 days old and 55–60 g in weight, were acquired from the Animal House of the National Research Center (Giza, Egypt). The rats had unlimited access to tap water and were fed a regular pellet diet. They were housed in polycarbonate cages with bedding made of wood chips, and they experienced a 12 h light/dark cycle at an ambient temperature of 22–24 °C. The rats were given two weeks to adjust to their new surroundings before the experiment began. Every effort was made to keep the animals as comfortable as possible during the study. The studies were conducted in accordance with the 1993 guidelines for the use and care of laboratory animals set out by the Canadian Council for Animal Care. Moreover, the trials fulfilled the ethical standards for animal care in research as set out by the NODCAR Animal Ethics Committee (protocol number: NODCAR/1/11-2023).

#### 4.3.2. Dosage Information 

Mammary gland tumors were induced in 55-day-old prepubertal rats after a single oral dosage of DMBA (100 mg/kg b.wt.) dissolved in 5 mL of sesame oil. The DMBA dosage was calculated using the methods described in [52,56,86]. The dried extract of SP was dissolved in 5 mL of deionized water, well-mixed, and sonicated in an ice-cold ultrasonic bath for 15 min. A daily dose of 1000 g/kg of SP for 22 weeks was chosen based on the pharmacological and toxicological features outlined in previous papers. When provided at doses ranging from 500 to 1500 mg/kg, SP has been shown to prevent liver cancer [32], oxidative stress, and toxicity [87,88,89]. According to Liu and Cao [90], the administration of 1500 to 4500 mg/kg of SP to rats over 90 days resulted in no adverse effects. Individual dosages of 1000 mg/kg b.wt./day were comparable to doses of 162 mg/kg b.wt./day for people. This computation used the approach for translating a body surface area-based dosage into an equivalent human dose [91]. The animal dosage (mg/kg × the animal’s Km factor/the human Km factor) is the formula for calculating the human dose equivalent. Our dosage fell within the range of 1–19 gm/day, the recommended daily SP intake for humans [92]. 

#### 4.3.3. Treatment Regime

The rats were divided into four groups at random, each with six members (n = 6), and would then participate in the following experiments. The control-group rats received a daily oral dose of 5 mL/kg b.wt of distilled water for 22 weeks. After a week, they were administered an oral dose of 5 mL/kg b.wt. of sesame oil. Rats in the SP group were administered an oral dosage of 1000 mg/kg b.wt. of SP for 22 weeks, followed by a single dose of 5 mL/kg b.wt. of sesame oil after a week. After one week of daily oral doses of distilled water for 22 weeks, a single oral dosage of DMBA dissolved in sesame oil (100 mg/kg b.wt.) was administered to the rats in the DMBA group to induce BC. Rats in the SP and DMBA groups were administered a single oral dosage of DMBA dissolved in sesame oil (100 mg/kg b.wt.) to induce BC following 22 weeks of daily SP treatments (1000 mg/kg b.wt.). The rats were terminated by cervical dislocation following the administration of a total dosage of 3% sodium pentobarbital (45 mg/kg, i.p.) after 22 weeks. This allowed for the collection of organ samples from every rat in the four groups.

#### 4.3.4. Sample Preparation

Following euthanasia, the thoracic and abdominal skin, as well as the underlying mammary glands (six on each side), were bluntly separated from the muscles that create the thoracic and abdominal walls. The mammary glands and surrounding skin were dissected, with some of the glands fixed in 10% buffered formalin for histopathological study and the rest processed for biochemical and oxidative stress analyses.

Some mammary glands were swiftly dehydrated, rinsed in an ice-cold normal saline solution, dried, and snap-frozen in liquid nitrogen for later biochemical analysis. Eight mammary glands from each rat were promptly preserved in 10% buffered formalin and fixed in paraffin for the histological examination. A portion of the breast tissues was ground up in an ice-cold 150 millimolar Tris-HCl buffer solution (1 part by weight to 10 parts by volume) to quantify oxidative stress indicators (pH 7.4). Then, using appropriate dilutions in different buffers as needed, the levels of malondialdehyde (MDA), protein carbonyl (P. carbonyl), total antioxidant capacity (TAC), and the activity of catalase (CAT) and superoxide dismutase (SOD) in breast tissues were measured.

#### 4.3.5. Determination of Oxidative Stress Markers

MDA was measured in the mammary glands to determine lipid oxidation [93], following the method reported by Gerard-Monnier et al. [94]. This includes the interaction of MDA and N-methyl-2-phenylindole, which results in a blue molecule with a maximum absorbance of 586 nm. A solution of 10 mM N-methyl-2-phenylindole in acetonitrile and methanol (3:1) was combined with 200 µL of breast tissue. The reaction began with the addition of 150 µL of pure HCl (37%). After an hour of incubation at 45 °C, blue absorbance was measured at 586 nm and we reported the results in nmol of MDA per mg of protein. 

P. carbonyl in the mammary glands was assessed using an established method that involved its interaction with 2,4-dinitrophenyl hydrazine (DNPH) to create protein hydrazones, which were then detected at 370 nm [95]. The protein pellets were centrifuged for 3 min and then combined with 10 mM DNPH and 2 N HCl. To precipitate the protein, 0.5 mL of breast tissue was treated with 20% trichloroacetic acid. Samples were stored at room temperature for an hour, with vortexing every 10 min, before being treated with 20% TCA and centrifuged for 5 min. To eliminate unbound DNPH, protein pellets were washed with 20% TCA, then three times with ethanol and ethyl acetate. Pellets were immersed in 6 M of guanidine hydrochloride for 15 min at 37 °C before being dissolved in 2 N HCl or 20 mM phosphate buffer (pH 2.3). The results were reported as nmol of carbonyl group per mg of protein, with a molar extinction coefficient of 22,000 M/cm. 

The ferric reducing antioxidant power (FRAP) test, developed by Benzie and Strain [96], was used to assess TAC in mammary homogenates. The FRAP reagent consisted of 2,4,6-tripyridyltriazine (TPTZ), FeCl_3_·6H_2_O (20 mM), and an acetate buffer. Three mL of FRAP reagent was added to 50 µL of samples, and the blue hue’s absorbance was measured at 593 nm for six min. The hue shift generated by antioxidants’ electron-donating properties was measured using ascorbic acid. 

The SOD level in breast tissue was evaluated using Nandi’s and Chatterjee’s method, which is based on SOD’s ability to inhibit pyrogallol oxidation in alkaline circumstances [97]. A breast sample of 20 µL was combined with 2.9 mL of 50 mM Tris-cacodylate buffer (pH 8.5) and 100 µL of 2.6 mM pyrogallol in 10 mM of HCl. The absorbance was recorded at 420 nm for two min. One unit of SOD is required to inhibit pyrogallol auto-oxidation by 50%, with results expressed in units per mg of protein.

Aebi [98] described the method for measuring CAT activity in mammary tissues by measuring the fast reduction of H_2_O_2_ at a wavelength of 240 nm and reporting the results in units per milligram of protein.

The Lowry technique, modified by Peterson [99], was used to determine the total protein content in mammary glands. The Shimadzu recording spectrophotometer (UV-160) was utilized to quantify absorbance in all recorded data.

#### 4.3.6. Histopathological Examination

H&E sections were examined using a Leica DMRB/E light microscope (Leica, Deerfield, IL, USA) to detect histopathological abnormalities in the mammary glands and classify BCs according to histological and cytological criteria [100]. There are three types of hyperplastic lesions: ductal or lobular hyperplasia, fibroadenoma, and ductal or lobular carcinomas in situ. These classifications were made based on the observed proliferation and cellular atypia levels.

#### 4.3.7. PCNA and ER Immunohistochemical Analyses

Deparaffinization of the tissue slices from the mammary glands of all groups was carried out by first rehydrating them with a series of graded alcohol treatments, and then by washing them three times in xylene. Three five-minute washes in PBS pH of 7.4 with 0.05% Tween 80 were performed on the slides in sequence. The slides were subjected to microwave irradiation for 30 min in a solution containing 0.01 M of sodium citrate (pH of 6.0) to accelerate antigen recovery. Heat treatment in a solution of 1% H_2_O_2_ in methanol was used to limit the activity of naturally occurring peroxidase on the slides. Submerging the specimens in a standardized goat serum for 25 min prevented non-specific binding. The slides were then treated with 1:100 of the anti-ER-antibody (Santa Cruz Biotechnology, Dallas, TX, USA) or 1:1000 of the anti-PCNA antibody (Abcam, Cambridge, MA, USA). At room temperature for one h, the slides were treated with streptavidin–biotin peroxidase (Dako, Carpinteria, CA, USA) and an anti-rabbit/mouse biotin-labeled secondary antibody after being immersed in Tris-buffered saline (TBS). A solution comprising 0.05% imidazole, 0.03% hydrogen peroxide, and 0.05% 3,3-diaminobenzidine tetrahydrochloride in a Tris HCL buffer (pH 7.6) was used to create a chromogenic peroxidase substrate. A counterstain called hematoxylin was applied to the sections. A Leica DMRB/E light microscope was used to look at the slides and count how many ER-positive cells and PCNA were in the lobules and ducts. The process involved counting 400–1000 cells in five fields of each slice to achieve this. Thus, at least six rats were randomly chosen for each group, and six samples of tissue were collected from each rat. To find out how many cells showed positive staining for PCNA and ER, these samples were analyzed.

#### 4.3.8. Apoptosis Analysis Obtained via TUNEL

Apoptosis was discovered in deparaffinized mammary gland slices using the TUNEL method. The ApopTag Plus Peroxidase In Situ Apoptosis Detection Kit (S7101, Sigma-Aldrich Pty Ltd., an associate of Merck KGaA, Darmstadt, Germany) was used according to the manufacturer’s instructions. The TUNEL method employed terminal deoxynucleotidyl transferase to label 3-OH DNA ending with digoxigenin nucleotides. This approach enables the detection of DNA fragmentation linked to apoptosis. For the enzymatic reaction, a suitable amount of peroxidase substrate was applied. For studying TUNEL slides, we used the Leica DMRB/E light microscope. TUNEL-positive cells exhibited brown, tightly packed nuclei. To measure the fraction of cells displaying a positive TUNEL expression, 1000 cells were examined and counted across five fields in each slice. Six rats were selected for each group, and six tissue samples were randomly obtained from each rat.

### 4.4. Quantification of the Total Phenolic Content in SP 

The total phenolic content of the SP extract was determined using the Folin–Ciocalteau reagent, following the technique published by Singleton et al. [101]. A solution was created by mixing 0.5 mL of Folin–Ciocalteu reagent with 2 mL of distilled water, and then adding 1 mL of the extract, which had a concentration of 1 mg/mL. After incubation at room temperature for 3 min, one mL of 20% (*w*/*v*) sodium carbonate solution was added to the mixture. The absorbance was measured at a wavelength of 765 nm using a UV/Vis spectrophotometer after one hour. A standard curve was established using gallic acid and was subsequently employed to measure the total phenolic content. The total phenolic content was determined by calculating the amount of gallic acid equivalent per gram of dry weight of plant material.

### 4.5. Quantification of the Total Antioxidant Capacity of SP 

The ferric reducing antioxidant power (FRAP) test was utilized to evaluate the total antioxidant capacity (TAC) of SP. The FRAP test was performed using the protocol described by Benzie and Strain [96].

### 4.6. Analysis of Biologically Active Substances Using Reversed-Phase High-Performance Liquid Chromatography Coupled with Mass Spectrometry and Tandem MS/MS (RP-HPLC-ESI-MS and MS/MS)

To improve the interpretation of biological response data, the extract of SP was analyzed using RP-HPLC-ESI-MS and MS/MS. This analysis aimed to identify the biologically active metabolites or potential biomarkers responsible for anticancer activities. The analyses were performed with an Agilent 1200 series quick-resolution system manufactured by Agilent Technologies in Santa Clara, CA, USA. The equipment consisted of a quaternary pump (G7104C) and an autosampler (G7129A). The volume of the injection was five microliters. The substances were isolated using Poroshell 120 HiLiC Plus (150 mm × 3 mm, 2.7 μm particle size) (Agilent Technologies) [38,45]. The utilized flow rate was 0.3 mL per minute. The system was connected to a 6530 Agilent Ultra-High-Definition Accurate-Mass Q-TOF LC/MS instrument using an electrospray (ESI) interface. LC/MS was configured to operate in negative and positive ionization modes. The data analysis was performed using MassHunter Qualitative Analysis B.06.00, which Agilent Technologies supplied [40,46]. The analysis encompassed the generation of potential molecular formulas, the observation of retention times (RTs), molecular ion peaks (*m*/*z*), and fragmentation patterns. The obtained data were cross-referenced with the Reaxys database (https://www.reaxys.com, accessed on 1 June 2024), KNApSAcK Core System database (http://www.knapsackfamily.com/knapsack_core/top.php, accessed on 1 June 2024), PubChem database (https://pubchem.ncbi.nlm.nih.gov/, accessed on 1 June 2024), and Lotus natural products database (https://lotus.naturalproducts.net/, accessed on 1 June 2024). In addition, relevant literature was obtained from the Egyptian Knowledge Bank (https://www.ekb.eg, accessed on 1 June 2024).

### 4.7. Cell Lines and Culture

The human ER-positive breast cancer cell line MCF-7 and its drug-resistant mutant MCF-7/ADR were purchased from the American Type Cell Collection (ATCC, Manassas, VA, USA). The cells were grown in Dulbecco’s Modified Eagle Medium, which was enhanced with 10% fetal bovine serum, 2 mM of glutamine, 100 U/mL of penicillin, 100 μg/mL of streptomycin, and 10 mM of HEPES. The cultures were kept in an environment with 5% CO_2_ at 37 °C and humidity regulated to maintain a pH of 7.4. A total of 90% of the medium was replaced with new medium every day during the cell passage.

#### 4.7.1. Measurement of the Toxicity of SP Using the MTT Test

To evaluate the cytotoxicity of SP, we performed the MTT test on both MCF-7 and MCF-7/ADR cell lines. Cells were placed into a 96-well plate, and 100 μL of SP ethanol extract, which had been diluted in DMSO (0.01%), was added into each well. Cells were exposed to a range of doses of SP and doxorubicin (DOX) (0.4, 1.6, 6.3, 25, and 100 μg/mL) for 24 h. The control group was administered with only DMSO. After the incubation time, a solution containing MTT at a concentration of 0.5 mg/mL was added to each well and left to incubate for an additional 4 h. The formazan crystals were dissolved by introducing 150 μL of dimethyl sulfoxide (DMSO). The measurement of the optical density was performed by determining the absorbance at a specific wavelength of 570 nm using a Bioline Elisa Microplate Reader (BD-R206, Bioline Technology, Maharashtra, India). The experiment was performed in duplicate and replicated three times. The IC50 value, which represents the concentration needed to inhibit 50% of cell proliferation, was used to assess the extract’s effectiveness in preventing cell growth.

#### 4.7.2. Evaluation of Apoptosis

An apoptosis assay was conducted using flow cytometry and the Annexin V technique, following BioVision Research Product’s guidelines (Catalog Number: K101-25, 980 Linda Vista Avenue, Mountain View, CA 94043 USA). Cells at a concentration of 1 × 10^6^ per well were placed in six-well microplates and treated with 100 and 25 μg/mL of SP for 24 h. After treatment, cells were spun for 5 min at 1000 rpm, rinsed with cold phosphate-buffered saline, and resuspended at 1 × 106 cells/mL in binding buffer. Five microliters each of Annexin V-FITC and propidium iodide (PI) were added, followed by 30 min incubation at 37 °C in the dark. After adding 400 μL of binding buffer, samples were analyzed using a Becton Dickinson FACS Calibur flow cytometer. Quadrant analysis identified necrotic cells (Q1), late-stage apoptotic cells (Q2), normal cells (Q3), and early-stage apoptotic cells (Q4).

#### 4.7.3. Analysis of the Cell Cycle

The impact of SP treatment on cell cycle arrangement was analyzed using a Becton Dickinson FACS Calibur flow cytometer and a propidium iodide flow cytometry kit (Abcam, ab139418), following the methodology reported by Xu et al. [102]. BC cells were cultured in 6-well plates at 5 × 10^5^ cells/mL. After 24 h, the medium was replaced with fresh medium containing 10 or 25 µL/mL of SP for another 24 h. Untreated cells were administered DMSO as a vehicle. Trypsin-treated cells were collected, rinsed with phosphate-buffered saline, fixed with 60% ethanol, and stained with a propidium iodide (PI) solution. Cell phase distribution was assessed with the Becton Dickinson FACS Calibur Flow Cytometer, and data from 10,000 cells per sample were processed using BD Cell Quest Pro software (version 5.1).

#### 4.7.4. Quantification of ROS

Cells were grown in 6-well plates and treated with SP at doses of 10 and 25 µg/mL to measure ROS. After 24 h, the control and SP-treated cells were washed twice with PBS. They were then treated with the fluorescent dye DCFH-DA (15 μM, Invitrogen Kit, Thermo Fisher Scientific, Waltham, MA, USA, Catalog Number: 88-5930) at 37 °C for 30 min. After another wash, the cells were trypsin-collected and examined on a Becton Dickinson FACS Calibur Flow Cytometer with 488 nm excitation and 525 nm emission wavelengths. Each sample was measured using 5000 events.

#### 4.7.5. Quantification of Apoptotic Protein Expressions Using the Enzyme-Linked Immunosorbent Assay (ELISA)

The amounts of apoptotic proteins were measured using ELISA colorimetric kits. The proteins analyzed included Bcl-2 (a marker for preventing cell death), BAX, caspases 3, 7, 9, and cytochrome c (markers for cell death). The Bcl-2 ELISA kit was acquired from Invitrogen Zymed^®^ (Cat. No. 99-0042, Invitrogen Corporation, Carlsbad, CA, USA). The BAX ELISA kit was obtained from DRG^®^ (Human, EIA-4487). The caspase-8 and 9 ELISA kits were obtained from DRG^®^ (Casp8 EIA-4863, Casp9 EIA-4860), whereas the caspase-7 kit was obtained from Invitrogen (Invitrogen™ EH71RBX5). The cytochrome c ELISA kit used in this study was sourced from Abcam (Human ELISA Kit ab110215). The procedure adhered to the instructions provided by the manufacturer. The cells were cultivated in Roswell Park Memorial Institute (RPMI) medium, which was enriched with 10% fetal bovine serum and kept in a 37 °C incubator. Cells were distributed evenly in each well of a 96-well plate at the number of cells ranging from 1.2 to 1.8 × 10 ^4^ cells per well. Each of the wells contained 100 μL of complete growth media and 100 μL of SP extract. Cells were incubated for 24 h prior to the experiment. The cells were subsequently disrupted using a cell extraction buffer. The lysate was diluted in Standard Diluent Buffer across the whole test range and assessed for the presence of human active BAX, Bcl-2, caspase-7, caspase-8, caspase-9, or cytochrome c. The experiments were conducted in triplicates.

### 4.8. Assessment of System Pharmacology

#### 4.8.1. Screening of Active Ingredients from SP and Gathering Their Targets 

The major twenty-five active metabolites of SP were chosen that were previously identified via LC-MS and MS/MS. The 2D-structure files (sdf), PubChem IDs, and SMILES of these active metabolites were acquired from the PubChem database https://pubchem.ncbi.nlm.nih.gov/, accessed on 1 January 2024) as reported by Kim et al. [103] (Table S1B). The metabolites’ 2D structure files underwent additional filtering based on their drug-likeness (with a QED value greater than 0.3) and oral bioavailability (Lipinski’s and Pfizer’s rules). This evaluation was performed using ADMET Lab 2.0, a service provided by Xiong et al. [104] (Table S2), which may be accessed at https://admetmesh.scbdd.com/service/evaluation/index, accessed on 10 October 2023. The Swiss target prediction web tool (http://www.swisstargetprediction.ch, accessed on 1 January 2024) [105] and the STITCH database (http://stitch.embl.de, accessed on 2 January 2024) [106] were used to search for the matching targets of the main active components (Table S2). 

Furthermore, their biological targets (452 in total) were predicted using the Binding DB server (https://www.bindingdb.org/rwd/bind/index.jsp, accessed on 10 January 2024) [107], Swiss Target Prediction webtool (http://www.swisstargetprediction.ch/, accessed on 2 January 2024) [105], and STITCH database (http://stitch.embl.de/, accessed on 10 January 2024) [106] (Table S3). To standardize the data, the UniProt database (https://www.uniprot.org/, accessed on 18 January 2024) [108] was utilized to obtain the UniProt IDs of the corresponding genes (Table S3).

#### 4.8.2. Gathering of BC Target Genes and Prediction of Active Ingredients for BC Treatment

Using the DisGeNET database (https://www.disgenet.org/, accessed on 20 January 2024) [109], a search was conducted using the terms BC, tumor, and malignant carcinoma. This search identified cancer-related genes, as shown in Table S4. Using FunRich 3.1.3 software’s Venn diagram intersection [110], the obtained data of cancer-linked genes (5831 genes annotated with uniport) were matched with previously predicted target diseases of SP active ingredients (452 targets). Next the common targets shared by the putative targets and cancer-associated genes were identified by an intersection analysis, and a Venn diagram was generated.

#### 4.8.3. Constructing Protein–Protein Interaction Network

The STRING database (https://cn.string-db.org/, accessed on 22 January 2024) was used to create a protein–protein interaction (PPI) network. Cytoscape 3.9.0 software was used to perform further analyses and embellishments. Firstly, we input potential therapeutic targets for BC into the online database STRING, setting parameters and constraints (limit the species as Homo sapiens, and filtered through the combined score ≥ 0.95 as the threshold). Download the TSV format of the PPI network and import the obtained results into Cytoscape 3.9.0 software to create network diagram for visualization. Through the CytoHubba plug-in, calculate each node of the network and screen core target genes.

#### 4.8.4. Evaluation of the Potential Targets’ Biological Functions

Gene ontology (GO) enrichment analysis and Kyoto Encyclopedia of Genes and Genomes (KEGG) pathway enrichment analysis were conducted to examine the functional biology of potential targets thoroughly and extensively. KEGG pathway analysis results were assessed on the ShinyGO 0.77 (http://bioinformatics.sdstate.edu/go/, accessed on 1 February 2024) [45,111]. The GO enrichment analysis was conducted on Fun Rich 3.1.3 software to extract the key GO terms. GO terms with *p*-values less than 0.05 and enrichment scores higher than 5 were regarded as significant and were examined further. The GO analysis of SP metabolites against BC considered three factors: biological process (BP), molecular function (MF), and cellular component (CC). An adjusted *p*-value threshold of 0.05 was used for pathway discovery. Through the online tools bioinformatics platform http://www.bioinformatics.com.cn/, accessed on 2 February 2024), a visualization analysis was carried out to create a bubble chart and histogram.

#### 4.8.5. The Component–Target Pathway Network

Cytoscape 3.9.0 software was used to construct and visualize a component–target pathway network diagram, while the CytoHubba plug-in was used to rank the top 5 compounds used for constructing the component–target pathway.

#### 4.8.6. Molecular Docking

Molecular docking analysis was conducted using the main genes retrieved from the protein–protein interaction network and the substances gathered in the Spirulina ingredients target network for the purpose of investigating the binding affinity of SP metabolites against target enzymes (EGFR, PI3K, and MAPK(ERK)). A molecular docking simulation was performed using the Molecular Operating Environment (MOE), 2019.0102 software, 2023 (Chemical Computing Group ULC, 910-1010 Sherbrooke St. W., Montreal, QC H3A 2R7, Canada). By adding hydrogen, minimizing energy use, and computing partial charges, a database of active substances was created. This prepared database was ultimately stored with the mdb extension [45,112]. The target enzymes, EGFR, PI3K, and MAPK (ERK) were obtained using PDB IDs 4wkq, 5xgj, and 2zoq, respectively, from the Protein Data Bank (www.rcsb.org). Using the MOE’s automated rapid preparation order, they were readied and verified. With Amber10 Forcefield, the docking simulation was put into practice. Through the use of scoring functions and posture visualization, ligand–protein complex interactions may be evaluated [113]. For co-crystallized ligand protein isozymes (EGFR: 1.5863, PI3K: 0.2683, MAPK(ERK): 1.2392), the Root Mean Square Deviation (RMSD) values were used to evaluate the validity of docking experiments.

### 4.9. Western Blot Analysis 

MCF-7 cells, meticulously grown in six-well plates with a density of around 1 × 10^6^ cells per well, were administered two distinct dosages of SP within 24 h. Following a precise incubation period, the cells were rinsed with cold PBS. After being obtained, the cells were moved into sterile microcentrifuge tubes. Protein extraction was carried out in accordance with the manufacturer’s instructions using the Ready PrepTM protein extraction kit from Bio-Rad Inc. (Catalog #163-2086). The tubes were thereafter subjected to centrifugation with a force of 15,000× *g* for a period of 15 min at a temperature of 4 °C. The protein content in the supernatant was quantified using the Bradford assay (Bio Basic Inc., Markham, ON, Canada, Cat. No. SK304). The experiment involved loading and separating 30 mg of total protein lysate using a 15% SDS-polyacrylamide gel electrophoresis (PAGE) technique. This was achieved by using the TGX Stain-Free™ FastCast™ Acrylamide Kit (SDS-PAGE) offered by Bio-Rad Laboratories Inc. (Catalog Number: 161-0181). The proteins were subsequently deposited onto polyvinylidene difluoride (PVDF) membranes using Bio-Rad equipment. To obstruct the membranes, a solution of 3% bovine serum albumin (BSA) in TBS-Tween (comprising 20 mM Tris, pH 7.5, 150 mM NaCl, and 0.1% Tween 20) was administered and left to incubate for a duration of one h. The primary antibodies targeting Akt, pAkt, EGFR, pEGFR, ERK1/2, and p-ERK1/2 were acquired from Santa Cruz Biotechnology, Inc. (www.scbt.com). The membranes were kept at a temperature of 4 °C overnight and exposed to primary antibodies that specifically targeted Akt (A11, sc377457), pAkt (Thr 308, sc-135650), EGFR (3H2094, sc-71033), pEGFR (15A2, sc-81488), ERK1/2 (12A4, SC-81457), and p-ERK1/2 (cat# 12D4, sc-81492) at a dilution ratio of 1:1000. Afterwards, the blots were exposed to horseradish peroxidase (HRP)-conjugated secondary antibodies (goat anti-rabbit IgG-HRP-1 mg from Goat Mab-Novus Biologicals) in a solution containing 5% milk in TBS-T for 1 h at room temperature. The chemiluminescent signals were identified utilizing an enhanced chemiluminescence detection kit (Clarity™ Western ECL substrate Bio-Rad cat#170-5060), and the pictures were recorded using a CCD camera-based imager. Image analysis software was employed to measure the intensity of the bands corresponding to the target proteins, which were then adjusted based on the β-actin control sample (a protein used as a reference) using the ChemiDoc MP imager.

### 4.10. Statistical Analysis

Statistical software SPSS version 20 (SPSS Inc., Chicago, IL, USA) and the graph plotting software GraphPad Prism 5 (GraphPad Software, San Diego, CA, USA) were utilized for statistical analysis and graph generation. Values are presented as the mean ± standard error of the mean (SEM) of six rats per group in Chicago, IL, USA. An analysis of variance (ANOVA), followed by a post hoc Dunnett’s test, was employed to assess and compare group variations. Any *p*-values below 0.05 (*p* < 0.05) were deemed statistically significant.

## 5. Conclusions

The current study unequivocally establishes that SP acts as a chemopreventive agent against BC, attributing its efficacy to limit cell proliferation, induce apoptosis, antiestrogenic effects, and antioxidant properties. The validation of these effects was conducted in an MCF-7 cell line, representing human breast adenocarcinomas expressing the estrogen receptor. Through comprehensive phytoconstituent profiling, network-based pharmacology, and molecular docking analyses, key therapeutic component and their targets for BC were accurately identified. Central to this investigation are key compounds including 7-hydroxycoumarin derivatives of cinnamic acid, hinokinin, valeric acid, and α-linolenic acid. Three important proteins—EGFR, PI3K-AKT, and ERK1/2 MAPK—are the specific targets of these substances. The findings suggest that SP constituents induce apoptosis and impede cell proliferation by inhibiting the EGFR/Akt and ERK1/2 MAPK signaling pathways. Moving forward, the development of small-molecule inhibitors targeting the EGFR/Akt and ERK1/2 MAPK pathways for BC prevention and therapy requires a rigorous validation of active components and their corresponding targets.

## Figures and Tables

**Table 1:** Pathological traits’ occurrence in normal and DMBA-treated rats treated with SP.

Mammary Gland Pathology	Control	SP	DMBA	SP + DMBA
Epithelial dysplasia of ducts	0	0	25.00 ± 0.26% *	0
Fibroadenoma	0	0	12.00 ± 0.31% *	4.0 ± 0.50% * #
Atypical lobular hyperplasia	0	0	55.00 ± 0.26% *	3.50 ± 0.76% * #
Ductal carcinoma in situ	0	0	8.00 ± 0.26% *	0

Data are represented as the mean ± S.E.M for six rats in each group. * *p* < 0.05 vs. control group. # *p* < 0.05 vs. DMBA group.

**Table 2:** SP’s effect on mammary oxidative stress indicators in control and DMBA-treated rats.

Parameters	Control	SP	DMBA	SP + DMBA
**MDA**	0.25 ± 0.01	0.26 ± 0.02	0.31 ± 0.02 *	0.25 ± 0.03 #
**P. Carbonyl**	0.65± 0.07	0.75 ± 0.04	2.11 ± 0.09 *	0.67 ± 0.053 #
**TAC**	46.82 ± 0.53	47.79 ± 1.09	38.57 ± 0.23 *	46.12± 0.77 #
**CAT**	17.48 ±0.18	18.61 ±0.51	15.67 ±0.21 *	17.55± 0.51 #
**SOD**	10.35 ± 0.16	10.18 ± 0.19	3.30 ± 0.43 *	7.90 ± 0.76 #

Values are expressed as mean ± SEM for six animals in each group. Units of MDA, P. carbonyl, and TAC are nmol/mg protein; units of SOD and CAT enzymes are units/mg protein. * *p* < 0.05 vs. control group. # *p* < 0.05 vs. DMBA group.

**Table 3:** Metabolites characterized in Spirulina.

#	RT (min)	Experimental *m*/*z* ^a^ [M-H]^−^	Experimental *m*/*z* ^a^ [M+H]^+^	Ionization Mode	Molecular Formula	Score	Error (mDa)	Main Fragments	DBE	Proposed Compound	Peak Area	%
1	2.17	116.0725	118.0874	N/P	C_5_H_11_NO_2_	95.75	−0.76	99.0115, 87.0750, 73.0812, 59.0152, 55.0185	1	Valine	1.07 × 10^6^	1.59
2	2.41	130.0875	132.1031	N/P	C_6_H_13_NO_2_	87.52	−0.09	112.9869	1	Leucine/Isoleucine I	1.70 × 10^5^	0.25
3	2.64	103.0410		N	C_4_H_8_O_3_	81.38	−0.97	59.0146	1	Hydroxybutanoic acid I	2.36 × 10^5^	0.35
4	2.87	130.0882	132.1030	N/P	C_6_H_13_NO_2_	94.91	−0.91	112.9870	1	Leucine/Isoleucine II	5.86 × 10^5^	0.88
5	2.99	103.0412		N	C_4_H_8_O_3_	79.77	−1.10	59.0146	1	Hydroxybutanoic acid II	1.53 × 10^5^	0.23
6	3.23	103.0412		N	C_4_H_8_O_3_	81.48	−0.93	59.0154	1	Hydroxybutanoic acid III	8.83 × 10^4^	0.13
7	3.46	164.0725	166.0873	N/P	C_9_H_11_NO_2_	79.58	−0.57	147.0459, 103.0565	5	Phenylalanine	6.59 × 10^5^	0.99
8	4.87	203.0840	205.0980	N/P	C_11_H_12_N_2_O_2_	75.57	−1.13	159.0920, 142.0648, 116.0511	7	Tryptophan	4.79 × 10^4^	0.07
9	5.34	117.0563		N	C_5_H_10_O_3_	75.68	0.06	59.0121	1	Hydroxyvaleric acid	3.35 × 10^5^	0.50
10	7.26	219.0535		N	C_9_H_8_N_4_O_3_	79.04	−1.22	176.0481, 163.0311, 148.0512,	8	6-Propionyllumazine	1.87 × 10^5^	0.28
11	7.46	172.0990	174.1121	N/P	C_8_H_15_NO_3_	81.90	−0.06	130.0864, 128.1065, 102.0569	2	Swainsonine	2.48 × 10^5^	0.37
12	8.17	172.0983	174.1118	N/P	C_8_H_15_NO_3_	86.61	−0.43	130.0876	2	Acetyl leucine/Isoleucine I	1.33 × 10^5^	0.20
13	8.52	172.0992	174.1125	N/P	C_8_H_15_NO_3_	90.64	−1.24	130.0879	2	Acetyl leucine/Isoleucine II	1.55 × 10^5^	0.23
14	8.99	353.1032	355.1114	N/P	C_20_H_18_O_6_	99.13	−0.08	334.6895, 187.0386, 165.0562	12	Hinokinin	4.33 × 10^5^	0.65
15	9.11	165.0579	167.0710	N/P	C_9_H_10_O_3_	93.32	−1.14	121.0661, 93.0351	5	*p*-Dihydrocoumaric acid	8.67 × 10^6^	12.96
16	10.5	101.0616	103.0762	N/P	C_5_H_10_O_2_	94.98	−0.81	59.0121	1	Valeric/Isovaleric acid I	2.84 × 10^6^	4.25
17	11	101.0616		N	C_5_H_10_O_2_	81.92	−0.81	59.0121	1	Valeric/Isovaleric acid II	6.11 × 10^5^	0.91
18	11.69	200.1305		N	C_10_H_19_NO_3_	79.00	−1.29	130.09	2	N-butyryl-Leucine/Isoleiucine I	1.76 × 10^5^	0.26
19	12.04	200.1301		N	C_10_H_19_NO_3_	95.68	−0.93	130.09	2	N-butyryl-Leucine/Isoleiucine II	2.07 × 10^5^	0.31
20	12.28	200.1301		N	C_10_H_19_NO_3_	82.58	−0.98	116.0724, 102.0569	2	N-valeryl-valine	1.01 × 10^5^	0.15
21	13.6	115.0773	117.0919	N/P	C_6_H_12_O_2_	94.85	−0.88	59.02	1	Butyl acetate	3.34 × 10^6^	5.00
22	13.7	197.0805		N	C_10_H_14_O_4_	81.43	1.47	135.0103, 115.0770, 97.0737	4	Germicidin M	7.39 × 10^5^	1.10
23	14.3	149.0618	151.0762	N/P	C_9_H_10_O_2_	94.48	−0.96	105.0711, 77.0012	5	Dihydrocinammic acid	1.30 × 10^7^	19.40
24	16.51	262.1456		N	C_15_H_21_NO_3_	97.38	−0.74	164.00	6	N-caproylphenylalanine	3.44 × 10^5^	0.51
25	22.39	366.2657		N	C_21_H_37_NO_4_	86.55	−1.13	88.0416	4	N-Linoleoyl serine I	1.50 × 10^5^	0.22
26	22.62	366.2663		N	C_21_H_37_NO_4_	76.36	−1.02	88.0415	4	N-Linoleoyl serine II	4.45 × 10^4^	0.07
27	22.86	293.2139		N	C_18_H_30_O_3_	89.8	−1.73	275.2024, 231.2131	4	Hydroxylinolenic acid I	2.24 × 10^5^	0.33
28	22.97	369.2087		N	C_23_H_30_O_4_	92.42	−1.53	352.8626, 227.0032, 198.7446	9	Peyssonoic acid B	1.19 × 10^5^	0.18
29	23.09	366.2663		N	C_21_H_37_NO_4_	85.53	−1.49	88.0416	4	N-Linoleoyl serine III	7.66 × 10^4^	0.11
30	23.33	269.2139		N	C_16_H_30_O_3_	85.75	−1.73	251.2019, 209.0007	2	Oxopalmitic acid	1.70 × 10^5^	0.25
31	23.45	293.2137		N	C_18_H_30_O_3_	89.88	−1.57	275.2046,	4	Hydroxylinolenic acid II	1.91 × 10^5^	0.28
32	23.78	295.2291		N	C_18_H_32_O_3_	93.98	−1.3	277.2177, 217.0039	3	Hydroxylinoleic acid I	2.11 × 10^5^	0.32
33	23.92	293.2134	295.2259	N/P	C_18_H_30_O_3_	87.97	−1.47	275.2030, 231.2128	4	Hydroxylinolenic acid III	3.12 × 10^5^	0.47
34	24.15	293.2137	295.2262	N/P	C_18_H_30_O_3_	77.56	−1.56	275.2027, 231.2066	4	Hydroxylinolenic acid IV	4.08 × 10^5^	0.61
35	24.62	293.2134		N	C_18_H_30_O_3_	80.96	−1.21	275.2018, 231.2120	4	Hydroxylinolenic acid V	5.76 × 10^5^	0.86
36	24.62	295.2279		N	C_18_H_32_O_3_	92.6	−0.21	277.2214, 217.0071	3	Hydroxylinoleic acid II	6.76 × 10^4^	0.10
37	24.98	295.2291	297.2435	N/P	C_18_H_32_O_3_	93.52	−1.15	277.2170, 217.0068	3	Hydroxylinoleic acid III	5.24 × 10^5^	0.78
38	25.09	293.2136	295.2255	N/P	C_18_H_30_O_3_	79.27	−1.42	275.2027, 231.2120	4	Hydroxylinolenic acid VI	2.13 × 10^5^	0.32
39	25.33	295.2289		N	C_18_H_32_O_3_	94.84	−1.15	277.2169, 217.0011	3	Hydroxylinoleic acid IV	8.34 × 10^5^	1.25
40	25.80	295.2291	297.2435	N/P	C_18_H_32_O_3_	80.66	−1.24	277.2182, 217.0044	3	Hydroxylinoleic acid V	6.18 × 10^5^	0.92
41	25.92	355.2295		N	C_23_H_32_O_3_	90.61	−1.79	193.2759, 179.1080, 163.1136	8	Dihyroetretinate I	5.03 × 10^5^	0.75
42	26.15	293.2137	295.2282	N/P	C_18_H_30_O_3_	78.75	−1.36	275.1995, 231.2088	4	Hydroxylinolenic acid VII	4.45 × 10^6^	6.66
43	26.15	555.2865		N	C_25_H_48_O_11_S	90.82	−1.88	499.0104, 436.8754, 418.8524, 401.0186, 225.0091	2	1-*O*-Palmitoyl−3-*O*-(6-sulfo−6-deoxy-alpha-D-glucopyranosyl)-L-glycerol	1.05 × 10^6^	1.58
44	26.27	297.2448	299.2594	N/P	C_18_H_34_O_3_	93.4	−1.23	279.2339, 217.0036	2	Hydroxyoleic acid I	1.46 × 10^5^	0.22
45	26.62	297.2448	299.2583	N/P	C_18_H_34_O_3_	93.95	−1.3	279.2358, 217.0038	2	Hydroxyoleic acid II	3.44 × 10^5^	0.51
46	26.97	293.2130	295.2279	N/P	C_18_H_30_O_3_	83.16	−0.84	275.1982, 230.9760	4	Hydroxylinolenic acid VIII	3.11 × 10^5^	0.47
47	27.09	297.2450	299.2594	N/P	C_18_H_34_O_3_	93.38	−1.31	279.2355, 217.0031	2	Hydroxyoleic acid III	5.23 × 10^5^	0.78
48	28.03	295.2291	297.2442	N/P	C_18_H_32_O_3_	79.69	−1.3	277.2203, 217.0050	3	Hydroxylinoleic acid VI	1.52 × 10^5^	0.23
49	28.38	353.2138		N	C_23_H_30_O_3_	91.38	−1.6	177.0907, 163.1124	9	Etretinate	7.47 × 10^5^	1.12
50	28.85	297.2444	299.2590	N/P	C_18_H_34_O_3_	82.02	−0.82	217.0027	2	Hydroxyoleic acid IV	1.29 × 10^5^	0.19
51	28.97	397.2401	399.2443	N/P	C_25_H_34_O_4_	95.02	0.43	337.2188	9	(7E,12E/Z,20Z,18S)-Variabilin	2.04 × 10^5^	0.30
52	30.50	355.2292	357.2442	N/P	C_23_H_32_O_3_	84.92	−1.87	179.1108	8	Dihyroetretinate II	1.26 × 10^5^	0.19
53	30.74	277.2186		N	C_18_H_30_O_2_	79.66	−1.22	N.D.	4	Linolenic acid I	4.39 × 10^4^	0.07
54	30.97	277.2192	279.2332	N/P	C_18_H_30_O_2_	88.5	−1.79	259.2083, 233.2274	4	Linolenic acid II	3.63 × 10^6^	5.43
55	31.32	277.2188	279.2328	N/P	C_18_H_30_O_2_	92.13	−1.44	259.2055, 233.2272	4	Linolenic acid III	1.26 × 10^6^	1.89
56	31.44	339.2352		N	C_23_H_32_O_2_	86.38	−2.18	163.1135	8	2,2′-Bis(4-methyl6-tert-butylphenol) methane	6.17 × 10^6^	9.23
57	31.68	453.2282		N	C_27_H_34_O_6_	95.02	0.43	N.D.	11	Chromequinolide	3.29 × 10^5^	0.49
58	31.79	277.2186		N	C_18_H_30_O_2_	93.55	−1.99	N.D.	4	Linolenic acid IV	9.82 × 10^5^	1.47
59	31.9	253.2186		N	C_16_H_30_O_2_	93.88	−1.26	209.1569	2	Palmitoleic acid I	1.38 × 10^6^	2.06
60	32.3	253.2185		N	C_16_H_30_O_2_	81.08	1.22	190.167	2	Palmitoleic acid II	9.43 × 10^5^	1.41
61	32.62	279.2344		N	C_18_H_32_O_2_	92.07	−1.42	261.2208, 200.8727	3	Linoleic acid I	1.49 × 10^6^	2.23
62	33.20	279.2343		N	C_18_H_32_O_2_	93.63	−1.32	261.2161	3	Linoleic acid II	1.62 × 10^6^	2.41
63	34.03	255.2336		N	C_16_H_32_O_2_	85.46	−0.58	236.9892	1	Palmitic acid I	6.40 × 10^4^	0.10
64	34.50	255.2336		N	C_16_H_32_O_2_	97.14	−0.43	226.9553	1	Palmitic acid II	2.58 × 10^5^	0.39
65	34.61	281.2495		N	C_18_H_34_O_2_	94.8	−0.99	262.3939	2	Oleic acid I	2.84 × 10^5^	0.42
66	34.97	281.2490		N	C_18_H_34_O_2_	98.15	−0.43	262.6257	2	Oleic acid II	3.23 × 10^5^	0.48
67	37.44	283.2652		N	C_18_H_36_O_2_	83	−0.91	262.6257	1	Stearic acid	1.70 × 10^5^	0.25

N.D., undetected. The letter codes I, II, etc., indicate different isomers. DBE: double-bond equivalence, N: negative ionization mode, P: positive ionization mode. Peak area: lowest value; 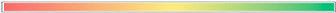 Highest value.

**Table 4:** Docking simulation results of top two active ingredients against target enzymes EGFR, PI3K, and MAPK(ERK).

Active Metabolites PubChem Id (CID)	Target Enzymes	Binding Score kcal/mol	Key Amino Acid Residues	Type of Binding	Two-Dimensional Representation
442879	EGFR	−6.2373	Lys745 CSO797	Hydrophobic H-Bonding	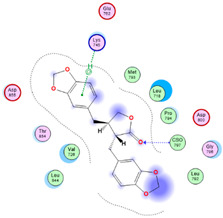
5312775	EGFR	−6.1899	Lys745 Glu762 Asp855	H-Bonding H-Bonding H-Bonding	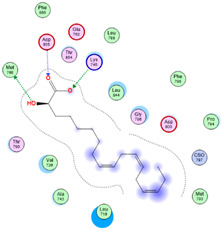
51683	PI3K	−5.1383	Asp810 Asp933 Lys802	H-Bonding, Ionic H-Bonding H-Bonding	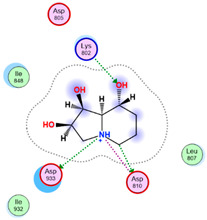
129846263	PI3K	−4.8623	Lys802 Tyr836	H-Bonding, Ionic H-Bonding	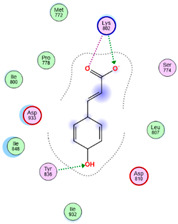
5312775	ERK	−4.9854	Ser170 Asn171 Lys168 Ala52	H-Bonding H-Bonding H-Bonding H-Bonding	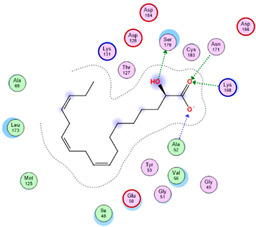
46178008	ERK	−4.8738	Lys168 Asp184 Glu50	2 H-Bonding H-Bonding Hydrophobic	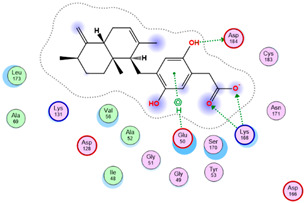

## Data Availability

The data presented in this study are available on request from the corresponding authors.

## Supplementary Materials

The following supporting information can be downloaded at: https://www.mdpi.com/article/10.3390/md22070328/s1; Tables S1–S10 for network pharmacology assays.
