# Supplementary material for: Combining In Vitro, In Vivo, and Network Pharmacology Assays to Identify Targets and Molecular Mechanisms of Spirulina-Derived Biomolecules against Breast Cancer
Source: Mar Drugs. 2024 Jul 22;22(7):328. doi: 10.3390/md22070328 (PMC11278317; doi:10.3390/md22070328)

Supplementary Information

Combining in vitro, in vivo, and network pharmacology assays to identify targets and molecular mechanisms of spirulina-derived biomolecules against breast cancer

Soha Osama Hassanin[1], Amany Mohammed Mohmmed Hegab[2], Reham Hassan Mekky[3], Mohamed Adel Said[4], Mona Gaafar Ahmed Hussein[5], Alaaeldin Ahmed Hamza [2,6*], Amr Amin[7†]

1 Biochemistry Department, Modern University for Technology and information, Cairo 11585, Egypt

2 Egyptian drug authority (EDA), formerly National Organization of Drug Control and Research. Developmental Pharmacology and acute toxicity department,

2 Biology department, Egyptian drug authority (EDA), formerly National Organization of Drug Control and Research

(NODCAR)., Giza,12611 Egypt.

3 Department of Pharmacognosy, Faculty of Pharmacy, Egyptian Russian University, Badr City, Cairo-Suez Road, 11829, Cairo, Egypt,

4 Department of Pharmaceutical Chemistry, Faculty of Pharmacy, Egyptian Russian University, Badr City, Cairo, 11829, Egypt

5 Department of Pharmacology and Toxicology department, Faculty of pharmacy, Modern university for Technology and Information, Cairo, Egypt.

Orchid ID: 0000-0002-6396-7126

6 Medical Research Council, Academy of Scientific Research and Technology, Cairo 11334, Egypt

7 Basic Medical Sciences, College of Medicine, University of Sharjah, Sharjah 27272. UAE.

†Correspondence **a.amin@sharjah.ac.ae**

*Correspondence **alaa17mm@gmail.com**

Supplementary Tables

Table S1-A : Metabolites characterized in SP

Peak no.	RT (min)	Experimental *m/z*[a] [M-H]⁻	Experimental *m/z*[a] [M+H]⁺	Theoretical mass (M)	Ionization Mode	Molecular formula	Score	Error (ppm)	Error (mDa)	Main fragments	DBE	Proposed compound	Class	Subclass	Reference	Peak Area	%
1	2.17	116.0725	118.0874	117.0797	N/P	$C_5H_{11}NO_2$	95.8	-0.6	-0.8	99.0115, 87.0750, 73.0812, 59.0152, 55.0185	1	Valine	Nitrogenous compounds	Amino acid	Terezinha Schneider et al 2023	1.07E+06	1.59
2	2.41	130.0875	132.1031	131.0949	N/P	$C_6H_{13}NO_2$	87.5	-0.7	-0.1	112.9869	1	Leucine/Isoleucine I	Nitrogenous compounds	Amino acid	Terezinha Schneider et al 2023	1.70E+05	0.25
3	2.64	103.0410		104.4830	N	$C_4H_8O_3$	81.4	-9.3	-1.0	59.0146	1	Hydroxybutanoic acid I	Fatty acids	Fatty acid	Reaxys DB	2.36E+05	0.35
4	2.87	130.0882	132.1030	131.0949	N/P	$C_6H_{13}NO_2$	94.9	-6.9	-0.9	112.9870	1	Leucine/Isoleucine II	Nitrogenous compounds	Amino acid	Terezinha Schneider et al 2023	5.86E+05	0.88
5	2.99	103.0412		104.4830	N	$C_4H_8O_3$	79.8	###	-1.1	59.0146	1	Hydroxybutanoic acid II	Fatty acids	Fatty acid	Reaxys DB	1.53E+05	0.23

6	3.23	103.0412		104.4830	N	$C_4H_8O_3$	81.5	-8.9	-0.9	59.0154	1	Hydroxybutanoic acid III	Fatty acids	Fatty acid	Reaxys DB	8.83E+04	0.13
7	3.46	164.0725	166.0873	165.0790	N/P	$C_9H_{11}NO_2$	79.6	-3.7	-0.6	147.0459, 103.0565	5	Phenylalanine	Nitrogenous compounds	Amino acid	Terezinha Schneider et al 2023	6.59E+05	0.99
8	4.87	203.0840	205.0980	204.0906	N/P	$C_{11}H_{12}N_2O_2$	75.6	-5.5	-1.1	159.0920, 142.0648, 116.0511	7	Tryptophan	Nitrogenous compounds	Amino acid	Terezinha Schneider et al 2023	4.79E+04	0.07
9	5.34	117.0563		118.0630	N	$C_5H_{10}O_3$	75.7	0.5	0.1	59.0121	1	Hydroxyvaleric acid	Fatty acids	Fatty acid	Sharma et al 2010	3.35E+05	0.50
10	7.26	219.0535		220.0609	N	$C_9H_8N_4O_3$	79.0	-5.6	-1.2	176.0481, 163.0311, 148.0512,	8	6-Propionyllumazine	Nitrogenous compounds	Alkaloid	Inoue et al 1990	1.87E+05	0.28
11	7.46	172.0990	174.1121	173.1063	N/P	$C_8H_{15}NO_3$	81.9	-6.1	-0.1	130.0864, 128.1065, 102.0569	2	Swainsonine	Nitrogenous compounds	Alkaloid	Davis et al 1984	2.48E+05	0.37
12	8.17	172.0983	174.1118	173.1063	N/P	$C_8H_{15}NO_3$	86.6	-2.5	-0.4	130.0876	2	Acetyl leucine/Isoleucine I	Nitrogenous compounds	Amino acid	Tawaraya et al 2014	1.33E+05	0.20
13	8.52	172.0992	174.1125	173.1063	N/P	$C_8H_{15}NO_3$	90.6	-7.2	-1.2	130.0879	2	Acetyl leucine/Isoleucine II	Nitrogenous compounds	Amino acid	Tawaraya et al 2014	1.55E+05	0.23
14	8.99	353.1032	355.1114	354.1104	N/P	$C_{20}H_{18}O_6$	99.1	-0.2	-0.1	334.6895, 187.0386, 165.0562	12	Hinokinin	Phenolic derivatives	Lignan	Cheng et al 2003	4.33E+05	0.65
15	9.11	165.0579	167.0710	166.0641	N/P	$C_9H_{10}O_3$	93.3	-6.8	-1.1	121.0661, 93.0351	5	*p*-Dihydrocoumaric acid	Phenolic derivatives	Dihydrocinammic acid	Schmitt & Terezinha Schneider 2001	8.67E+06	12.96
16	10.5	101.0616	103.0762	102.0689	N/P	$C_5H_{10}O_2$	95.0	-7.9	-0.8	59.0121	1	Valeric /isovaleric acid I	Fatty acids	Fatty acid	Reaxys DB	2.84E+06	4.25
17	11	101.0616		102.0689	N	$C_5H_{10}O_2$	81.9	-8.0	-0.8	59.0121	1	Valeric /isovaleric acid II	Fatty acids	Fatty acid	Reaxys DB	6.11E+05	0.91
18	11.69	200.1305		201.1375	N	$C_{10}H_{19}NO_3$	79.0	-6.4	-1.3	130.09	2	N-butyryl-Leucine/Isoleucine I	Nitrogenous compounds	Fatty acids Amino acids Conjugates	Reaxys DB	1.76E+05	0.26
19	12.04	200.1301		201.1375	N	$C_{10}H_{19}NO_3$	95.7	-4.6	-0.9	130.09	2	N-butyryl-Leucine/Isoleucine II	Nitrogenous compounds	Fatty acids Amino acids Conjugates	Reaxys DB	2.07E+05	0.31
20	12.28	200.1301		201.1375	N	$C_{10}H_{19}NO_3$	82.6	-4.9	-1.0	116.0724, 102.0569	2	N-valeryl-valine	Nitrogenous compounds	Fatty acids Amino acids Conjugates	Reaxys DB	1.01E+05	0.15
21	13.6	115.0773	117.0919	116.0847	N/P	$C_6H_{12}O_2$	94.9	-7.9	-0.9	59.02	1	Butyl acetate	Fatty acids	Fatty acid	Reaxys DB	3.34E+06	5.00
22	13.7	197.0805		198.0877	N	$C_{10}H_{14}O_4$	81.4	7.4	1.5	135.0103, 115.0770, 97.0737	4	Germicidin M	Phenolic derivatives	Chromanes	Du et al 20189	7.39E+05	1.10
23	14.3	149.0618	151.0762	150.0690	N/P	$C_9H_{10}O_2$	94.5	-6.4	-1.0	105.0711, 77.0012	5	Dihydrocinammic acid	Phenolic derivatives	Dihydrocinammic acid	Reaxys DB / KNDB	1.30E+07	19.40

24	16.51	262.1456		263.1456	N	$C_{15}H_{21}NO_3$	97.4	-2.8	-0.7	164.00	6	N-caproylphenylalanine	Nitrogenous compounds	Fatty acids Amino acids Conjugates	Reaxys DB	3.44E+05	0.51
25	22.39	366.2657		367.2734	N	$C_{21}H_{37}NO_4$	86.6	-3.1	-1.1	88.0416	4	N-Linoleoyl serine I	Nitrogenous compounds	Fatty acids Amino acids Conjugates	Reaxys DB	1.50E+05	0.22
26	22.62	366.2663		367.2734	N	$C_{21}H_{37}NO_4$	76.4	-2.8	-1.0	88.0415	4	N-Linoleoyl serine II	Nitrogenous compounds	Fatty acids Amino acids Conjugates	Reaxys DB	4.45E+04	0.07
27	22.86	293.2139		294.2211	N	$C_{18}H_{30}O_3$	89.8	-5.9	-1.7	275.2024, 231.2131	4	Hydroxylinolenic acid I	Fatty acids	Fatty acid	Reaxys DB	2.24E+05	0.33
28	22.97	369.2087		370.2144	N	$C_{23}H_{30}O_4$	92.4	-4.1	-1.5	352.8626, 227.0032, 198.7446	9	Peyssonoic acid B	Terpenes	Sesquiterpene hydroquinones	Lane et al 2010	1.19E+05	0.18
29	23.09	366.2663		367.2734	N	$C_{21}H_{37}NO_4$	85.5	-4.9	-1.5	88.0416	4	N-Linoleoyl serine III	Nitrogenous compounds	Fatty acids Amino acids Conjugates	Reaxys DB	7.66E+04	0.11
30	23.33	269.2139		270.2212	N	$C_{16}H_{30}O_3$	85.8	-6.4	-1.7	251.2019, 209.0007	2	Oxopalmitic acid	Fatty acids	Fatty acid	Reaxys DB	1.70E+05	0.25
31	23.45	293.2137		294.2211	N	$C_{18}H_{30}O_3$	89.9	-5.3	-1.6	275.2046,	4	Hydroxylinolenic acid II	Fatty acids	Fatty acid	Reaxys DB	1.91E+05	0.28
32	23.78	295.2291		296.2364	N	$C_{18}H_{32}O_3$	94.0	-4.4	-1.3	277.2177, 217.0039	3	Hydroxylinoleic acid I	Fatty acids	Fatty acid	Reaxys DB	2.11E+05	0.32
33	23.92	293.2134	295.2259	294.2211	N/P	$C_{18}H_{30}O_3$	88.0	-5.0	-1.5	275.2030, 231.2128	4	Hydroxylinolenic acid III	Fatty acids	Fatty acid	Reaxys DB	3.12E+05	0.47
34	24.15	293.2137	295.2262	294.2211	N/P	$C_{18}H_{30}O_3$	77.6	-5.3	-1.6	275.2027, 231.2066	4	Hydroxylinolenic acid IV	Fatty acids	Fatty acid	Reaxys DB	4.08E+05	0.61
35	24.62	293.2134		294.2211	N	$C_{18}H_{30}O_3$	81.0	-4.1	-1.2	275.2018, 231.2120	4	Hydroxylinolenic acid V	Fatty acids	Fatty acid	Reaxys DB	5.76E+05	0.86
36	24.62	295.2279		296.2364	N	$C_{18}H_{32}O_3$	92.6	-0.7	-0.2	277.2214, 217.0071	3	Hydroxylinoleic acid II	Fatty acids	Fatty acid	Reaxys DB	6.76E+04	0.10
37	24.98	295.2291	297.2435	296.2364	N/P	$C_{18}H_{32}O_3$	93.5	-3.9	-1.2	277.2170, 217.0068	3	Hydroxylinoleic acid III	Fatty acids	Fatty acid	Reaxys DB	5.24E+05	0.78
38	25.09	293.2136	295.2255	294.2211	N/P	$C_{18}H_{30}O_3$	79.3	-4.8	-1.4	275.2027, 231.2120	4	Hydroxylinolenic acid VI	Fatty acids	Fatty acid	Reaxys DB	2.13E+05	0.32
39	25.33	295.2289		296.2364	N	$C_{18}H_{32}O_3$	94.8	-3.9	-1.2	277.2169, 217.0011	3	Hydroxylinoleic acid IV	Fatty acids	Fatty acid	Reaxys DB	8.34E+05	1.25
40	25.80	295.2291	297.2435	296.2364	N/P	$C_{18}H_{32}O_3$	80.7	-4.2	-1.2	277.2182, 217.0044	3	Hydroxylinoleic acid V	Fatty acids	Fatty acid	Reaxys DB	6.18E+05	0.92

41	25.92	355.2295		356.2369	N	$C_{23}H_{32}O_3$	90.6	-5.0	-1.8	193.2759, 179.1080, 163.1136	8	Dihyroetretinate I	Terpenes	Retinoid	Reaxys DB	5.03E+05	0.75
42	26.15	293.2137	295.2282	294.2211	N/P	$C_{18}H_{30}O_3$	78.8	-4.6	-1.4	275.1995, 231.2088	4	Hydroxylinolenic acid VII	Fatty acids	Fatty acid	Reaxys DB	4.45E+06	6.66
43	26.2	555.2865		556.2936	N	$C_{25}H_{48}O_{11}S$	90.8	-3.4	-1.9	499.0104, 436.8754, 418.8524, 401.0186, 225.0091	2	1-*O*-Palmitoyl-3-*O*-(6-sulfo-6-deoxy-alpha-D-glucopyranosyl)-L-glycerol	Sulfur compounds	Sulfur compounds	Reaxys DB/ Lotus/ Allaoua et al 2016	1.05E+06	1.58
44	26.27	297.2448	299.2594	298.2520	N/P	$C_{18}H_{34}O_3$	93.4	-4.1	-1.2	279.2339, 217.0036	2	Hydroxyoleic acid I	Fatty acids	Fatty acid	Reaxys DB	1.46E+05	0.22
45	26.62	297.2448	299.2583	298.2520	N/P	$C_{18}H_{34}O_3$	94.0	-4.4	-1.3	279.2358, 217.0038	2	Hydroxyoleic acid II	Fatty acids	Fatty acid	Reaxys DB	3.44E+05	0.51
46	26.97	293.2130	295.2279	294.2211	N/P	$C_{18}H_{30}O_3$	83.2	-2.9	-0.8	275.1982, 230.9760	4	Hydroxylinolenic acid VII*I*	Fatty acids	Fatty acid	Reaxys DB	3.11E+05	0.47
47	27.09	297.2450	299.2594	298.2520	N/P	$C_{18}H_{34}O_3$	93.4	-4.4	-1.3	279.2355, 217.0031	2	Hydroxyoleic acid III	Fatty acids	Fatty acid	Reaxys DB	5.23E+05	0.78
48	28.03	295.2291	297.2442	296.2364	N/P	$C_{18}H_{32}O_3$	79.7	-4.4	-1.3	277.2203, 217.0050	3	Hydroxylinoleic acid VI	Fatty acids	Fatty acid	Reaxys DB	1.52E+05	0.23
49	28.38	353.2138		354.2211	N	$C_{23}H_{30}O_3$	91.4	-4.5	-1.6	177.0907, 163.1124	9	Etretinate	Terpenes	Retinoid	Reaxys DB/ KNDB	7.47E+05	1.12
50	28.85	297.2444	299.2590	298.2520	N/P	$C_{18}H_{34}O_3$	82.0	-2.7	-0.8	217.003	2	Hydroxyoleic acid IV	Fatty acids	Fatty acid	Reaxys DB	1.29E+05	0.19
51	28.97	397.2401	399.2443	398.2471	N/P	$C_{25}H_{34}O_4$	95.0	0.9	0.4	337.219	9	(7E,12E/Z,20Z,18S)-Variabilin	Terpenes	Sesterterpene	Balansa et al 2010	2.04E+05	0.30
52	30.50	355.2292	357.2442	356.2369	N/P	$C_{23}H_{32}O_3$	84.9	-5.2	-1.9	179.111	8	Dihyroetretinate II	Terpenes	Retinoid	Reaxys DB	1.26E+05	0.19
53	30.74	277.2186		278.2264	N	$C_{18}H_{30}O_2$	79.7	-4.4	-1.2	N.D.	4	Linolenic acid I	Fatty acids	Fatty acid	Terezinha Schneider et al 2023	4.39E+04	0.07
54	30.97	277.2192	279.2332	278.2264	N/P	$C_{18}H_{30}O_2$	88.5	-6.4	-1.8	259.2083, 233.2274	4	Linolenic acid II	Fatty acids	Fatty acid	Terezinha Schneider et al 2023	3.63E+06	5.43

55	31.32	277.2188	279.2328	278.2264	N/P	$C_{18}H_{30}O_2$	92.1	-5.2	-1.4	259.2055, 233.2272	4	Linolenic acid III	Fatty acids	Fatty acid	Terezinha Schneider et al 2023	1.26E+06	1.89
56	31.44	339.2352		340.2424	N	$C_{23}H_{32}O_2$	86.4	-6.4	-2.2	163.114	8	2,2'-Bis(4-methyl6-tert-butylphenol) methane	Phenolic derivatives	Phenol	Reaxys DB/ Qiao el al 2022	6.17E+06	9.23
57	31.68	453.2282		454.2351	N	$C_{27}H_{34}O_6$	95.0	0.9	0.4	N.D.	11	Chromequinolide	Phenolic derivatives	Quinone	Horie et al 2008	3.29E+05	0.49
58	31.79	277.2186		278.2264	N	$C_{18}H_{30}O_2$	93.6	-4.3	-2.0	N.D.	4	Linolenic acid IV	Fatty acids	Fatty acid	Terezinha Schneider et al 2023	9.82E+05	1.47
59	31.9	253.2186		254.2258	N	$C_{16}H_{30}O_2$	93.9	-5.0	-1.3	209.157	2	Palmitoleic acid I	Fatty acids	Fatty acid	Terezinha Schneider et al 2023	1.38E+06	2.06
60	32.3	253.2185		254.2258	N	$C_{16}H_{30}O_2$	81.1	-4.8	1.2	190.167	2	Palmitoleic acid II	Fatty acids	Fatty acid	Terezinha Schneider et al 2023	9.43E+05	1.41
61	32.62	279.2344		280.2413	N	$C_{18}H_{32}O_2$	92.1	-5.1	-1.4	261.2208, 200.8727	3	Linoleic acid I	Fatty acids	Fatty acid	Terezinha Schneider et al 2023	1.49E+06	2.23
62	33.20	279.2343		280.2413	N	$C_{18}H_{32}O_2$	93.6	-4.7	-1.3	261.216	3	Linoleic acid II	Fatty acids	Fatty acid	Terezinha Schneider et al 2023	1.62E+06	2.41
63	34.03	255.2336		256.2460	N	$C_{16}H_{32}O_2$	85.5	-2.3	-0.6	236.989	1	Palmitic acid I	Fatty acids	Fatty acid	Terezinha Schneider et al 2023	6.40E+04	0.10
64	34.50	255.2336		256.2460	N	$C_{16}H_{32}O_2$	97.1	-1.7	-0.4	226.955	1	Palmitic acid II	Fatty acids	Fatty acid	Terezinha Schneider et al 2023	2.58E+05	0.39
65	34.61	281.2495		282.2559	N	$C_{18}H_{34}O_2$	94.8	-3.5	-1.0	262.394	2	Oleic acid I	Fatty acids	Fatty acid	Terezinha Schneider et al 2023	2.84E+05	0.42
66	34.97	281.2490		282.2559	N	$C_{18}H_{34}O_2$	98.2	-1.5	-0.4	262.626	2	Oleic acid II	Fatty acids	Fatty acid	Terezinha Schneider et al 2023	3.23E+05	0.48
67	37.44	283.2652		284.2724	N	$C_{18}H_{36}O_2$	83.0	-3.2	-0.9	262.626	1	Stearic acid	Fatty acids	Fatty acid	Terezinha Schneider et al 2023	1.70E+05	0.25

[a]Detected ions were $[M-H]^-$ and/or $[M+H]^+$. Isomers are described by letters I, II, etc. N.D., not detected signal, below 5 mAU or masked by a compound with a higher signal.

Peak area: Lowest value Higher value

References

Allaoua, Z.; Benkhaled, M.; Dibi, A.; Long, C.; Aberkane, M.C.; Bouzidi, S.; Kassah-Laouar, A.; Haba, H. Chemical composition, antioxidant and antibacterial properties of *Pteranthus dichotomus* from Algerian Sahara. *Nat Prod Res* **2016**, *30*, 700-704, doi:10.1080/14786419.2015.1038811.

	Davis, D.; Schwarz, P.; Hernandez, T.; Mitchell, M.; Warnock, B.; Elbein, A.D. Isolation and Characterization of Swainsonine from Texas Locoweed (*Astragalus emoryanus*) 1. *Plant Physiology* **1984**, *76*, 972-975, doi:10.1104/pp.76.4.972.
	Du, Y.; Sun, J.; Gong, Q.; Wang, Y.; Fu, P.; Zhu, W. New α-pyridones with quorum-sensing inhibitory activity from diversity-enhanced extracts of a Streptomyces sp. derived from marine algae. *Journal of agricultural and food chemistry* **2018**, *66*, 1807-1812.
	Horie, S.; Tsutsumi, S.; Takada, Y.; Kimura, J. Antibacterial Quinone Metabolites from the Brown Alga,*Sargassum sagamianum*. *Bulletin of the Chemical Society of Japan* **2008**, *81*, 1125-1130, doi:10.1246/bcsj.81.1125.
	Inoue, S.; Okada, K.; Tanino, H.; Kakoi, H.; Horii, N. 6-Propionyllumazines from the marine polychaete, *Odontosyllis undecimdonta*. *Chemistry Letters* **1990**, *19*, 367-368.
	KNApSAcK-Core-System. (http://www.knapsackfamily.com/knapsack_jsp/top.html).
	Lane, A.L.; Mular, L.; Drenkard, E.J.; Shearer, T.L.; Engel, S.; Fredericq, S.; Fairchild, C.R.; Prudhomme, J.; Le Roch, K.; Hay, M.E., et al. Ecological leads for natural product discovery: Novel sesquiterpene hydroquinones from the red macroalga Peyssonnelia sp. *Tetrahedron* **2010**, *66*, 455-461, doi:10.1016/j.tet.2009.11.042.
	Lotus Natural Products database (https://lotus.naturalproducts.net/)
	Qiao, J.; Lu, G.; Wu, G.; Liu, H.; Wang, W.; Zhang, T.; Xie, G.; Qin, M. Influence of different pretreatments and drying methods on the chemical compositions and bioactivities of *Smilacis Glabrae* Rhizoma. *Chinese Medicine* **2022**, *17*, 54, doi:10.1186/s13020-022-00614-7.
	Reaxys. (http://www.reaxys.com).
	Terezinha Schneider, A.; Costa Deprá, M.; Rodrigues Dias, R.; Queiroz Zepka, L.; Jacob-Lopes, E. Chapter 5 - Microalgae as superfood. In Algae Materials, Arunkumar, K., Arun, A., Raja, R., Palaniappan, R., Eds. Academic Press: 2023; https://doi.org/10.1016/B978-0-443-18816-9.00023-Xpp. 93-102.

TableS1-B

Proposed active ingredients of Spirulina, their subclass, smiles, PubChem Id and further comments

Proposed compound (bioactives)	Subclass	Pubchem ID (CID)	COMMENTS	SMILES
N-valeryl-valine	Amino acid conjugated with fatty acid	36689743		CCCCC(=O)NC(C(C)C)C(=O)O
6-Propionyllumazine	Alkaloid	639142		CCC(=O)C1=CN=C2C(=N1)C(=O)NC(=O)N2
Swainsonine	Alkaloid	51683		C1CC(C2C(C(CN2C1)O)O)O
Hinokinin	Lignan	442879		C1C(C(C(=O)O1)CC2=CC3=C(C=C2)OCO3)CC4=CC5=C(C=C4)OCO5
p -Dihydrocoumaric acid	Dihydrocinammic acid	129846263		C1=CC(C=CC1C=CC(=O)O)O
Germicidin M	Chromanes	146684237	No Targets Identified	CC1=C(C=C(OC1=O)C(C)C(C)O)O
Dihydrocinammic acid	Dihydrocinammic acid	107		C1=CC=C(C=C1)CCC(=O)O
Linoleoyl serine	Amino acid conjugated with fatty acid	4190406		CCOC(=O)C1=C(SC2=C1CCC(C2)C)NC(=O)C3=CC=C(C=C3)OC(C)C(=O)OCC
Peyssonoic acid B	Sesquiterpene hydroquinones	46178008		CC1CCC2(C(C1=C)CC=C(C2CC3=CC(=C(C=C3O)CC(=O)O)O)C)C
Dihyroetretinate I	Retinoid	88833376		CCOC(=O)C=C(C)CCC=C(C)C=CC1=C(C(=C(C=C1C)OC)C)C
1-*O* -Palmitoyl-3-*O* -(6-sulfo-6-deoxy-alpha-D-glucopyranosyl)-L-glycerol	Sulfure compounds	101560606		CCCCCCCCCCCCCCCC(=O)OCC(COC1C(C(C(C(O1)CS(=O)(=O)O)O)O)O)O
Etretinate	Retinoid	5282375		CCOC(=O)C=C(C)C=CC=C(C)C=CC1=C(C(=C(C=C1C)OC)C)C
(7E,12E/Z,20Z,18S)-Variabilin	Sesterterpene	54678431		CC1=C(C(=CC(C)CCCC(=CCCC(=CCCC2=COC=C2)C)C)OC1=O)O
2,2'-Bis(4-methyl6-tert-butylphenol) methane	Phenol	66237		CC(C)(C)C1=C(C=CC(=C1)C(C)(C)C2=CC(=C(C=C2)O)C(C)(C)C)O
chromequinolide	Quinone	162870052		CC1=CC(=O)C2=C(C1=O)C=CC(O2)(C)CCC=C(C)CCC=C3CCC(OC3=O)C(C)(C)O
Leucine	Amino acid	6106		CC(C)CC(C(=O)O)N
isoleucine	Amino acid	6306		CCC(C)C(C(=O)O)N
Valeric acid	Fatty acid	7991		CCCCC(=O)O
Isovaleric acid	Fatty acid	10430		CC(C)CC(=O)O
Butyl acetate	Fatty acid	31272		CCCCOC(=O)C
Hydroxybutanoic acid I	Fatty acid	10413		C(CC(=O)O)CO
Hydroxyvaleric acid	Fatty acid	98009		CCCC(C(=O)O)O
Hydroxylinolenic acid II	Fatty acid	5312775		CCC=CCC=CCC=CCCCCCCCC(C(=O)O)O
Palmitoleic acid I	Fatty acid	445638		CCCCCCC=CCCCCCCCC(=O)O
Valine	Amino acid	6287		CC(C)C(C(=O)O)N

Table S2

Spirulina active ingredients filteration by the evaluation of their drug-likeness (QED) and oral bioavailability (Lipinski's rule/Pfizer rule) using ADMET Lab 2.0

Proposed compound (bioactives)	SMILES	QED (drug-likeness)	Lipinski rule (oral bioavailability)	Pfizer rule (oral bioavailability)
N-valeryl-valine	CCCCC(=O)NC(C(=O)O)C(C)C	0.682	Accepted	Accepted
6-Propionyllumazine	CCC(=O)c1cnc2[nH]c(=O)[nH]	0.669	Accepted	Accepted
Swainsonine	OC1CN2CCCC(O)C2C1O	0.419	Accepted	Accepted
Hinokinin	O=C1OCC(Cc2ccc3c(c2)OCO3)	0.787	Accepted	Rejected
p-Dihydrocoumaric acid	O=C(O)C=CC1=CCC(O)C=C1	0.598	Accepted	Accepted
Germicidin M	Cc1c(O)cc(C(C)C(C)O)oc1=O	0.747	Accepted	Accepted
Dihydrocinammic acid	O=C(O)CCc1ccccc1	0.712	Accepted	Accepted
Linoleoyl serine	CCOC(=O)c1c(NC(=O)c2ccc(O	0.579	Accepted	Accepted
Peyssonoic acid B	C=C1C(C)CCC2(C)C1CC=C(C)C	0.523	Accepted	Accepted
Dihyroetretinate I	CCOC(=O)C=C(C)CCC=C(C)C=	0.332	Accepted	Rejected
1-O-Palmitoyl-3-O-(6-sulfo-6-deoxy-	CCCCCCCCCCCCCCCC(=O)OC	0.379	Rejected	Accepted
Etretinate	CCOC(=O)C=C(C)C=CC=C(C)C	0.361	Accepted	Rejected
(7E,12E/Z,20Z,18S)-Variabilin	CC(=CCCc1ccoc1)CCC=C(C)CC	0.385	Accepted	Rejected
2,2'-Bis(4-methyl6-tert-butylphenol) methane	CC(C)(C)c1cc(C(C)(C)c2ccc(O)	0.702	Accepted	Rejected
chromequinolide	CC(=CCCC1(C)C=CC2=C(O1)C	0.347	Accepted	Accepted
Leucine	CC(C)CC(N)C(=O)O	0.584	Accepted	Accepted
isoleucine	CCC(C)C(N)C(=O)O	0.584	Accepted	Accepted
Valeric acid	CCCCC(=O)O	0.582	Accepted	Accepted
Isovaleric acid	CC(C)CC(=O)O	0.567	Accepted	Accepted
Butyl acetate	CCCCOC(C)=O	0.411	Accepted	Accepted
Hydroxybutanoic acid I	O=C(O)CCCO	0.523	Accepted	Accepted
Hydroxyvaleric acid	CCCC(=O)C(O)O	0.499	Accepted	Accepted
Hydroxylinolenic acid II	CCC=CCC=CCC=CCCCCCCC(=C	0.306	Accepted	Rejected
Palmitoleic acid I	CCCCCCC=CCCCCCCCC(=O)O	0.356	Accepted	Rejected
Valine	CC(C)C(N)C(=O)O	0.537	Accepted	Accepted

Table S3

*G*athering the predicted targets of active ingredients from spirulina

Proposed compound (bioactives) PubChem ID (CID)	Predicted TARGETs	Common name of predicted targets	UNI PROT ID of predicted targets
107	11-beta-hydroxysteroid dehydrogenase 1	HSD11B1	P28845
107	Aldose reductase (by homology)	AKR1B1	P15121
107	Aminopeptidase B	RNPEP	Q9H4A4
107	Carboxypeptidase A1	CPA1	P15085
107	Carboxypeptidase B2 isoform A	CPB2	Q96IY4
107	C-terminal-binding protein 2	CTBP2	P56545
107	Cyclooxygenase-1	PTGS1	P23219
107	Egl nine homolog 3	EGLN3	Q9H6Z9
107	Estradiol 17-beta-dehydrogenase 3	HSD17B3	P37058
107	Free fatty acid receptor 1	FFAR1	O14842
107	Glutamate carboxypeptidase II	FOLH1	Q04609
107	G-protein coupled receptor 120	FFAR4	Q5NUL3
107	HMG-CoA reductase (by homology)	HMGCR	P04035
107	Hydroxycarboxylic acid receptor 2	HCAR2	Q8TDS4
107	L-type amino acid transporter 1	SLC7A5	Q01650
107	Mast cell carboxypeptidase A	CPA3	P15088
107	Monocarboxylate transporter 1 (by homology)	SLC16A1	P53985
107	Peptidyl-glycine alpha-amidating monooxygenase	PAM	P19021
107	Prostanoid EP1 receptor	PTGER1	P34995
107	Prostanoid FP receptor	PTGFR	P43088
107	Solute carrier family 22 member 6 (by homology)	SLC22A6	Q4U2R8
107	Voltage-gated calcium channel alpha2/delta subunit 1 (by homology)	CACNA2D1	P54289
6106	Adenosine A3 receptor	ADORA3	P0DMS8
6106	Betaine transporter	SLC6A12	P48065
6106	Excitatory amino acid transporter 2	SLC1A2	P43004
6106	Excitatory amino acid transporter 3	SLC1A1	P43005
6106	GABA receptor rho-1 subunit	GABRR1	P24046

6106	GABA transporter 2 (by homology)	SLC6A13	Q9NSD5
6106	GABA transporter 3	SLC6A11	P48066
6106	GABA-A receptor; alpha-1/beta-2/gamma-2	GABRA1 GABRB2 GABRG2	P14867 P47870 P18507
6106	GABA-B receptor	GABBR2 GABBR1	O75899 Q9UBS5
6106	Gamma-butyrobetaine dioxygenase	BBOX1	O75936
6106	Glutamate receptor ionotropic kainate 1	GRIK1	P39086
6106	Glutamate receptor ionotropic kainate 2	GRIK2	Q13002
6106	Glutamate receptor ionotropic kainate 3	GRIK3	Q13003
6106	Glutamate receptor ionotropic kainate 5	GRIK5	Q16478
6106	Glutamate receptor ionotropic, AMPA 1	GRIA1	P42261
6106	Glutamate receptor ionotropic, AMPA 2	GRIA2	P42262
6106	Glutamate receptor ionotropic, AMPA 4	GRIA4	P48058
6106	Kynurenine 3-monooxygenase	KMO	O15229
6106	Leukotriene A4 hydrolase	LTA4H	P09960
6106	Metabotropic glutamate receptor 1	GRM1	Q13255
6106	Metabotropic glutamate receptor 2	GRM2	Q14416
6106	Metabotropic glutamate receptor 3	GRM3	Q14832
6106	Metabotropic glutamate receptor 4	GRM4	Q14833
6106	Metabotropic glutamate receptor 5	GRM5	P41594
6106	Metabotropic glutamate receptor 6	GRM6	O15303
6106	Metabotropic glutamate receptor 7	GRM7	Q14831
6106	Metabotropic glutamate receptor 8	GRM8	O00222
6106	Ornithine aminotransferase, mitochondrial	OAT	P04181
6106	Plasminogen	PLG	P00747
6106	Tyrosine 3-hydroxylase	TH	P07101
6106	Voltage-gated calcium channel alpha2/delta subunit 1 (by homology)	CACNA2D1	P54289
6287	Adenosine A3 receptor	ADORA3	P0DMS8
6287	GABA A receptor alpha-2/beta-2/gamma-2	GABRA2 GABRB2 GABRG2	P47869 P47870 P18507
6287	GABA A receptor alpha-3/beta-2/gamma-2	GABRA3 GABRB2 GABRG2	P34903 P47870 P18507
6287	GABA receptor rho-1 subunit	GABRR1	P24046
6287	GABA transporter 1	SLC6A1	P30531
6287	GABA transporter 2 (by homology)	SLC6A13	Q9NSD5
6287	GABA transporter 3	SLC6A11	P48066
6287	GABA-B receptor	GABBR2 GABBR1	O75899 Q9UBS5

6287	GABA-B receptor (by homology)	GABBR1	Q9UBS5
6287	Histone deacetylase 3	HDAC3	O15379
6287	Metabotropic glutamate receptor 4	GRM4	Q14833
6287	Metabotropic glutamate receptor 5	GRM5	P41594
6287	Metabotropic glutamate receptor 7	GRM7	Q14831
6287	Metabotropic glutamate receptor 8	GRM8	O00222
6287	Ornithine aminotransferase, mitochondrial	OAT	P04181
6287	Voltage-gated calcium channel alpha2/delta subunit 1 (by homology)	CACNA2D1	P54289
6306	Adenosine A3 receptor	ADORA3	P0DMS8
6306	Excitatory amino acid transporter 2	SLC1A2	P43004
6306	Excitatory amino acid transporter 3	SLC1A1	P43005
6306	GABA receptor rho-1 subunit	GABRR1	P24046
6306	GABA transporter 2 (by homology)	SLC6A13	Q9NSD5
6306	GABA transporter 3	SLC6A11	P48066
6306	GABA-A receptor; alpha-1/beta-2/gamma-2	GABRA1 GABRB2 GABRG2	P14867 P47870 P18507
6306	GABA-B receptor	GABBR2 GABBR1	O75899 Q9UBS5
6306	Gamma-butyrobetaine dioxygenase	BBOX1	O75936
6306	Glutamate receptor ionotropic kainate 1	GRIK1	P39086
6306	Glutamate receptor ionotropic kainate 2	GRIK2	Q13002
6306	Glutamate receptor ionotropic kainate 3	GRIK3	Q13003
6306	Glutamate receptor ionotropic kainate 5	GRIK5	Q16478
6306	Glutamate receptor ionotropic, AMPA 1	GRIA1	P42261
6306	Glutamate receptor ionotropic, AMPA 2	GRIA2	P42262
6306	Glutamate receptor ionotropic, AMPA 4	GRIA4	P48058
6306	Leukotriene A4 hydrolase	LTA4H	P09960
6306	Metabotropic glutamate receptor 1	GRM1	Q13255
6306	Metabotropic glutamate receptor 2	GRM2	Q14416
6306	Metabotropic glutamate receptor 3	GRM3	Q14832
6306	Metabotropic glutamate receptor 4	GRM4	Q14833
6306	Metabotropic glutamate receptor 5	GRM5	P41594
6306	Metabotropic glutamate receptor 6	GRM6	O15303
6306	Metabotropic glutamate receptor 7	GRM7	Q14831
6306	Metabotropic glutamate receptor 8	GRM8	O00222
6306	Ornithine aminotransferase, mitochondrial	OAT	P04181

6306	Plasminogen	PLG	P00747
6306	Voltage-gated calcium channel alpha2/delta subunit 1 (by homology)	CACNA2D1	P54289
7991	11-beta-hydroxysteroid dehydrogenase 1	HSD11B1	P28845
7991	Aldo-keto reductase family 1 member B10	AKR1B10	O60218
7991	Androgen Receptor	AR	P10275
7991	Bile acid receptor FXR	NR1H4	Q96RI1
7991	Corticosteroid binding globulin	SERPINA6	P08185
7991	Cytochrome P450 19A1	CYP19A1	P11511
7991	DNA polymerase beta (by homology)	POLB	P06746
7991	Dual specificity phosphatase Cdc25A	CDC25A	P30304
7991	Egl nine homolog 1	EGLN1	Q9GZT9
7991	Estradiol 17-beta-dehydrogenase 3	HSD17B3	P37058
7991	Fatty acid binding protein adipocyte	FABP4	P15090
7991	Fatty acid binding protein epidermal	FABP5	Q01469
7991	Fatty acid binding protein intestinal	FABP2	P12104
7991	Fatty acid binding protein muscle	FABP3	P05413
7991	Free fatty acid receptor 1	FFAR1	O14842
7991	GABA-B receptor (by homology)	GABBR1	Q9UBS5
7991	Glucose-6-phosphate 1-dehydrogenase	G6PD	P11413
7991	G-protein coupled bile acid receptor 1	GPBAR1	Q8TDU6
7991	Histone deacetylase 3	HDAC3	O15379
7991	Histone lysine demethylase PHF8	PHF8	Q9UPP1
7991	HMG-CoA reductase	HMGCR	P04035
7991	Lysine-specific demethylase 2A	KDM2A	Q9Y2K7
7991	Lysine-specific demethylase 5C	KDM5C	P41229
7991	Peroxisome proliferator-activated receptor alpha	PPARA	Q07869
7991	Peroxisome proliferator-activated receptor delta	PPARD	Q03181
7991	Prostanoid EP2 receptor	PTGER2	P43116
7991	Prostanoid FP receptor	PTGFR	P43088
7991	Protein farnesyltransferase	FNTA FNTB	P49354 P49356
7991	Solute carrier family 22 member 6 (by homology)	SLC22A6	Q4U2R8
7991	Testis-specific androgen-binding protein	SHBG	P04278
7991	UDP-glucuronosyltransferase 2B7	UGT2B7	P16662
7991	Vitamin D receptor	VDR	P11473

10413	Egl nine homolog 1	EGLN1	Q9GZT9
10413	Histone deacetylase 3	HDAC3	O15379
10430	Egl nine homolog 1	EGLN1	Q9GZT9
10430	GABA A receptor alpha-2/beta-2/gamma-2	GABRA2 GABRB2 GABRG2	P47869 P47870 P18507
10430	GABA A receptor alpha-3/beta-2/gamma-2	GABRA3 GABRB2 GABRG2	P34903 P47870 P18507
10430	GABA receptor rho-1 subunit	GABRR1	P24046
10430	GABA transporter 1	SLC6A1	P30531
10430	GABA transporter 2 (by homology)	SLC6A13	Q9NSD5
10430	GABA transporter 3	SLC6A11	P48066
10430	GABA-A receptor; alpha-1/beta-2/gamma-2	GABRA1 GABRB2 GABRG2	P14867 P47870 P18507
10430	GABA-B receptor	GABBR2 GABBR1	O75899 Q9UBS5
10430	Histone deacetylase 3	HDAC3	O15379
10430	Solute carrier family 22 member 6 (by homology)	SLC22A6	Q4U2R8
31272	Acetylcholinesterase	ACHE	P22303
31272	Beta-chymotrypsin	CTRB1	P17538
31272	Carbonic anhydrase I	CA1	P00915
31272	Carbonic anhydrase IX	CA9	Q16790
31272	Cytochrome P450 11B1	CYP11B1	P15538
31272	Cytochrome P450 11B2	CYP11B2	P19099
31272	Cytochrome P450 2A6	CYP2A6	P11509
31272	Dopamine transporter	SLC6A3	Q01959
31272	Histamine H3 receptor	HRH3	Q9Y5N1
31272	Huntingtin	HTT	P42858
31272	Indoleamine 2,3-dioxygenase	IDO1	P14902
31272	Leukocyte elastase	ELANE	P08246
31272	Neuronal acetylcholine receptor; alpha3/beta4	CHRNA3 CHRNB4	P32297 P30926
31272	Nischarin	NISCH	Q9Y2I1
31272	Norepinephrine transporter	SLC6A2	P23975
31272	Plasma kallikrein	KLKB1	P03952
31272	Protein-glutamine gamma-glutamyltransferase	TGM2	P21980
31272	Thymidylate synthase	TYMS	P04818
31272	Urokinase-type plasminogen activator	PLAU	P00749
51683	Alpha-galactosidase A	GLA	P06280
51683	Alpha-L-fucosidase 2	FUCA2	Q9BTY2

51683	Alpha-L-fucosidase I	FUCA1	P04066
51683	Anti-estrogen binding site (AEBS)	EBP	Q15125
51683	Beta-galactosidase	GLB1	P16278
51683	Beta-glucocerebrosidase	GBA	P04062
51683	Beta-glucosidase	GBA2	Q9HCG7
51683	Beta-mannosidase (by homology)	MANBA	O00462
51683	Ceramide glucosyltransferase	UGCG	Q16739
51683	Endo-beta-N-acetylglucosaminidase	ENGASE	Q8NFI3
51683	Glycogen debranching enzyme	AGL	P35573
51683	Heat shock 70 kDa protein 1	HSPA1A	P0DMV8
51683	Lanosterol synthase	LSS	P48449
51683	Liver glycogen phosphorylase	PYGL	P06737
51683	Lysosomal alpha-glucosidase	GAA	P10253
51683	Lysosomal alpha-mannosidase	MAN2B1	O00754
51683	Maltase-glucoamylase	MGAM	O43451
51683	Neutral alpha-glucosidase AB	GANAB	Q14697
51683	Neutral alpha-glucosidase C	GANC	Q8TET4
51683	Ornithine decarboxylase (by homology)	ODC1	P11926
51683	Phospholipase A2 group 1B	PLA2G1B	P04054
51683	Phospholipase A2 group IIA	PLA2G2A	P14555
51683	Polyadenylate-binding protein 1	PABPC1	P11940
51683	Sigma opioid receptor	SIGMAR1	Q99720
51683	Sucrase-isomaltase	SI	P14410
51683	Vesicular acetylcholine transporter	SLC18A3	Q16572
66237	11-beta-hydroxysteroid dehydrogenase 1	HSD11B1	P28845
66237	Alpha-1b adrenergic receptor	ADRA1B	P35368
66237	Androgen Receptor	AR	P10275
66237	Arachidonate 5-lipoxygenase	ALOX5	P09917
66237	Beta-secretase 1	BACE1	P56817
66237	Bile acid receptor FXR	NR1H4	Q96RI1
66237	Butyrylcholinesterase	BCHE	P06276
66237	Carbonic anhydrase II	CA2	P00918
66237	Carbonic anhydrase IX	CA9	Q16790
66237	Cathepsin (V and K)	CTSV	O60911

66237	CDC7/DBF4 (Cell division cycle 7-related protein kinase/Activator of S phase kinase)	DBF4 CDC7	Q9UBU7 O00311
66237	Cholecystokinin B receptor	CCKBR	P32239
66237	C-X-C chemokine receptor type 3	CXCR3	P49682
66237	Cyclin-dependent kinase 4	CDK4	P11802
66237	Cyclooxygenase-1	PTGS1	P23219
66237	Epoxide hydratase	EPHX2	P34913
66237	Estrogen receptor alpha	ESR1	P03372
66237	Estrogen receptor beta	ESR2	Q92731
66237	Estrogen-related receptor gamma	ESRRG	P62508
66237	GABA-A receptor; alpha-1/beta-2/gamma-2	GABRA1 GABRB2 GABRG2	P14867 P47870 P18507
66237	GABA-A receptor; alpha-1/beta-3/gamma-2	GABRB3 GABRG2 GABRA1	P28472 P18507 P14867
66237	GABA-B receptor	GABBR2 GABBR1	O75899 Q9UBS5
66237	G-protein coupled bile acid receptor 1	GPBAR1	Q8TDU6
66237	Heat shock protein HSP 90-alpha	HSP90AA1	P07900
66237	Heat shock protein HSP 90-beta	HSP90AB1	P08238
66237	Hepatocyte growth factor receptor	MET	P08581
66237	Melanocortin receptor 4	MC4R	P32245
66237	Metabotropic glutamate receptor 2	GRM2	Q14416
66237	Monoamine oxidase B	MAOB	P27338
66237	NAD-dependent deacetylase sirtuin 2	SIRT2	Q8IXJ6
66237	Neurokinin 1 receptor	TACR1	P25103
66237	Nischarin	NISCH	Q9Y2I1
66237	Nitric-oxide synthase, brain	NOS1	P29475
66237	Nitric-oxide synthase, endothelial	NOS3	P29474
66237	Norepinephrine transporter	SLC6A2	P23975
66237	Nuclear receptor ROR-gamma	RORC	P51449
66237	Orexin receptor 1	HCRTR1	O43613
66237	Orexin receptor 2	HCRTR2	O43614
66237	Phosphodiesterase 2A	PDE2A	O00408
66237	Poly [ADP-ribose] polymerase-1	PARP1	P09874
66237	Progesterone receptor	PGR	P06401
66237	Prostaglandin E synthase	PTGES	O14684
66237	Prostanoid IP receptor	PTGIR	P43119
66237	Protein-tyrosine phosphatase 1B	PTPN1	P18031

66237	Serotonin 2a (5-HT2a) receptor	HTR2A	P28223
66237	Serotonin 2b (5-HT2b) receptor	HTR2B	P41595
66237	Serotonin 2c (5-HT2c) receptor	HTR2C	P28335
66237	Serotonin 6 (5-HT6) receptor	HTR6	P50406
66237	Sigma opioid receptor	SIGMAR1	Q99720
66237	Thrombin and coagulation factor X	F10	P00742
66237	Thromboxane A2 receptor	TBXA2R	P21731
66237	Tyrosine-protein kinase JAK3	JAK3	P52333
66237	Vanilloid receptor	TRPV1	Q8NER1
66237	Vascular endothelial growth factor receptor 2	KDR	P35968
66237	Voltage-gated potassium channel subunit Kv1.3	KCNA3	P22001
98009	11-beta-hydroxysteroid dehydrogenase 1	HSD11B1	P28845
98009	Aldo-keto reductase family 1 member B10	AKR1B10	O60218
98009	Androgen Receptor	AR	P10275
98009	ATP-citrate synthase	ACLY	P53396
98009	Bile acid receptor FXR	NR1H4	Q96RI1
98009	Cytochrome P450 19A1	CYP19A1	P11511
98009	DNA polymerase beta (by homology)	POLB	P06746
98009	Dual specificity phosphatase Cdc25A	CDC25A	P30304
98009	Egl nine homolog 1	EGLN1	Q9GZT9
98009	Fatty acid binding protein adipocyte	FABP4	P15090
98009	Fatty acid binding protein epidermal	FABP5	Q01469
98009	Fatty acid binding protein intestinal	FABP2	P12104
98009	Fatty acid binding protein muscle	FABP3	P05413
98009	Free fatty acid receptor 1	FFAR1	O14842
98009	Glucose-6-phosphate 1-dehydrogenase	G6PD	P11413
98009	G-protein coupled bile acid receptor 1	GPBAR1	Q8TDU6
98009	Histone deacetylase 3	HDAC3	O15379
98009	HMG-CoA reductase	HMGCR	P04035
98009	Neuronal acetylcholine receptor protein alpha-7 subunit (by homology)	CHRNA7	P36544
98009	Peroxisome proliferator-activated receptor alpha	PPARA	Q07869
98009	Peroxisome proliferator-activated receptor delta	PPARD	Q03181
98009	Solute carrier family 22 member 6 (by homology)	SLC22A6	Q4U2R8
98009	Vitamin D receptor	VDR	P11473

442879	3-phosphoinositide dependent protein kinase-1	PDPK1	O15530
442879	Acyl-protein thioesterase 1	LYPLA1	O75608
442879	Acyl-protein thioesterase 2	LYPLA2	O95372
442879	Adenosine A2a receptor	ADORA2A	P29274
442879	Adenosine A3 receptor	ADORA3	P0DMS8
442879	Alkaline phosphatase, tissue-nonspecific isozyme	ALPL	P05186
442879	AMP-activated protein kinase, beta-1 subunit	PRKAB1	Q9Y478
442879	Anandamide amidohydrolase	FAAH	O00519
442879	Apoptosis regulator Bcl-2	BCL2	P10415
442879	Arachidonate 12-lipoxygenase	ALOX12	P18054
442879	Arachidonate 15-lipoxygenase	ALOX15	P16050
442879	Arachidonate 5-lipoxygenase	ALOX5	P09917
442879	Carbonic anhydrase IX	CA9	Q16790
442879	Carbonic anhydrase XII	CA12	O43570
442879	Cathepsin S	CTSS	P25774
442879	c-Jun N-terminal kinase 1	MAPK8	P45983
442879	c-Jun N-terminal kinase 2	MAPK9	P45984
442879	c-Jun N-terminal kinase 3	MAPK10	P53779
442879	Cyclin-dependent kinase 2	CDK2	P24941
442879	Cyclin-dependent kinase 2/cyclin A	CDK2 CCNA1 CCNA2	P24941
442879	Cyclin-dependent kinase 5/CDK5 activator 1	CDK5	Q00535
442879	Cytochrome P450 19A1	CYP19A1	P11511
442879	Cytochrome P450 2C9	CYP2C9	P11712
442879	dCTP pyrophosphatase 1	DCTPP1	Q9H773
442879	Dopamine transporter	SLC6A3	Q01959
442879	Dual specificity mitogen-activated protein kinase kinase 1	MAP2K1	Q02750
442879	Dual specificity mitogen-activated protein kinase kinase 5	MAP2K5	Q13163
442879	Dual specificity protein kinase CLK2	CLK2	P49760
442879	Dual specificity protein kinase CLK3	CLK3	P49761
442879	Dual specificity protein kinase CLK4	CLK4	Q9HAZ1
442879	Dual specificty protein kinase CLK1	CLK1	P49759
442879	Endothelin receptor ET-A	EDNRA	P25101
442879	Endothelin receptor ET-B	EDNRB	P24530

442879	Ephrin receptor	EPHB4	P54760
442879	Fibroblast growth factor receptor 3	FGFR3	P22607
442879	GABA-A receptor; alpha-5/beta-3/gamma-2	GABRB3 GABRG2 GABRA5	P28472 P18507 P31644
442879	Glucocorticoid receptor	NR3C1	P04150
442879	Glutaminyl-peptide cyclotransferase	QPCT	Q16769
442879	Glycogen synthase kinase-3 alpha	GSK3A	P49840
442879	Glycogen synthase kinase-3 beta	GSK3B	P49841
442879	G-protein coupled receptor kinase 2	GRK2	P25098
442879	Heat shock protein HSP 90-alpha	HSP90AA1	P07900
442879	Hexokinase type IV	GCK	P35557
442879	Induced myeloid leukemia cell differentiation protein Mcl-1	MCL1	Q07820
442879	Kinesin-1 heavy chain/ Tyrosine-protein kinase receptor RET	RET	P07949
442879	Leucine-rich repeat serine/threonine-protein kinase 2	LRRK2	Q5S007
442879	LIM domain kinase 1	LIMK1	P53667
442879	Macrophage colony stimulating factor receptor	CSF1R	P07333
442879	MAP kinase p38 delta	MAPK13	O15264
442879	MAP kinase-activated protein kinase 2	MAPKAPK2	P49137
442879	MAP kinase-interacting serine/threonine-protein kinase MNK1	MKNK1	Q9BUB5
442879	MAP/microtubule affinity-regulating kinase 2	MARK2	Q7KZI7
442879	Mineralocorticoid receptor	NR3C2	P08235
442879	Mitogen-activated protein kinase 15	MAPK15	Q8TD08
442879	Nerve growth factor receptor Trk-A	NTRK1	P04629
442879	Norepinephrine transporter	SLC6A2	P23975
442879	Phosphatidylinositol-4-phosphate 3-kinase C2 domain-containing beta polypeptide	PIK3C2B	O00750
442879	Phosphatidylinositol-4-phosphate 3-kinase C2 domain-containing subunit gamma	PIK3C2G	O75747
442879	Phosphatidylinositol-5-phosphate 4-kinase type-2 gamma	PIP4K2C	Q8TBX8
442879	Phospholipase A2 group IIA	PLA2G2A	P14555
442879	PI3-kinase p110-alpha subunit	PIK3CA	P42336
442879	PI3-kinase p110-alpha/p85-alpha	PIK3CA PIK3R1	P42336 P27986
442879	PI3-kinase p110-beta subunit	PIK3CB	P42338

442879	PI3-kinase p110-delta subunit	PIK3CD	O00329
442879	PI3-kinase p110-gamma subunit	PIK3CG	P48736
442879	Poly [ADP-ribose] polymerase-1	PARP1	P09874
442879	Potassium-transporting ATPase	ATP4B ATP4A	P51164 P20648
442879	Receptor protein-tyrosine kinase erbB-2	ERBB2	P04626
442879	Receptor protein-tyrosine kinase erbB-4	ERBB4	Q15303
442879	Rho-associated protein kinase 1	ROCK1	Q13464
442879	Rho-associated protein kinase 2	ROCK2	O75116
442879	Ribosomal protein S6 kinase alpha 3	RPS6KA3	P51812
442879	Serine/threonine-protein kinase Aurora-A	AURKA	O14965
442879	Serine/threonine-protein kinase Aurora-B	AURKB	Q96GD4
442879	Serine/threonine-protein kinase B-raf	BRAF	P15056
442879	Serine/threonine-protein kinase Chk1	CHEK1	O14757
442879	Serine/threonine-protein kinase Chk2	CHEK2	O96017
442879	Serine/threonine-protein kinase c-TAK1	MARK3	P27448
442879	Serine/threonine-protein kinase D2	PRKD2	Q9BZL6
442879	Serine/threonine-protein kinase MST2	STK3	Q13188
442879	Serine/threonine-protein kinase mTOR	MTOR	P42345
442879	Serine/threonine-protein kinase NEK2	NEK2	P51955
442879	Serine/threonine-protein kinase PLK1	PLK1	P53350
442879	Serine/threonine-protein kinase PLK2	PLK2	Q9NYY3
442879	Serine/threonine-protein kinase PLK3	PLK3	Q9H4B4
442879	Serine/threonine-protein kinase PLK4	PLK4	O00444
442879	Serine/threonine-protein kinase RAF	RAF1	P04049
442879	Serine/threonine-protein kinase receptor R3	ACVRL1	P37023
442879	Serine/threonine-protein kinase Sgk1	SGK1	O00141
442879	Sodium/hydrogen exchanger 1	SLC9A1	P19634
442879	SPS1/STE20-related protein kinase YSK4	MAP3K19	Q56UN5
442879	SUMO-activating enzyme	SAE1 UBA2	Q9UBE0 Q9UBT2
442879	Testis-specific androgen-binding protein	SHBG	P04278
442879	Thymidylate synthase	TYMS	P04818
442879	Tyrosine-protein kinase ABL	ABL1	P00519
442879	Tyrosine-protein kinase BTK	BTK	Q06187
442879	Tyrosine-protein kinase JAK1	JAK1	P23458

442879	Tyrosine-protein kinase JAK2	JAK2	O60674
442879	Vascular endothelial growth factor receptor 1	FLT1	P17948
442879	X-box-binding protein 1	XBP1	P17861
445638	11-beta-hydroxysteroid dehydrogenase 1	HSD11B1	P28845
445638	11-beta-hydroxysteroid dehydrogenase 2	HSD11B2	P80365
445638	Acetylcholinesterase	ACHE	P22303
445638	Acyl-CoA desaturase	SCD	O00767
445638	Adenosine A3 receptor	ADORA3	P0DMS8
445638	Aldo-keto reductase family 1 member B10	AKR1B10	O60218
445638	Anandamide amidohydrolase	FAAH	O00519
445638	Androgen Receptor	AR	P10275
445638	Apoptosis regulator Bcl-2	BCL2	P10415
445638	Arachidonate 12-lipoxygenase	ALOX12	P18054
445638	Arachidonate 5-lipoxygenase	ALOX5	P09917
445638	Autotaxin	ENPP2	Q13822
445638	Beta-secretase 1	BACE1	P56817
445638	Bile acid receptor FXR	NR1H4	Q96RI1
445638	Butyrylcholinesterase	BCHE	P06276
445638	Cannabinoid receptor 1	CNR1	P21554
445638	Carboxylesterase 2	CES2	O00748
445638	Cathepsin G	CTSG	P08311
445638	CD81 antigen	CD81	P60033
445638	Chymase	CMA1	P23946
445638	Corticosteroid binding globulin	SERPINA6	P08185
445638	Cyclooxygenase-1	PTGS1	P23219
445638	Cyclooxygenase-2	PTGS2	P35354
445638	Cytochrome P450 17A1	CYP17A1	P05093
445638	Cytochrome P450 19A1	CYP19A1	P11511
445638	Cytochrome P450 26A1	CYP26A1	O43174
445638	Cytochrome P450 26B1	CYP26B1	Q9NR63
445638	Cytochrome P450 2C19	CYP2C19	P33261
445638	Cytochrome P450 51 (by homology)	CYP51A1	Q16850
445638	DNA polymerase beta	POLB	P06746
445638	DNA topoisomerase I	TOP1	P11387

445638	DNA topoisomerase II alpha	TOP2A	P11388
445638	Dopamine transporter	SLC6A3	Q01959
445638	Dual specificity phosphatase Cdc25A	CDC25A	P30304
445638	Dual specificity phosphatase Cdc25B	CDC25B	P30305
445638	Endothelin receptor ET-A (by homology)	EDNRA	P25101
445638	Estrogen receptor alpha	ESR1	P03372
445638	Estrogen receptor beta	ESR2	Q92731
445638	Fatty acid binding protein adipocyte	FABP4	P15090
445638	Fatty acid binding protein epidermal	FABP5	Q01469
445638	Fatty acid binding protein intestinal	FABP2	P12104
445638	Fatty acid binding protein muscle	FABP3	P05413
445638	Fatty acid-binding protein, liver	FABP1	P07148
445638	Free fatty acid receptor 1	FFAR1	O14842
445638	G protein-coupled receptor 44	PTGDR2	Q9Y5Y4
445638	Gamma-secretase	PSEN2 PSENEN NCSTN APH1A PSEN1 APH1B	P49810 Q9NZ42 Q92542 Q96BI3 P49768 Q8WW43
445638	Glucocorticoid receptor	NR3C1	P04150
445638	Glucose-6-phosphate 1-dehydrogenase	G6PD	P11413
445638	G-protein coupled receptor 120	FFAR4	Q5NUL3
445638	Hepatocyte nuclear factor 4-alpha	HNF4A	P41235
445638	HMG-CoA reductase	HMGCR	P04035
445638	Induced myeloid leukemia cell differentiation protein Mcl-1	MCL1	Q07820
445638	Inosine-5'-monophosphate dehydrogenase 2	IMPDH2	P12268
445638	Leukotriene B4 receptor 1	LTB4R	Q15722
445638	Low molecular weight phosphotyrosine protein phosphatase	ACP1	P24666
445638	LXR-alpha	NR1H3	Q13133
445638	MAP kinase ERK1	MAPK3	P27361
445638	Mineralocorticoid receptor	NR3C2	P08235
445638	Monocarboxylate transporter 1 (by homology)	SLC16A1	P53985
445638	Muscarinic acetylcholine receptor M2	CHRM2	P08172
445638	Niemann-Pick C1-like protein 1	NPC1L1	Q9UHC9
445638	Nitric oxide synthase, inducible	NOS2	P35228
445638	Norepinephrine transporter	SLC6A2	P23975

445638	Nuclear receptor ROR-alpha	RORA	P35398
445638	Nuclear receptor ROR-gamma	RORC	P51449
445638	Nuclear receptor subfamily 1 group I member 3 (by homology)	NR1I3	Q14994
445638	Oxoeicosanoid receptor 1	OXER1	Q8TDS5
445638	p53-binding protein Mdm-2	MDM2	Q00987
445638	Peroxisome proliferator-activated receptor alpha	PPARA	Q07869
445638	Peroxisome proliferator-activated receptor delta	PPARD	Q03181
445638	Peroxisome proliferator-activated receptor gamma	PPARG	P37231
445638	Phosphodiesterase 4D	PDE4D	Q08499
445638	Phospholipase A2 group 1B	PLA2G1B	P04054
445638	Progesterone receptor	PGR	P06401
445638	Prolyl endopeptidase	PREP	P48147
445638	Prostaglandin E synthase	PTGES	O14684
445638	Prostanoid DP receptor	PTGDR	Q13258
445638	Prostanoid EP1 receptor	PTGER1	P34995
445638	Prostanoid EP2 receptor	PTGER2	P43116
445638	Prostanoid EP3 receptor	PTGER3	P43115
445638	Prostanoid EP4 receptor	PTGER4	P35408
445638	Prostanoid IP receptor	PTGIR	P43119
445638	Protein farnesyltransferase	FNTA FNTB	P49354 P49356
445638	Protein kinase C eta	PRKCH	P24723
445638	Protein-tyrosine phosphatase 1B	PTPN1	P18031
445638	Protein-tyrosine phosphatase 1C	PTPN6	P29350
445638	Protein-tyrosine phosphatase 2C	PTPN11	Q06124
445638	Receptor-type tyrosine-protein phosphatase F (LAR)	PTPRF	P10586
445638	Serine/threonine-protein kinase PIM2	PIM2	Q9P1W9
445638	Serotonin transporter	SLC6A4	P31645
445638	Sigma opioid receptor	SIGMAR1	Q99720
445638	Solute carrier family 22 member 12	SLC22A12	Q96S37
445638	Solute carrier family 22 member 6 (by homology)	SLC22A6	Q4U2R8
445638	Squalene synthetase	FDFT1	P37268
445638	Steroid 5-alpha-reductase 2	SRD5A2	P31213
445638	T-cell protein-tyrosine phosphatase	PTPN2	P17706
445638	Telomerase reverse transcriptase	TERT	O14746

445638	Testis-specific androgen-binding protein	SHBG	P04278
445638	Transient receptor potential cation channel subfamily M member 8	TRPM8	Q7Z2W7
445638	Vanilloid receptor	TRPV1	Q8NER1
639142	Aldose reductase	AKR1B1	P15121
639142	Arachidonate 5-lipoxygenase	ALOX5	P09917
639142	Carbonic anhydrase I	CA1	P00915
639142	Carbonic anhydrase II	CA2	P00918
639142	Carbonic anhydrase IX	CA9	Q16790
639142	Carbonic anhydrase XII	CA12	O43570
639142	Cyclin-dependent kinase 2	CDK2	P24941
639142	Epidermal growth factor receptor erbB1	EGFR	P00533
639142	Epoxide hydrolase 1	EPHX1	P07099
639142	Fructose-1,6-bisphosphatase	FBP1	P09467
639142	G protein-coupled receptor 44	PTGDR2	Q9Y5Y4
639142	Glycogen synthase kinase-3 beta	GSK3B	P49841
639142	Lysine-specific demethylase 4B	KDM4B	O94953
639142	Lysine-specific demethylase 4C	KDM4C	Q9H3R0
639142	MAP kinase-interacting serine/threonine-protein kinase MNK1	MKNK1	Q9BUB5
639142	Matrix metalloproteinase 13	MMP13	P45452
639142	Matrix metalloproteinase 3	MMP3	P08254
639142	Monoamine oxidase A	MAOA	P21397
639142	Muscarinic acetylcholine receptor M1	CHRM1	P11229
639142	Neprilysin (by homology)	MME	P08473
639142	Nicotinamide phosphoribosyltransferase	NAMPT	P43490
639142	PI3-kinase p110-delta subunit	PIK3CD	O00329
639142	PI3-kinase p110-gamma subunit	PIK3CG	P48736
639142	Serotonin 2c (5-HT2c) receptor	HTR2C	P28335
5282375	Acetyl-CoA carboxylase 2	ACACB	O00763
5282375	Bile acid receptor FXR	NR1H4	Q96RI1
5282375	Glucose-6-phosphate 1-dehydrogenase	G6PD	P11413
5282375	MAP kinase-activated protein kinase 2	MAPKAPK2	P49137
5282375	Metabotropic glutamate receptor 5 (by homology)	GRM5	P41594
5282375	Orexin receptor 1	HCRTR1	O43613

5282375	Orexin receptor 2	HCRTR2	O43614
5282375	Retinoid X receptor alpha	RXRA	P19793
5282375	Steroid 5-alpha-reductase 1	SRD5A1	P18405
5282375	Steroid 5-alpha-reductase 2	SRD5A2	P31213
5312775	11-beta-hydroxysteroid dehydrogenase 1	HSD11B1	P28845
5312775	11-beta-hydroxysteroid dehydrogenase 2	HSD11B2	P80365
5312775	Acetyl-CoA carboxylase 2	ACACB	O00763
5312775	Acyl-CoA desaturase	SCD	O00767
5312775	ADAM17	ADAM17	P78536
5312775	Adenosine A3 receptor	ADORA3	P0DMS8
5312775	Aldo-keto reductase family 1 member B10	AKR1B10	O60218
5312775	Anandamide amidohydrolase	FAAH	O00519
5312775	Apoptosis regulator Bcl-2	BCL2	P10415
5312775	Apoptosis regulator Bcl-W	BCL2L2	Q92843
5312775	Arachidonate 5-lipoxygenase	ALOX5	P09917
5312775	Bcl-2-related protein A1	BCL2A1	Q16548
5312775	Cannabinoid receptor 1 (by homology)	CNR1	P21554
5312775	Carboxylesterase 2	CES2	O00748
5312775	Casein kinase II alpha	CSNK2A1	P68400
5312775	Cathepsin G	CTSG	P08311
5312775	CD81 antigen	CD81	P60033
5312775	Chymase	CMA1	P23946
5312775	c-Jun N-terminal kinase 1	MAPK8	P45983
5312775	c-Jun N-terminal kinase 3	MAPK10	P53779
5312775	Corticosteroid binding globulin	SERPINA6	P08185
5312775	Cyclooxygenase-1	PTGS1	P23219
5312775	Cyclooxygenase-2	PTGS2	P35354
5312775	Cytochrome P450 17A1	CYP17A1	P05093
5312775	Cytochrome P450 19A1	CYP19A1	P11511
5312775	Cytochrome P450 51 (by homology)	CYP51A1	Q16850
5312775	DNA polymerase beta	POLB	P06746
5312775	DNA topoisomerase I	TOP1	P11387
5312775	Dual specificity phosphatase Cdc25A	CDC25A	P30304
5312775	Dual specificity phosphatase Cdc25B	CDC25B	P30305

5312775	Dual specificity protein phosphatase 3	DUSP3	P51452
5312775	Estrogen receptor alpha	ESR1	P03372
5312775	Estrogen receptor beta	ESR2	Q92731
5312775	Fatty acid binding protein adipocyte	FABP4	P15090
5312775	Fatty acid binding protein epidermal	FABP5	Q01469
5312775	Fatty acid binding protein muscle	FABP3	P05413
5312775	Fatty acid-binding protein, liver	FABP1	P07148
5312775	Free fatty acid receptor 1	FFAR1	O14842
5312775	G protein-coupled receptor 44	PTGDR2	Q9Y5Y4
5312775	G protein-coupled receptor kinase 6	GRK6	P43250
5312775	Glucagon receptor	GCGR	P47871
5312775	Glucose-6-phosphate 1-dehydrogenase	G6PD	P11413
5312775	Glutamine synthetase	GLUL	P15104
5312775	Glycogen synthase kinase-3 alpha	GSK3A	P49840
5312775	Glycogen synthase kinase-3 beta	GSK3B	P49841
5312775	G-protein coupled bile acid receptor 1	GPBAR1	Q8TDU6
5312775	Hematopoietic cell protein-tyrosine phosphatase 70Z-PEP	PTPN22	Q9Y2R2
5312775	Hepatocyte nuclear factor 4-alpha	HNF4A	P41235
5312775	HMG-CoA reductase	HMGCR	P04035
5312775	Induced myeloid leukemia cell differentiation protein Mcl-1	MCL1	Q07820
5312775	Inosine-5'-monophosphate dehydrogenase 2	IMPDH2	P12268
5312775	Interleukin-6	IL6	P05231
5312775	Leukotriene B4 receptor 1	LTB4R	Q15722
5312775	Low molecular weight phosphotyrosine protein phosphatase	ACP1	P24666
5312775	MAP kinase p38 alpha	MAPK14	Q16539
5312775	Niemann-Pick C1-like protein 1	NPC1L1	Q9UHC9
5312775	Nitric oxide synthase, inducible	NOS2	P35228
5312775	Nuclear receptor ROR-gamma	RORC	P51449
5312775	Peptidyl-glycine alpha-amidating monooxygenase	PAM	P19021
5312775	Peptidyl-prolyl cis-trans isomerase NIMA-interacting 1	PIN1	Q13526
5312775	Peroxisome proliferator-activated receptor alpha	PPARA	Q07869
5312775	Peroxisome proliferator-activated receptor delta	PPARD	Q03181

5312775	Peroxisome proliferator-activated receptor gamma	PPARG	P37231
5312775	Phosphodiesterase 4D	PDE4D	Q08499
5312775	Phospholipase A2 group 1B	PLA2G1B	P04054
5312775	Plasminogen activator inhibitor-1	SERPINE1	P05121
5312775	Prenyl protein specific protease	RCE1	Q9Y256
5312775	Progesterone receptor	PGR	P06401
5312775	Prolyl endopeptidase	PREP	P48147
5312775	Prostaglandin E synthase	PTGES	O14684
5312775	Prostanoid EP1 receptor	PTGER1	P34995
5312775	Prostanoid EP2 receptor	PTGER2	P43116
5312775	Prostanoid EP4 receptor	PTGER4	P35408
5312775	Prostanoid IP receptor	PTGIR	P43119
5312775	Protein farnesyltransferase	FNTA FNTB	P49354 P49356
5312775	Protein kinase C eta	PRKCH	P24723
5312775	Protein-tyrosine phosphatase 1B	PTPN1	P18031
5312775	Protein-tyrosine phosphatase 1C	PTPN6	P29350
5312775	Protein-tyrosine phosphatase 2C	PTPN11	Q06124
5312775	Protein-tyrosine phosphatase LC-PTP	PTPN7	P35236
5312775	Receptor-type tyrosine-protein phosphatase F (LAR)	PTPRF	P10586
5312775	Retinoid X receptor alpha	RXRA	P19793
5312775	Serine/threonine-protein kinase Chk1	CHEK1	O14757
5312775	Serine/threonine-protein kinase Chk2	CHEK2	O96017
5312775	Solute carrier family 22 member 12	SLC22A12	Q96S37
5312775	Squalene synthetase (by homology)	FDFT1	P37268
5312775	T-cell protein-tyrosine phosphatase	PTPN2	P17706
5312775	Telomerase reverse transcriptase	TERT	O14746
5312775	Testis-specific androgen-binding protein	SHBG	P04278
5312775	TGF-beta receptor type I	TGFBR1	P36897
5312775	Thromboxane-A synthase	TBXAS1	P24557
5312775	Thyroid hormone receptor alpha	THRA	P10827
5312775	Thyroid hormone receptor beta-1	THRB	P10828
5312775	Type-1 angiotensin II receptor	AGTR1	P30556
5312775	Tyrosine-protein phosphatase non-receptor type 9	PTPN9	P43378
36689743	11-beta-hydroxysteroid dehydrogenase 1	HSD11B1	P28845

36689743	Acetylcholinesterase	ACHE	P22303
36689743	Acyl coenzyme A:cholesterol acyltransferase	CES1	P23141
36689743	Acyl-CoA:dihydroxyacetonephosphateacyltransferase	GNPAT	O15228
36689743	Adenosine A1 receptor (by homology)	ADORA1	P30542
36689743	Aldehyde reductase (by homology)	AKR1A1	P14550
36689743	Aldo-keto reductase family 1 member B10	AKR1B10	O60218
36689743	Aldo-keto reductase family 1 member C2	AKR1C2	P52895
36689743	Aldo-keto-reductase family 1 member C3	AKR1C3	P42330
36689743	Aldose reductase (by homology)	AKR1B1	P15121
36689743	Aminopeptidase B	RNPEP	Q9H4A4
36689743	Angiotensin-converting enzyme	ACE	P12821
36689743	Angiotensin-converting enzyme 2	ACE2	Q9BYF1
36689743	Aspartyl aminopeptidase	DNPEP	Q9ULA0
36689743	Beta-secretase 1	BACE1	P56817
36689743	Bile acid receptor FXR	NR1H4	Q96RI1
36689743	Branched-chain-amino-acid aminotransferase, mitochondrial	BCAT2	O15382
36689743	Calpain 1	CAPN1	P07384
36689743	Cannabinoid receptor 1 (by homology)	CNR1	P21554
36689743	Cathepsin (B and K)	CTSB	P07858
36689743	CREB-binding protein/p53	CREBBP	Q92793
36689743	Cyclooxygenase-1	PTGS1	P23219
36689743	Cyclooxygenase-2	PTGS2	P35354
36689743	Cytosolic phospholipase A2	PLA2G4A	P47712
36689743	Dipeptidyl peptidase IV	DPP4	P27487
36689743	Disks large homolog 4	DLG4	P78352
36689743	Dual specificity mitogen-activated protein kinase kinase 1	MAP2K1	Q02750
36689743	Endothelin receptor ET-A (by homology)	EDNRA	P25101
36689743	Epidermal growth factor receptor erbB1	EGFR	P00533
36689743	Epoxide hydratase	EPHX2	P34913
36689743	Estradiol 17-beta-dehydrogenase 3	HSD17B3	P37058
36689743	Estrogen receptor beta	ESR2	Q92731
36689743	Fatty acid-binding protein, liver (by homology)	FABP1	P07148
36689743	G protein-coupled receptor 44	PTGDR2	Q9Y5Y4

36689743	Glutathione S-transferase A1	GSTA1	P08263
36689743	Glutathione S-transferase Pi	GSTP1	P09211
36689743	G-protein coupled receptor 35	GPR35	Q9HC97
36689743	G-protein coupled receptor kinase 2	GRK2	P25098
36689743	Heat shock 70 kDa protein 1	HSPA1A	P0DMV8
36689743	Heat shock protein HSP 90-alpha	HSP90AA1	P07900
36689743	Hepatocyte growth factor receptor	MET	P08581
36689743	HLA class I histocompatibility antigen A-3	HLA-A	P04439
36689743	Hydroxycarboxylic acid receptor 2	HCAR2	Q8TDS4
36689743	Inhibitor of apoptosis protein 3	XIAP	P98170
36689743	Integrin alpha1/beta1 complex	ITGB1 ITGA1	P05556 P56199
36689743	Integrin alpha2/beta1	ITGB1 ITGA2	P05556 P17301
36689743	Integrin alpha-IIb/beta-3	ITGA2B ITGB3	P08514 P05106
36689743	Intercellular adhesion molecule-1	ICAM1	P05362
36689743	Interleukin-8	CXCL8	P10145
36689743	Interleukin-8 receptor A	CXCR1	P25024
36689743	Kynurenine 3-monooxygenase	KMO	O15229
36689743	Leucine aminopeptidase	LAP3	P28838
36689743	Leukotriene A4 hydrolase	LTA4H	P09960
36689743	Lysine-specific demethylase 4D	KDM4D	Q6B0I6
36689743	Lysine-specific demethylase 4D-like	KDM4E	B2RXH2
36689743	Lysosomal protective protein	CTSA	P10619
36689743	Macrophage migration inhibitory factor	MIF	P14174
36689743	MAP kinase ERK2	MAPK1	P28482
36689743	MAP kinase p38 alpha	MAPK14	Q16539
36689743	MAP kinase signal-integrating kinase 2	MKNK2	Q9HBH9
36689743	MAP kinase-interacting serine/threonine-protein kinase MNK1	MKNK1	Q9BUB5
36689743	Matrix metalloproteinase 2	MMP2	P08253
36689743	Metabotropic glutamate receptor 4	GRM4	Q14833
36689743	Mitogen-activated protein kinase kinase kinase 8	MAP3K8	P41279
36689743	Monoamine oxidase B	MAOB	P27338
36689743	Multidrug resistance-associated protein 1	ABCC1	P33527
36689743	Muscle glycogen phosphorylase	PYGM	P11217

36689743	N-acylsphingosine-amidohydrolase (by homology)	NAAA	Q02083
36689743	NAD-dependent deacetylase sirtuin 2	SIRT2	Q8IXJ6
36689743	Neprilysin	MME	P08473
36689743	Neurotensin receptor 3	SORT1	Q99523
36689743	Nuclear receptor subfamily 4 group A member 1	NR4A1	P22736
36689743	Palmitoleoyl-protein carboxylesterase NOTUM	NOTUM	Q6P988
36689743	Pepsinogen C (by homology)	PGC	P20142
36689743	Peptidyl-glycine alpha-amidating monooxygenase	PAM	P19021
36689743	Peroxisome proliferator-activated receptor delta	PPARD	Q03181
36689743	Peroxisome proliferator-activated receptor gamma	PPARG	P37231
36689743	Phosphodiesterase 7A	PDE7A	Q13946
36689743	Phosphodiesterase 9A	PDE9A	O76083
36689743	Phospholipase A2 group IIA	PLA2G2A	P14555
36689743	Phospholipase A2 group V	PLA2G5	P39877
36689743	Plectin	PLEC	Q15149
36689743	Poly [ADP-ribose] polymerase 10	PARP10	Q53GL7
36689743	Poly [ADP-ribose] polymerase 15	PARP15	Q460N3
36689743	Poly [ADP-ribose] polymerase-1	PARP1	P09874
36689743	Prostanoid DP receptor	PTGDR	Q13258
36689743	Prostanoid EP1 receptor	PTGER1	P34995
36689743	Protein kinase C alpha	PRKCA	P17252
36689743	Renin	REN	P00797
36689743	Selectin E	SELE	P16581
36689743	Serine/threonine protein phosphatase PP1-alpha catalytic subunit	PPP1CA	P62136
36689743	Serine/threonine-protein phosphatase	PPP5C	Q9BPW0
36689743	Sodium/glucose cotransporter 1	SLC5A1	P13866
36689743	Solute carrier family 22 member 12	SLC22A12	Q96S37
36689743	Solute carrier organic anion transporter family member 1B1	SLCO1B1	Q9Y6L6
36689743	Thrombin	F2	P00734
36689743	Thromboxane-A synthase	TBXAS1	P24557
36689743	Thyroid hormone receptor beta-1	THRB	P10828
36689743	Transthyretin	TTR	P02766
36689743	Vitronectin receptor alpha	ITGAV	P06756

46178008	11-beta-hydroxysteroid dehydrogenase 1	HSD11B1	P28845
46178008	Adenosine A1 receptor (by homology)	ADORA1	P30542
46178008	Adenosine A2a receptor (by homology)	ADORA2A	P29274
46178008	Bile acid receptor FXR	NR1H4	Q96RI1
46178008	Bromodomain-containing protein 4	BRD4	O60885
46178008	Cannabinoid receptor 1 (by homology)	CNR1	P21554
46178008	Cannabinoid receptor 2	CNR2	P34972
46178008	Carnitine O-palmitoyltransferase 1, liver isoform	CPT1A	P50416
46178008	Carnitine O-palmitoyltransferase 1, muscle isoform	CPT1B	Q92523
46178008	Carnitine palmitoyltransferase 2	CPT2	P23786
46178008	Casein kinase II alpha	CSNK2A1	P68400
46178008	Caspase-1	CASP1	P29466
46178008	Cholecystokinin B receptor (by homology)	CCKBR	P32239
46178008	Chymase	CMA1	P23946
46178008	Cyclin-dependent kinase 1/cyclin B	CCNB3 CDK1 CCNB1 CCNB2	Q8WWL7 P06493 P14635 O95067
46178008	Cyclin-dependent kinase 2/cyclin A	CDK2 CCNA1 CCNA2	P24941
46178008	Cyclin-dependent kinase 9	CDK9	P50750
46178008	Cyclooxygenase-2	PTGS2	P35354
46178008	Cytochrome P450 26A1	CYP26A1	O43174
46178008	Cytochrome P450 26B1	CYP26B1	Q9NR63
46178008	Epoxide hydratase	EPHX2	P34913
46178008	GABA transporter 1	SLC6A1	P30531
46178008	Glycogen synthase kinase-3 alpha	GSK3A	P49840
46178008	Glycogen synthase kinase-3 beta	GSK3B	P49841
46178008	G-protein coupled bile acid receptor 1	GPBAR1	Q8TDU6
46178008	G-protein coupled receptor 55	GPR55	Q9Y2T6
46178008	Hematopoietic cell protein-tyrosine phosphatase 70Z-PEP	PTPN22	Q9Y2R2
46178008	Hepatocyte nuclear factor 4-alpha	HNF4A	P41235
46178008	HMG-CoA reductase (by homology)	HMGCR	P04035
46178008	Inosine-5'-monophosphate dehydrogenase 1	IMPDH1	P20839
46178008	Inosine-5'-monophosphate dehydrogenase 2	IMPDH2	P12268
46178008	Integrin alpha-V/beta-3	ITGAV ITGB3	P06756 P05106

46178008	Macrophage colony stimulating factor receptor (by homology)	CSF1R	P07333
46178008	Melatonin receptor 1A	MTNR1A	P48039
46178008	Melatonin receptor 1B	MTNR1B	P49286
46178008	Peroxisome proliferator-activated receptor alpha	PPARA	Q07869
46178008	Peroxisome proliferator-activated receptor delta	PPARD	Q03181
46178008	Plasma retinol-binding protein	RBP4	P02753
46178008	Prostanoid DP receptor	PTGDR	Q13258
46178008	Prostanoid EP3 receptor	PTGER3	P43115
46178008	Protein-tyrosine phosphatase 1C	PTPN6	P29350
46178008	Protein-tyrosine phosphatase 2C	PTPN11	Q06124
46178008	Serotonin 2a (5-HT2a) receptor (by homology)	HTR2A	P28223
46178008	TGF-beta receptor type I	TGFBR1	P36897
46178008	Thromboxane-A synthase	TBXAS1	P24557
46178008	Thyroid hormone receptor alpha	THRA	P10827
46178008	Type-1 angiotensin II receptor (by homology)	AGTR1	P30556
46178008	Tyrosine-protein phosphatase non-receptor type 9	PTPN9	P43378
54678431	Tyrosine-protein phosphatase non-receptor type 2		P17706
88833376	Acetyl-CoA carboxylase 2	ACACB	O00763
88833376	Alpha-1,6-mannosyl-glycoprotein 2-beta-N-acetylglucosaminyltransferase	MGAT2	Q10469
88833376	Bile acid receptor FXR	NR1H4	Q96RI1
88833376	Diacylglycerol O-acyltransferase 2	DGAT2	Q96PD7
88833376	Glucose-6-phosphate 1-dehydrogenase	G6PD	P11413
88833376	HERG	KCNH2	Q12809
88833376	MAP kinase-activated protein kinase 2	MAPKAPK2	P49137
88833376	Metabotropic glutamate receptor 5 (by homology)	GRM5	P41594
88833376	Orexin receptor 1	HCRTR1	O43613
88833376	Orexin receptor 2	HCRTR2	O43614
88833376	Steroid 5-alpha-reductase 1	SRD5A1	P18405
88833376	Steroid 5-alpha-reductase 2	SRD5A2	P31213
101560606	Reverse transcriptase		Q06347
129846263	Aldose reductase	AKR1B1	P15121
129846263	Carbonic anhydrase I	CA1	P00915
129846263	Carbonic anhydrase II	CA2	P00918

129846263	Carbonic anhydrase III	CA3	P07451
129846263	Carbonic anhydrase IV	CA4	P22748
129846263	Carbonic anhydrase IX	CA9	Q16790
129846263	Carbonic anhydrase VA	CA5A	P35218
129846263	Carbonic anhydrase VB	CA5B	Q9Y2D0
129846263	Carbonic anhydrase VI	CA6	P23280
129846263	Carbonic anhydrase VII	CA7	P43166
129846263	Carbonic anhydrase XII	CA12	O43570
129846263	Carbonic anhydrase XIII (by homology)	CA13	Q8N1Q1
129846263	Carbonic anhydrase XIV	CA14	Q9ULX7
129846263	Carboxypeptidase A1	CPA1	P15085
129846263	Epidermal growth factor receptor erbB1	EGFR	P00533
129846263	Estrogen receptor beta	ESR2	Q92731
129846263	Fructose-1,6-bisphosphatase	FBP1	P09467
129846263	Macrophage migration inhibitory factor	MIF	P14174
129846263	Tyrosine-protein kinase FYN	FYN	P06241
129846263	Tyrosine-protein kinase LCK	LCK	P06239
129846263	Uracil nucleotide/cysteinyl leukotriene receptor	GPR17	Q13304
162870052	Cannabinoid receptor 1		P21554
162870052	Protein kinase C alpha type	PRKCA	P04409

Table S4

Gathering of breast cancer target genes using DisGent database			
Disease	Gene	Gene UniProt ID	Gene_Full_Name
Malignant neoplasm of breast	CAMTA1	Q9Y6Y1	calmodulin binding transcription activator 1
Malignant neoplasm of breast	MORC2	Q9Y6X9	MORC family CW-type zinc finger 2
Malignant neoplasm of breast	ZHX2	Q9Y6X8	zinc fingers and homeoboxes 2
Malignant neoplasm of breast	PIAS3	Q9Y6X2	protein inhibitor of activated STAT 3
Malignant neoplasm of breast	SETBP1	Q9Y6X0	SET binding protein 1
Malignant neoplasm of breast	ICOS	Q9Y6W8	inducible T cell costimulator
Malignant neoplasm of breast	DUSP10	Q9Y6W6	dual specificity phosphatase 10
Malignant neoplasm of breast	WASF2	Q9Y6W5	WASP family member 2
Malignant neoplasm of breast	SCIN	Q9Y6U3	scinderin
Malignant neoplasm of breast	MAP3K4	Q9Y6R4	mitogen-activated protein kinase kinase kinase 4
Malignant neoplasm of breast	NCOA3	Q9Y6Q9	nuclear receptor coactivator 3
Malignant neoplasm of breast	TNFRSF11A	Q9Y6Q6	TNF receptor superfamily member 11a
Malignant neoplasm of breast	CDH10	Q9Y6N8	cadherin 10
Malignant neoplasm of breast	ROBO1	Q9Y6N7	roundabout guidance receptor 1
Malignant neoplasm of breast	COX11	Q9Y6N1	cytochrome c oxidase copper chaperone COX11
Malignant neoplasm of breast	NDUFB9	Q9Y6M9	NADH:ubiquinone oxidoreductase subunit B9

Malignant neoplasm of breast	SLC4A7	Q9Y6M7	solute carrier family 4 member 7
Malignant neoplasm of breast	SLC30A1	Q9Y6M5	solute carrier family 30 member 1
Malignant neoplasm of breast	IGF2BP2	Q9Y6M1	insulin like growth factor 2 mRNA binding protein 2
Malignant neoplasm of breast	SLCO1B1	Q9Y6L6	solute carrier organic anion transporter family member 1B1
Malignant neoplasm of breast	IKBKG	Q9Y6K9	inhibitor of nuclear factor kappa B kinase regulatory subunit gamma
Malignant neoplasm of breast	OAS3	Q9Y6K5	2'-5'-oligoadenylate synthetase 3
Malignant neoplasm of breast	DNMT3A	Q9Y6K1	DNA methyltransferase 3 alpha
Malignant neoplasm of breast	CABIN1	Q9Y6J0	calcineurin binding protein 1
Malignant neoplasm of breast	WSB1	Q9Y6I7	WD repeat and SOCS box containing 1
Malignant neoplasm of breast	USP3	Q9Y6I4	ubiquitin specific peptidase 3
Malignant neoplasm of breast	GJA3	Q9Y6H8	gap junction protein alpha 3
Malignant neoplasm of breast	CHCHD2	Q9Y6H1	coiled-coil-helix-coiled-coil-helix domain containing 2
Malignant neoplasm of breast	WNT6	Q9Y6F9	Wnt family member 6
Malignant neoplasm of breast	PARP3	Q9Y6F1	poly(ADP-ribose) polymerase family member 3
Malignant neoplasm of breast	SIRT4	Q9Y6E7	sirtuin 4
Malignant neoplasm of breast	STK24	Q9Y6E0	serine/threonine kinase 24
Malignant neoplasm of breast	MAD1L1	Q9Y6D9	mitotic arrest deficient 1 like 1
Malignant neoplasm of breast	ARFGEF1	Q9Y6D6	ADP ribosylation factor guanine nucleotide exchange factor 1
Malignant neoplasm of breast	ARFGEF2	Q9Y6D5	ADP ribosylation factor guanine nucleotide exchange factor 2

Malignant neoplasm of breast	CFAP20	Q9Y6A4	cilia and flagella associated protein 20
Malignant neoplasm of breast	CACNG2	Q9Y698	calcium voltage-gated channel auxiliary subunit gamma 2
Malignant neoplasm of breast	CLIC4	Q9Y696	chloride intracellular channel 4
Malignant neoplasm of breast	SLC12A7	Q9Y666	solute carrier family 12 member 7
Malignant neoplasm of breast	HS3ST3A1	Q9Y663	heparan sulfate-glucosamine 3-sulfotransferase 3A1
Malignant neoplasm of breast	HS3ST3B1	Q9Y662	heparan sulfate-glucosamine 3-sulfotransferase 3B1
Malignant neoplasm of breast	SPIN1	Q9Y657	spindlin 1
Malignant neoplasm of breast	CPQ	Q9Y646	carboxypeptidase Q
Malignant neoplasm of breast	NPTN	Q9Y639	neuroplastin
Malignant neoplasm of breast	GPC6	Q9Y625	glypican 6
Malignant neoplasm of breast	F11R	Q9Y624	F11 receptor
Malignant neoplasm of breast	NCOR2	Q9Y618	nuclear receptor corepressor 2
Malignant neoplasm of breast	PSAT1	Q9Y617	phosphoserine aminotransferase 1
Malignant neoplasm of breast	FHOD1	Q9Y613	formin homology 2 domain containing 1
Malignant neoplasm of breast	PUS1	Q9Y606	pseudouridine synthase 1
Malignant neoplasm of breast	ETV7	Q9Y603	ETS variant transcription factor 7
Malignant neoplasm of breast	BACE2	Q9Y5Z0	beta-secretase 2
Malignant neoplasm of breast	LYVE1	Q9Y5Y7	lymphatic vessel endothelial hyaluronan receptor 1
Malignant neoplasm of breast	ST14	Q9Y5Y6	suppression of tumorigenicity 14

Malignant neoplasm of breast	GPR45	Q9Y5Y3	G protein-coupled receptor 45
Malignant neoplasm of breast	FLVCR1	Q9Y5Y0	FLVCR heme transporter 1
Malignant neoplasm of breast	LIPG	Q9Y5X9	lipase G, endothelial type
Malignant neoplasm of breast	NR2E3	Q9Y5X4	nuclear receptor subfamily 2 group E member 3
Malignant neoplasm of breast	SNX9	Q9Y5X1	sorting nexin 9
Malignant neoplasm of breast	WIF1	Q9Y5W5	WNT inhibitory factor 1
Malignant neoplasm of breast	KLF2	Q9Y5W3	Kruppel like factor 2
Malignant neoplasm of breast	MPC1	Q9Y5U8	mitochondrial pyruvate carrier 1
Malignant neoplasm of breast	INSIG2	Q9Y5U4	insulin induced gene 2
Malignant neoplasm of breast	DNAJC15	Q9Y5T4	DnaJ heat shock protein family (Hsp40) member C15
Malignant neoplasm of breast	CDC42BPB	Q9Y5S2	CDC42 binding protein kinase beta
Malignant neoplasm of breast	CERT1	Q9Y5P4	ceramide transporter 1
Malignant neoplasm of breast	RAI2	Q9Y5P3	retinoic acid induced 2
Malignant neoplasm of breast	CSAG2	Q9Y5P2	CSAG family member 2
Malignant neoplasm of breast	CSAG3	Q9Y5P2	CSAG family member 3
Malignant neoplasm of breast	HRH3	Q9Y5N1	histamine receptor H3
Malignant neoplasm of breast	FAM215A	Q9Y5M1	family with sequence similarity 215 member A
Malignant neoplasm of breast	PCYT1B	Q9Y5K3	phosphate cytidylyltransferase 1, choline, beta
Malignant neoplasm of breast	KLK4	Q9Y5K2	kallikrein related peptidase 4

Malignant neoplasm of breast	PHLDA3	Q9Y5J5	pleckstrin homology like domain family A member 3
Malignant neoplasm of breast	HEY1	Q9Y5J3	hes related family bHLH transcription factor with YRPW motif 1
Malignant neoplasm of breast	CLDN16	Q9Y5I7	claudin 16
Malignant neoplasm of breast	PCDHGB6	Q9Y5F9	protocadherin gamma subfamily B, 6
Malignant neoplasm of breast	PCDHGB7	Q9Y5F8	protocadherin gamma subfamily B, 7
Malignant neoplasm of breast	PCDHB15	Q9Y5E8	protocadherin beta 15
Malignant neoplasm of breast	RIPK3	Q9Y572	receptor interacting serine/threonine kinase 3
Malignant neoplasm of breast	NEUROG3	Q9Y4Z2	neurogenin 3
Malignant neoplasm of breast	CCL27	Q9Y4X3	C-C motif chemokine ligand 27
Malignant neoplasm of breast	RNF115	Q9Y4L5	ring finger protein 115
Malignant neoplasm of breast	HYOU1	Q9Y4L1	hypoxia up-regulated 1
Malignant neoplasm of breast	TRAF6	Q9Y4K3	TNF receptor associated factor 6
Malignant neoplasm of breast	LOXL2	Q9Y4K0	lysyl oxidase like 2
Malignant neoplasm of breast	MYO5A	Q9Y4I1	myosin VA
Malignant neoplasm of breast	IRS2	Q9Y4H2	insulin receptor substrate 2
Malignant neoplasm of breast	FARP1	Q9Y4F1	FERM, ARH/RhoGEF and pleckstrin domain protein 1
Malignant neoplasm of breast	DAAM1	Q9Y4D1	dishevelled associated activator of morphogenesis 1
Malignant neoplasm of breast	KDM3A	Q9Y4C1	lysine demethylase 3A
Malignant neoplasm of breast	DCAF1	Q9Y4B6	DDB1 and CUL4 associated factor 1

Malignant neoplasm of breast	NFE2L3	Q9Y4A8	nuclear factor, erythroid 2 like 3
Malignant neoplasm of breast	TLN1	Q9Y490	talin 1
Malignant neoplasm of breast	ATP6V0A2	Q9Y487	ATPase H+ transporting V0 subunit a2
Malignant neoplasm of breast	PRKAB1	Q9Y478	protein kinase AMP-activated non-catalytic subunit beta 1
Malignant neoplasm of breast	L3MBTL1	Q9Y468	L3MBTL histone methyl-lysine binding protein 1
Malignant neoplasm of breast	SALL2	Q9Y467	spalt like transcription factor 2
Malignant neoplasm of breast	NR2E1	Q9Y466	nuclear receptor subfamily 2 group E member 1
Malignant neoplasm of breast	DYRK1B	Q9Y463	dual specificity tyrosine phosphorylation regulated kinase 1B
Malignant neoplasm of breast	NOC2L	Q9Y3T9	NOC2 like nucleolar associated transcriptional repressor
Malignant neoplasm of breast	WNK2	Q9Y3S1	WNK lysine deficient protein kinase 2
Malignant neoplasm of breast	TSC22D4	Q9Y3Q8	TSC22 domain family member 4
Malignant neoplasm of breast	TMED3	Q9Y3Q3	transmembrane p24 trafficking protein 3
Malignant neoplasm of breast	STARD13	Q9Y3M8	StAR related lipid transfer domain containing 13
Malignant neoplasm of breast	RTCB	Q9Y3I0	RNA 2',3'-cyclic phosphate and 5'-OH ligase
Malignant neoplasm of breast	STRAP	Q9Y3F4	serine/threonine kinase receptor associated protein
Malignant neoplasm of breast	CHMP3	Q9Y3E7	charged multivesicular body protein 3
Malignant neoplasm of breast	PTRH2	Q9Y3E5	peptidyl-tRNA hydrolase 2
Malignant neoplasm of breast	MRPS23	Q9Y3D9	mitochondrial ribosomal protein S23
Malignant neoplasm of breast	AK6	Q9Y3D8	adenylate kinase 6

Malignant neoplasm of breast	MRPS18C	Q9Y3D5	mitochondrial ribosomal protein S18C
Malignant neoplasm of breast	UFC1	Q9Y3C8	ubiquitin-fold modifier conjugating enzyme 1
Malignant neoplasm of breast	RNF11	Q9Y3C5	ring finger protein 11
Malignant neoplasm of breast	NOP16	Q9Y3C1	NOP16 nucleolar protein
Malignant neoplasm of breast	SF3B6	Q9Y3B4	splicing factor 3b subunit 6
Malignant neoplasm of breast	TMED5	Q9Y3A6	transmembrane p24 trafficking protein 5
Malignant neoplasm of breast	SH3GLB1	Q9Y371	SH3 domain containing GRB2 like, endophilin B1
Malignant neoplasm of breast	STARD10	Q9Y365	StAR related lipid transfer domain containing 10
Malignant neoplasm of breast	SLC6A5	Q9Y345	solute carrier family 6 member 5
Malignant neoplasm of breast	KLK5	Q9Y337	kallikrein related peptidase 5
Malignant neoplasm of breast	LSM2	Q9Y333	LSM2 homolog, U6 small nuclear RNA and mRNA degradation associated
Malignant neoplasm of breast	TMX2	Q9Y320	thioredoxin related transmembrane protein 2
Malignant neoplasm of breast	MEMO1	Q9Y316	mediator of cell motility 1
Malignant neoplasm of breast	MTO1	Q9Y2Z2	mitochondrial tRNA translation optimization 1
Malignant neoplasm of breast	ZBTB32	Q9Y2Y4	zinc finger and BTB domain containing 32
Malignant neoplasm of breast	ZNF281	Q9Y2X9	zinc finger protein 281
Malignant neoplasm of breast	GIT1	Q9Y2X7	GIT ArfGAP 1
Malignant neoplasm of breast	KCNIP3	Q9Y2W7	potassium voltage-gated channel interacting protein 3
Malignant neoplasm of breast	MAP3K2	Q9Y2U5	mitogen-activated protein kinase kinase kinase 2

Malignant neoplasm of breast	GPR55	Q9Y2T6	G protein-coupled receptor 55
Malignant neoplasm of breast	AXIN2	Q9Y2T1	axin 2
Malignant neoplasm of breast	POLDIP2	Q9Y2S7	DNA polymerase delta interacting protein 2
Malignant neoplasm of breast	CRYL1	Q9Y2S2	crystallin lambda 1
Malignant neoplasm of breast	MRPS7	Q9Y2R9	mitochondrial ribosomal protein S7
Malignant neoplasm of breast	PTPN22	Q9Y2R2	protein tyrosine phosphatase non-receptor type 22
Malignant neoplasm of breast	MRPS28	Q9Y2Q9	mitochondrial ribosomal protein S28
Malignant neoplasm of breast	LAMTOR2	Q9Y2Q5	late endosomal/lysosomal adaptor, MAPK and MTOR activator 2
Malignant neoplasm of breast	GSTK1	Q9Y2Q3	glutathione S-transferase kappa 1
Malignant neoplasm of breast	SLC27A6	Q9Y2P4	solute carrier family 27 member 6
Malignant neoplasm of breast	FAN1	Q9Y2M0	FANCD2 and FANCI associated nuclease 1
Malignant neoplasm of breast	TRAPPC8	Q9Y2L5	trafficking protein particle complex 8
Malignant neoplasm of breast	KDM2A	Q9Y2K7	lysine demethylase 2A
Malignant neoplasm of breast	SIK3	Q9Y2K2	SIK family kinase 3
Malignant neoplasm of breast	PADI2	Q9Y2J8	peptidyl arginine deiminase 2
Malignant neoplasm of breast	EPB41L3	Q9Y2J2	erythrocyte membrane protein band 4.1 like 3
Malignant neoplasm of breast	PIKFYVE	Q9Y2I7	phosphoinositide kinase, FYVE-type zinc finger containing
Malignant neoplasm of breast	NINL	Q9Y2I6	ninein like
Malignant neoplasm of breast	NISCH	Q9Y2I1	nischarin

Malignant neoplasm of breast	INPP5F	Q9Y2H2	inositol polyphosphate-5-phosphatase F
Malignant neoplasm of breast	ANKRD6	Q9Y2G4	ankyrin repeat domain 6
Malignant neoplasm of breast	CARD8	Q9Y2G2	caspase recruitment domain family member 8
Malignant neoplasm of breast	DIP2C	Q9Y2E4	disco interacting protein 2 homolog C
Malignant neoplasm of breast	ZNF652	Q9Y2D9	zinc finger protein 652
Malignant neoplasm of breast	ATF5	Q9Y2D1	activating transcription factor 5
Malignant neoplasm of breast	TLR6	Q9Y2C9	toll like receptor 6
Malignant neoplasm of breast	NCKAP1	Q9Y2A7	NCK associated protein 1
Malignant neoplasm of breast	BTRC	Q9Y297	beta-transducin repeat containing E3 ubiquitin protein ligase
Malignant neoplasm of breast	DRG1	Q9Y295	developmentally regulated GTP binding protein 1
Malignant neoplasm of breast	ASF1A	Q9Y294	anti-silencing function 1A histone chaperone
Malignant neoplasm of breast	SLC5A6	Q9Y289	solute carrier family 5 member 6
Malignant neoplasm of breast	ITM2B	Q9Y287	integral membrane protein 2B
Malignant neoplasm of breast	SIGLEC7	Q9Y286	sialic acid binding Ig like lectin 7
Malignant neoplasm of breast	CFL2	Q9Y281	cofilin 2
Malignant neoplasm of breast	HS3ST2	Q9Y278	heparan sulfate-glucosamine 3-sulfotransferase 2
Malignant neoplasm of breast	BCS1L	Q9Y276	BCS1 homolog, ubiquinol-cytochrome c reductase complex chaperone
Malignant neoplasm of breast	TNFSF13B	Q9Y275	TNF superfamily member 13b
Malignant neoplasm of breast	RASD1	Q9Y272	ras related dexamethasone induced 1

Malignant neoplasm of breast	CYSLTR1	Q9Y271	cysteinyl leukotriene receptor 1
Malignant neoplasm of breast	RUVBL1	Q9Y265	RuvB like AAA ATPase 1
Malignant neoplasm of breast	FOXA2	Q9Y261	forkhead box A2
Malignant neoplasm of breast	RCE1	Q9Y256	Ras converting CAAX endopeptidase 1
Malignant neoplasm of breast	POLH	Q9Y253	DNA polymerase eta
Malignant neoplasm of breast	RNF6	Q9Y252	ring finger protein 6
Malignant neoplasm of breast	HPSE	Q9Y251	heparanase
Malignant neoplasm of breast	LZTS1	Q9Y250	leucine zipper tumor suppressor 1
Malignant neoplasm of breast	GINS2	Q9Y248	GINS complex subunit 2
Malignant neoplasm of breast	POMP	Q9Y244	proteasome maturation protein
Malignant neoplasm of breast	AKT3	Q9Y243	AKT serine/threonine kinase 3
Malignant neoplasm of breast	NOD1	Q9Y239	nucleotide binding oligomerization domain containing 1
Malignant neoplasm of breast	DLEC1	Q9Y238	DLEC1 cilia and flagella associated protein
Malignant neoplasm of breast	RUVBL2	Q9Y230	RuvB like AAA ATPase 2
Malignant neoplasm of breast	RTRAF	Q9Y224	RNA transcription, translation and transport factor
Malignant neoplasm of breast	NIP7	Q9Y221	nucleolar pre-rRNA processing protein NIP7
Malignant neoplasm of breast	JAG2	Q9Y219	jagged canonical Notch ligand 2
Malignant neoplasm of breast	TRPC6	Q9Y210	transient receptor potential cation channel subfamily C member 6
Malignant neoplasm of breast	LAMP3	Q9UQV4	lysosomal associated membrane protein 3

Malignant neoplasm of breast	SH2B3	Q9UQQ2	SH2B adaptor protein 3
Malignant neoplasm of breast	TNN	Q9UQP3	tenascin N
Malignant neoplasm of breast	HDAC5	Q9UQL6	histone deacetylase 5
Malignant neoplasm of breast	PPP1R3C	Q9UQK1	protein phosphatase 1 regulatory subunit 3C
Malignant neoplasm of breast	ERVW-1	Q9UQF0	endogenous retrovirus group W member 1, envelope
Malignant neoplasm of breast	CLCA2	Q9UQC9	chloride channel accessory 2
Malignant neoplasm of breast	GAB2	Q9UQC2	GRB2 associated binding protein 2
Malignant neoplasm of breast	AURKC	Q9UQB9	aurora kinase C
Malignant neoplasm of breast	CTNND2	Q9UQB3	catenin delta 2
Malignant neoplasm of breast	SPG7	Q9UQ90	SPG7 matrix AAA peptidase subunit, paraplegin
Malignant neoplasm of breast	EXO1	Q9UQ84	exonuclease 1
Malignant neoplasm of breast	PA2G4	Q9UQ80	proliferation-associated 2G4
Malignant neoplasm of breast	CNTN6	Q9UQ52	contactin 6
Malignant neoplasm of breast	SRRM2	Q9UQ35	serine/arginine repetitive matrix 2
Malignant neoplasm of breast	MOK	Q9UQ07	MOK protein kinase
Malignant neoplasm of breast	KCNH4	Q9UQ05	potassium voltage-gated channel subfamily H member 4
Malignant neoplasm of breast	CILK1	Q9UPZ9	ciliogenesis associated kinase 1
Malignant neoplasm of breast	THSD7A	Q9UPZ6	thrombospondin type 1 domain containing 7A
Malignant neoplasm of breast	HPS5	Q9UPZ3	HPS5 biogenesis of lysosomal organelles complex 2 subunit 2

Malignant neoplasm of breast	WASF3	Q9UPY6	WASP family member 3
Malignant neoplasm of breast	SLC7A11	Q9UPY5	solute carrier family 7 member 11
Malignant neoplasm of breast	DICER1	Q9UPY3	dicer 1, ribonuclease III
Malignant neoplasm of breast	MINAR1	Q9UPX6	membrane integral NOTCH2 associated receptor 1
Malignant neoplasm of breast	SATB2	Q9UPW6	SATB homeobox 2
Malignant neoplasm of breast	USP22	Q9UPT9	ubiquitin specific peptidase 22
Malignant neoplasm of breast	PHF8	Q9UPP1	PHD finger protein 8
Malignant neoplasm of breast	TRIM33	Q9UPN9	tripartite motif containing 33
Malignant neoplasm of breast	CEP131	Q9UPN4	centrosomal protein 131
Malignant neoplasm of breast	MACF1	Q9UPN3	microtubule actin crosslinking factor 1
Malignant neoplasm of breast	LHX6	Q9UPM6	LIM homeobox 6
Malignant neoplasm of breast	PLAGL2	Q9UPG8	PLAG1 like zinc finger 2
Malignant neoplasm of breast	ADAMTS8	Q9UP79	ADAM metallopeptidase with thrombospondin type 1 motif 8
Malignant neoplasm of breast	PLA2G4C	Q9UP65	phospholipase A2 group IVC
Malignant neoplasm of breast	FZD1	Q9UP38	frizzled class receptor 1
Malignant neoplasm of breast	WDR3	Q9UNX4	WD repeat domain 3
Malignant neoplasm of breast	NOVA2	Q9UNW9	NOVA alternative splicing regulator 2
Malignant neoplasm of breast	GPR132	Q9UNW8	G protein-coupled receptor 132
Malignant neoplasm of breast	TIMELESS	Q9UNS1	timeless circadian regulator

Malignant neoplasm of breast	ABCG2	Q9UNQ0	ATP binding cassette subfamily G member 2 (Junior blood group)
Malignant neoplasm of breast	PROCR	Q9UNN8	protein C receptor
Malignant neoplasm of breast	FAF1	Q9UNN5	Fas associated factor 1
Malignant neoplasm of breast	ING4	Q9UNL4	inhibitor of growth family member 4
Malignant neoplasm of breast	PLA2G2D	Q9UNK4	phospholipase A2 group IID
Malignant neoplasm of breast	CELA1	Q9UNI1	chymotrypsin like elastase 1
Malignant neoplasm of breast	CDC14A	Q9UNH5	cell division cycle 14A
Malignant neoplasm of breast	TNFSF18	Q9UNG2	TNF superfamily member 18
Malignant neoplasm of breast	MAGED2	Q9UNF1	MAGE family member D2
Malignant neoplasm of breast	STUB1	Q9UNE7	STIP1 homology and U-box containing protein 1
Malignant neoplasm of breast	RPH3AL	Q9UNE2	rabphilin 3A like (without C2 domains)
Malignant neoplasm of breast	EDAR	Q9UNE0	ectodysplasin A receptor
Malignant neoplasm of breast	POLI	Q9UNA4	DNA polymerase iota
Malignant neoplasm of breast	ARHGAP26	Q9UNA1	Rho GTPase activating protein 26
Malignant neoplasm of breast	ADAMTS5	Q9UNA0	ADAM metallopeptidase with thrombospondin type 1 motif 5
Malignant neoplasm of breast	G3BP2	Q9UN86	G3BP stress granule assembly factor 2
Malignant neoplasm of breast	SLC6A14	Q9UN76	solute carrier family 6 member 14
Malignant neoplasm of breast	NDRG2	Q9UN36	NDRG family member 2
Malignant neoplasm of breast	SLC45A2	Q9UMX9	solute carrier family 45 member 2

Malignant neoplasm of breast	BOK	Q9UMX3	BCL2 family apoptosis regulator BOK
Malignant neoplasm of breast	UBQLN1	Q9UMX0	ubiquilin 1
Malignant neoplasm of breast	USP18	Q9UMW8	ubiquitin specific peptidase 18
Malignant neoplasm of breast	SYNPO2	Q9UMS6	synaptopodin 2
Malignant neoplasm of breast	BARX2	Q9UMQ3	BARX homeobox 2
Malignant neoplasm of breast	KMT2B	Q9UMN6	lysine methyltransferase 2B
Malignant neoplasm of breast	ICAM5	Q9UMF0	intercellular adhesion molecule 5
Malignant neoplasm of breast	SPATA2	Q9UM82	spermatogenesis associated 2
Malignant neoplasm of breast	ALK	Q9UM73	ALK receptor tyrosine kinase
Malignant neoplasm of breast	PLAGL1	Q9UM63	PLAG1 like zinc finger 1
Malignant neoplasm of breast	MYO6	Q9UM54	myosin VI
Malignant neoplasm of breast	NOTCH3	Q9UM47	notch receptor 3
Malignant neoplasm of breast	HHLA2	Q9UM44	HERV-H LTR-associating 2
Malignant neoplasm of breast	FZR1	Q9UM11	fizzy and cell division cycle 20 related 1
Malignant neoplasm of breast	PADI4	Q9UM07	peptidyl arginine deiminase 4
Malignant neoplasm of breast	SLC7A7	Q9UM01	solute carrier family 7 member 7
Malignant neoplasm of breast	MMP17	Q9ULZ9	matrix metallopeptidase 17
Malignant neoplasm of breast	PYCARD	Q9ULZ3	PYD and CARD domain containing
Malignant neoplasm of breast	APLN	Q9ULZ1	apelin

Malignant neoplasm of breast	NOB1	Q9ULX3	NIN1 (RPN12) binding protein 1 homolog
Malignant neoplasm of breast	PADI3	Q9ULW8	peptidyl arginine deiminase 3
Malignant neoplasm of breast	TPX2	Q9ULW0	TPX2 microtubule nucleation factor
Malignant neoplasm of breast	CBLC	Q9ULV8	Cbl proto-oncogene C
Malignant neoplasm of breast	CORO1C	Q9ULV4	coronin 1C
Malignant neoplasm of breast	FZD4	Q9ULV1	frizzled class receptor 4
Malignant neoplasm of breast	ZMYND8	Q9ULU4	zinc finger MYND-type containing 8
Malignant neoplasm of breast	TBC1D24	Q9ULP9	TBC1 domain family member 24
Malignant neoplasm of breast	SHROOM4	Q9ULL8	shroom family member 4
Malignant neoplasm of breast	VANGL2	Q9ULK5	VANGL planar cell polarity protein 2
Malignant neoplasm of breast	MED23	Q9ULK4	mediator complex subunit 23
Malignant neoplasm of breast	GRID1	Q9ULK0	glutamate ionotropic receptor delta type subunit 1
Malignant neoplasm of breast	ZMIZ1	Q9ULJ6	zinc finger MIZ-type containing 1
Malignant neoplasm of breast	ASAP1	Q9ULH1	ArfGAP with SH3 domain, ankyrin repeat and PH domain 1
Malignant neoplasm of breast	KIDINS220	Q9ULH0	kinase D interacting substrate 220
Malignant neoplasm of breast	SLC39A10	Q9ULF5	solute carrier family 39 member 10
Malignant neoplasm of breast	PALD1	Q9ULE6	phosphatase domain containing paladin 1
Malignant neoplasm of breast	INTU	Q9ULD6	inturned planar cell polarity protein
Malignant neoplasm of breast	MTUS1	Q9ULD2	microtubule associated scaffold protein 1

Malignant neoplasm of breast	OGDHL	Q9ULD0	oxoglutarate dehydrogenase like
Malignant neoplasm of breast	PADI1	Q9ULC6	peptidyl arginine deiminase 1
Malignant neoplasm of breast	ACSL5	Q9ULC5	acyl-CoA synthetase long chain family member 5
Malignant neoplasm of breast	MCTS1	Q9ULC4	MCTS1 re-initiation and release factor
Malignant neoplasm of breast	RAB23	Q9ULC3	RAB23, member RAS oncogene family
Malignant neoplasm of breast	DNPEP	Q9ULA0	aspartyl aminopeptidase
Malignant neoplasm of breast	MYT1L	Q9UL68	myelin transcription factor 1 like
Malignant neoplasm of breast	TRPC5	Q9UL62	transient receptor potential cation channel subfamily C member 5
Malignant neoplasm of breast	ZNF236	Q9UL36	zinc finger protein 236
Malignant neoplasm of breast	RAB22A	Q9UL26	RAB22A, member RAS oncogene family
Malignant neoplasm of breast	RAB21	Q9UL25	RAB21, member RAS oncogene family
Malignant neoplasm of breast	PLAAT4	Q9UL19	phospholipase A and acyltransferase 4
Malignant neoplasm of breast	SARDH	Q9UL12	sarcosine dehydrogenase
Malignant neoplasm of breast	INTS6	Q9UL03	integrator complex subunit 6
Malignant neoplasm of breast	DSE	Q9UL01	dermatan sulfate epimerase
Malignant neoplasm of breast	TENM1	Q9UKZ4	teneurin transmembrane protein 1
Malignant neoplasm of breast	ZHX1	Q9UKY1	zinc fingers and homeoboxes 1
Malignant neoplasm of breast	ITGA11	Q9UKX5	integrin subunit alpha 11
Malignant neoplasm of breast	MYH2	Q9UKX2	myosin heavy chain 2

Malignant neoplasm of breast	ELF5	Q9UKW6	E74 like ETS transcription factor 5
Malignant neoplasm of breast	VAV3	Q9UKW4	vav guanine nucleotide exchange factor 3
Malignant neoplasm of breast	AGO2	Q9UKV8	argonaute RISC catalytic component 2
Malignant neoplasm of breast	AMFR	Q9UKV5	autocrine motility factor receptor
Malignant neoplasm of breast	HDAC9	Q9UKV0	histone deacetylase 9
Malignant neoplasm of breast	ANGPTL2	Q9UKU9	angiopoietin like 2
Malignant neoplasm of breast	ACAD8	Q9UKU7	acyl-CoA dehydrogenase family member 8
Malignant neoplasm of breast	IKZF3	Q9UKT9	IKAROS family zinc finger 3
Malignant neoplasm of breast	FBXO5	Q9UKT4	F-box protein 5
Malignant neoplasm of breast	IKZF2	Q9UKS7	IKAROS family zinc finger 2
Malignant neoplasm of breast	KLK13	Q9UKR3	kallikrein related peptidase 13
Malignant neoplasm of breast	KLK12	Q9UKR0	kallikrein related peptidase 12
Malignant neoplasm of breast	KLK9	Q9UKQ9	kallikrein related peptidase 9
Malignant neoplasm of breast	UTS2R	Q9UKP6	urotensin 2 receptor
Malignant neoplasm of breast	ADAMTS6	Q9UKP5	ADAM metallopeptidase with thrombospondin type 1 motif 6
Malignant neoplasm of breast	RALY	Q9UKM9	RALY heterogeneous nuclear ribonucleoprotein
Malignant neoplasm of breast	RCOR1	Q9UKL0	REST corepressor 1
Malignant neoplasm of breast	NUDT5	Q9UKK9	nudix hydrolase 5
Malignant neoplasm of breast	NXT1	Q9UKK6	nuclear transport factor 2 like export factor 1

Malignant neoplasm of breast	PARP4	Q9UKK3	poly(ADP-ribose) polymerase family member 4
Malignant neoplasm of breast	POU2F3	Q9UKI9	POU class 2 homeobox 3
Malignant neoplasm of breast	TLK1	Q9UKI8	tousled like kinase 1
Malignant neoplasm of breast	CDC42EP3	Q9UKI2	CDC42 effector protein 3
Malignant neoplasm of breast	APPL1	Q9UKG1	adaptor protein, phosphotyrosine interacting with PH domain and leucine zipper 1
Malignant neoplasm of breast	CPSF3	Q9UKF6	cleavage and polyadenylation specific factor 3
Malignant neoplasm of breast	ADAM29	Q9UKF5	ADAM metallopeptidase domain 29
Malignant neoplasm of breast	TNIK	Q9UKE5	TRAF2 and NCK interacting kinase
Malignant neoplasm of breast	AJAP1	Q9UKB5	adherens junctions associated protein 1
Malignant neoplasm of breast	DNAJC12	Q9UKB3	DnaJ heat shock protein family (Hsp40) member C12
Malignant neoplasm of breast	PTBP2	Q9UKA9	polypyrimidine tract binding protein 2
Malignant neoplasm of breast	RCAN3	Q9UKA8	RCAN family member 3
Malignant neoplasm of breast	FBXO10	Q9UK96	F-box protein 10
Malignant neoplasm of breast	JPT1	Q9UK76	Jupiter microtubule associated homolog 1
Malignant neoplasm of breast	DBR1	Q9UK59	debranching RNA lariats 1
Malignant neoplasm of breast	CCNL1	Q9UK58	cyclin L1
Malignant neoplasm of breast	ING1	Q9UK53	inhibitor of growth family member 1
Malignant neoplasm of breast	RPS6KA6	Q9UK32	ribosomal protein S6 kinase A6
Malignant neoplasm of breast	GDF2	Q9UK05	growth differentiation factor 2

Malignant neoplasm of breast	GGA1	Q9UJY5	golgi associated, gamma adaptin ear containing, ARF binding protein 1
Malignant neoplasm of breast	HSPB8	Q9UJY1	heat shock protein family B (small) member 8
Malignant neoplasm of breast	ANAPC4	Q9UJX5	anaphase promoting complex subunit 4
Malignant neoplasm of breast	ANAPC5	Q9UJX4	anaphase promoting complex subunit 5
Malignant neoplasm of breast	ANAPC7	Q9UJX3	anaphase promoting complex subunit 7
Malignant neoplasm of breast	OSGIN1	Q9UJX0	oxidative stress induced growth inhibitor 1
Malignant neoplasm of breast	DNMT3L	Q9UJW3	DNA methyltransferase 3 like
Malignant neoplasm of breast	DCTN4	Q9UJW0	dynactin subunit 4
Malignant neoplasm of breast	PURG	Q9UJV8	purine rich element binding protein G
Malignant neoplasm of breast	FOXD3	Q9UJU5	forkhead box D3
Malignant neoplasm of breast	LEF1	Q9UJU2	lymphoid enhancer binding factor 1
Malignant neoplasm of breast	FBXL7	Q9UJT9	F-box and leucine rich repeat protein 7
Malignant neoplasm of breast	SALL4	Q9UJQ4	spalt like transcription factor 4
Malignant neoplasm of breast	ERRFI1	Q9UJM3	ERBB receptor feedback inhibitor 1
Malignant neoplasm of breast	ZFP69B	Q9UJL9	ZFP69 zinc finger protein B
Malignant neoplasm of breast	MOSPD1	Q9UJG1	motile sperm domain containing 1
Malignant neoplasm of breast	RASAL2	Q9UJF2	RAS protein activator like 2
Malignant neoplasm of breast	HOOK1	Q9UJC3	hook microtubule tethering protein 1
Malignant neoplasm of breast	MCM8	Q9UJA3	minichromosome maintenance 8 homologous recombination repair factor

Malignant neoplasm of breast	CRLS1	Q9UJA2	cardiolipin synthase 1
Malignant neoplasm of breast	CDH22	Q9UJ99	cadherin 22
Malignant neoplasm of breast	MSRA	Q9UJ68	methionine sulfoxide reductase A
Malignant neoplasm of breast	RABGEF1	Q9UJ41	RAB guanine nucleotide exchange factor 1
Malignant neoplasm of breast	ST6GALNAC2	Q9UJ37	ST6 N-acetylgalactosaminide alpha-2,6-sialyltransferase 2
Malignant neoplasm of breast	PLXNA1	Q9UIW2	plexin A1
Malignant neoplasm of breast	CNOT7	Q9UIV1	CCR4-NOT transcription complex subunit 7
Malignant neoplasm of breast	SIX4	Q9UIU6	SIX homeobox 4
Malignant neoplasm of breast	MBD1	Q9UIS9	methyl-CpG binding domain protein 1
Malignant neoplasm of breast	LNPEP	Q9UIQ6	leucyl and cystinyl aminopeptidase
Malignant neoplasm of breast	FKBPL	Q9UIM3	FKBP prolyl isomerase like
Malignant neoplasm of breast	TMEFF2	Q9UIK5	transmembrane protein with EGF like and two follistatin like domains 2
Malignant neoplasm of breast	DAPK2	Q9UIK4	death associated protein kinase 2
Malignant neoplasm of breast	ZDHHC2	Q9UIJ5	zinc finger DHHC-type palmitoyltransferase 2
Malignant neoplasm of breast	KLF15	Q9UIH9	Kruppel like factor 15
Malignant neoplasm of breast	SLCO3A1	Q9UIG8	solute carrier organic anion transporter family member 3A1
Malignant neoplasm of breast	PSORS1C2	Q9UIG4	psoriasis susceptibility 1 candidate 2
Malignant neoplasm of breast	BAZ1B	Q9UIG0	bromodomain adjacent to zinc finger domain 1B
Malignant neoplasm of breast	MUTYH	Q9UIF7	mutY DNA glycosylase

Malignant neoplasm of breast	VPS51	Q9UID3	VPS51 subunit of GARP complex
Malignant neoplasm of breast	MAD2L2	Q9UI95	mitotic arrest deficient 2 like 2
Malignant neoplasm of breast	CPA4	Q9UI42	carboxypeptidase A4
Malignant neoplasm of breast	PRSS50	Q9UI38	serine protease 50
Malignant neoplasm of breast	DACH1	Q9UI36	dachshund family transcription factor 1
Malignant neoplasm of breast	GLS2	Q9UI32	glutaminase 2
Malignant neoplasm of breast	EIF2B4	Q9UI10	eukaryotic translation initiation factor 2B subunit delta
Malignant neoplasm of breast	EVL	Q9UI08	Enah/Vasp-like
Malignant neoplasm of breast	ADGRE2	Q9UHX3	adhesion G protein-coupled receptor E2
Malignant neoplasm of breast	PUF60	Q9UHX1	poly(U) binding splicing factor 60
Malignant neoplasm of breast	SLC12A6	Q9UHW9	solute carrier family 12 member 6
Malignant neoplasm of breast	GPN3	Q9UHW5	GPN-loop GTPase 3
Malignant neoplasm of breast	MED13	Q9UHV7	mediator complex subunit 13
Malignant neoplasm of breast	SERTAD1	Q9UHV2	SERTA domain containing 1
Malignant neoplasm of breast	ZNHIT2	Q9UHR6	zinc finger HIT-type containing 2
Malignant neoplasm of breast	SMPX	Q9UHP9	small muscle protein X-linked
Malignant neoplasm of breast	CEMIP2	Q9UHN6	cell migration inducing hyaluronidase 2
Malignant neoplasm of breast	AMACR	Q9UHK6	alpha-methylacyl-CoA racemase
Malignant neoplasm of breast	ADAMTS1	Q9UHI8	ADAM metallopeptidase with thrombospondin type 1 motif 1

Malignant neoplasm of breast	DDX20	Q9UHI6	DEAD-box helicase 20
Malignant neoplasm of breast	PCSK1N	Q9UHG2	proprotein convertase subtilisin/kexin type 1 inhibitor
Malignant neoplasm of breast	DCDC2	Q9UHG0	doublecortin domain containing 2
Malignant neoplasm of breast	TRPS1	Q9UHF7	transcriptional repressor GATA binding 1
Malignant neoplasm of breast	IL17B	Q9UHF5	interleukin 17B
Malignant neoplasm of breast	IL20RA	Q9UHF4	interleukin 20 receptor subunit alpha
Malignant neoplasm of breast	NAT8B	Q9UHF3	N-acetyltransferase 8B (putative, gene/pseudogene)
Malignant neoplasm of breast	EGFL7	Q9UHF1	EGF like domain multiple 7
Malignant neoplasm of breast	STEAP1	Q9UHE8	STEAP family member 1
Malignant neoplasm of breast	SEPTIN9	Q9UHD8	septin 9
Malignant neoplasm of breast	TBK1	Q9UHD2	TANK binding kinase 1
Malignant neoplasm of breast	CHORDC1	Q9UHD1	cysteine and histidine rich domain containing 1
Malignant neoplasm of breast	IL19	Q9UHD0	interleukin 19
Malignant neoplasm of breast	MLH3	Q9UHC1	mutL homolog 3
Malignant neoplasm of breast	LIMA1	Q9UHB6	LIM domain and actin binding 1
Malignant neoplasm of breast	LAMTOR3	Q9UHA4	late endosomal/lysosomal adaptor, MAPK and MTOR activator 3
Malignant neoplasm of breast	SUN2	Q9UH99	Sad1 and UNC84 domain containing 2
Malignant neoplasm of breast	EBF1	Q9UH73	EBF transcription factor 1
Malignant neoplasm of breast	APOBEC3B	Q9UH17	apolipoprotein B mRNA editing enzyme catalytic subunit 3B

Malignant neoplasm of breast	NDRG3	Q9UGV2	NDRG family member 3
Malignant neoplasm of breast	TCF20	Q9UGU0	transcription factor 20
Malignant neoplasm of breast	SUSD2	Q9UGT4	sushi domain containing 2
Malignant neoplasm of breast	POLL	Q9UGP5	DNA polymerase lambda
Malignant neoplasm of breast	LIMD1	Q9UGP4	LIM domains containing 1
Malignant neoplasm of breast	PARP2	Q9UGN5	poly(ADP-ribose) polymerase 2
Malignant neoplasm of breast	DMBT1	Q9UGM3	deleted in malignant brain tumors 1
Malignant neoplasm of breast	CHRNA9	Q9UGM1	cholinergic receptor nicotinic alpha 9 subunit
Malignant neoplasm of breast	KDM5B	Q9UGL1	lysine demethylase 5B
Malignant neoplasm of breast	STAP2	Q9UGK3	signal transducing adaptor family member 2
Malignant neoplasm of breast	PRKAG2	Q9UGJ0	protein kinase AMP-activated non-catalytic subunit gamma 2
Malignant neoplasm of breast	PRKAG3	Q9UGI9	protein kinase AMP-activated non-catalytic subunit gamma 3
Malignant neoplasm of breast	TES	Q9UGI8	testin LIM domain protein
Malignant neoplasm of breast	KCNN3	Q9UGI6	potassium calcium-activated channel subfamily N member 3
Malignant neoplasm of breast	ZRANB1	Q9UGI0	zinc finger RANBP2-type containing 1
Malignant neoplasm of breast	SLC23A2	Q9UGH3	solute carrier family 23 member 2
Malignant neoplasm of breast	OR12D3	Q9UGF7	olfactory receptor family 12 subfamily D member 3
Malignant neoplasm of breast	RGS17	Q9UGC6	regulator of G protein signaling 17
Malignant neoplasm of breast	CNOT8	Q9UFF9	CCR4-NOT transcription complex subunit 8

Malignant neoplasm of breast	FBXL17	Q9UF56	F-box and leucine rich repeat protein 17
Malignant neoplasm of breast	EPHA6	Q9UF33	EPH receptor A6
Malignant neoplasm of breast	PLEKHB1	Q9UF11	pleckstrin homology domain containing B1
Malignant neoplasm of breast	STK39	Q9UEW8	serine/threonine kinase 39
Malignant neoplasm of breast	ZNF629	Q9UEG4	zinc finger protein 629
Malignant neoplasm of breast	KL	Q9UEF7	klotho
Malignant neoplasm of breast	MALT1	Q9UDY8	MALT1 paracaspase
Malignant neoplasm of breast	DNAJB4	Q9UDY4	DnaJ heat shock protein family (Hsp40) member B4
Malignant neoplasm of breast	CLIP2	Q9UDT6	CAP-Gly domain containing linker protein 2
Malignant neoplasm of breast	PPP1R1B	Q9UD71	protein phosphatase 1 regulatory inhibitor subunit 1B
Malignant neoplasm of breast	REV1	Q9UBZ9	REV1 DNA directed polymerase
Malignant neoplasm of breast	LPAR3	Q9UBY5	lysophosphatidic acid receptor 3
Malignant neoplasm of breast	SLC9A2	Q9UBY0	solute carrier family 9 member A2
Malignant neoplasm of breast	KLK11	Q9UBX7	kallikrein related peptidase 11
Malignant neoplasm of breast	FBLN5	Q9UBX5	fibulin 5
Malignant neoplasm of breast	SEL1L	Q9UBV2	SEL1L adaptor subunit of ERAD E3 ubiquitin ligase
Malignant neoplasm of breast	NXF1	Q9UBU9	nuclear RNA export factor 1
Malignant neoplasm of breast	MORF4L1	Q9UBU8	mortality factor 4 like 1
Malignant neoplasm of breast	GHRL	Q9UBU3	ghrelin and obestatin prepropeptide

Malignant neoplasm of breast	DKK2	Q9UBU2	dickkopf WNT signaling pathway inhibitor 2
Malignant neoplasm of breast	POLK	Q9UBT6	DNA polymerase kappa
Malignant neoplasm of breast	DKK4	Q9UBT3	dickkopf WNT signaling pathway inhibitor 4
Malignant neoplasm of breast	UBA2	Q9UBT2	ubiquitin like modifier activating enzyme 2
Malignant neoplasm of breast	RPS6KB2	Q9UBS0	ribosomal protein S6 kinase B2
Malignant neoplasm of breast	CTSZ	Q9UBR2	cathepsin Z
Malignant neoplasm of breast	HEY2	Q9UBP5	hes related family bHLH transcription factor with YRPW motif 2
Malignant neoplasm of breast	DKK3	Q9UBP4	dickkopf WNT signaling pathway inhibitor 3
Malignant neoplasm of breast	SPAST	Q9UBP0	spastin
Malignant neoplasm of breast	HDAC6	Q9UBN7	histone deacetylase 6
Malignant neoplasm of breast	TNFRSF10D	Q9UBN6	TNF receptor superfamily member 10d
Malignant neoplasm of breast	TRPC4	Q9UBN4	transient receptor potential cation channel subfamily C member 4
Malignant neoplasm of breast	PEMT	Q9UBM1	phosphatidylethanolamine N-methyltransferase
Malignant neoplasm of breast	CPNE7	Q9UBL6	copine 7
Malignant neoplasm of breast	ASH2L	Q9UBL3	ASH2 like, histone lysine methyltransferase complex subunit
Malignant neoplasm of breast	ARPP21	Q9UBL0	cAMP regulated phosphoprotein 21
Malignant neoplasm of breast	MTRR	Q9UBK8	5-methyltetrahydrofolate-homocysteine methyltransferase reductase
Malignant neoplasm of breast	PPARGC1A	Q9UBK2	PPARG coactivator 1 alpha
Malignant neoplasm of breast	ABCD2	Q9UBJ2	ATP binding cassette subfamily D member 2

Malignant neoplasm of breast	STOML1	Q9UBI4	stomatin like 1
Malignant neoplasm of breast	XPR1	Q9UBH6	xenotropic and polytropic retrovirus receptor 1
Malignant neoplasm of breast	MRC2	Q9UBG0	mannose receptor C type 2
Malignant neoplasm of breast	MAGEC2	Q9UBF1	MAGE family member C2
Malignant neoplasm of breast	NLK	Q9UBE8	nemo like kinase
Malignant neoplasm of breast	SAE1	Q9UBE0	SUMO1 activating enzyme subunit 1
Malignant neoplasm of breast	DNMT3B	Q9UBC3	DNA methyltransferase 3 beta
Malignant neoplasm of breast	MBD2	Q9UBB5	methyl-CpG binding domain protein 2
Malignant neoplasm of breast	THAP10	Q9P2Z0	THAP domain containing 10
Malignant neoplasm of breast	UVRAG	Q9P2Y5	UV radiation resistance associated
Malignant neoplasm of breast	DELEC1	Q9P2X7	deleted in esophageal cancer 1
Malignant neoplasm of breast	IMPACT	Q9P2X3	impact RWD domain protein
Malignant neoplasm of breast	STX18	Q9P2W9	syntaxin 18
Malignant neoplasm of breast	B3GAT1	Q9P2W7	beta-1,3-glucuronyltransferase 1
Malignant neoplasm of breast	PSMC3IP	Q9P2W1	PSMC3 interacting protein
Malignant neoplasm of breast	SLC17A6	Q9P2U8	solute carrier family 17 member 6
Malignant neoplasm of breast	GMPR2	Q9P2T1	guanosine monophosphate reductase 2
Malignant neoplasm of breast	ADAMTS9	Q9P2N4	ADAM metallopeptidase with thrombospondin type 1 motif 9
Malignant neoplasm of breast	PCDH10	Q9P2E7	protocadherin 10

Malignant neoplasm of breast	KIF17	Q9P2E2	kinesin family member 17
Malignant neoplasm of breast	CHD7	Q9P2D1	chromodomain helicase DNA binding protein 7
Malignant neoplasm of breast	CFAP97	Q9P2B7	cilia and flagella associated protein 97
Malignant neoplasm of breast	ARMCX1	Q9P291	armadillo repeat containing X-linked 1
Malignant neoplasm of breast	BCCIP	Q9P287	BRCA2 and CDKN1A interacting protein
Malignant neoplasm of breast	PAK5	Q9P286	p21 (RAC1) activated kinase 5
Malignant neoplasm of breast	SEMA5B	Q9P283	semaphorin 5B
Malignant neoplasm of breast	TENM3	Q9P273	teneurin transmembrane protein 3
Malignant neoplasm of breast	RCC2	Q9P258	regulator of chromosome condensation 2
Malignant neoplasm of breast	STIM2	Q9P246	stromal interaction molecule 2
Malignant neoplasm of breast	CNTN3	Q9P232	contactin 3
Malignant neoplasm of breast	CCDC88C	Q9P219	coiled-coil domain containing 88C
Malignant neoplasm of breast	ZSWIM5	Q9P217	zinc finger SWIM-type containing 5
Malignant neoplasm of breast	KIAA1522	Q9P206	KIAA1522
Malignant neoplasm of breast	ZBTB4	Q9P1Z0	zinc finger and BTB domain containing 4
Malignant neoplasm of breast	PHRF1	Q9P1Y6	PHD and ring finger domains 1
Malignant neoplasm of breast	PIM2	Q9P1W9	Pim-2 proto-oncogene, serine/threonine kinase
Malignant neoplasm of breast	DOK5	Q9P104	docking protein 5
Malignant neoplasm of breast	PIPOX	Q9P0Z9	pipecolic acid and sarcosine oxidase

Malignant neoplasm of breast	CACNA1I	Q9P0X4	calcium voltage-gated channel subunit alpha1 I
Malignant neoplasm of breast	SH3BP4	Q9P0V3	SH3 domain binding protein 4
Malignant neoplasm of breast	SENP1	Q9P0U3	SUMO specific peptidase 1
Malignant neoplasm of breast	TMEM14C	Q9P0S9	transmembrane protein 14C
Malignant neoplasm of breast	PKD2L1	Q9P0L9	polycystin 2 like 1, transient receptor potential cation channel
Malignant neoplasm of breast	FOXJ2	Q9P0K8	forkhead box J2
Malignant neoplasm of breast	RAI14	Q9P0K7	retinoic acid induced 14
Malignant neoplasm of breast	ADAM22	Q9P0K1	ADAM metallopeptidase domain 22
Malignant neoplasm of breast	MRPL36	Q9P0J6	mitochondrial ribosomal protein L36
Malignant neoplasm of breast	NDUFA13	Q9P0J0	NADH:ubiquinone oxidoreductase subunit A13
Malignant neoplasm of breast	KLK14	Q9P0G3	kallikrein related peptidase 14
Malignant neoplasm of breast	NDUFAF4	Q9P032	NADH:ubiquinone oxidoreductase complex assembly factor 4
Malignant neoplasm of breast	DSPP	Q9NZW4	dentin sialophosphoprotein
Malignant neoplasm of breast	SELENON	Q9NZV5	selenoprotein N
Malignant neoplasm of breast	VSX1	Q9NZR4	visual system homeobox 1
Malignant neoplasm of breast	LRP1B	Q9NZR2	LDL receptor related protein 1B
Malignant neoplasm of breast	TMOD4	Q9NZQ9	tropomodulin 4
Malignant neoplasm of breast	TRPM5	Q9NZQ8	transient receptor potential cation channel subfamily M member 5
Malignant neoplasm of breast	CD274	Q9NZQ7	CD274 molecule

Malignant neoplasm of breast	NCKIPSD	Q9NZQ3	NCK interacting protein with SH3 domain
Malignant neoplasm of breast	DNAJC27	Q9NZQ0	DnaJ heat shock protein family (Hsp40) member C27
Malignant neoplasm of breast	ARHGEF12	Q9NZN5	Rho guanine nucleotide exchange factor 12
Malignant neoplasm of breast	EHD2	Q9NZN4	EH domain containing 2
Malignant neoplasm of breast	NOP53	Q9NZM5	NOP53 ribosome biogenesis factor
Malignant neoplasm of breast	ITSN2	Q9NZM3	intersectin 2
Malignant neoplasm of breast	MYOF	Q9NZM1	myoferlin
Malignant neoplasm of breast	RGL1	Q9NZL6	ral guanine nucleotide dissociation stimulator like 1
Malignant neoplasm of breast	ZNF224	Q9NZL3	zinc finger protein 224
Malignant neoplasm of breast	EIF2AK3	Q9NZJ5	eukaryotic translation initiation factor 2 alpha kinase 3
Malignant neoplasm of breast	DTL	Q9NZJ0	denticleless E3 ubiquitin protein ligase homolog
Malignant neoplasm of breast	IGF2BP1	Q9NZI8	insulin like growth factor 2 mRNA binding protein 1
Malignant neoplasm of breast	PTTG3P	Q9NZH4	pituitary tumor-transforming 3, pseudogene
Malignant neoplasm of breast	PLAC8	Q9NZF1	placenta associated 8
Malignant neoplasm of breast	SPG21	Q9NZD8	SPG21 abhydrolase domain containing, maspardin
Malignant neoplasm of breast	SMARCAL1	Q9NZC9	SWI/SNF related, matrix associated, actin dependent regulator of chromatin, subfamily a like 1
Malignant neoplasm of breast	WWOX	Q9NZC7	WW domain containing oxidoreductase
Malignant neoplasm of breast	EHF	Q9NZC4	ETS homologous factor
Malignant neoplasm of breast	GDE1	Q9NZC3	glycerophosphodiester phosphodiesterase 1

Malignant neoplasm of breast	RTEL1	Q9NZ71	regulator of telomere elongation helicase 1
Malignant neoplasm of breast	GGA3	Q9NZ52	golgi associated, gamma adaptin ear containing, ARF binding protein 3
Malignant neoplasm of breast	CISD1	Q9NZ45	CDGSH iron sulfur domain 1
Malignant neoplasm of breast	USE1	Q9NZ43	unconventional SNARE in the ER 1
Malignant neoplasm of breast	GTSE1	Q9NYZ3	G2 and S-phase expressed 1
Malignant neoplasm of breast	SLC25A37	Q9NYZ2	solute carrier family 25 member 37
Malignant neoplasm of breast	FASTKD2	Q9NYY8	FAST kinase domains 2
Malignant neoplasm of breast	PLK2	Q9NYY3	polo like kinase 2
Malignant neoplasm of breast	IL20	Q9NYY1	interleukin 20
Malignant neoplasm of breast	TAS2R13	Q9NYV9	taste 2 receptor member 13
Malignant neoplasm of breast	RRN3	Q9NYV6	RRN3 homolog, RNA polymerase I transcription factor
Malignant neoplasm of breast	CDK12	Q9NYV4	cyclin dependent kinase 12
Malignant neoplasm of breast	MAP3K20	Q9NYL2	mitogen-activated protein kinase kinase kinase 20
Malignant neoplasm of breast	TLR7	Q9NYK1	toll like receptor 7
Malignant neoplasm of breast	TAB2	Q9NYJ8	TGF-beta activated kinase 1 (MAP3K7) binding protein 2
Malignant neoplasm of breast	DLL3	Q9NYJ7	delta like canonical Notch ligand 3
Malignant neoplasm of breast	ANAPC11	Q9NYG5	anaphase promoting complex subunit 11
Malignant neoplasm of breast	ZDHHC3	Q9NYG2	zinc finger DHHC-type palmitoyltransferase 3
Malignant neoplasm of breast	DACT1	Q9NYF0	dishevelled binding antagonist of beta catenin 1

Malignant neoplasm of breast	HOXC10	Q9NYD6	homeobox C10
Malignant neoplasm of breast	DNAH9	Q9NYC9	dynein axonemal heavy chain 9
Malignant neoplasm of breast	ABI2	Q9NYB9	abl interactor 2
Malignant neoplasm of breast	TERF2IP	Q9NYB0	TERF2 interacting protein
Malignant neoplasm of breast	SPHK1	Q9NYA1	sphingosine kinase 1
Malignant neoplasm of breast	DDX56	Q9NY93	DEAD-box helicase 56
Malignant neoplasm of breast	AATF	Q9NY61	apoptosis antagonizing transcription factor
Malignant neoplasm of breast	SMPD3	Q9NY59	sphingomyelin phosphodiesterase 3
Malignant neoplasm of breast	OBP2A	Q9NY56	odorant binding protein 2A
Malignant neoplasm of breast	CACNA2D2	Q9NY47	calcium voltage-gated channel auxiliary subunit alpha2delta 2
Malignant neoplasm of breast	SCN3A	Q9NY46	sodium voltage-gated channel alpha subunit 3
Malignant neoplasm of breast	CLDND1	Q9NY35	claudin domain containing 1
Malignant neoplasm of breast	DPP3	Q9NY33	dipeptidyl peptidase 3
Malignant neoplasm of breast	BTG4	Q9NY30	BTG anti-proliferation factor 4
Malignant neoplasm of breast	STAB1	Q9NY15	stabilin 1
Malignant neoplasm of breast	SAGE1	Q9NXZ1	sarcoma antigen 1
Malignant neoplasm of breast	BABAM2	Q9NXR7	BRISC and BRCA1 A complex member 2
Malignant neoplasm of breast	ARHGEF38	Q9NXL2	Rho guanine nucleotide exchange factor 38
Malignant neoplasm of breast	PGPEP1	Q9NXJ5	pyroglutamyl-peptidase I

Malignant neoplasm of breast	THUMPD1	Q9NXG2	THUMP domain containing 1
Malignant neoplasm of breast	SIRT5	Q9NXA8	sirtuin 5
Malignant neoplasm of breast	SYBU	Q9NX95	syntabulin
Malignant neoplasm of breast	TMEM161A	Q9NX61	transmembrane protein 161A
Malignant neoplasm of breast	MARCHF5	Q9NX47	membrane associated ring-CH-type finger 5
Malignant neoplasm of breast	ADPRS	Q9NX46	ADP-ribosylserine hydrolase
Malignant neoplasm of breast	SOHLH2	Q9NX45	spermatogenesis and oogenesis specific basic helix-loop-helix 2
Malignant neoplasm of breast	SDHAF2	Q9NX18	succinate dehydrogenase complex assembly factor 2
Malignant neoplasm of breast	DDIT4	Q9NX09	DNA damage inducible transcript 4
Malignant neoplasm of breast	C1orf109	Q9NX04	chromosome 1 open reading frame 109
Malignant neoplasm of breast	NLRP2	Q9NX02	NLR family pyrin domain containing 2
Malignant neoplasm of breast	ASB6	Q9NWX5	ankyrin repeat and SOCS box containing 6
Malignant neoplasm of breast	BABAM1	Q9NWV8	BRISC and BRCA1 A complex member 1
Malignant neoplasm of breast	OXSM	Q9NWU1	3-oxoacyl-ACP synthase, mitochondrial
Malignant neoplasm of breast	HIF1AN	Q9NWT6	hypoxia inducible factor 1 subunit alpha inhibitor
Malignant neoplasm of breast	RMND1	Q9NWS8	required for meiotic nuclear division 1 homolog
Malignant neoplasm of breast	PIH1D1	Q9NWS0	PIH1 domain containing 1
Malignant neoplasm of breast	PAG1	Q9NWQ8	phosphoprotein membrane anchor with glycosphingolipid microdomains 1
Malignant neoplasm of breast	CUEDC1	Q9NWM3	CUE domain containing 1

Malignant neoplasm of breast	SMOX	Q9NWM0	spermine oxidase
Malignant neoplasm of breast	SLC52A1	Q9NWF4	solute carrier family 52 member 1
Malignant neoplasm of breast	TMEM45A	Q9NWC5	transmembrane protein 45A
Malignant neoplasm of breast	ARGLU1	Q9NWB6	arginine and glutamate rich 1
Malignant neoplasm of breast	GPATCH2	Q9NW75	G-patch domain containing 2
Malignant neoplasm of breast	FANCL	Q9NW38	FA complementation group L
Malignant neoplasm of breast	NLE1	Q9NVX2	notchless homolog 1
Malignant neoplasm of breast	PNPO	Q9NVS9	pyridoxamine 5'-phosphate oxidase
Malignant neoplasm of breast	MRPS18A	Q9NVS2	mitochondrial ribosomal protein S18A
Malignant neoplasm of breast	ASF1B	Q9NVP2	anti-silencing function 1B histone chaperone
Malignant neoplasm of breast	DDX18	Q9NVP1	DEAD-box helicase 18
Malignant neoplasm of breast	PRMT7	Q9NVM4	protein arginine methyltransferase 7
Malignant neoplasm of breast	ATAD3A	Q9NVI7	ATPase family AAA domain containing 3A
Malignant neoplasm of breast	FANCI	Q9NVI1	FA complementation group I
Malignant neoplasm of breast	FBXO28	Q9NVF7	F-box protein 28
Malignant neoplasm of breast	PARVA	Q9NVD7	parvin alpha
Malignant neoplasm of breast	SETD4	Q9NVD3	SET domain containing 4
Malignant neoplasm of breast	UQCC1	Q9NVA1	ubiquinol-cytochrome c reductase complex assembly factor 1
Malignant neoplasm of breast	TMEM39A	Q9NV64	transmembrane protein 39A

Malignant neoplasm of breast	RNF19A	Q9NV58	ring finger protein 19A, RBR E3 ubiquitin protein ligase
Malignant neoplasm of breast	IMP3	Q9NV31	IMP U3 small nucleolar ribonucleoprotein 3
Malignant neoplasm of breast	POT1	Q9NUX5	protection of telomeres 1
Malignant neoplasm of breast	TDP1	Q9NUW8	tyrosyl-DNA phosphodiesterase 1
Malignant neoplasm of breast	ABCB8	Q9NUT2	ATP binding cassette subfamily B member 8
Malignant neoplasm of breast	SLC39A9	Q9NUM3	solute carrier family 39 member 9
Malignant neoplasm of breast	ZFP57	Q9NU63	ZFP57 zinc finger protein
Malignant neoplasm of breast	ANKEF1	Q9NU02	ankyrin repeat and EF-hand domain containing 1
Malignant neoplasm of breast	FAM217B	Q9NTX9	family with sequence similarity 217 member B
Malignant neoplasm of breast	RNF146	Q9NTX7	ring finger protein 146
Malignant neoplasm of breast	ECHDC1	Q9NTX5	ethylmalonyl-CoA decarboxylase 1
Malignant neoplasm of breast	ZFP64	Q9NTW7	ZFP64 zinc finger protein
Malignant neoplasm of breast	CUTC	Q9NTM9	cutC copper transporter
Malignant neoplasm of breast	OLA1	Q9NTK5	Obg like ATPase 1
Malignant neoplasm of breast	DEPP1	Q9NTK1	DEPP1 autophagy regulator
Malignant neoplasm of breast	SACM1L	Q9NTJ5	SAC1 like phosphatidylinositide phosphatase
Malignant neoplasm of breast	MAN2C1	Q9NTJ4	mannosidase alpha class 2C member 1
Malignant neoplasm of breast	SMC4	Q9NTJ3	structural maintenance of chromosomes 4
Malignant neoplasm of breast	PDS5B	Q9NTI5	PDS5 cohesin associated factor B

Malignant neoplasm of breast	SIRT3	Q9NTG7	sirtuin 3
Malignant neoplasm of breast	PKDREJ	Q9NTG1	polycystin family receptor for egg jelly
Malignant neoplasm of breast	TENM2	Q9NT68	teneurin transmembrane protein 2
Malignant neoplasm of breast	ATG3	Q9NT62	autophagy related 3
Malignant neoplasm of breast	DIAPH3	Q9NSV4	diaphanous related formin 3
Malignant neoplasm of breast	TREX1	Q9NSU2	three prime repair exonuclease 1
Malignant neoplasm of breast	CISH	Q9NSE2	cytokine inducible SH2 containing protein
Malignant neoplasm of breast	ST6GALNAC1	Q9NSC7	ST6 N-acetylgalactosaminide alpha-2,6-sialyltransferase 1
Malignant neoplasm of breast	SALL1	Q9NSC2	spalt like transcription factor 1
Malignant neoplasm of breast	CTNNBIP1	Q9NSA3	catenin beta interacting protein 1
Malignant neoplasm of breast	FGF21	Q9NSA1	fibroblast growth factor 21
Malignant neoplasm of breast	KIF15	Q9NS87	kinesin family member 15
Malignant neoplasm of breast	CA10	Q9NS85	carbonic anhydrase 10
Malignant neoplasm of breast	SLC7A10	Q9NS82	solute carrier family 7 member 10
Malignant neoplasm of breast	CYSLTR2	Q9NS75	cysteinyl leukotriene receptor 2
Malignant neoplasm of breast	GKN1	Q9NS71	gastrokine 1
Malignant neoplasm of breast	KCNH7	Q9NS40	potassium voltage-gated channel subfamily H member 7
Malignant neoplasm of breast	CREBZF	Q9NS37	CREB/ATF bZIP transcription factor
Malignant neoplasm of breast	SPANXB1	Q9NS25	SPANX family member B1

Malignant neoplasm of breast	RASSF1	Q9NS23	Ras association domain family member 1
Malignant neoplasm of breast	LTBP3	Q9NS15	latent transforming growth factor beta binding protein 3
Malignant neoplasm of breast	C1GALT1	Q9NS00	core 1 synthase, glycoprotein-N-acetylgalactosamine 3-beta-galactosyltransferase 1
Malignant neoplasm of breast	HELLS	Q9NRZ9	helicase, lymphoid specific
Malignant neoplasm of breast	PHPT1	Q9NRX4	phosphohistidine phosphatase 1
Malignant neoplasm of breast	PNO1	Q9NRX1	partner of NOB1 homolog
Malignant neoplasm of breast	DUSP22	Q9NRW4	dual specificity phosphatase 22
Malignant neoplasm of breast	RAB6B	Q9NRW1	RAB6B, member RAS oncogene family
Malignant neoplasm of breast	HEBP1	Q9NRV9	heme binding protein 1
Malignant neoplasm of breast	TMPRSS4	Q9NRS4	transmembrane serine protease 4
Malignant neoplasm of breast	DROSHA	Q9NRR4	drosha ribonuclease III
Malignant neoplasm of breast	CMC2	Q9NRP2	C-X9-C motif containing 2
Malignant neoplasm of breast	OSTC	Q9NRP0	oligosaccharyltransferase complex non-catalytic subunit
Malignant neoplasm of breast	LATS2	Q9NRM7	large tumor suppressor kinase 2
Malignant neoplasm of breast	IL17RB	Q9NRM6	interleukin 17 receptor B
Malignant neoplasm of breast	ZNF277	Q9NRM2	zinc finger protein 277
Malignant neoplasm of breast	ABCB10	Q9NRK6	ATP binding cassette subfamily B member 10
Malignant neoplasm of breast	CCL28	Q9NRJ3	C-C motif chemokine ligand 28
Malignant neoplasm of breast	C8orf17	Q9NRJ1	chromosome 8 open reading frame 17

Malignant neoplasm of breast	SMYD2	Q9NRG4	SET and MYND domain containing 2
Malignant neoplasm of breast	CHRAC1	Q9NRG0	chromatin accessibility complex subunit 1
Malignant neoplasm of breast	TSHZ2	Q9NRE2	teashirt zinc finger homeobox 2
Malignant neoplasm of breast	MMP26	Q9NRE1	matrix metallopeptidase 26
Malignant neoplasm of breast	DUOX1	Q9NRD9	dual oxidase 1
Malignant neoplasm of breast	FBXO8	Q9NRD0	F-box protein 8
Malignant neoplasm of breast	SIRT7	Q9NRC8	sirtuin 7
Malignant neoplasm of breast	ST7	Q9NRC1	suppression of tumorigenicity 7
Malignant neoplasm of breast	PDGFC	Q9NRA1	platelet derived growth factor C
Malignant neoplasm of breast	SPHK2	Q9NRA0	sphingosine kinase 2
Malignant neoplasm of breast	TLR8	Q9NR97	toll like receptor 8
Malignant neoplasm of breast	TLR9	Q9NR96	toll like receptor 9
Malignant neoplasm of breast	SLC2A4RG	Q9NR83	SLC2A4 regulator
Malignant neoplasm of breast	ARHGEF4	Q9NR80	Rho guanine nucleotide exchange factor 4
Malignant neoplasm of breast	KLHL1	Q9NR64	kelch like family member 1
Malignant neoplasm of breast	DLL4	Q9NR61	delta like canonical Notch ligand 4
Malignant neoplasm of breast	BATF3	Q9NR55	basic leucine zipper ATF-like transcription factor 3
Malignant neoplasm of breast	NANS	Q9NR45	N-acetylneuraminate synthase
Malignant neoplasm of breast	DDX21	Q9NR30	DExD-box helicase 21

Malignant neoplasm of breast	DIABLO	Q9NR28	diablo IAP-binding mitochondrial protein
Malignant neoplasm of breast	GDF3	Q9NR23	growth differentiation factor 3
Malignant neoplasm of breast	ACSS2	Q9NR19	acyl-CoA synthetase short chain family member 2
Malignant neoplasm of breast	PDLIM7	Q9NR12	PDZ and LIM domain 7
Malignant neoplasm of breast	BIRC6	Q9NR09	baculoviral IAP repeat containing 6
Malignant neoplasm of breast	TCIM	Q9NR00	transcriptional and immune response regulator
Malignant neoplasm of breast	DAZ1	Q9NQZ3	deleted in azoospermia 1
Malignant neoplasm of breast	ZNF331	Q9NQX6	zinc finger protein 331
Malignant neoplasm of breast	GPHN	Q9NQX3	gephyrin
Malignant neoplasm of breast	ANLN	Q9NQW6	anillin actin binding protein
Malignant neoplasm of breast	PAK6	Q9NQU5	p21 (RAC1) activated kinase 6
Malignant neoplasm of breast	INCENP	Q9NQS7	inner centromere protein
Malignant neoplasm of breast	NECTIN3	Q9NQS3	nectin cell adhesion molecule 3
Malignant neoplasm of breast	G6PC2	Q9NQR9	glucose-6-phosphatase catalytic subunit 2
Malignant neoplasm of breast	KMT5A	Q9NQR1	lysine methyltransferase 5A
Malignant neoplasm of breast	PFDN4	Q9NQP4	prefoldin subunit 4
Malignant neoplasm of breast	RPRD1B	Q9NQG5	regulation of nuclear pre-mRNA domain containing 1B
Malignant neoplasm of breast	CYLD	Q9NQC7	CYLD lysine 63 deubiquitinase
Malignant neoplasm of breast	RTN4	Q9NQC3	reticulon 4

Malignant neoplasm of breast	TCF7L2	Q9NQB0	transcription factor 7 like 2
Malignant neoplasm of breast	A1CF	Q9NQ94	APOBEC1 complementation factor
Malignant neoplasm of breast	HEYL	Q9NQ87	hes related family bHLH transcription factor with YRPW motif like
Malignant neoplasm of breast	MEPE	Q9NQ76	matrix extracellular phosphoglycoprotein
Malignant neoplasm of breast	PLCB1	Q9NQ66	phospholipase C beta 1
Malignant neoplasm of breast	LZTFL1	Q9NQ48	leucine zipper transcription factor like 1
Malignant neoplasm of breast	SCUBE2	Q9NQ36	signal peptide, CUB domain and EGF like domain containing 2
Malignant neoplasm of breast	AKIP1	Q9NQ31	A-kinase interacting protein 1
Malignant neoplasm of breast	RIC8A	Q9NPQ8	RIC8 guanine nucleotide exchange factor A
Malignant neoplasm of breast	FANCF	Q9NPI8	FA complementation group F
Malignant neoplasm of breast	BRD7	Q9NPI1	bromodomain containing 7
Malignant neoplasm of breast	NOX4	Q9NPH5	NADPH oxidase 4
Malignant neoplasm of breast	ISYNA1	Q9NPH2	inositol-3-phosphate synthase 1
Malignant neoplasm of breast	ZDHHC4	Q9NPG8	zinc finger DHHC-type palmitoyltransferase 4
Malignant neoplasm of breast	NGB	Q9NPG2	neuroglobin
Malignant neoplasm of breast	FZD3	Q9NPG1	frizzled class receptor 3
Malignant neoplasm of breast	IL23A	Q9NPF7	interleukin 23 subunit alpha
Malignant neoplasm of breast	CHST11	Q9NPF2	carbohydrate sulfotransferase 11
Malignant neoplasm of breast	CD320	Q9NPF0	CD320 molecule

Malignant neoplasm of breast	UBE2T	Q9NPD8	ubiquitin conjugating enzyme E2 T
Malignant neoplasm of breast	SLCO1B3	Q9NPD5	solute carrier organic anion transporter family member 1B3
Malignant neoplasm of breast	SIX2	Q9NPC8	SIX homeobox 2
Malignant neoplasm of breast	KCNK9	Q9NPC2	potassium two pore domain channel subfamily K member 9
Malignant neoplasm of breast	LTB4R2	Q9NPC1	leukotriene B4 receptor 2
Malignant neoplasm of breast	ACKR4	Q9NPB9	atypical chemokine receptor 4
Malignant neoplasm of breast	ENY2	Q9NPA8	ENY2 transcription and export complex 2 subunit
Malignant neoplasm of breast	TREM1	Q9NP99	triggering receptor expressed on myeloid cells 1
Malignant neoplasm of breast	SLC39A2	Q9NP94	solute carrier family 39 member 2
Malignant neoplasm of breast	MRPS30	Q9NP92	mitochondrial ribosomal protein S30
Malignant neoplasm of breast	TNFRSF12A	Q9NP84	TNF receptor superfamily member 12A
Malignant neoplasm of breast	SARS2	Q9NP81	seryl-tRNA synthetase 2, mitochondrial
Malignant neoplasm of breast	VTA1	Q9NP79	vesicle trafficking 1
Malignant neoplasm of breast	MLXIPL	Q9NP71	MLX interacting protein like
Malignant neoplasm of breast	ARFGAP3	Q9NP61	ADP ribosylation factor GTPase activating protein 3
Malignant neoplasm of breast	IL1RAPL2	Q9NP60	interleukin 1 receptor accessory protein like 2
Malignant neoplasm of breast	SLC40A1	Q9NP59	solute carrier family 40 member 1
Malignant neoplasm of breast	ABCB6	Q9NP58	ATP binding cassette subfamily B member 6 (Langereis blood group)
Malignant neoplasm of breast	PDE7B	Q9NP56	phosphodiesterase 7B

Malignant neoplasm of breast	TUFT1	Q9NNX1	tuftelin 1
Malignant neoplasm of breast	TXNRD2	Q9NNW7	thioredoxin reductase 2
Malignant neoplasm of breast	JPH1	Q9HDC5	junctophilin 1
Malignant neoplasm of breast	NRXN3	Q9HDB5;Q9Y4C0	neurexin 3
Malignant neoplasm of breast	RETN	Q9HD89	resistin
Malignant neoplasm of breast	MYO10	Q9HD67	myosin X
Malignant neoplasm of breast	BCL2L10	Q9HD36	BCL2 like 10
Malignant neoplasm of breast	GOPC	Q9HD26	golgi associated PDZ and coiled-coil motif containing
Malignant neoplasm of breast	SRA1	Q9HD15	steroid receptor RNA activator 1
Malignant neoplasm of breast	S100A14	Q9HCY8	S100 calcium binding protein A14
Malignant neoplasm of breast	ZNF304	Q9HCX3	zinc finger protein 304
Malignant neoplasm of breast	BRMS1	Q9HCU9	BRMS1 transcriptional repressor and anoikis regulator
Malignant neoplasm of breast	CELSR2	Q9HCU4	cadherin EGF LAG seven-pass G-type receptor 2
Malignant neoplasm of breast	TCF7L1	Q9HCS4	transcription factor 7 like 1
Malignant neoplasm of breast	GALNT9	Q9HCQ5	polypeptide N-acetylgalactosaminyltransferase 9
Malignant neoplasm of breast	TP53AIP1	Q9HCN2	tumor protein p53 regulated apoptosis inducing protein 1
Malignant neoplasm of breast	EPB41L5	Q9HCM4	erythrocyte membrane protein band 4.1 like 5
Malignant neoplasm of breast	CHD8	Q9HCK8	chromodomain helicase DNA binding protein 8
Malignant neoplasm of breast	AGO4	Q9HCK5	argonaute RISC component 4

Malignant neoplasm of breast	MAGEE1	Q9HCI5	MAGE family member E1
Malignant neoplasm of breast	ANO8	Q9HCE9	anoctamin 8
Malignant neoplasm of breast	SMURF1	Q9HCE7	SMAD specific E3 ubiquitin protein ligase 1
Malignant neoplasm of breast	ZNF532	Q9HCE3	zinc finger protein 532
Malignant neoplasm of breast	EPG5	Q9HCE0	ectopic P-granules autophagy protein 5 homolog
Malignant neoplasm of breast	TANC2	Q9HCD6	tetratricopeptide repeat, ankyrin repeat and coiled-coil containing 2
Malignant neoplasm of breast	NCOA5	Q9HCD5	nuclear receptor coactivator 5
Malignant neoplasm of breast	GDPD2	Q9HCC8	glycerophosphodiester phosphodiesterase domain containing 2
Malignant neoplasm of breast	MCCC2	Q9HCC0	methylcrotonoyl-CoA carboxylase 2
Malignant neoplasm of breast	NEK6	Q9HC98	NIMA related kinase 6
Malignant neoplasm of breast	GPR35	Q9HC97	G protein-coupled receptor 35
Malignant neoplasm of breast	MUC5B	Q9HC84	mucin 5B, oligomeric mucus/gel-forming
Malignant neoplasm of breast	SENP2	Q9HC62	SUMO specific peptidase 2
Malignant neoplasm of breast	SLC24A3	Q9HC58	solute carrier family 24 member 3
Malignant neoplasm of breast	WFDC1	Q9HC57	WAP four-disulfide core domain 1
Malignant neoplasm of breast	CBX8	Q9HC52	chromobox 8
Malignant neoplasm of breast	EML4	Q9HC35	EMAP like 4
Malignant neoplasm of breast	NOD2	Q9HC29	nucleotide binding oligomerization domain containing 2
Malignant neoplasm of breast	PROK2	Q9HC23	prokineticin 2

Malignant neoplasm of breast	SLC25A19	Q9HC21	solute carrier family 25 member 19
Malignant neoplasm of breast	OTOF	Q9HC10	otoferlin
Malignant neoplasm of breast	ARNT2	Q9HBZ2	aryl hydrocarbon receptor nuclear translocator 2
Malignant neoplasm of breast	NOX3	Q9HBY0	NADPH oxidase 3
Malignant neoplasm of breast	LGR6	Q9HBX8	leucine rich repeat containing G protein-coupled receptor 6
Malignant neoplasm of breast	LRRC4	Q9HBW1	leucine rich repeat containing 4
Malignant neoplasm of breast	LPAR2	Q9HBW0	lysophosphatidic acid receptor 2
Malignant neoplasm of breast	CDH20	Q9HBT6	cadherin 20
Malignant neoplasm of breast	SPC25	Q9HBM1	SPC25 component of NDC80 kinetochore complex
Malignant neoplasm of breast	TNS1	Q9HBL0	tensin 1
Malignant neoplasm of breast	PLAC1	Q9HBJ0	placenta enriched 1
Malignant neoplasm of breast	CYP4F11	Q9HBI6	cytochrome P450 family 4 subfamily F member 11
Malignant neoplasm of breast	PARVB	Q9HBI1	parvin beta
Malignant neoplasm of breast	PARVG	Q9HBI0	parvin gamma
Malignant neoplasm of breast	MKNK2	Q9HBH9	MAPK interacting serine/threonine kinase 2
Malignant neoplasm of breast	IL21	Q9HBE4	interleukin 21
Malignant neoplasm of breast	TRPV4	Q9HBA0	transient receptor potential cation channel subfamily V member 4
Malignant neoplasm of breast	FANCE	Q9HB96	FA complementation group E
Malignant neoplasm of breast	CACYBP	Q9HB71	calcyclin binding protein

Malignant neoplasm of breast	NTN4	Q9HB63	netrin 4
Malignant neoplasm of breast	SP110	Q9HB58	SP110 nuclear body protein
Malignant neoplasm of breast	CYP3A43	Q9HB55	cytochrome P450 family 3 subfamily A member 43
Malignant neoplasm of breast	SCPEP1	Q9HB40	serine carboxypeptidase 1
Malignant neoplasm of breast	BCL2L12	Q9HB09	BCL2 like 12
Malignant neoplasm of breast	C12orf10	Q9HB07	chromosome 12 open reading frame 10
Malignant neoplasm of breast	BCO1	Q9HAY6	beta-carotene oxygenase 1
Malignant neoplasm of breast	MAGEF1	Q9HAY2	MAGE family member F1
Malignant neoplasm of breast	UGT1A8	Q9HAW9	UDP glucuronosyltransferase family 1 member A8
Malignant neoplasm of breast	CLSPN	Q9HAW4	claspin
Malignant neoplasm of breast	BRF2	Q9HAW0	BRF2 RNA polymerase III transcription initiation factor subunit
Malignant neoplasm of breast	EDA2R	Q9HAV5	ectodysplasin A2 receptor
Malignant neoplasm of breast	SMURF2	Q9HAU4	SMAD specific E3 ubiquitin protein ligase 2
Malignant neoplasm of breast	ADGRL3	Q9HAR2	adhesion G protein-coupled receptor L3
Malignant neoplasm of breast	MLXIP	Q9HAP2	MLX interacting protein
Malignant neoplasm of breast	FBRS	Q9HAH7	fibrosin
Malignant neoplasm of breast	SLC52A2	Q9HAB3	solute carrier family 52 member 2
Malignant neoplasm of breast	CERS4	Q9HA82	ceramide synthase 4
Malignant neoplasm of breast	LIN28A	Q9H9Z2	lin-28 homolog A

Malignant neoplasm of breast	ELP3	Q9H9T3	elongator acetyltransferase complex subunit 3
Malignant neoplasm of breast	NANOG	Q9H9S0	Nanog homeobox
Malignant neoplasm of breast	AGO3	Q9H9G7	argonaute RISC catalytic component 3
Malignant neoplasm of breast	SFXN1	Q9H9B4	sideroflexin 1
Malignant neoplasm of breast	EHMT1	Q9H9B1	euchromatic histone lysine methyltransferase 1
Malignant neoplasm of breast	JPT2	Q9H910	Jupiter microtubule associated homolog 2
Malignant neoplasm of breast	LINC00472	Q9H8W2	long intergenic non-protein coding RNA 472
Malignant neoplasm of breast	ECT2	Q9H8V3	epithelial cell transforming 2
Malignant neoplasm of breast	ZNF395	Q9H8N7	zinc finger protein 395
Malignant neoplasm of breast	C10orf88	Q9H8K7	chromosome 10 open reading frame 88
Malignant neoplasm of breast	TUBA4B	Q9H853	tubulin alpha 4b
Malignant neoplasm of breast	DCLRE1B	Q9H816	DNA cross-link repair 1B
Malignant neoplasm of breast	PACC1	Q9H813	proton activated chloride channel 1
Malignant neoplasm of breast	KAT8	Q9H7Z6	lysine acetyltransferase 8
Malignant neoplasm of breast	ZNF703	Q9H7S9	zinc finger protein 703
Malignant neoplasm of breast	SCAF1	Q9H7N4	SR-related CTD associated factor 1
Malignant neoplasm of breast	TDRD3	Q9H7E2	tudor domain containing 3
Malignant neoplasm of breast	WDR26	Q9H7D7	WD repeat domain 26
Malignant neoplasm of breast	SMYD3	Q9H7B4	SET and MYND domain containing 3

Malignant neoplasm of breast	PEAK1	Q9H792	pseudopodium enriched atypical kinase 1
Malignant neoplasm of breast	EGLN3	Q9H6Z9	egl-9 family hypoxia inducible factor 3
Malignant neoplasm of breast	ANTXR1	Q9H6X2	ANTXR cell adhesion molecule 1
Malignant neoplasm of breast	RIOX1	Q9H6W3	ribosomal oxygenase 1
Malignant neoplasm of breast	BCAS3	Q9H6U6	BCAS3 microtubule associated cell migration factor
Malignant neoplasm of breast	ESRP2	Q9H6T0	epithelial splicing regulatory protein 2
Malignant neoplasm of breast	WDCP	Q9H6R7	WD repeat and coiled coil containing
Malignant neoplasm of breast	ILRUN	Q9H6K1	inflammation and lipid regulator with UBA-like and NBR1-like domains
Malignant neoplasm of breast	SOX17	Q9H6I2	SRY-box transcription factor 17
Malignant neoplasm of breast	TUT1	Q9H6E5	terminal uridylyl transferase 1, U6 snRNA-specific
Malignant neoplasm of breast	STN1	Q9H668	STN1 subunit of CST complex
Malignant neoplasm of breast	RERGL	Q9H628	RERG like
Malignant neoplasm of breast	PIF1	Q9H611	PIF1 5'-to-3' DNA helicase
Malignant neoplasm of breast	FAM124B	Q9H5Z6	family with sequence similarity 124 member B
Malignant neoplasm of breast	CDCP1	Q9H5V8	CUB domain containing protein 1
Malignant neoplasm of breast	ZBTB3	Q9H5J0	zinc finger and BTB domain containing 3
Malignant neoplasm of breast	PIEZO2	Q9H5I5	piezo type mechanosensitive ion channel component 2
Malignant neoplasm of breast	SUV39H2	Q9H5I1	suppressor of variegation 3-9 homolog 2
Malignant neoplasm of breast	ZNF644	Q9H582	zinc finger protein 644

Malignant neoplasm of breast	ACTL8	Q9H568	actin like 8
Malignant neoplasm of breast	ALG2	Q9H553	ALG2 alpha-1,3/1,6-mannosyltransferase
Malignant neoplasm of breast	GSTO2	Q9H4Y5	glutathione S-transferase omega 2
Malignant neoplasm of breast	RGCC	Q9H4X1	regulator of cell cycle
Malignant neoplasm of breast	RNF41	Q9H4P4	ring finger protein 41
Malignant neoplasm of breast	SMARCAD1	Q9H4L7	SWI/SNF-related, matrix-associated actin-dependent regulator of chromatin, subfamily a, containing DEAD/H box 1
Malignant neoplasm of breast	RIBC2	Q9H4K1	RIB43A domain with coiled-coils 2
Malignant neoplasm of breast	ZHX3	Q9H4I2	zinc fingers and homeoboxes 3
Malignant neoplasm of breast	FAM83D	Q9H4H8	family with sequence similarity 83 member D
Malignant neoplasm of breast	DEF6	Q9H4E7	DEF6 guanine nucleotide exchange factor
Malignant neoplasm of breast	RHOJ	Q9H4E5	ras homolog family member J
Malignant neoplasm of breast	PLK3	Q9H4B4	polo like kinase 3
Malignant neoplasm of breast	GOLPH3	Q9H4A6	golgi phosphoprotein 3
Malignant neoplasm of breast	WNK1	Q9H4A3	WNK lysine deficient protein kinase 1
Malignant neoplasm of breast	MAP1LC3A	Q9H492	microtubule associated protein 1 light chain 3 alpha
Malignant neoplasm of breast	PIGU	Q9H490	phosphatidylinositol glycan anchor biosynthesis class U
Malignant neoplasm of breast	POFUT1	Q9H488	protein O-fucosyltransferase 1
Malignant neoplasm of breast	CUEDC2	Q9H467	CUE domain containing 2
Malignant neoplasm of breast	SRMS	Q9H3Y6	src-related kinase lacking C-terminal regulatory tyrosine and N-terminal myristylation sites

Malignant neoplasm of breast	UNC45A	Q9H3U1	unc-45 myosin chaperone A
Malignant neoplasm of breast	SEMA6B	Q9H3T3	semaphorin 6B
Malignant neoplasm of breast	PTPN23	Q9H3S7	protein tyrosine phosphatase non-receptor type 23
Malignant neoplasm of breast	TPK1	Q9H3S4	thiamin pyrophosphokinase 1
Malignant neoplasm of breast	SEMA4A	Q9H3S1	semaphorin 4A
Malignant neoplasm of breast	CENPH	Q9H3R5	centromere protein H
Malignant neoplasm of breast	KDM4C	Q9H3R0	lysine demethylase 4C
Malignant neoplasm of breast	ACBD3	Q9H3P7	acyl-CoA binding domain containing 3
Malignant neoplasm of breast	HRH4	Q9H3N8	histamine receptor H4
Malignant neoplasm of breast	TMX1	Q9H3N1	thioredoxin related transmembrane protein 1
Malignant neoplasm of breast	ATXN3L	Q9H3M9	ataxin 3 like
Malignant neoplasm of breast	TXNIP	Q9H3M7	thioredoxin interacting protein
Malignant neoplasm of breast	DPAGT1	Q9H3H5	dolichyl-phosphate N-acetylglucosaminephosphotransferase 1
Malignant neoplasm of breast	TRIT1	Q9H3H1	tRNA isopentenyltransferase 1
Malignant neoplasm of breast	TP63	Q9H3D4	tumor protein p63
Malignant neoplasm of breast	GGNBP2	Q9H3C7	gametogenetin binding protein 2
Malignant neoplasm of breast	FOXP1	Q9H334	forkhead box P1
Malignant neoplasm of breast	EPB41L4B	Q9H329	erythrocyte membrane protein band 4.1 like 4B
Malignant neoplasm of breast	TTYH1	Q9H313	tweety family member 1

Malignant neoplasm of breast	SLCO5A1	Q9H2Y9	solute carrier organic anion transporter family member 5A1
Malignant neoplasm of breast	HIPK2	Q9H2X6	homeodomain interacting protein kinase 2
Malignant neoplasm of breast	KLK15	Q9H2R5	kallikrein related peptidase 15
Malignant neoplasm of breast	TAOK3	Q9H2K8	TAO kinase 3
Malignant neoplasm of breast	TNKS2	Q9H2K2	tankyrase 2
Malignant neoplasm of breast	LRMDA	Q9H2I8	leucine rich melanocyte differentiation associated
Malignant neoplasm of breast	SLC38A1	Q9H2H9	solute carrier family 38 member 1
Malignant neoplasm of breast	SLK	Q9H2G2	STE20 like kinase
Malignant neoplasm of breast	SEMA6A	Q9H2E6	semaphorin 6A
Malignant neoplasm of breast	CXCL16	Q9H2A7	C-X-C motif chemokine ligand 16
Malignant neoplasm of breast	IL25	Q9H293	interleukin 25
Malignant neoplasm of breast	VPS11	Q9H270	VPS11 core subunit of CORVET and HOPS complexes
Malignant neoplasm of breast	CDH23	Q9H251	cadherin related 23
Malignant neoplasm of breast	P2RY12	Q9H244	purinergic receptor P2Y12
Malignant neoplasm of breast	MAGEH1	Q9H213	MAGE family member H1
Malignant neoplasm of breast	CDT1	Q9H211	chromatin licensing and DNA replication factor 1
Malignant neoplasm of breast	OR10A4	Q9H209	olfactory receptor family 10 subfamily A member 4
Malignant neoplasm of breast	OR2AG1	Q9H205	olfactory receptor family 2 subfamily AG member 1 (gene/pseudogene)
Malignant neoplasm of breast	MED28	Q9H204	mediator complex subunit 28

Malignant neoplasm of breast	ECRG4	Q9H1Z8	ECRG4 augurin precursor
Malignant neoplasm of breast	ATG5	Q9H1Y0	autophagy related 5
Malignant neoplasm of breast	SLC25A51	Q9H1U9	solute carrier family 25 member 51
Malignant neoplasm of breast	MEGF9	Q9H1U4	multiple EGF like domains 9
Malignant neoplasm of breast	MYLK2	Q9H1R3	myosin light chain kinase 2
Malignant neoplasm of breast	ISCU	Q9H1K1	iron-sulfur cluster assembly enzyme
Malignant neoplasm of breast	WNT5B	Q9H1J7	Wnt family member 5B
Malignant neoplasm of breast	ASCC2	Q9H1I8	activating signal cointegrator 1 complex subunit 2
Malignant neoplasm of breast	NUCKS1	Q9H1E3	nuclear casein kinase and cyclin dependent kinase substrate 1
Malignant neoplasm of breast	TRPV6	Q9H1D0	transient receptor potential cation channel subfamily V member 6
Malignant neoplasm of breast	MUC3B	Q9H195	mucin 3B, cell surface associated
Malignant neoplasm of breast	SIL1	Q9H173	SIL1 nucleotide exchange factor
Malignant neoplasm of breast	ZBP1	Q9H171	Z-DNA binding protein 1
Malignant neoplasm of breast	BCL11A	Q9H165	BAF chromatin remodeling complex subunit BCL11A
Malignant neoplasm of breast	ALX4	Q9H161	ALX homeobox 4
Malignant neoplasm of breast	ING2	Q9H160	inhibitor of growth family member 2
Malignant neoplasm of breast	RBM38	Q9H0Z9	RNA binding motif protein 38
Malignant neoplasm of breast	ATG10	Q9H0Y0	autophagy related 10
Malignant neoplasm of breast	CCDC8	Q9H0W5	coiled-coil domain containing 8

Malignant neoplasm of breast	RAB1B	Q9H0U4	RAB1B, member RAS oncogene family
Malignant neoplasm of breast	MAGT1	Q9H0U3	magnesium transporter 1
Malignant neoplasm of breast	GABARAPL1	Q9H0R8	GABA type A receptor associated protein like 1
Malignant neoplasm of breast	QRSL1	Q9H0R6	glutaminyl-tRNA amidotransferase subunit QRSL1
Malignant neoplasm of breast	NT5C3A	Q9H0P0	5'-nucleotidase, cytosolic IIIA
Malignant neoplasm of breast	RAB6C	Q9H0N0	RAB6C, member RAS oncogene family
Malignant neoplasm of breast	WWP1	Q9H0M0	WW domain containing E3 ubiquitin protein ligase 1
Malignant neoplasm of breast	SIK2	Q9H0K1	salt inducible kinase 2
Malignant neoplasm of breast	RACGAP1	Q9H0H5	Rac GTPase activating protein 1
Malignant neoplasm of breast	INTS2	Q9H0H0	integrator complex subunit 2
Malignant neoplasm of breast	SHARPIN	Q9H0F6	SHANK associated RH domain interactor
Malignant neoplasm of breast	BRD8	Q9H0E9	bromodomain containing 8
Malignant neoplasm of breast	USP44	Q9H0E7	ubiquitin specific peptidase 44
Malignant neoplasm of breast	ZNF541	Q9H0D2	zinc finger protein 541
Malignant neoplasm of breast	ILKAP	Q9H0C8	ILK associated serine/threonine phosphatase
Malignant neoplasm of breast	CRISPLD2	Q9H0B8	cysteine rich secretory protein LCCL domain containing 2
Malignant neoplasm of breast	NAT10	Q9H0A0	N-acetyltransferase 10
Malignant neoplasm of breast	FAM107B	Q9H098	family with sequence similarity 107 member B
Malignant neoplasm of breast	PAIP1	Q9H074	poly(A) binding protein interacting protein 1

Malignant neoplasm of breast	TMEM126A	Q9H061	transmembrane protein 126A
Malignant neoplasm of breast	HOXD1	Q9GZZ0	homeobox D1
Malignant neoplasm of breast	PBOV1	Q9GZY1	prostate and breast cancer overexpressed 1
Malignant neoplasm of breast	TWSG1	Q9GZX9	twisted gastrulation BMP signaling modulator 1
Malignant neoplasm of breast	AICDA	Q9GZX7	activation induced cytidine deaminase
Malignant neoplasm of breast	IL22	Q9GZX6	interleukin 22
Malignant neoplasm of breast	ZNF350	Q9GZX5	zinc finger protein 350
Malignant neoplasm of breast	PRDM14	Q9GZV8	PR/SET domain 14
Malignant neoplasm of breast	WWTR1	Q9GZV5	WW domain containing transcription regulator 1
Malignant neoplasm of breast	EIF5A2	Q9GZV4	eukaryotic translation initiation factor 5A2
Malignant neoplasm of breast	CTDSP1	Q9GZU7	CTD small phosphatase 1
Malignant neoplasm of breast	PEG3	Q9GZU2	paternally expressed 3
Malignant neoplasm of breast	MCOLN1	Q9GZU1	mucolipin 1
Malignant neoplasm of breast	EGLN1	Q9GZT9	egl-9 family hypoxia inducible factor 1
Malignant neoplasm of breast	SRR	Q9GZT4	serine racemase
Malignant neoplasm of breast	REXO4	Q9GZR2	REX4 homolog, 3'-5' exonuclease
Malignant neoplasm of breast	MAP1LC3B	Q9GZQ8	microtubule associated protein 1 light chain 3 beta
Malignant neoplasm of breast	NMUR2	Q9GZQ4	neuromedin U receptor 2
Malignant neoplasm of breast	DERL2	Q9GZP9	derlin 2

Malignant neoplasm of breast	PDGFD	Q9GZP0	platelet derived growth factor D
Malignant neoplasm of breast	TINAGL1	Q9GZM7	tubulointerstitial nephritis antigen like 1
Malignant neoplasm of breast	SLC39A8	Q9C0K1	solute carrier family 39 member 8
Malignant neoplasm of breast	BHLHE41	Q9C0J9	basic helix-loop-helix family member e41
Malignant neoplasm of breast	SRCIN1	Q9C0H9	SRC kinase signaling inhibitor 1
Malignant neoplasm of breast	ZNF436	Q9C0F3	zinc finger protein 436
Malignant neoplasm of breast	ASXL3	Q9C0F0	ASXL transcriptional regulator 3
Malignant neoplasm of breast	AMBRA1	Q9C0C7	autophagy and beclin 1 regulator 1
Malignant neoplasm of breast	SEMA4C	Q9C0C4	semaphorin 4C
Malignant neoplasm of breast	ZDHHC5	Q9C0B5	zinc finger DHHC-type palmitoyltransferase 5
Malignant neoplasm of breast	FTO	Q9C0B1	FTO alpha-ketoglutarate dependent dioxygenase
Malignant neoplasm of breast	DCLK3	Q9C098	doublecortin like kinase 3
Malignant neoplasm of breast	PKIB	Q9C010	cAMP-dependent protein kinase inhibitor beta
Malignant neoplasm of breast	FOXQ1	Q9C009	forkhead box Q1
Malignant neoplasm of breast	SPRY4	Q9C004	sprouty RTK signaling antagonist 4
Malignant neoplasm of breast	NLRP1	Q9C000	NLR family pyrin domain containing 1
Malignant neoplasm of breast	API5	Q9BZZ5	apoptosis inhibitor 5
Malignant neoplasm of breast	SIGLEC1	Q9BZZ2	sialic acid binding Ig like lectin 1
Malignant neoplasm of breast	UCK2	Q9BZX2	uridine-cytidine kinase 2

Malignant neoplasm of breast	TSGA10	Q9BZW7	testis specific 10
Malignant neoplasm of breast	TM6SF1	Q9BZW5	transmembrane 6 superfamily member 1
Malignant neoplasm of breast	SLC19A3	Q9BZV2	solute carrier family 19 member 3
Malignant neoplasm of breast	FOXP3	Q9BZS1	forkhead box P3
Malignant neoplasm of breast	PRKD2	Q9BZL6	protein kinase D2
Malignant neoplasm of breast	PPP1R12C	Q9BZL4	protein phosphatase 1 regulatory subunit 12C
Malignant neoplasm of breast	UBL5	Q9BZL1	ubiquitin like 5
Malignant neoplasm of breast	TBL1XR1	Q9BZK7	TBL1X receptor 1
Malignant neoplasm of breast	IRX2	Q9BZI1	iroquois homeobox 2
Malignant neoplasm of breast	BARHL1	Q9BZE3	BarH like homeobox 1
Malignant neoplasm of breast	NUF2	Q9BZD4	NUF2 component of NDC80 kinetochore complex
Malignant neoplasm of breast	CPEB1	Q9BZB8	cytoplasmic polyadenylation element binding protein 1
Malignant neoplasm of breast	NSD3	Q9BZ95	nuclear receptor binding SET domain protein 3
Malignant neoplasm of breast	PITPNM3	Q9BZ71	PITPNM family member 3
Malignant neoplasm of breast	ADAM33	Q9BZ11	ADAM metallopeptidase domain 33
Malignant neoplasm of breast	RHOBTB2	Q9BYZ6	Rho related BTB domain containing 2
Malignant neoplasm of breast	LDHAL6B	Q9BYZ2	lactate dehydrogenase A like 6B
Malignant neoplasm of breast	POTEKP	Q9BYX7	POTE ankyrin domain family member K, pseudogene
Malignant neoplasm of breast	SETD2	Q9BYW2	SET domain containing 2, histone lysine methyltransferase

Malignant neoplasm of breast	SRXN1	Q9BYN0	sulfiredoxin 1
Malignant neoplasm of breast	RBCK1	Q9BYM8	RANBP2-type and C3HC4-type zinc finger containing 1
Malignant neoplasm of breast	ACE2	Q9BYF1	angiotensin I converting enzyme 2
Malignant neoplasm of breast	TMPRSS13	Q9BYE2	transmembrane serine protease 13
Malignant neoplasm of breast	MRPL9	Q9BYD2	mitochondrial ribosomal protein L9
Malignant neoplasm of breast	MRPL13	Q9BYD1	mitochondrial ribosomal protein L13
Malignant neoplasm of breast	FUT8	Q9BYC5	fucosyltransferase 8
Malignant neoplasm of breast	GNB1L	Q9BYB4	G protein subunit beta 1 like
Malignant neoplasm of breast	DUSP16	Q9BY84	dual specificity phosphatase 16
Malignant neoplasm of breast	ANGPTL4	Q9BY76	angiopoietin like 4
Malignant neoplasm of breast	CADM1	Q9BY67	cell adhesion molecule 1
Malignant neoplasm of breast	EIF2A	Q9BY44	eukaryotic translation initiation factor 2A
Malignant neoplasm of breast	HDAC8	Q9BY41	histone deacetylase 8
Malignant neoplasm of breast	TEX101	Q9BY14	testis expressed 101
Malignant neoplasm of breast	BEX2	Q9BXY8	brain expressed X-linked 2
Malignant neoplasm of breast	RSPO3	Q9BXY4	R-spondin 3
Malignant neoplasm of breast	MAK16	Q9BXY0	MAK16 homolog
Malignant neoplasm of breast	ANKRD30A	Q9BXX3	ankyrin repeat domain 30A
Malignant neoplasm of breast	EMILIN2	Q9BXX0	elastin microfibril interfacer 2

Malignant neoplasm of breast	FANCD2	Q9BXW9	FA complementation group D2
Malignant neoplasm of breast	MAP1LC3C	Q9BXW4	microtubule associated protein 1 light chain 3 gamma
Malignant neoplasm of breast	SNHG12	Q9BXW3	small nucleolar RNA host gene 12
Malignant neoplasm of breast	RNF17	Q9BXT8	ring finger protein 17
Malignant neoplasm of breast	TEX15	Q9BXT5	testis expressed 15, meiosis and synapsis associated
Malignant neoplasm of breast	NUSAP1	Q9BXS6	nucleolar and spindle associated protein 1
Malignant neoplasm of breast	AP1M1	Q9BXS5	adaptor related protein complex 1 subunit mu 1
Malignant neoplasm of breast	CFHR5	Q9BXR6	complement factor H related 5
Malignant neoplasm of breast	TLR10	Q9BXR5	toll like receptor 10
Malignant neoplasm of breast	QTRT1	Q9BXR0	queuine tRNA-ribosyltransferase catalytic subunit 1
Malignant neoplasm of breast	SLC12A9	Q9BXP2	solute carrier family 12 member 9
Malignant neoplasm of breast	FSD1L	Q9BXM9	fibronectin type III and SPRY domain containing 1 like
Malignant neoplasm of breast	PINK1	Q9BXM7	PTEN induced kinase 1
Malignant neoplasm of breast	CDCA4	Q9BXL8	cell division cycle associated 4
Malignant neoplasm of breast	TBC1D10A	Q9BXI6	TBC1 domain family member 10A
Malignant neoplasm of breast	SPZ1	Q9BXG8	spermatogenic leucine zipper 1
Malignant neoplasm of breast	HCAR1	Q9BXC0	hydroxycarboxylic acid receptor 1
Malignant neoplasm of breast	OSBPL11	Q9BXB4	oxysterol binding protein like 11
Malignant neoplasm of breast	LGR4	Q9BXB1	leucine rich repeat containing G protein-coupled receptor 4

Malignant neoplasm of breast	PLVAP	Q9BX97	plasmalemma vesicle associated protein
Malignant neoplasm of breast	SORBS1	Q9BX66	sorbin and SH3 domain containing 1
Malignant neoplasm of breast	BRIP1	Q9BX63	BRCA1 interacting protein C-terminal helicase 1
Malignant neoplasm of breast	SYCP2	Q9BX26	synaptonemal complex protein 2
Malignant neoplasm of breast	GATA5	Q9BWX5	GATA binding protein 5
Malignant neoplasm of breast	PHF7	Q9BWX1	PHD finger protein 7
Malignant neoplasm of breast	BOC	Q9BWV1	BOC cell adhesion associated, oncogene regulated
Malignant neoplasm of breast	CDK19	Q9BWU1	cyclin dependent kinase 19
Malignant neoplasm of breast	CARD10	Q9BWT7	caspase recruitment domain family member 10
Malignant neoplasm of breast	PAPOLG	Q9BWT3	poly(A) polymerase gamma
Malignant neoplasm of breast	CDCA7	Q9BWT1	cell division cycle associated 7
Malignant neoplasm of breast	FAIM2	Q9BWQ8	Fas apoptotic inhibitory molecule 2
Malignant neoplasm of breast	RHPN1-AS1	Q9BWJ2	RHPN1 antisense RNA 1 (head to head)
Malignant neoplasm of breast	RPAP1	Q9BWH6	RNA polymerase II associated protein 1
Malignant neoplasm of breast	RBM4	Q9BWF3	RNA binding motif protein 4
Malignant neoplasm of breast	REPIN1	Q9BWE0	replication initiator 1
Malignant neoplasm of breast	ACAT2	Q9BWD1	acetyl-CoA acetyltransferase 2
Malignant neoplasm of breast	CCDC106	Q9BWC9	coiled-coil domain containing 106
Malignant neoplasm of breast	DDA1	Q9BW61	DET1 and DDB1 associated 1

Malignant neoplasm of breast	KIFC1	Q9BW19	kinesin family member C1
Malignant neoplasm of breast	TIPIN	Q9BVW5	TIMELESS interacting protein
Malignant neoplasm of breast	GNL3	Q9BVP2	G protein nucleolar 3
Malignant neoplasm of breast	RUSC1	Q9BVN2	RUN and SH3 domain containing 1
Malignant neoplasm of breast	DUSP23	Q9BVJ7	dual specificity phosphatase 23
Malignant neoplasm of breast	PHF20	Q9BVI0	PHD finger protein 20
Malignant neoplasm of breast	ST6GALNAC5	Q9BVH7	ST6 N-acetylgalactosaminide alpha-2,6-sialyltransferase 5
Malignant neoplasm of breast	TRIM62	Q9BVG3	tripartite motif containing 62
Malignant neoplasm of breast	NTMT1	Q9BV86	N-terminal Xaa-Pro-Lys N-methyltransferase 1
Malignant neoplasm of breast	RNF126	Q9BV68	ring finger protein 126
Malignant neoplasm of breast	VAMP8	Q9BV40	vesicle associated membrane protein 8
Malignant neoplasm of breast	TRAF4	Q9BUZ4	TNF receptor associated factor 4
Malignant neoplasm of breast	CHAC1	Q9BUX1	ChaC glutathione specific gamma-glutamylcyclotransferase 1
Malignant neoplasm of breast	BDH2	Q9BUT1	3-hydroxybutyrate dehydrogenase 2
Malignant neoplasm of breast	WRAP53	Q9BUR4	WD repeat containing antisense to TP53
Malignant neoplasm of breast	HTATIP2	Q9BUP3	HIV-1 Tat interactive protein 2
Malignant neoplasm of breast	DERL1	Q9BUN8	derlin 1
Malignant neoplasm of breast	PDCD10	Q9BUL8	programmed cell death 10
Malignant neoplasm of breast	MSTO1	Q9BUK6	misato mitochondrial distribution and morphology regulator 1

Malignant neoplasm of breast	CRB3	Q9BUF7	crumbs cell polarity complex component 3
Malignant neoplasm of breast	MED18	Q9BUE0	mediator complex subunit 18
Malignant neoplasm of breast	TMEM70	Q9BUB7	transmembrane protein 70
Malignant neoplasm of breast	MKNK1	Q9BUB5	MAPK interacting serine/threonine kinase 1
Malignant neoplasm of breast	CHRDL1	Q9BU40	chordin like 1
Malignant neoplasm of breast	LMF2	Q9BU23	lipase maturation factor 2
Malignant neoplasm of breast	FSD1	Q9BTV5	fibronectin type III and SPRY domain containing 1
Malignant neoplasm of breast	TMEM43	Q9BTV4	transmembrane protein 43
Malignant neoplasm of breast	PI4K2A	Q9BTU6	phosphatidylinositol 4-kinase type 2 alpha
Malignant neoplasm of breast	LRRC1	Q9BTT6	leucine rich repeat containing 1
Malignant neoplasm of breast	ANP32E	Q9BTT0	acidic nuclear phosphoprotein 32 family member E
Malignant neoplasm of breast	H2AJ	Q9BTM1	H2A.J histone
Malignant neoplasm of breast	PAGR1	Q9BTK6	PAXIP1 associated glutamate rich protein 1
Malignant neoplasm of breast	MTA3	Q9BTC8	metastasis associated 1 family member 3
Malignant neoplasm of breast	TCHP	Q9BT92	trichoplein keratin filament binding
Malignant neoplasm of breast	SOX7	Q9BT81	SRY-box transcription factor 7
Malignant neoplasm of breast	INPP5K	Q9BT40	inositol polyphosphate-5-phosphatase K
Malignant neoplasm of breast	LIMD2	Q9BT23	LIM domain containing 2
Malignant neoplasm of breast	ALG1	Q9BT22	ALG1 chitobiosyldiphosphodolichol beta-mannosyltransferase

Malignant neoplasm of breast	RTKN	Q9BST9	rhotekin
Malignant neoplasm of breast	MARVELD1	Q9BSK0	MARVEL domain containing 1
Malignant neoplasm of breast	PIMREG	Q9BSJ6	PICALM interacting mitotic regulator
Malignant neoplasm of breast	RHNO1	Q9BSD3	RAD9-HUS1-RAD1 interacting nuclear orphan 1
Malignant neoplasm of breast	LXN	Q9BS40	latexin
Malignant neoplasm of breast	CENPK	Q9BS16	centromere protein K
Malignant neoplasm of breast	TRIM56	Q9BRZ2	tripartite motif containing 56
Malignant neoplasm of breast	SLC50A1	Q9BRV3	solute carrier family 50 member 1
Malignant neoplasm of breast	MIEN1	Q9BRT3	migration and invasion enhancer 1
Malignant neoplasm of breast	LARP6	Q9BRS8	La ribonucleoprotein 6, translational regulator
Malignant neoplasm of breast	RIOK1	Q9BRS2	RIO kinase 1
Malignant neoplasm of breast	ARHGAP9	Q9BRR9	Rho GTPase activating protein 9
Malignant neoplasm of breast	ZKSCAN3	Q9BRR0	zinc finger with KRAB and SCAN domains 3
Malignant neoplasm of breast	ORAI3	Q9BRQ5	ORAI calcium release-activated calcium modulator 3
Malignant neoplasm of breast	PYGO2	Q9BRQ0	pygopus family PHD finger 2
Malignant neoplasm of breast	OVOL2	Q9BRP0	ovo like zinc finger 2
Malignant neoplasm of breast	SDF4	Q9BRK5	stromal cell derived factor 4
Malignant neoplasm of breast	LZTS2	Q9BRK4	leucine zipper tumor suppressor 2
Malignant neoplasm of breast	SH2D3A	Q9BRG2	SH2 domain containing 3A

Malignant neoplasm of breast	PLCD4	Q9BRC7	phospholipase C delta 4
Malignant neoplasm of breast	CORO1B	Q9BR76	coronin 1B
Malignant neoplasm of breast	SULT4A1	Q9BR01	sulfotransferase family 4A member 1
Malignant neoplasm of breast	SLC25A21	Q9BQT8	solute carrier family 25 member 21
Malignant neoplasm of breast	HEPH	Q9BQS7	hephaestin
Malignant neoplasm of breast	GORASP1	Q9BQQ3	golgi reassembly stacking protein 1
Malignant neoplasm of breast	FERMT1	Q9BQL6	fermitin family member 1
Malignant neoplasm of breast	AIF1L	Q9BQI0	allograft inflammatory factor 1 like
Malignant neoplasm of breast	SYT3	Q9BQG1	synaptotagmin 3
Malignant neoplasm of breast	MYBBP1A	Q9BQG0	MYB binding protein 1a
Malignant neoplasm of breast	SENP7	Q9BQF6	SUMO specific peptidase 7
Malignant neoplasm of breast	SELENOS	Q9BQE4	selenoprotein S
Malignant neoplasm of breast	SOST	Q9BQB4	sclerostin
Malignant neoplasm of breast	WDR77	Q9BQA1	WD repeat domain 77
Malignant neoplasm of breast	TBL1Y	Q9BQ87	transducin beta like 1 Y-linked
Malignant neoplasm of breast	MACROD1	Q9BQ69	mono-ADP ribosylhydrolase 1
Malignant neoplasm of breast	PDCD1LG2	Q9BQ51	programmed cell death 1 ligand 2
Malignant neoplasm of breast	MRPL34	Q9BQ48	mitochondrial ribosomal protein L34
Malignant neoplasm of breast	MAPKAP1	Q9BPZ7	MAPK associated protein 1

Malignant neoplasm of breast	HOPX	Q9BPY8	HOP homeobox
Malignant neoplasm of breast	MICU1	Q9BPX6	mitochondrial calcium uptake 1
Malignant neoplasm of breast	GAL3ST1	Q99999	galactose-3-O-sulfotransferase 1
Malignant neoplasm of breast	AKAP9	Q99996	A-kinase anchoring protein 9
Malignant neoplasm of breast	VGLL1	Q99990	vestigial like family member 1
Malignant neoplasm of breast	GDF15	Q99988	growth differentiation factor 15
Malignant neoplasm of breast	VRK1	Q99986	VRK serine/threonine kinase 1
Malignant neoplasm of breast	SEMA3C	Q99985	semaphorin 3C
Malignant neoplasm of breast	TEP1	Q99973	telomerase associated protein 1
Malignant neoplasm of breast	RARRES2	Q99969	retinoic acid receptor responder 2
Malignant neoplasm of breast	CITED2	Q99967	Cbp/p300 interacting transactivator with Glu/Asp rich carboxy-terminal domain 2
Malignant neoplasm of breast	CITED1	Q99966	Cbp/p300 interacting transactivator with Glu/Asp rich carboxy-terminal domain 1
Malignant neoplasm of breast	SH3GL2	Q99962	SH3 domain containing GRB2 like 2, endophilin A1
Malignant neoplasm of breast	SH3GL1	Q99961	SH3 domain containing GRB2 like 1, endophilin A2
Malignant neoplasm of breast	FOXC2	Q99958	forkhead box C2
Malignant neoplasm of breast	DUSP9	Q99956	dual specificity phosphatase 9
Malignant neoplasm of breast	PRRT1	Q99946	proline rich transmembrane protein 1
Malignant neoplasm of breast	RNF5	Q99942	ring finger protein 5
Malignant neoplasm of breast	OPRPN	Q99935	opiorphin prepropeptide

Malignant neoplasm of breast	BAG1	Q99933	BAG cochaperone 1
Malignant neoplasm of breast	SPAG8	Q99932	sperm associated antigen 8
Malignant neoplasm of breast	ASCL2	Q99929	achaete-scute family bHLH transcription factor 2
Malignant neoplasm of breast	PRMT1	Q99873	protein arginine methyltransferase 1
Malignant neoplasm of breast	EBNA1BP2	Q99848	EBNA1 binding protein 2
Malignant neoplasm of breast	MYD88	Q99836	MYD88 innate immune signal transduction adaptor
Malignant neoplasm of breast	SMO	Q99835	smoothened, frizzled class receptor
Malignant neoplasm of breast	CIB1	Q99828	calcium and integrin binding 1
Malignant neoplasm of breast	ARHGDIG	Q99819	Rho GDP dissociation inhibitor gamma
Malignant neoplasm of breast	TSG101	Q99816	tumor susceptibility 101
Malignant neoplasm of breast	EPAS1	Q99814	endothelial PAS domain protein 1
Malignant neoplasm of breast	PRRX2	Q99811	paired related homeobox 2
Malignant neoplasm of breast	NKX3-1	Q99801	NK3 homeobox 1
Malignant neoplasm of breast	ACO2	Q99798	aconitase 2
Malignant neoplasm of breast	CMKLR1	Q99788	chemerin chemokine-like receptor 1
Malignant neoplasm of breast	MAP3K3	Q99759	mitogen-activated protein kinase kinase kinase 3
Malignant neoplasm of breast	ABCA3	Q99758	ATP binding cassette subfamily A member 3
Malignant neoplasm of breast	TXN2	Q99757	thioredoxin 2
Malignant neoplasm of breast	PIP5K1A	Q99755	phosphatidylinositol-4-phosphate 5-kinase type 1 alpha

Malignant neoplasm of breast	NPAS2	Q99743	neuronal PAS domain protein 2
Malignant neoplasm of breast	CDC6	Q99741	cell division cycle 6
Malignant neoplasm of breast	CCL19	Q99731	C-C motif chemokine ligand 19
Malignant neoplasm of breast	BARD1	Q99728	BRCA1 associated RING domain 1
Malignant neoplasm of breast	TIMP4	Q99727	TIMP metallopeptidase inhibitor 4
Malignant neoplasm of breast	SIGMAR1	Q99720	sigma non-opioid intracellular receptor 1
Malignant neoplasm of breast	SMAD5	Q99717	SMAD family member 5
Malignant neoplasm of breast	COL12A1	Q99715	collagen type XII alpha 1 chain
Malignant neoplasm of breast	KCNJ15	Q99712	potassium inwardly rectifying channel subfamily J member 15
Malignant neoplasm of breast	RBBP8	Q99708	RB binding protein 8, endonuclease
Malignant neoplasm of breast	MTR	Q99707	5-methyltetrahydrofolate-homocysteine methyltransferase
Malignant neoplasm of breast	DOK1	Q99704	docking protein 1
Malignant neoplasm of breast	PITX2	Q99697	paired like homeodomain 2
Malignant neoplasm of breast	FEZ1	Q99689	fasciculation and elongation protein zeta 1
Malignant neoplasm of breast	MGLL	Q99685	monoglyceride lipase
Malignant neoplasm of breast	MAP3K5	Q99683	mitogen-activated protein kinase kinase kinase 5
Malignant neoplasm of breast	CGRRF1	Q99675	cell growth regulator with ring finger domain 1
Malignant neoplasm of breast	KIF2C	Q99661	kinesin family member 2C
Malignant neoplasm of breast	OSMR	Q99650	oncostatin M receptor

Malignant neoplasm of breast	PKMYT1	Q99640	protein kinase, membrane associated tyrosine/threonine 1
Malignant neoplasm of breast	RAD9A	Q99638	RAD9 checkpoint clamp component A
Malignant neoplasm of breast	COPS8	Q99627	COP9 signalosome subunit 8
Malignant neoplasm of breast	CDX2	Q99626	caudal type homeobox 2
Malignant neoplasm of breast	SLC38A3	Q99624	solute carrier family 38 member 3
Malignant neoplasm of breast	PHB2	Q99623	prohibitin 2
Malignant neoplasm of breast	SPSB2	Q99619	splA/ryanodine receptor domain and SOCS box containing 2
Malignant neoplasm of breast	EIF3C	Q99613	eukaryotic translation initiation factor 3 subunit C
Malignant neoplasm of breast	KLF6	Q99612	Kruppel like factor 6
Malignant neoplasm of breast	SEPHS2	Q99611	selenophosphate synthetase 2
Malignant neoplasm of breast	NDN	Q99608	necdin, MAGE family member
Malignant neoplasm of breast	ELF4	Q99607	E74 like ETS transcription factor 4
Malignant neoplasm of breast	TIMM17A	Q99595	translocase of inner mitochondrial membrane 17A
Malignant neoplasm of breast	TBX5	Q99593	T-box transcription factor 5
Malignant neoplasm of breast	SCAF11	Q99590	SR-related CTD associated factor 11
Malignant neoplasm of breast	MNT	Q99583	MAX network transcriptional repressor
Malignant neoplasm of breast	POP1	Q99575	POP1 homolog, ribonuclease P/MRP subunit
Malignant neoplasm of breast	P2RX7	Q99572	purinergic receptor P2X 7
Malignant neoplasm of breast	PIK3R4	Q99570	phosphoinositide-3-kinase regulatory subunit 4

Malignant neoplasm of breast	NUP88	Q99567	nucleoporin 88
Malignant neoplasm of breast	MAP3K14	Q99558	mitogen-activated protein kinase kinase kinase 14
Malignant neoplasm of breast	DNAJC2	Q99543	DnaJ heat shock protein family (Hsp40) member C2
Malignant neoplasm of breast	PLIN2	Q99541	perilipin 2
Malignant neoplasm of breast	LGMN	Q99538	legumain
Malignant neoplasm of breast	VAT1	Q99536	vesicle amine transport 1
Malignant neoplasm of breast	GPER1	Q99527	G protein-coupled estrogen receptor 1
Malignant neoplasm of breast	SORT1	Q99523	sortilin 1
Malignant neoplasm of breast	NEU1	Q99519	neuraminidase 1
Malignant neoplasm of breast	EYA1	Q99502	EYA transcriptional coactivator and phosphatase 1
Malignant neoplasm of breast	S1PR3	Q99500	sphingosine-1-phosphate receptor 3
Malignant neoplasm of breast	PARK7	Q99497	Parkinsonism associated deglycase
Malignant neoplasm of breast	RNF2	Q99496	ring finger protein 2
Malignant neoplasm of breast	AGAP2	Q99490	ArfGAP with GTPase domain, ankyrin repeat and PH domain 2
Malignant neoplasm of breast	DDO	Q99489	D-aspartate oxidase
Malignant neoplasm of breast	SDF2	Q99470	stromal cell derived factor 2
Malignant neoplasm of breast	NOTCH4	Q99466	notch receptor 4
Malignant neoplasm of breast	PSMD1	Q99460	proteasome 26S subunit, non-ATPase 1
Malignant neoplasm of breast	CDC5L	Q99459	cell division cycle 5 like

Malignant neoplasm of breast	SEC62	Q99442	SEC62 homolog, preprotein translocation factor
Malignant neoplasm of breast	ATP6V0B	Q99437	ATPase H+ transporting V0 subunit b
Malignant neoplasm of breast	PSMB7	Q99436	proteasome 20S subunit beta 7
Malignant neoplasm of breast	ACOX2	Q99424	acyl-CoA oxidase 2
Malignant neoplasm of breast	SCN2A	Q99250	sodium voltage-gated channel alpha subunit 2
Malignant neoplasm of breast	AMELX	Q99217	amelogenin X-linked
Malignant neoplasm of breast	MUC4	Q99102	mucin 4, cell surface associated
Malignant neoplasm of breast	TCF12	Q99081	transcription factor 12
Malignant neoplasm of breast	HBEGF	Q99075	heparin binding EGF like growth factor
Malignant neoplasm of breast	RMDN3	Q96TC7	regulator of microtubule dynamics 3
Malignant neoplasm of breast	YME1L1	Q96TA2	YME1 like 1 ATPase
Malignant neoplasm of breast	NIBAN2	Q96TA1	niban apoptosis regulator 2
Malignant neoplasm of breast	UHRF1	Q96T88	ubiquitin like with PHD and ring finger domains 1
Malignant neoplasm of breast	SLC9A7	Q96T83	solute carrier family 9 member A7
Malignant neoplasm of breast	SPEN	Q96T58	spen family transcriptional repressor
Malignant neoplasm of breast	MBOAT4	Q96T53	membrane bound O-acyltransferase domain containing 4
Malignant neoplasm of breast	IMMP2L	Q96T52	inner mitochondrial membrane peptidase subunit 2
Malignant neoplasm of breast	RUFY1	Q96T51	RUN and FYVE domain containing 1
Malignant neoplasm of breast	RSF1	Q96T23	remodeling and spacing factor 1

Malignant neoplasm of breast	MAP7D2	Q96T17	MAP7 domain containing 2
Malignant neoplasm of breast	SIN3A	Q96ST3	SIN3 transcription regulator family member A
Malignant neoplasm of breast	CYP2S1	Q96SQ9	cytochrome P450 family 2 subfamily S member 1
Malignant neoplasm of breast	CPXM1	Q96SM3	carboxypeptidase X, M14 family member 1
Malignant neoplasm of breast	GPX7	Q96SL4	glutathione peroxidase 7
Malignant neoplasm of breast	STRBP	Q96SI9	spermatid perinuclear RNA binding protein
Malignant neoplasm of breast	DCLRE1C	Q96SD1	DNA cross-link repair 1C
Malignant neoplasm of breast	SMC6	Q96SB8	structural maintenance of chromosomes 6
Malignant neoplasm of breast	SRPK1	Q96SB4	SRSF protein kinase 1
Malignant neoplasm of breast	PPP1R9B	Q96SB3	protein phosphatase 1 regulatory subunit 9B
Malignant neoplasm of breast	PEBP4	Q96S96	phosphatidylethanolamine binding protein 4
Malignant neoplasm of breast	HAPLN3	Q96S86	hyaluronan and proteoglycan link protein 3
Malignant neoplasm of breast	RASL10B	Q96S79	RAS like family 10 member B
Malignant neoplasm of breast	RANBP9	Q96S59	RAN binding protein 9
Malignant neoplasm of breast	PIGS	Q96S52	phosphatidylinositol glycan anchor biosynthesis class S
Malignant neoplasm of breast	NODAL	Q96S42	nodal growth differentiation factor
Malignant neoplasm of breast	TRIB1	Q96RU8	tribbles pseudokinase 1
Malignant neoplasm of breast	TRIB3	Q96RU7	tribbles pseudokinase 3
Malignant neoplasm of breast	USP28	Q96RU2	ubiquitin specific peptidase 28

Malignant neoplasm of breast	CAMKK2	Q96RR4	calcium/calmodulin dependent protein kinase kinase 2
Malignant neoplasm of breast	GFM1	Q96RP9	G elongation factor mitochondrial 1
Malignant neoplasm of breast	UIMC1	Q96RL1	ubiquitin interaction motif containing 1
Malignant neoplasm of breast	CITED4	Q96RK1	Cbp/p300 interacting transactivator with Glu/Asp rich carboxy-terminal domain 4
Malignant neoplasm of breast	CIC	Q96RK0	capicua transcriptional repressor
Malignant neoplasm of breast	TNFRSF13C	Q96RJ3	TNF receptor superfamily member 13C
Malignant neoplasm of breast	TAAR1	Q96RJ0	trace amine associated receptor 1
Malignant neoplasm of breast	NR1H4	Q96RI1	nuclear receptor subfamily 1 group H member 4
Malignant neoplasm of breast	F2RL3	Q96RI0	F2R like thrombin or trypsin receptor 3
Malignant neoplasm of breast	ZNF300	Q96RE9	zinc finger protein 300
Malignant neoplasm of breast	FCRL5	Q96RD9	Fc receptor like 5
Malignant neoplasm of breast	PANX1	Q96RD7	pannexin 1
Malignant neoplasm of breast	PANX2	Q96RD6	pannexin 2
Malignant neoplasm of breast	SPAG5	Q96R06	sperm associated antigen 5
Malignant neoplasm of breast	HHIP	Q96QV1	hedgehog interacting protein
Malignant neoplasm of breast	ACCS	Q96QU6	1-aminocyclopropane-1-carboxylate synthase homolog (inactive)
Malignant neoplasm of breast	TRPM7	Q96QT4	transient receptor potential cation channel subfamily M member 7
Malignant neoplasm of breast	SCGB3A1	Q96QR1	secretoglobin family 3A member 1
Malignant neoplasm of breast	ALPK1	Q96QP1	alpha kinase 1

Malignant neoplasm of breast	SLC38A2	Q96QD8	solute carrier family 38 member 2
Malignant neoplasm of breast	DLC1	Q96QB1	DLC1 Rho GTPase activating protein
Malignant neoplasm of breast	YPEL2	Q96QA6	yippee like 2
Malignant neoplasm of breast	KIF20B	Q96Q89	kinesin family member 20B
Malignant neoplasm of breast	ALKBH3	Q96Q83	alkB homolog 3, alpha-ketoglutaratedependent dioxygenase
Malignant neoplasm of breast	DERL3	Q96Q80	derlin 3
Malignant neoplasm of breast	CDK15	Q96Q40	cyclin dependent kinase 15
Malignant neoplasm of breast	FLACC1	Q96Q35	flagellum associated containing coiled-coil domains 1
Malignant neoplasm of breast	SMG1	Q96Q15	SMG1 nonsense mediated mRNA decay associated PI3K related kinase
Malignant neoplasm of breast	TRAPPC9	Q96Q05	trafficking protein particle complex 9
Malignant neoplasm of breast	LMTK3	Q96Q04	lemur tyrosine kinase 3
Malignant neoplasm of breast	CSMD1	Q96PZ7	CUB and Sushi multiple domains 1
Malignant neoplasm of breast	PSMG3-AS1	Q96PY0	PSMG3 antisense RNA 1 (head to head)
Malignant neoplasm of breast	CCDC85A	Q96PX6	coiled-coil domain containing 85A
Malignant neoplasm of breast	NEDD4L	Q96PU5	NEDD4 like E3 ubiquitin protein ligase
Malignant neoplasm of breast	UHRF2	Q96PU4	ubiquitin like with PHD and ring finger domains 2
Malignant neoplasm of breast	AQP10	Q96PS8	aquaporin 10
Malignant neoplasm of breast	TRERF1	Q96PN7	transcriptional regulating factor 1
Malignant neoplasm of breast	RCHY1	Q96PM5	ring finger and CHY zinc finger domain containing 1

Malignant neoplasm of breast	SCGB3A2	Q96PL1	secretoglobin family 3A member 2
Malignant neoplasm of breast	RBM14-RBM4	Q96PK6	RBM14-RBM4 readthrough
Malignant neoplasm of breast	RBM14	Q96PK6	RNA binding motif protein 14
Malignant neoplasm of breast	NOX5	Q96PH1	NADPH oxidase 5
Malignant neoplasm of breast	BBC3	Q96PG8;Q9BXH1	BCL2 binding component 3
Malignant neoplasm of breast	TGM7	Q96PF1	transglutaminase 7
Malignant neoplasm of breast	IL17F	Q96PD4	interleukin 17F
Malignant neoplasm of breast	MIA2	Q96PC5	MIA SH3 domain ER export factor 2
Malignant neoplasm of breast	LRRC3B	Q96PB8	leucine rich repeat containing 3B
Malignant neoplasm of breast	CASD1	Q96PB1	CAS1 domain containing 1
Malignant neoplasm of breast	NECAB3	Q96P71	N-terminal EF-hand calcium binding protein 3
Malignant neoplasm of breast	IPO9	Q96P70	importin 9
Malignant neoplasm of breast	FCRL3	Q96P31	Fc receptor like 3
Malignant neoplasm of breast	NLRP3	Q96P20	NLR family pyrin domain containing 3
Malignant neoplasm of breast	RPRD1A	Q96P16	regulation of nuclear pre-mRNA domain containing 1A
Malignant neoplasm of breast	NSUN5	Q96P11	NOP2/Sun RNA methyltransferase 5
Malignant neoplasm of breast	BIRC8	Q96P09	baculoviral IAP repeat containing 8
Malignant neoplasm of breast	PRAP1	Q96NZ9	proline rich acidic protein 1
Malignant neoplasm of breast	FOXN4	Q96NZ1	forkhead box N4

Malignant neoplasm of breast	MUS81	Q96NY9	MUS81 structure-specific endonuclease subunit
Malignant neoplasm of breast	NECTIN4	Q96NY8	nectin cell adhesion molecule 4
Malignant neoplasm of breast	LRRC7	Q96NW7	leucine rich repeat containing 7
Malignant neoplasm of breast	AIFM3	Q96NN9	apoptosis inducing factor mitochondria associated 3
Malignant neoplasm of breast	FAM210A	Q96ND0	family with sequence similarity 210 member A
Malignant neoplasm of breast	LINC00052	Q96N35	long intergenic non-protein coding RNA 52
Malignant neoplasm of breast	CFAP54	Q96N23	cilia and flagella associated protein 54
Malignant neoplasm of breast	ZNF75A	Q96N20	zinc finger protein 75a
Malignant neoplasm of breast	WDR92	Q96MX6	WD repeat domain 92
Malignant neoplasm of breast	ROBO3	Q96MS0	roundabout guidance receptor 3
Malignant neoplasm of breast	C5orf34	Q96MH7	chromosome 5 open reading frame 34
Malignant neoplasm of breast	NSMCE2	Q96MF7	NSE2 (MMS21) homolog, SMC5-SMC6 complex SUMO ligase
Malignant neoplasm of breast	FGD4	Q96M96	FYVE, RhoGEF and PH domain containing 4
Malignant neoplasm of breast	TBATA	Q96M53	thymus, brain and testes associated
Malignant neoplasm of breast	HYLS1	Q96M11	HYLS1 centriolar and ciliogenesis associated
Malignant neoplasm of breast	C10orf90	Q96M02	chromosome 10 open reading frame 90
Malignant neoplasm of breast	RMDN2	Q96LZ7	regulator of microtubule dynamics 2
Malignant neoplasm of breast	IMMP1L	Q96LU5	inner mitochondrial membrane peptidase subunit 1
Malignant neoplasm of breast	UBE2E2	Q96LR5	ubiquitin conjugating enzyme E2 E2

Malignant neoplasm of breast	TAFA4	Q96LR4	TAFA chemokine like family member 4
Malignant neoplasm of breast	BMF	Q96LC9	Bcl2 modifying factor
Malignant neoplasm of breast	MRGPRX1	Q96LB2	MAS related GPR family member X1
Malignant neoplasm of breast	MRGPRX3	Q96LB0	MAS related GPR family member X3
Malignant neoplasm of breast	MRGPRX4	Q96LA9	MAS related GPR family member X4
Malignant neoplasm of breast	PRMT6	Q96LA8	protein arginine methyltransferase 6
Malignant neoplasm of breast	KIF16B	Q96L93	kinesin family member 16B
Malignant neoplasm of breast	SNX27	Q96L92	sorting nexin 27
Malignant neoplasm of breast	NSD1	Q96L73	nuclear receptor binding SET domain protein 1
Malignant neoplasm of breast	KCNH8	Q96L42	potassium voltage-gated channel subfamily H member 8
Malignant neoplasm of breast	MARK4	Q96L34	microtubule affinity regulating kinase 4
Malignant neoplasm of breast	SUSD3	Q96L08	sushi domain containing 3
Malignant neoplasm of breast	EGLN2	Q96KS0	egl-9 family hypoxia inducible factor 2
Malignant neoplasm of breast	LMLN	Q96KR4	leishmanolysin like peptidase
Malignant neoplasm of breast	EHMT2	Q96KQ7	euchromatic histone lysine methyltransferase 2
Malignant neoplasm of breast	PPP1R13B	Q96KQ4	protein phosphatase 1 regulatory subunit 13B
Malignant neoplasm of breast	TNIP3	Q96KP6	TNFAIP3 interacting protein 3
Malignant neoplasm of breast	LRATD2	Q96KN1	LRAT domain containing 2
Malignant neoplasm of breast	PBK	Q96KB5	PDZ binding kinase

Malignant neoplasm of breast	CLPTM1L	Q96KA5	CLPTM1 like
Malignant neoplasm of breast	ZNF668	Q96K58	zinc finger protein 668
Malignant neoplasm of breast	PDLIM2	Q96JY6	PDZ and LIM domain 2
Malignant neoplasm of breast	MAEL	Q96JY0	maelstrom spermatogenic transposon silencer
Malignant neoplasm of breast	FEM1C	Q96JP0	fem-1 homolog C
Malignant neoplasm of breast	LCOR	Q96JN0	ligand dependent nuclear receptor corepressor
Malignant neoplasm of breast	L3MBTL3	Q96JM7	L3MBTL histone methyl-lysine binding protein 3
Malignant neoplasm of breast	ZNF469	Q96JG9	zinc finger protein 469
Malignant neoplasm of breast	MAGED4	Q96JG8	MAGE family member D4
Malignant neoplasm of breast	MAGED4B	Q96JG8	MAGE family member D4B
Malignant neoplasm of breast	ST6GAL2	Q96JF0	ST6 beta-galactoside alpha-2,6-sialyltransferase 2
Malignant neoplasm of breast	SEC16B	Q96JE7	SEC16 homolog B, endoplasmic reticulum export factor
Malignant neoplasm of breast	VPS39	Q96JC1	VPS39 subunit of HOPS complex
Malignant neoplasm of breast	LOXL4	Q96JB6	lysyl oxidase like 4
Malignant neoplasm of breast	COG3	Q96JB2	component of oligomeric golgi complex 3
Malignant neoplasm of breast	DNAH8	Q96JB1	dynein axonemal heavy chain 8
Malignant neoplasm of breast	PLEKHA8	Q96JA3	pleckstrin homology domain containing A8
Malignant neoplasm of breast	LRIG1	Q96JA1	leucine rich repeats and immunoglobulin like domains 1
Malignant neoplasm of breast	PIWIL1	Q96J94	piwi like RNA-mediated gene silencing 1

Malignant neoplasm of breast	EPSTI1	Q96J88	epithelial stromal interaction 1
Malignant neoplasm of breast	ABCC11	Q96J66	ATP binding cassette subfamily C member 11
Malignant neoplasm of breast	ABCC12	Q96J65	ATP binding cassette subfamily C member 12
Malignant neoplasm of breast	TXNDC15	Q96J42	thioredoxin domain containing 15
Malignant neoplasm of breast	ITCH	Q96J02	itchy E3 ubiquitin protein ligase
Malignant neoplasm of breast	PAWR	Q96IZ0	pro-apoptotic WT1 regulator
Malignant neoplasm of breast	CPB2	Q96IY4	carboxypeptidase B2
Malignant neoplasm of breast	ABHD14B	Q96IU4	abhydrolase domain containing 14B
Malignant neoplasm of breast	ZNF496	Q96IT1	zinc finger protein 496
Malignant neoplasm of breast	TENT5A	Q96IP4	terminal nucleotidyltransferase 5A
Malignant neoplasm of breast	BOD1	Q96IK1	biorientation of chromosomes in cell division 1
Malignant neoplasm of breast	ABHD8	Q96I13	abhydrolase domain containing 8
Malignant neoplasm of breast	HES6	Q96HZ4	hes family bHLH transcription factor 6
Malignant neoplasm of breast	SGSM3	Q96HU1	small G protein signaling modulator 3
Malignant neoplasm of breast	NAF1	Q96HR8	nuclear assembly factor 1 ribonucleoprotein
Malignant neoplasm of breast	SFRP2	Q96HF1	secreted frizzled related protein 2
Malignant neoplasm of breast	PRR11	Q96HE9	proline rich 11
Malignant neoplasm of breast	ERO1A	Q96HE7	endoplasmic reticulum oxidoreductase 1 alpha
Malignant neoplasm of breast	PDLIM5	Q96HC4	PDZ and LIM domain 5

Malignant neoplasm of breast	APIP	Q96GX9	APAF1 interacting protein
Malignant neoplasm of breast	MASTL	Q96GX5	microtubule associated serine/threonine kinase like
Malignant neoplasm of breast	ATXN7L3B	Q96GX2	ataxin 7 like 3B
Malignant neoplasm of breast	MACIR	Q96GV9	macrophage immunometabolism regulator
Malignant neoplasm of breast	ACSBG1	Q96GR2	acyl-CoA synthetase bubblegum family member 1
Malignant neoplasm of breast	C16orf58	Q96GQ5	chromosome 16 open reading frame 58
Malignant neoplasm of breast	SCARF2	Q96GP6	scavenger receptor class F member 2
Malignant neoplasm of breast	SMARCD1	Q96GM5	SWI/SNF related, matrix associated, actin dependent regulator of chromatin, subfamily d, member 1
Malignant neoplasm of breast	AURKB	Q96GD4	aurora kinase B
Malignant neoplasm of breast	PDXP	Q96GD0	pyridoxal phosphatase
Malignant neoplasm of breast	VMP1	Q96GC9	vacuole membrane protein 1
Malignant neoplasm of breast	SLC35A4	Q96G79	solute carrier family 35 member A4
Malignant neoplasm of breast	KLHDC7B	Q96G42	kelch domain containing 7B
Malignant neoplasm of breast	CERS2	Q96G23	ceramide synthase 2
Malignant neoplasm of breast	PERP	Q96FX8	p53 apoptosis effector related to PMP22
Malignant neoplasm of breast	OTUB1	Q96FW1	OTU deubiquitinase, ubiquitin aldehyde binding 1
Malignant neoplasm of breast	THOC1	Q96FV9	THO complex 1
Malignant neoplasm of breast	SIPA1	Q96FS4	signal-induced proliferation-associated 1
Malignant neoplasm of breast	S100A16	Q96FQ6	S100 calcium binding protein A16

Malignant neoplasm of breast	KIF12	Q96FN5	kinesin family member 12
Malignant neoplasm of breast	PGAP3	Q96FM1	post-GPI attachment to proteins phospholipase 3
Malignant neoplasm of breast	GALNT14	Q96FL9	polypeptide N-acetylgalactosaminyltransferase 14
Malignant neoplasm of breast	DYNLL2	Q96FJ2	dynein light chain LC8-type 2
Malignant neoplasm of breast	CDCA5	Q96FF9	cell division cycle associated 5
Malignant neoplasm of breast	PIK3IP1	Q96FE7	phosphoinositide-3-kinase interacting protein 1
Malignant neoplasm of breast	ZNF503	Q96F45	zinc finger protein 503
Malignant neoplasm of breast	TRIM11	Q96F44	tripartite motif containing 11
Malignant neoplasm of breast	NRBF2	Q96F24	nuclear receptor binding factor 2
Malignant neoplasm of breast	SAT2	Q96F10	spermidine/spermine N1-acetyltransferase family member 2
Malignant neoplasm of breast	CYFIP2	Q96F07	cytoplasmic FMR1 interacting protein 2
Malignant neoplasm of breast	MYEOV	Q96EZ4	myeloma overexpressed
Malignant neoplasm of breast	ADAT3	Q96EY9	adenosine deaminase tRNA specific 3
Malignant neoplasm of breast	DNAJA3	Q96EY1	DnaJ heat shock protein family (Hsp40) member A3
Malignant neoplasm of breast	WDR34	Q96EX3	WD repeat domain 34
Malignant neoplasm of breast	DAZAP1	Q96EP5	DAZ associated protein 1
Malignant neoplasm of breast	CHFR	Q96EP1	checkpoint with forkhead and ring finger domains
Malignant neoplasm of breast	RNF31	Q96EP0	ring finger protein 31
Malignant neoplasm of breast	MTMR8	Q96EF0	myotubularin related protein 8

Malignant neoplasm of breast	SIRT1	Q96EB6	sirtuin 1
Malignant neoplasm of breast	KLRG1	Q96E93	killer cell lectin like receptor G1
Malignant neoplasm of breast	OMA1	Q96E52	OMA1 zinc metallopeptidase
Malignant neoplasm of breast	SPACA9	Q96E40	sperm acrosome associated 9
Malignant neoplasm of breast	NUS1	Q96E22	NUS1 dehydrodolichyl diphosphate synthase subunit
Malignant neoplasm of breast	MTBP	Q96DY7	MDM2 binding protein
Malignant neoplasm of breast	RTP4	Q96DX8	receptor transporter protein 4
Malignant neoplasm of breast	TRIM44	Q96DX7	tripartite motif containing 44
Malignant neoplasm of breast	ZBTB10	Q96DT7	zinc finger and BTB domain containing 10
Malignant neoplasm of breast	ATG4C	Q96DT6	autophagy related 4C cysteine peptidase
Malignant neoplasm of breast	MUCL1	Q96DR8	mucin like 1
Malignant neoplasm of breast	RMC1	Q96DM3	regulator of MON1-CCZ1
Malignant neoplasm of breast	MSI2	Q96DH6	musashi RNA binding protein 2
Malignant neoplasm of breast	ESS2	Q96DF8	ess-2 splicing factor homolog
Malignant neoplasm of breast	FXYD5	Q96DB9	FXYD domain containing ion transport regulator 5
Malignant neoplasm of breast	RMDN1	Q96DB5	regulator of microtubule dynamics 1
Malignant neoplasm of breast	HDAC11	Q96DB2	histone deacetylase 11
Malignant neoplasm of breast	HVCN1	Q96D96	hydrogen voltage gated channel 1
Malignant neoplasm of breast	HAVCR1	Q96D42	hepatitis A virus cellular receptor 1

Malignant neoplasm of breast	ORAI1	Q96D31	ORAI calcium release-activated calcium modulator 1
Malignant neoplasm of breast	ZNF501	Q96CX3	zinc finger protein 501
Malignant neoplasm of breast	OPTN	Q96CV9	optineurin
Malignant neoplasm of breast	ISOC1	Q96CN7	isochorismatase domain containing 1
Malignant neoplasm of breast	CTHRC1	Q96CG8	collagen triple helix repeat containing 1
Malignant neoplasm of breast	RHBDF1	Q96CC6	rhomboid 5 homolog 1
Malignant neoplasm of breast	DCPS	Q96C86	decapping enzyme, scavenger
Malignant neoplasm of breast	ROPN1L	Q96C74	rhophilin associated tail protein 1 like
Malignant neoplasm of breast	UPRT	Q96BW1	uracil phosphoribosyltransferase homolog
Malignant neoplasm of breast	ALKBH8	Q96BT7	alkB homolog 8, tRNA methyltransferase
Malignant neoplasm of breast	TESC	Q96BS2	tescalcin
Malignant neoplasm of breast	SGK3	Q96BR1	serum/glucocorticoid regulated kinase family member 3
Malignant neoplasm of breast	PINX1	Q96BK5	PIN2 (TERF1) interacting telomerase inhibitor 1
Malignant neoplasm of breast	SLC22A18	Q96BI1	solute carrier family 22 member 18
Malignant neoplasm of breast	SPSB1	Q96BD6	splA/ryanodine receptor domain and SOCS box containing 1
Malignant neoplasm of breast	SLCO4A1	Q96BD0	solute carrier organic anion transporter family member 4A1
Malignant neoplasm of breast	CREB3L1	Q96BA8	cAMP responsive element binding protein 3 like 1
Malignant neoplasm of breast	SH3KBP1	Q96B97	SH3 domain containing kinase binding protein 1
Malignant neoplasm of breast	RGMA	Q96B86	repulsive guidance molecule BMP co-receptor a

Malignant neoplasm of breast	ARRDC3	Q96B67	arrestin domain containing 3
Malignant neoplasm of breast	AKT1S1	Q96B36	AKT1 substrate 1
Malignant neoplasm of breast	ISG20	Q96AZ6	interferon stimulated exonuclease gene 20
Malignant neoplasm of breast	MIB2	Q96AX9	mindbomb E3 ubiquitin protein ligase 2
Malignant neoplasm of breast	E2F7	Q96AV8	E2F transcription factor 7
Malignant neoplasm of breast	PBXIP1	Q96AQ6	PBX homeobox interacting protein 1
Malignant neoplasm of breast	TMEM125	Q96AQ2	transmembrane protein 125
Malignant neoplasm of breast	ZUP1	Q96AP4	zinc finger containing ubiquitin peptidase 1
Malignant neoplasm of breast	NABP1	Q96AH0	nucleic acid binding protein 1
Malignant neoplasm of breast	LRRC59	Q96AG4	leucine rich repeat containing 59
Malignant neoplasm of breast	FUBP1	Q96AE4	far upstream element binding protein 1
Malignant neoplasm of breast	PNPLA2	Q96AD5	patatin like phospholipase domain containing 2
Malignant neoplasm of breast	FERMT2	Q96AC1	fermitin family member 2
Malignant neoplasm of breast	EMID1	Q96A84	EMI domain containing 1
Malignant neoplasm of breast	MAGOHB	Q96A72	mago homolog B, exon junction complex subunit
Malignant neoplasm of breast	AZIN2	Q96A70	antizyme inhibitor 2
Malignant neoplasm of breast	RERG	Q96A58	RAS like estrogen regulated growth inhibitor
Malignant neoplasm of breast	TP53INP1	Q96A56	tumor protein p53 inducible nuclear protein 1
Malignant neoplasm of breast	ADIPOR1	Q96A54	adiponectin receptor 1

Malignant neoplasm of breast	TSACC	Q96A04	TSSK6 activating cochaperone
Malignant neoplasm of breast	RILPL2	Q969X0	Rab interacting lysosomal protein like 2
Malignant neoplasm of breast	PMEPA1	Q969W9	prostate transmembrane protein, androgen induced 1
Malignant neoplasm of breast	MRTFA	Q969V6	myocardin related transcription factor A
Malignant neoplasm of breast	MUL1	Q969V5	mitochondrial E3 ubiquitin protein ligase 1
Malignant neoplasm of breast	WBP2	Q969T9	WW domain binding protein 2
Malignant neoplasm of breast	NEIL2	Q969S2	nei like DNA glycosylase 2
Malignant neoplasm of breast	ARL11	Q969Q4	ADP ribosylation factor like GTPase 11
Malignant neoplasm of breast	TRIM63	Q969Q1	tripartite motif containing 63
Malignant neoplasm of breast	TOP1MT	Q969P6	DNA topoisomerase I mitochondrial
Malignant neoplasm of breast	FBXO32	Q969P5	F-box protein 32
Malignant neoplasm of breast	PIGT	Q969N2	phosphatidylinositol glycan anchor biosynthesis class T
Malignant neoplasm of breast	MAL2	Q969L2	mal, T cell differentiation protein 2 (gene/pseudogene)
Malignant neoplasm of breast	TMEM54	Q969K7	transmembrane protein 54
Malignant neoplasm of breast	MYDGF	Q969H8	myeloid derived growth factor
Malignant neoplasm of breast	CNKSR1	Q969H4	connector enhancer of kinase suppressor of Ras 1
Malignant neoplasm of breast	FBXW7	Q969H0	F-box and WD repeat domain containing 7
Malignant neoplasm of breast	NKD1	Q969G9	NKD inhibitor of WNT signaling pathway 1
Malignant neoplasm of breast	CAVIN3	Q969G5	caveolae associated protein 3

Malignant neoplasm of breast	SMARCE1	Q969G3	SWI/SNF related, matrix associated, actin dependent regulator of chromatin, subfamily e, member 1
Malignant neoplasm of breast	KISS1R	Q969F8	KISS1 receptor
Malignant neoplasm of breast	NKD2	Q969F2	NKD inhibitor of WNT signaling pathway 2
Malignant neoplasm of breast	UCN3	Q969E3	urocortin 3
Malignant neoplasm of breast	TSLP	Q969D9	thymic stromal lymphopoietin
Malignant neoplasm of breast	MR1	Q95460	major histocompatibility complex, class I-related
Malignant neoplasm of breast	PHKB	Q93100	phosphorylase kinase regulatory subunit beta
Malignant neoplasm of breast	PTP4A1	Q93096	protein tyrosine phosphatase 4A1
Malignant neoplasm of breast	BHMT	Q93088	betaine--homocysteine S-methyltransferase
Malignant neoplasm of breast	P2RX5	Q93086	purinergic receptor P2X 5
Malignant neoplasm of breast	ATP2A3	Q93084	ATPase sarcoplasmic/endoplasmic reticulum Ca2+ transporting 3
Malignant neoplasm of breast	MT1L	Q93083	metallothionein 1L, pseudogene
Malignant neoplasm of breast	MED12	Q93074	mediator complex subunit 12
Malignant neoplasm of breast	LPP	Q93052	LIM domain containing preferred translocation partner in lipoma
Malignant neoplasm of breast	TNFRSF25	Q93038	TNF receptor superfamily member 25
Malignant neoplasm of breast	CUL5	Q93034	cullin 5
Malignant neoplasm of breast	USP7	Q93009	ubiquitin specific peptidase 7
Malignant neoplasm of breast	USP9X	Q93008	ubiquitin specific peptidase 9 X-linked
Malignant neoplasm of breast	DVL3	Q92997	dishevelled segment polarity protein 3

Malignant neoplasm of breast	USP13	Q92995	ubiquitin specific peptidase 13
Malignant neoplasm of breast	BRF1	Q92994	BRF1 RNA polymerase III transcription initiation factor subunit
Malignant neoplasm of breast	KAT5	Q92993	lysine acetyltransferase 5
Malignant neoplasm of breast	DLX4	Q92988	distal-less homeobox 4
Malignant neoplasm of breast	IRF7	Q92985	interferon regulatory factor 7
Malignant neoplasm of breast	ARHGEF2	Q92974	Rho/Rac guanine nucleotide exchange factor 2
Malignant neoplasm of breast	SLCO2A1	Q92959	solute carrier organic anion transporter family member 2A1
Malignant neoplasm of breast	TNFRSF14	Q92956	TNF receptor superfamily member 14
Malignant neoplasm of breast	PRG4	Q92954	proteoglycan 4
Malignant neoplasm of breast	FOXJ1	Q92949	forkhead box J1
Malignant neoplasm of breast	KHSRP	Q92945	KH-type splicing regulatory protein
Malignant neoplasm of breast	BAD	Q92934	BCL2 associated agonist of cell death
Malignant neoplasm of breast	PTPRN2	Q92932	protein tyrosine phosphatase receptor type N2
Malignant neoplasm of breast	SMARCC1	Q92922	SWI/SNF related, matrix associated, actin dependent regulator of chromatin subfamily c member 1
Malignant neoplasm of breast	MAP4K1	Q92918	mitogen-activated protein kinase kinase kinase kinase 1
Malignant neoplasm of breast	FGF13	Q92913	fibroblast growth factor 13
Malignant neoplasm of breast	SLC5A5	Q92911	solute carrier family 5 member 5
Malignant neoplasm of breast	GATA6	Q92908	GATA binding protein 6
Malignant neoplasm of breast	COPS5	Q92905	COP9 signalosome subunit 5

Malignant neoplasm of breast	CDS1	Q92903	CDP-diacylglycerol synthase 1
Malignant neoplasm of breast	HPS1	Q92902	HPS1 biogenesis of lysosomal organelles complex 3 subunit 1
Malignant neoplasm of breast	ERCC4	Q92889	ERCC excision repair 4, endonuclease catalytic subunit
Malignant neoplasm of breast	ARHGEF1	Q92888	Rho guanine nucleotide exchange factor 1
Malignant neoplasm of breast	ABCC2	Q92887	ATP binding cassette subfamily C member 2
Malignant neoplasm of breast	RAD50	Q92878	RAD50 double strand break repair protein
Malignant neoplasm of breast	KLK6	Q92876	kallikrein related peptidase 6
Malignant neoplasm of breast	NEO1	Q92859	neogenin 1
Malignant neoplasm of breast	SEMA4D	Q92854	semaphorin 4D
Malignant neoplasm of breast	CASP10	Q92851	caspase 10
Malignant neoplasm of breast	GHSR	Q92847	growth hormone secretagogue receptor
Malignant neoplasm of breast	TANK	Q92844	TRAF family member associated NFKB activator
Malignant neoplasm of breast	BCL2L2-PABPN1	Q92843	BCL2L2-PABPN1 readthrough
Malignant neoplasm of breast	BCL2L2	Q92843	BCL2 like 2
Malignant neoplasm of breast	DDX17	Q92841	DEAD-box helicase 17
Malignant neoplasm of breast	HAS1	Q92839	hyaluronan synthase 1
Malignant neoplasm of breast	EDA	Q92838	ectodysplasin A
Malignant neoplasm of breast	FRAT1	Q92837	FRAT regulator of WNT signaling pathway 1
Malignant neoplasm of breast	INPP5D	Q92835	inositol polyphosphate-5-phosphatase D

Malignant neoplasm of breast	NELL1	Q92832	neural EGFL like 1
Malignant neoplasm of breast	KAT2B	Q92831	lysine acetyltransferase 2B
Malignant neoplasm of breast	KAT2A	Q92830	lysine acetyltransferase 2A
Malignant neoplasm of breast	HOXB13	Q92826	homeobox B13
Malignant neoplasm of breast	PCSK5	Q92824	proprotein convertase subtilisin/kexin type 5
Malignant neoplasm of breast	NRCAM	Q92823	neuronal cell adhesion molecule
Malignant neoplasm of breast	GGH	Q92820	gamma-glutamyl hydrolase
Malignant neoplasm of breast	HAS2	Q92819	hyaluronan synthase 2
Malignant neoplasm of breast	GOLGA1	Q92805	golgin A1
Malignant neoplasm of breast	EZH1	Q92800	enhancer of zeste 1 polycomb repressive complex 2 subunit
Malignant neoplasm of breast	DLG3	Q92796	discs large MAGUK scaffold protein 3
Malignant neoplasm of breast	KAT6A	Q92794	lysine acetyltransferase 6A
Malignant neoplasm of breast	CREBBP	Q92793	CREB binding protein
Malignant neoplasm of breast	PROX1	Q92786	prospero homeobox 1
Malignant neoplasm of breast	DPF3	Q92784	double PHD fingers 3
Malignant neoplasm of breast	HDAC2	Q92769	histone deacetylase 2
Malignant neoplasm of breast	FRZB	Q92765	frizzled related protein
Malignant neoplasm of breast	GTF2H4	Q92759	general transcription factor IIH subunit 4
Malignant neoplasm of breast	TFAP2C	Q92754	transcription factor AP-2 gamma

Malignant neoplasm of breast	RORB	Q92753	RAR related orphan receptor B
Malignant neoplasm of breast	THRSP	Q92748	thyroid hormone responsive
Malignant neoplasm of breast	HTRA1	Q92743	HtrA serine peptidase 1
Malignant neoplasm of breast	USP6NL	Q92738	USP6 N-terminal like
Malignant neoplasm of breast	TFG	Q92734	trafficking from ER to golgi regulator
Malignant neoplasm of breast	ESR2	Q92731	estrogen receptor 2
Malignant neoplasm of breast	RND1	Q92730	Rho family GTPase 1
Malignant neoplasm of breast	PTPRU	Q92729	protein tyrosine phosphatase receptor type U
Malignant neoplasm of breast	RAD54L	Q92698	RAD54 like
Malignant neoplasm of breast	NECTIN2	Q92692	nectin cell adhesion molecule 2
Malignant neoplasm of breast	ANP32B	Q92688	acidic nuclear phosphoprotein 32 family member B
Malignant neoplasm of breast	ALG3	Q92685	ALG3 alpha-1,3- mannosyltransferase
Malignant neoplasm of breast	CENPI	Q92674	centromere protein I
Malignant neoplasm of breast	SORL1	Q92673	sortilin related receptor 1
Malignant neoplasm of breast	AKAP1	Q92667	A-kinase anchoring protein 1
Malignant neoplasm of breast	GTF3A	Q92664	general transcription factor IIIA
Malignant neoplasm of breast	FCGR1B	Q92637	Fc fragment of IgG receptor Ib
Malignant neoplasm of breast	LPAR1	Q92633	lysophosphatidic acid receptor 1
Malignant neoplasm of breast	DYRK2	Q92630	dual specificity tyrosine phosphorylation regulated kinase 2

Malignant neoplasm of breast	APPBP2	Q92624	amyloid beta precursor protein binding protein 2
Malignant neoplasm of breast	ZNF516	Q92618	zinc finger protein 516
Malignant neoplasm of breast	ZNF592	Q92610	zinc finger protein 592
Malignant neoplasm of breast	CNOT9	Q92600	CCR4-NOT transcription complex subunit 9
Malignant neoplasm of breast	HSPH1	Q92598	heat shock protein family H (Hsp110) member 1
Malignant neoplasm of breast	NDRG1	Q92597	N-myc downstream regulated 1
Malignant neoplasm of breast	MAML1	Q92585	mastermind like transcriptional coactivator 1
Malignant neoplasm of breast	CCL17	Q92583	C-C motif chemokine ligand 17
Malignant neoplasm of breast	TSC1	Q92574	TSC complex subunit 1
Malignant neoplasm of breast	NR4A3	Q92570	nuclear receptor subfamily 4 group A member 3
Malignant neoplasm of breast	PIK3R3	Q92569	phosphoinositide-3-kinase regulatory subunit 3
Malignant neoplasm of breast	FIG4	Q92562	FIG4 phosphoinositide 5-phosphatase
Malignant neoplasm of breast	BAP1	Q92560	BRCA1 associated protein 1
Malignant neoplasm of breast	WASF1	Q92558	WASP family member 1
Malignant neoplasm of breast	TOPBP1	Q92547	DNA topoisomerase II binding protein 1
Malignant neoplasm of breast	TM9SF4	Q92544	transmembrane 9 superfamily member 4
Malignant neoplasm of breast	NCSTN	Q92542	nicastrin
Malignant neoplasm of breast	SHC3	Q92529	SHC adaptor protein 3
Malignant neoplasm of breast	FAM3C	Q92520	FAM3 metabolism regulating signaling molecule C

Malignant neoplasm of breast	PIEZO1	Q92508	piezo type mechanosensitive ion channel component 1
Malignant neoplasm of breast	HSD17B8	Q92506	hydroxysteroid 17-beta dehydrogenase 8
Malignant neoplasm of breast	SLC39A7	Q92504	solute carrier family 39 member 7
Malignant neoplasm of breast	SEC14L1	Q92503	SEC14 like lipid binding 1
Malignant neoplasm of breast	STARD8	Q92502	StAR related lipid transfer domain containing 8
Malignant neoplasm of breast	DDX1	Q92499	DEAD-box helicase 1
Malignant neoplasm of breast	AQP3	Q92482	aquaporin 3 (Gill blood group)
Malignant neoplasm of breast	TFAP2B	Q92481	transcription factor AP-2 beta
Malignant neoplasm of breast	DDB2	Q92466	damage specific DNA binding protein 2
Malignant neoplasm of breast	ST8SIA4	Q92187	ST8 alpha-N-acetyl-neuraminide alpha-2,8-sialyltransferase 4
Malignant neoplasm of breast	ST8SIA2	Q92186	ST8 alpha-N-acetyl-neuraminide alpha-2,8-sialyltransferase 2
Malignant neoplasm of breast	ST8SIA1	Q92185	ST8 alpha-N-acetyl-neuraminide alpha-2,8-sialyltransferase 1
Malignant neoplasm of breast	TMEM158	Q8WZ71	transmembrane protein 158 (gene/pseudogene)
Malignant neoplasm of breast	TTN	Q8WZ42	titin
Malignant neoplasm of breast	LRTOMT	Q8WZ04;Q96E66	leucine rich transmembrane and O-methyltransferase domain containing
Malignant neoplasm of breast	ZCCHC14	Q8WYQ9	zinc finger CCHC-type containing 14
Malignant neoplasm of breast	DGCR8	Q8WYQ5	DGCR8 microprocessor complex subunit
Malignant neoplasm of breast	RIN2	Q8WYP3	Ras and Rab interactor 2
Malignant neoplasm of breast	ATG4A	Q8WYN0	autophagy related 4A cysteine peptidase

Malignant neoplasm of breast	SSH1	Q8WYL5	slingshot protein phosphatase 1
Malignant neoplasm of breast	ING5	Q8WYH8	inhibitor of growth family member 5
Malignant neoplasm of breast	KAT6B	Q8WYB5	lysine acetyltransferase 6B
Malignant neoplasm of breast	ARNTL2	Q8WYA1	aryl hydrocarbon receptor nuclear translocator like 2
Malignant neoplasm of breast	MYLIP	Q8WY64	myosin regulatory light chain interacting protein
Malignant neoplasm of breast	PPM1E	Q8WY54	protein phosphatase, Mg2+/Mn2+ dependent 1E
Malignant neoplasm of breast	NANOS1	Q8WY41	nanos C2HC-type zinc finger 1
Malignant neoplasm of breast	BRI3BP	Q8WY22	BRI3 binding protein
Malignant neoplasm of breast	SORCS1	Q8WY21	sortilin related VPS10 domain containing receptor 1
Malignant neoplasm of breast	PIBF1	Q8WXW3	progesterone immunomodulatory binding factor 1
Malignant neoplasm of breast	DNAAF4	Q8WXU2	dynein axonemal assembly factor 4
Malignant neoplasm of breast	GATAD2B	Q8WXI9	GATA zinc finger domain containing 2B
Malignant neoplasm of breast	CLEC4D	Q8WXI8	C-type lectin domain family 4 member D
Malignant neoplasm of breast	MUC16	Q8WXI7	mucin 16, cell surface associated
Malignant neoplasm of breast	CNKSR2	Q8WXI2	connector enhancer of kinase suppressor of Ras 2
Malignant neoplasm of breast	SYNE2	Q8WXH0	spectrin repeat containing nuclear envelope protein 2
Malignant neoplasm of breast	MADD	Q8WXG6	MAP kinase activating death domain
Malignant neoplasm of breast	HTR3C	Q8WXA8	5-hydroxytryptamine receptor 3C
Malignant neoplasm of breast	PALLD	Q8WX93	palladin, cytoskeletal associated protein

Malignant neoplasm of breast	NELFB	Q8WX92	negative elongation factor complex member B
Malignant neoplasm of breast	IGFBPL1	Q8WX77	insulin like growth factor binding protein like 1
Malignant neoplasm of breast	PRPF31	Q8WWY3	pre-mRNA processing factor 31
Malignant neoplasm of breast	HPSE2	Q8WWQ2	heparanase 2 (inactive)
Malignant neoplasm of breast	GIMAP1	Q8WWP7	GTPase, IMAP family member 1
Malignant neoplasm of breast	ARAP3	Q8WWN8	ArfGAP with RhoGAP domain, ankyrin repeat and PH domain 3
Malignant neoplasm of breast	CYGB	Q8WWM9	cytoglobin
Malignant neoplasm of breast	CKAP2	Q8WWK9	cytoskeleton associated protein 2
Malignant neoplasm of breast	NRG4	Q8WWG1	neuregulin 4
Malignant neoplasm of breast	ZFPM2	Q8WW38	zinc finger protein, FOG family member 2
Malignant neoplasm of breast	HMGB4	Q8WW32	high mobility group box 4
Malignant neoplasm of breast	TEKT4	Q8WW24	tektin 4
Malignant neoplasm of breast	UBLCP1	Q8WVY7	ubiquitin like domain containing CTD phosphatase 1
Malignant neoplasm of breast	C4orf3	Q8WVX3	chromosome 4 open reading frame 3
Malignant neoplasm of breast	TRAPPC12	Q8WVT3	trafficking protein particle complex 12
Malignant neoplasm of breast	TNFAIP8L1	Q8WVP5	TNF alpha induced protein 8 like 1
Malignant neoplasm of breast	UBE2Q2	Q8WVN8	ubiquitin conjugating enzyme E2 Q2
Malignant neoplasm of breast	STAG1	Q8WVM7	stromal antigen 1
Malignant neoplasm of breast	SKA2	Q8WVK7	spindle and kinetochore associated complex subunit 2

Malignant neoplasm of breast	TWIST2	Q8WVJ9	twist family bHLH transcription factor 2
Malignant neoplasm of breast	UCMA	Q8WVF2	upper zone of growth plate and cartilage matrix associated
Malignant neoplasm of breast	OSCP1	Q8WVF1	organic solute carrier partner 1
Malignant neoplasm of breast	PHLDA1	Q8WV24	pleckstrin homology like domain family A member 1
Malignant neoplasm of breast	DEPDC1B	Q8WUY9	DEP domain containing 1B
Malignant neoplasm of breast	SLC25A43	Q8WUT9	solute carrier family 25 member 43
Malignant neoplasm of breast	FBLIM1	Q8WUP2	filamin binding LIM protein 1
Malignant neoplasm of breast	SLC20A1	Q8WUM9	solute carrier family 20 member 1
Malignant neoplasm of breast	PDCD6IP	Q8WUM4	programmed cell death 6 interacting protein
Malignant neoplasm of breast	NUP133	Q8WUM0	nucleoporin 133
Malignant neoplasm of breast	CEMIP	Q8WUJ3	cell migration inducing hyaluronidase 1
Malignant neoplasm of breast	CYB5D2	Q8WUJ1	cytochrome b5 domain containing 2
Malignant neoplasm of breast	STYX	Q8WUJ0	serine/threonine/tyrosine interacting protein
Malignant neoplasm of breast	HDAC7	Q8WUI4	histone deacetylase 7
Malignant neoplasm of breast	PPP1R13L	Q8WUF5	protein phosphatase 1 regulatory subunit 13 like
Malignant neoplasm of breast	CT55	Q8WUE5	cancer/testis antigen 55
Malignant neoplasm of breast	CHPT1	Q8WUD6	choline phosphotransferase 1
Malignant neoplasm of breast	FRS2	Q8WU20	fibroblast growth factor receptor substrate 2
Malignant neoplasm of breast	NPRL2	Q8WTW4	NPR2 like, GATOR1 complex subunit

Malignant neoplasm of breast	SETD7	Q8WTS6	SET domain containing 7, histone lysine methyltransferase
Malignant neoplasm of breast	GDPD5	Q8WTR4	glycerophosphodiester phosphodiesterase domain containing 5
Malignant neoplasm of breast	GIPC2	Q8TF65	GIPC PDZ domain containing family member 2
Malignant neoplasm of breast	UBASH3B	Q8TF42	ubiquitin associated and SH3 domain containing B
Malignant neoplasm of breast	USP33	Q8TEY7	ubiquitin specific peptidase 33
Malignant neoplasm of breast	CREB3L4	Q8TEY5	cAMP responsive element binding protein 3 like 4
Malignant neoplasm of breast	PARD3	Q8TEW0	par-3 family cell polarity regulator
Malignant neoplasm of breast	RAPGEF6	Q8TEU7	Rap guanine nucleotide exchange factor 6
Malignant neoplasm of breast	DOT1L	Q8TEK3	DOT1 like histone lysine methyltransferase
Malignant neoplasm of breast	SLC37A2	Q8TED4	solute carrier family 37 member 2
Malignant neoplasm of breast	RHBDD1	Q8TEB9	rhomboid domain containing 1
Malignant neoplasm of breast	NSUN6	Q8TEA1	NOP2/Sun RNA methyltransferase 6
Malignant neoplasm of breast	MORC4	Q8TE76	MORC family CW-type zinc finger 4
Malignant neoplasm of breast	ADAMTS18	Q8TE60	ADAM metallopeptidase with thrombospondin type 1 motif 18
Malignant neoplasm of breast	ADAMTS19	Q8TE59	ADAM metallopeptidase with thrombospondin type 1 motif 19
Malignant neoplasm of breast	ADAMTS17	Q8TE56	ADAM metallopeptidase with thrombospondin type 1 motif 17
Malignant neoplasm of breast	MICAL1	Q8TDZ2	microtubule associated monooxygenase, calponin and LIM domain containing 1
Malignant neoplasm of breast	ASAP3	Q8TDY4	ArfGAP with SH3 domain, ankyrin repeat and PH domain 3
Malignant neoplasm of breast	RB1CC1	Q8TDY2	RB1 inducible coiled-coil 1

Malignant neoplasm of breast	CSGALNACT1	Q8TDX6	chondroitin sulfate N-acetylgalactosaminyltransferase 1
Malignant neoplasm of breast	ST7L	Q8TDW4	suppression of tumorigenicity 7 like
Malignant neoplasm of breast	GPR119	Q8TDV5	G protein-coupled receptor 119
Malignant neoplasm of breast	GPR151	Q8TDV0	G protein-coupled receptor 151
Malignant neoplasm of breast	GPBAR1	Q8TDU6	G protein-coupled bile acid receptor 1
Malignant neoplasm of breast	VN1R17P	Q8TDU5	vomeronasal 1 receptor 17 pseudogene
Malignant neoplasm of breast	OXER1	Q8TDS5	oxoeicosanoid receptor 1
Malignant neoplasm of breast	HCAR2	Q8TDS4	hydroxycarboxylic acid receptor 2
Malignant neoplasm of breast	HAVCR2	Q8TDQ0	hepatitis A virus cellular receptor 2
Malignant neoplasm of breast	RNASEH2C	Q8TDP1	ribonuclease H2 subunit C
Malignant neoplasm of breast	DLG5	Q8TDM6	discs large MAGUK scaffold protein 5
Malignant neoplasm of breast	BCAS4	Q8TDM0	breast carcinoma amplified sequence 4
Malignant neoplasm of breast	DMXL2	Q8TDJ6	Dmx like 2
Malignant neoplasm of breast	CHD5	Q8TDI0	chromodomain helicase DNA binding protein 5
Malignant neoplasm of breast	HELQ	Q8TDG4	helicase, POLQ like
Malignant neoplasm of breast	ACTRT1	Q8TDG2	actin related protein T1
Malignant neoplasm of breast	DDX54	Q8TDD1	DEAD-box helicase 54
Malignant neoplasm of breast	BRSK1	Q8TDC3	BR serine/threonine kinase 1
Malignant neoplasm of breast	DTX3L	Q8TDB6	deltex E3 ubiquitin ligase 3L

Malignant neoplasm of breast	MAGEE2	Q8TD90	MAGE family member E2
Malignant neoplasm of breast	CALML6	Q8TD86	calmodulin like 6
Malignant neoplasm of breast	CD200R1	Q8TD46	CD200 receptor 1
Malignant neoplasm of breast	CCHCR1	Q8TD31	coiled-coil alpha-helical rod protein 1
Malignant neoplasm of breast	GPT2	Q8TD30	glutamic--pyruvic transaminase 2
Malignant neoplasm of breast	SLC2A12	Q8TD20	solute carrier family 2 member 12
Malignant neoplasm of breast	ZNF398	Q8TD17	zinc finger protein 398
Malignant neoplasm of breast	MAPK15	Q8TD08	mitogen-activated protein kinase 15
Malignant neoplasm of breast	AGR3	Q8TD06	anterior gradient 3, protein disulphide isomerase family member
Malignant neoplasm of breast	RHPN1	Q8TCX5	rhophilin Rho GTPase binding protein 1
Malignant neoplasm of breast	PREX1	Q8TCU6	phosphatidylinositol-3,4,5-trisphosphate dependent Rac exchange factor 1
Malignant neoplasm of breast	HM13	Q8TCT9	histocompatibility minor 13
Malignant neoplasm of breast	SPPL3	Q8TCT6	signal peptide peptidase like 3
Malignant neoplasm of breast	CERK	Q8TCT0	ceramide kinase
Malignant neoplasm of breast	PIFO	Q8TCI5	primary cilia formation
Malignant neoplasm of breast	CPT1C	Q8TCG5	carnitine palmitoyltransferase 1C
Malignant neoplasm of breast	CIP2A	Q8TCG1	cellular inhibitor of PP2A
Malignant neoplasm of breast	METTL6	Q8TCB7	methyltransferase like 6
Malignant neoplasm of breast	IFI44	Q8TCB0	interferon induced protein 44

Malignant neoplasm of breast	SPATA18	Q8TC71	spermatogenesis associated 18
Malignant neoplasm of breast	PIWIL2	Q8TC59	piwi like RNA-mediated gene silencing 2
Malignant neoplasm of breast	TRMT10A	Q8TBZ6	tRNA methyltransferase 10A
Malignant neoplasm of breast	WDR20	Q8TBZ3	WD repeat domain 20
Malignant neoplasm of breast	MYCBPAP	Q8TBZ2	MYCBP associated protein
Malignant neoplasm of breast	PIP4K2C	Q8TBX8	phosphatidylinositol-5-phosphate 4-kinase type 2 gamma
Malignant neoplasm of breast	TBC1D16	Q8TBP0	TBC1 domain family member 16
Malignant neoplasm of breast	SETD6	Q8TBK2	SET domain containing 6, protein lysine methyltransferase
Malignant neoplasm of breast	SYNPR	Q8TBG9	synaptoporin
Malignant neoplasm of breast	PIGX	Q8TBF5	phosphatidylinositol glycan anchor biosynthesis class X
Malignant neoplasm of breast	ITFG1	Q8TB96	integrin alpha FG-GAP repeat containing 1
Malignant neoplasm of breast	PUM2	Q8TB72	pumilio RNA binding family member 2
Malignant neoplasm of breast	SLC35B2	Q8TB61	solute carrier family 35 member B2
Malignant neoplasm of breast	DEPTOR	Q8TB45	DEP domain containing MTOR interacting protein
Malignant neoplasm of breast	GSDMB	Q8TAX9	gasdermin B
Malignant neoplasm of breast	CYP2W1	Q8TAV3	cytochrome P450 family 2 subfamily W member 1
Malignant neoplasm of breast	UHMK1	Q8TAS1	U2AF homology motif kinase 1
Malignant neoplasm of breast	MPLKIP	Q8TAP9	M-phase specific PLK1 interacting protein
Malignant neoplasm of breast	WDR48	Q8TAF3	WD repeat domain 48

Malignant neoplasm of breast	PPP1R14C	Q8TAE6	protein phosphatase 1 regulatory inhibitor subunit 14C
Malignant neoplasm of breast	C12orf75	Q8TAD7	chromosome 12 open reading frame 75
Malignant neoplasm of breast	IL17D	Q8TAD2	interleukin 17D
Malignant neoplasm of breast	VANGL1	Q8TAA9	VANGL planar cell polarity protein 1
Malignant neoplasm of breast	KIF18A	Q8NI77	kinesin family member 18A
Malignant neoplasm of breast	CTCFL	Q8NI51	CCCTC-binding factor like
Malignant neoplasm of breast	NCOA7	Q8NI08	nuclear receptor coactivator 7
Malignant neoplasm of breast	COP1	Q8NHY2	COP1 E3 ubiquitin ligase
Malignant neoplasm of breast	RHOXF1	Q8NHV9	Rhox homeobox family member 1
Malignant neoplasm of breast	SGMS2	Q8NHU3	sphingomyelin synthase 2
Malignant neoplasm of breast	RASSF8	Q8NHQ8	Ras association domain family member 8
Malignant neoplasm of breast	CEP70	Q8NHQ1	centrosomal protein 70
Malignant neoplasm of breast	MOSPD2	Q8NHP6	motile sperm domain containing 2
Malignant neoplasm of breast	KDM2B	Q8NHM5	lysine demethylase 2B
Malignant neoplasm of breast	PRSS3P2	Q8NHM4	PRSS3 pseudogene 2
Malignant neoplasm of breast	LILRB1	Q8NHL6	leukocyte immunoglobulin like receptor B1
Malignant neoplasm of breast	KIR2DL5B	Q8NHK3	killer cell immunoglobulin like receptor, two Ig domains and long cytoplasmic tail 5B
Malignant neoplasm of breast	OR2T6	Q8NHC8	olfactory receptor family 2 subfamily T member 6
Malignant neoplasm of breast	OR1N1	Q8NGS0	olfactory receptor family 1 subfamily N member 1

Malignant neoplasm of breast	RDM1	Q8NG50	RAD52 motif containing 1
Malignant neoplasm of breast	CADM4	Q8NFZ8	cell adhesion molecule 4
Malignant neoplasm of breast	FBH1	Q8NFZ0	F-box DNA helicase 1
Malignant neoplasm of breast	KBTBD8	Q8NFY9	kelch repeat and BTB domain containing 8
Malignant neoplasm of breast	TET1	Q8NFU7	tet methylcytosine dioxygenase 1
Malignant neoplasm of breast	DNER	Q8NFT8	delta/notch like EGF repeat containing
Malignant neoplasm of breast	STEAP2	Q8NFT2	STEAP2 metalloreductase
Malignant neoplasm of breast	ADCY4	Q8NFM4	adenylate cyclase 4
Malignant neoplasm of breast	BHLHE22	Q8NFJ8	basic helix-loop-helix family member e22
Malignant neoplasm of breast	GPRC5A	Q8NFJ5	G protein-coupled receptor class C group 5 member A
Malignant neoplasm of breast	ENGASE	Q8NFI3	endo-beta-N-acetylglucosaminidase
Malignant neoplasm of breast	REPS2	Q8NFH8	RALBP1 associated Eps domain containing 2
Malignant neoplasm of breast	NUP43	Q8NFH3	nucleoporin 43
Malignant neoplasm of breast	FLCN	Q8NFG4	folliculin
Malignant neoplasm of breast	ARID1B	Q8NFD5	AT-rich interaction domain 1B
Malignant neoplasm of breast	ANKK1	Q8NFD2	ankyrin repeat and kinase domain containing 1
Malignant neoplasm of breast	BOD1L1	Q8NFC6	biorientation of chromosomes in cell division 1 like 1
Malignant neoplasm of breast	USP32	Q8NFA0	ubiquitin specific peptidase 32
Malignant neoplasm of breast	SYNE1	Q8NF91	spectrin repeat containing nuclear envelope protein 1

Malignant neoplasm of breast	DOCK8	Q8NF50	dedicator of cytokinesis 8
Malignant neoplasm of breast	LPCAT1	Q8NF37	lysophosphatidylcholine acyltransferase 1
Malignant neoplasm of breast	FBXO22	Q8NEZ5	F-box protein 22
Malignant neoplasm of breast	KMT2C	Q8NEZ4	lysine methyltransferase 2C
Malignant neoplasm of breast	VPS37A	Q8NEZ2	VPS37A subunit of ESCRT-I
Malignant neoplasm of breast	PPHLN1	Q8NEY8	periphilin 1
Malignant neoplasm of breast	NAV1	Q8NEY1	neuron navigator 1
Malignant neoplasm of breast	IL27	Q8NEV9	interleukin 27
Malignant neoplasm of breast	APPL2	Q8NEU8	adaptor protein, phosphotyrosine interacting with PH domain and leucine zipper 2
Malignant neoplasm of breast	ACVR1C	Q8NER5	activin A receptor type 1C
Malignant neoplasm of breast	TRPV1	Q8NER1	transient receptor potential cation channel subfamily V member 1
Malignant neoplasm of breast	SRARP	Q8NEQ6	steroid receptor associated and regulated protein
Malignant neoplasm of breast	SUPT20H	Q8NEM7	SPT20 homolog, SAGA complex component
Malignant neoplasm of breast	SHCBP1	Q8NEM2	SHC binding and spindle associated 1
Malignant neoplasm of breast	MCPH1	Q8NEM0	microcephalin 1
Malignant neoplasm of breast	CCDC54	Q8NEL0	coiled-coil domain containing 54
Malignant neoplasm of breast	NGDN	Q8NEJ9	neuroguidin
Malignant neoplasm of breast	FAM83F	Q8NEG4	family with sequence similarity 83 member F
Malignant neoplasm of breast	PIK3C3	Q8NEB9	phosphatidylinositol 3-kinase catalytic subunit type 3

Malignant neoplasm of breast	PLPP5	Q8NEB5	phospholipid phosphatase 5
Malignant neoplasm of breast	MCU	Q8NE86	mitochondrial calcium uniporter
Malignant neoplasm of breast	BVES	Q8NE79	blood vessel epicardial substance
Malignant neoplasm of breast	CHDH	Q8NE62	choline dehydrogenase
Malignant neoplasm of breast	SETD9	Q8NE22	SET domain containing 9
Malignant neoplasm of breast	BHLHE23	Q8NDY6	basic helix-loop-helix family member e23
Malignant neoplasm of breast	PSD4	Q8NDX1	pleckstrin and Sec7 domain containing 4
Malignant neoplasm of breast	TNRC6A	Q8NDV7	trinucleotide repeat containing adaptor 6A
Malignant neoplasm of breast	EHBP1	Q8NDI1	EH domain binding protein 1
Malignant neoplasm of breast	NPEPL1	Q8NDH3	aminopeptidase like 1
Malignant neoplasm of breast	TDRD9	Q8NDG6	tudor domain containing 9
Malignant neoplasm of breast	CARMIL3	Q8ND23	capping protein regulator and myosin 1 linker 3
Malignant neoplasm of breast	C11orf65	Q8NCR3	chromosome 11 open reading frame 65
Malignant neoplasm of breast	B3GALNT2	Q8NCR0	beta-1,3-N-acetylgalactosaminyltransferase 2
Malignant neoplasm of breast	RNF169	Q8NCN4	ring finger protein 169
Malignant neoplasm of breast	DYNC2H1	Q8NCM8	dynein cytoplasmic 2 heavy chain 1
Malignant neoplasm of breast	GALNT6	Q8NCL4	polypeptide N-acetylgalactosaminyltransferase 6
Malignant neoplasm of breast	HJURP	Q8NCD3	Holliday junction recognition protein
Malignant neoplasm of breast	PLA2G15	Q8NCC3	phospholipase A2 group XV

Malignant neoplasm of breast	NOA1	Q8NC60	nitric oxide associated 1
Malignant neoplasm of breast	RNF149	Q8NC42	ring finger protein 149
Malignant neoplasm of breast	ATAD1	Q8NBU5	ATPase family AAA domain containing 1
Malignant neoplasm of breast	SPC24	Q8NBT2	SPC24 component of NDC80 kinetochore complex
Malignant neoplasm of breast	POC1A	Q8NBT0	POC1 centriolar protein A
Malignant neoplasm of breast	TXNDC5	Q8NBS9	thioredoxin domain containing 5
Malignant neoplasm of breast	SLC4A11	Q8NBS3	solute carrier family 4 member 11
Malignant neoplasm of breast	PCSK9	Q8NBP7	proprotein convertase subtilisin/kexin type 9
Malignant neoplasm of breast	MFSD9	Q8NBP5	major facilitator superfamily domain containing 9
Malignant neoplasm of breast	SUMF1	Q8NBK3	sulfatase modifying factor 1
Malignant neoplasm of breast	GOLM1	Q8NBJ4	golgi membrane protein 1
Malignant neoplasm of breast	FANCB	Q8NB91	FA complementation group B
Malignant neoplasm of breast	KDM1B	Q8NB78	lysine demethylase 1B
Malignant neoplasm of breast	MLKL	Q8NB16	mixed lineage kinase domain like pseudokinase
Malignant neoplasm of breast	USP38	Q8NB14	ubiquitin specific peptidase 38
Malignant neoplasm of breast	ANKLE1	Q8NAG6	ankyrin repeat and LEM domain containing 1
Malignant neoplasm of breast	IQUB	Q8NA54	IQ motif and ubiquitin domain containing
Malignant neoplasm of breast	L3MBTL4	Q8NA19	L3MBTL histone methyl-lysine binding protein 4
Malignant neoplasm of breast	FSIP1	Q8NA03	fibrous sheath interacting protein 1

Malignant neoplasm of breast	TRIML1	Q8N9V2	tripartite motif family like 1
Malignant neoplasm of breast	TC2N	Q8N9U0	tandem C2 domains, nuclear
Malignant neoplasm of breast	SCAI	Q8N9R8	suppressor of cancer cell invasion
Malignant neoplasm of breast	BANP	Q8N9N5	BTG3 associated nuclear protein
Malignant neoplasm of breast	ASCC1	Q8N9N2	activating signal cointegrator 1 complex subunit 1
Malignant neoplasm of breast	DTX3	Q8N9I9	deltex E3 ubiquitin ligase 3
Malignant neoplasm of breast	CDYL2	Q8N8U2	chromodomain Y like 2
Malignant neoplasm of breast	ENAH	Q8N8S7	ENAH actin regulator
Malignant neoplasm of breast	ANKRD44	Q8N8A2	ankyrin repeat domain 44
Malignant neoplasm of breast	ZNF366	Q8N895	zinc finger protein 366
Malignant neoplasm of breast	TSPYL6	Q8N831	TSPY like 6
Malignant neoplasm of breast	STPG4	Q8N801	sperm-tail PG-rich repeat containing 4
Malignant neoplasm of breast	BEND7	Q8N7W2	BEN domain containing 7
Malignant neoplasm of breast	PAF1	Q8N7H5	PAF1 homolog, Paf1/RNA polymerase II complex component
Malignant neoplasm of breast	CBLL2	Q8N7E2	Cbl proto-oncogene like 2
Malignant neoplasm of breast	PCDH20	Q8N6Y1	protocadherin 20
Malignant neoplasm of breast	USHBP1	Q8N6Y0	USH1 protein network component harmonin binding protein 1
Malignant neoplasm of breast	GPR161	Q8N6U8	G protein-coupled receptor 161
Malignant neoplasm of breast	SIRT6	Q8N6T7	sirtuin 6

Malignant neoplasm of breast	EEF1AKNMT	Q8N6R0	eEF1A lysine and N-terminal methyltransferase
Malignant neoplasm of breast	CD177	Q8N6Q3	CD177 molecule
Malignant neoplasm of breast	C1orf52	Q8N6N3	chromosome 1 open reading frame 52
Malignant neoplasm of breast	ANKRD29	Q8N6D5	ankyrin repeat domain 29
Malignant neoplasm of breast	RNF182	Q8N6D2	ring finger protein 182
Malignant neoplasm of breast	MIR7-3HG	Q8N6C7	MIR7-3 host gene
Malignant neoplasm of breast	IGSF1	Q8N6C5	immunoglobulin superfamily member 1
Malignant neoplasm of breast	SLC5A8	Q8N695	solute carrier family 5 member 8
Malignant neoplasm of breast	CPSF7	Q8N684	cleavage and polyadenylation specific factor 7
Malignant neoplasm of breast	DRAM1	Q8N682	DNA damage regulated autophagy modulator 1
Malignant neoplasm of breast	EIF4E3	Q8N5X7	eukaryotic translation initiation factor 4E family member 3
Malignant neoplasm of breast	SLC25A41	Q8N5S1	solute carrier family 25 member 41
Malignant neoplasm of breast	CISD2	Q8N5K1	CDGSH iron sulfur domain 2
Malignant neoplasm of breast	SH2D3C	Q8N5H7	SH2 domain containing 3C
Malignant neoplasm of breast	NKAP	Q8N5F7	NFKB activating protein
Malignant neoplasm of breast	AFAP1	Q8N556	actin filament associated protein 1
Malignant neoplasm of breast	ZNF276	Q8N554	zinc finger protein 276
Malignant neoplasm of breast	OGFOD1	Q8N543	2-oxoglutarate and iron dependent oxygenase domain containing 1
Malignant neoplasm of breast	TMEM199	Q8N511	transmembrane protein 199

Malignant neoplasm of breast	AFAP1L2	Q8N4X5	actin filament associated protein 1 like 2
Malignant neoplasm of breast	PAQR4	Q8N4S7	progestin and adipoQ receptor family member 4
Malignant neoplasm of breast	HDDC3	Q8N4P3	HD domain containing 3
Malignant neoplasm of breast	NIN	Q8N4C6	ninein
Malignant neoplasm of breast	FBXO39	Q8N4B4	F-box protein 39
Malignant neoplasm of breast	GALNT4	Q8N4A0	polypeptide N-acetylgalactosaminyltransferase 4
Malignant neoplasm of breast	PNKD	Q8N490	PNKD metallo-beta-lactamase domain containing
Malignant neoplasm of breast	RYBP	Q8N488	RING1 and YY1 binding protein
Malignant neoplasm of breast	SFRP1	Q8N474	secreted frizzled related protein 1
Malignant neoplasm of breast	FGFRL1	Q8N441	fibroblast growth factor receptor like 1
Malignant neoplasm of breast	GALNT16	Q8N428	polypeptide N-acetylgalactosaminyltransferase 16
Malignant neoplasm of breast	NME8	Q8N427	NME/NM23 family member 8
Malignant neoplasm of breast	FBXW8	Q8N3Y1	F-box and WD repeat domain containing 8
Malignant neoplasm of breast	SYNPO	Q8N3V7	synaptopodin
Malignant neoplasm of breast	TMEM132C	Q8N3T6	transmembrane protein 132C
Malignant neoplasm of breast	MICALL1	Q8N3F8	MICAL like 1
Malignant neoplasm of breast	ARHGAP18	Q8N392	Rho GTPase activating protein 18
Malignant neoplasm of breast	MUC15	Q8N387	mucin 15, cell surface associated
Malignant neoplasm of breast	KDM8	Q8N371	lysine demethylase 8

Malignant neoplasm of breast	MT1M	Q8N339	metallothionein 1M
Malignant neoplasm of breast	PGBD3	Q8N328	piggyBac transposable element derived 3
Malignant neoplasm of breast	LTBP4	Q8N2S1	latent transforming growth factor beta binding protein 4
Malignant neoplasm of breast	ANKRD36B	Q8N2N9	ankyrin repeat domain 36B
Malignant neoplasm of breast	CLASRP	Q8N2M8	CLK4 associating serine/arginine rich protein
Malignant neoplasm of breast	ARHGAP24	Q8N264	Rho GTPase activating protein 24
Malignant neoplasm of breast	ARHGEF28	Q8N1W1	Rho guanine nucleotide exchange factor 28
Malignant neoplasm of breast	LINC01561	Q8N1V8	long intergenic non-protein coding RNA 1561
Malignant neoplasm of breast	CA13	Q8N1Q1	carbonic anhydrase 13
Malignant neoplasm of breast	CYP4Z2P	Q8N1L4	cytochrome P450 family 4 subfamily Z member 2, pseudogene
Malignant neoplasm of breast	DOCK4	Q8N1I0	dedicator of cytokinesis 4
Malignant neoplasm of breast	CMTR1	Q8N1G2	cap methyltransferase 1
Malignant neoplasm of breast	FBXL14	Q8N1E6	F-box and leucine rich repeat protein 14
Malignant neoplasm of breast	VPS52	Q8N1B4	VPS52 subunit of GARP complex
Malignant neoplasm of breast	PDIK1L	Q8N165	PDLIM1 interacting kinase 1 like
Malignant neoplasm of breast	CCAR2	Q8N163	cell cycle and apoptosis regulator 2
Malignant neoplasm of breast	GPC2	Q8N158	glypican 2
Malignant neoplasm of breast	ZFP82	Q8N141	ZFP82 zinc finger protein
Malignant neoplasm of breast	EID3	Q8N140	EP300 interacting inhibitor of differentiation 3

Malignant neoplasm of breast	ORMDL3	Q8N138	ORMDL sphingolipid biosynthesis regulator 3
Malignant neoplasm of breast	CNTROB	Q8N137	centrobin, centriole duplication and spindle assembly protein
Malignant neoplasm of breast	DAW1	Q8N136	dynein assembly factor with WD repeats 1
Malignant neoplasm of breast	RPTOR	Q8N122	regulatory associated protein of MTOR complex 1
Malignant neoplasm of breast	MMP21	Q8N119	matrix metallopeptidase 21
Malignant neoplasm of breast	MIER1	Q8N108	MIER1 transcriptional regulator
Malignant neoplasm of breast	NLGN4X	Q8N0W4	neuroligin 4 X-linked
Malignant neoplasm of breast	GCNT2	Q8N0V5	glucosaminyl (N-acetyl) transferase 2 (I blood group)
Malignant neoplasm of breast	C1orf87	Q8N0U7	chromosome 1 open reading frame 87
Malignant neoplasm of breast	LINC00518	Q8N0U6	long intergenic non-protein coding RNA 518
Malignant neoplasm of breast	APCDD1	Q8J025	APC down-regulated 1
Malignant neoplasm of breast	BLID	Q8IZY5	BH3-like motif containing, cell death inducer
Malignant neoplasm of breast	ASPM	Q8IZT6	abnormal spindle microtubule assembly
Malignant neoplasm of breast	CACNA2D3	Q8IZS8	calcium voltage-gated channel auxiliary subunit alpha2delta 3
Malignant neoplasm of breast	MYOCD	Q8IZQ8	myocardin
Malignant neoplasm of breast	HS6ST3	Q8IZP7	heparan sulfate 6-O-sulfotransferase 3
Malignant neoplasm of breast	ABI1	Q8IZP0	abl interactor 1
Malignant neoplasm of breast	SLC38A6	Q8IZM9	solute carrier family 38 member 6
Malignant neoplasm of breast	ZNF654	Q8IZM8	zinc finger protein 654

Malignant neoplasm of breast	PELP1	Q8IZL8	proline, glutamate and leucine rich protein 1
Malignant neoplasm of breast	ADGRF4	Q8IZF3	adhesion G protein-coupled receptor F4
Malignant neoplasm of breast	ADGRF5	Q8IZF2	adhesion G protein-coupled receptor F5
Malignant neoplasm of breast	DOCK3	Q8IZD9	dedicator of cytokinesis 3
Malignant neoplasm of breast	CMTM1	Q8IZ96	CKLF like MARVEL transmembrane domain containing 1
Malignant neoplasm of breast	TRMT2A	Q8IZ69	tRNA methyltransferase 2 homolog A
Malignant neoplasm of breast	NRSN1	Q8IZ57	neurensin 1
Malignant neoplasm of breast	DND1	Q8IYX4	DND microRNA-mediated repression inhibitor 1
Malignant neoplasm of breast	CCDC116	Q8IYX3	coiled-coil domain containing 116
Malignant neoplasm of breast	RNF168	Q8IYW5	ring finger protein 168
Malignant neoplasm of breast	HACE1	Q8IYU2	HECT domain and ankyrin repeat containing E3 ubiquitin protein ligase 1
Malignant neoplasm of breast	CCDC170	Q8IYT3	coiled-coil domain containing 170
Malignant neoplasm of breast	CMTR2	Q8IYT2	cap methyltransferase 2
Malignant neoplasm of breast	TMEFF1	Q8IYR6	transmembrane protein with EGF like and two follistatin like domains 1
Malignant neoplasm of breast	SMYD4	Q8IYR2	SET and MYND domain containing 4
Malignant neoplasm of breast	TRIM22	Q8IYM9	tripartite motif containing 22
Malignant neoplasm of breast	COLGALT2	Q8IYK4	collagen beta(1-O)galactosyltransferase 2
Malignant neoplasm of breast	SHLD1	Q8IYI0	shieldin complex subunit 1
Malignant neoplasm of breast	FANCM	Q8IYD8	FA complementation group M

Malignant neoplasm of breast	SLX4	Q8IY92	SLX4 structure-specific endonuclease subunit
Malignant neoplasm of breast	EFCAB13	Q8IY85	EF-hand calcium binding domain 13
Malignant neoplasm of breast	AMOTL1	Q8IY63	angiomotin like 1
Malignant neoplasm of breast	DDIAS	Q8IXT1	DNA damage induced apoptosis suppressor
Malignant neoplasm of breast	PARP9	Q8IXQ6	poly(ADP-ribose) polymerase family member 9
Malignant neoplasm of breast	KLHL7	Q8IXQ5	kelch like family member 7
Malignant neoplasm of breast	NRM	Q8IXM6	nurim
Malignant neoplasm of breast	MRPL41	Q8IXM3	mitochondrial ribosomal protein L41
Malignant neoplasm of breast	MSRB3	Q8IXL7	methionine sulfoxide reductase B3
Malignant neoplasm of breast	ASXL1	Q8IXJ9	ASXL transcriptional regulator 1
Malignant neoplasm of breast	SIRT2	Q8IXJ6	sirtuin 2
Malignant neoplasm of breast	TP53INP2	Q8IXH6	tumor protein p53 inducible nuclear protein 2
Malignant neoplasm of breast	TRARG1	Q8IXB3	trafficking regulator of GLUT4 (SLC2A4) 1
Malignant neoplasm of breast	DNAJC10	Q8IXB1	DnaJ heat shock protein family (Hsp40) member C10
Malignant neoplasm of breast	SKA3	Q8IX90	spindle and kinetochore associated complex subunit 3
Malignant neoplasm of breast	CCAR1	Q8IX12	cell division cycle and apoptosis regulator 1
Malignant neoplasm of breast	WWC1	Q8IX03	WW and C2 domain containing 1
Malignant neoplasm of breast	ANKHD1-EIF4EBP3	Q8IWZ3	ANKHD1-EIF4EBP3 readthrough
Malignant neoplasm of breast	ANKHD1	Q8IWZ3	ankyrin repeat and KH domain containing 1

Malignant neoplasm of breast	UNC45B	Q8IWX7	unc-45 myosin chaperone B
Malignant neoplasm of breast	ADHFE1	Q8IWW8	alcohol dehydrogenase iron containing 1
Malignant neoplasm of breast	CNTN4	Q8IWV2	contactin 4
Malignant neoplasm of breast	SULF1	Q8IWU6	sulfatase 1
Malignant neoplasm of breast	SULF2	Q8IWU5	sulfatase 2
Malignant neoplasm of breast	CUL9	Q8IWT3	cullin 9
Malignant neoplasm of breast	SCN4B	Q8IWT1	sodium voltage-gated channel beta subunit 4
Malignant neoplasm of breast	TRIM59	Q8IWR1	tripartite motif containing 59
Malignant neoplasm of breast	BRSK2	Q8IWQ3	BR serine/threonine kinase 2
Malignant neoplasm of breast	MGA	Q8IWI9	MAX dimerization protein MGA
Malignant neoplasm of breast	TEX14	Q8IWB6	testis expressed 14, intercellular bridge forming factor
Malignant neoplasm of breast	ARID3B	Q8IVW6	AT-rich interaction domain 3B
Malignant neoplasm of breast	IGDCC3	Q8IVU1	immunoglobulin superfamily DCC subclass member 3
Malignant neoplasm of breast	KSR1	Q8IVT5	kinase suppressor of ras 1
Malignant neoplasm of breast	MCAT	Q8IVS2	malonyl-CoA-acyl carrier protein transacylase
Malignant neoplasm of breast	FUNDC1	Q8IVP5	FUN14 domain containing 1
Malignant neoplasm of breast	MUSTN1	Q8IVN3	musculoskeletal, embryonic nuclear protein 1
Malignant neoplasm of breast	SLC22A9	Q8IVM8	solute carrier family 22 member 9
Malignant neoplasm of breast	P3H3	Q8IVL6	prolyl 3-hydroxylase 3

Malignant neoplasm of breast	P3H2	Q8IVL5	prolyl 3-hydroxylase 2
Malignant neoplasm of breast	C3orf35	Q8IVJ8	chromosome 3 open reading frame 35
Malignant neoplasm of breast	FOXP4	Q8IVH2	forkhead box P4
Malignant neoplasm of breast	RASGRP3	Q8IV61	RAS guanyl releasing protein 3
Malignant neoplasm of breast	LRRC49	Q8IUZ0	leucine rich repeat containing 49
Malignant neoplasm of breast	EGFL6	Q8IUX8	EGF like domain multiple 6
Malignant neoplasm of breast	AEBP1	Q8IUX7	AE binding protein 1
Malignant neoplasm of breast	ARMC8	Q8IUR7	armadillo repeat containing 8
Malignant neoplasm of breast	SIAH1	Q8IUQ4	siah E3 ubiquitin protein ligase 1
Malignant neoplasm of breast	CLEC10A	Q8IUN9	C-type lectin domain containing 10A
Malignant neoplasm of breast	NPAS4	Q8IUM7	neuronal PAS domain protein 4
Malignant neoplasm of breast	RBM45	Q8IUH3	RNA binding motif protein 45
Malignant neoplasm of breast	RIOX2	Q8IUF8	ribosomal oxygenase 2
Malignant neoplasm of breast	H2AC21	Q8IUE6	H2A clustered histone 21
Malignant neoplasm of breast	TICAM1	Q8IUC6	toll like receptor adaptor molecule 1
Malignant neoplasm of breast	CAMK1D	Q8IU85	calcium/calmodulin dependent protein kinase ID
Malignant neoplasm of breast	TMPRSS6	Q8IU80	transmembrane serine protease 6
Malignant neoplasm of breast	IFNLR1	Q8IU57	interferon lambda receptor 1
Malignant neoplasm of breast	KLB	Q86Z14	klotho beta

Malignant neoplasm of breast	HRNR	Q86YZ3	hornerin
Malignant neoplasm of breast	TREML1	Q86YW5	triggering receptor expressed on myeloid cells like 1
Malignant neoplasm of breast	MIB1	Q86YT6	mindbomb E3 ubiquitin protein ligase 1
Malignant neoplasm of breast	SLC13A5	Q86YT5	solute carrier family 13 member 5
Malignant neoplasm of breast	MCF2L2	Q86YR7	MCF.2 cell line derived transforming sequence-like 2
Malignant neoplasm of breast	LOC100288966	Q86YR6	POTE ankyrin domain family member D-like
Malignant neoplasm of breast	POTED	Q86YR6	POTE ankyrin domain family member D
Malignant neoplasm of breast	BPIFA4P	Q86YQ2	BPI fold containing family A member 4, pseudogene
Malignant neoplasm of breast	PPARGC1B	Q86YN6	PPARG coactivator 1 beta
Malignant neoplasm of breast	PDPN	Q86YL7	podoplanin
Malignant neoplasm of breast	DZIP1	Q86YF9	DAZ interacting zinc finger protein 1
Malignant neoplasm of breast	TMEM25	Q86YD3	transmembrane protein 25
Malignant neoplasm of breast	PTOV1	Q86YD1	PTOV1 extended AT-hook containing adaptor protein
Malignant neoplasm of breast	PALB2	Q86YC2	partner and localizer of BRCA2
Malignant neoplasm of breast	KMT5C	Q86Y97	lysine methyltransferase 5C
Malignant neoplasm of breast	STX12	Q86Y82	syntaxin 12
Malignant neoplasm of breast	CACUL1	Q86Y37	CDK2 associated cullin domain 1
Malignant neoplasm of breast	VRK2	Q86Y07	VRK serine/threonine kinase 2
Malignant neoplasm of breast	TMEM219	Q86XT9	transmembrane protein 219

Malignant neoplasm of breast	CEP57	Q86XR8	centrosomal protein 57
Malignant neoplasm of breast	TMED7-TICAM2	Q86XR7;Q9Y3B3	TMED7-TICAM2 readthrough
Malignant neoplasm of breast	TICAM2	Q86XR7;Q9Y3B3	toll like receptor adaptor molecule 2
Malignant neoplasm of breast	TMED7	Q86XR7;Q9Y3B3	transmembrane p24 trafficking protein 7
Malignant neoplasm of breast	PRIMA1	Q86XR5	proline rich membrane anchor 1
Malignant neoplasm of breast	ANKLE2	Q86XL3	ankyrin repeat and LEM domain containing 2
Malignant neoplasm of breast	VSIG1	Q86XK7	V-set and immunoglobulin domain containing 1
Malignant neoplasm of breast	SFR1	Q86XK3	SWI5 dependent homologous recombination repair protein 1
Malignant neoplasm of breast	FBXO11	Q86XK2	F-box protein 11
Malignant neoplasm of breast	PPP1R3B	Q86XI6	protein phosphatase 1 regulatory subunit 3B
Malignant neoplasm of breast	NCAPG2	Q86XI2	non-SMC condensin II complex subunit G2
Malignant neoplasm of breast	SNX32	Q86XE0	sorting nexin 32
Malignant neoplasm of breast	CARM1	Q86X55	coactivator associated arginine methyltransferase 1
Malignant neoplasm of breast	CHSY1	Q86X52	chondroitin sulfate synthase 1
Malignant neoplasm of breast	LSR	Q86X29	lipolysis stimulated lipoprotein receptor
Malignant neoplasm of breast	TMEM17	Q86X19	transmembrane protein 17
Malignant neoplasm of breast	RALGAPB	Q86X10	Ral GTPase activating protein non-catalytic subunit beta
Malignant neoplasm of breast	STING1	Q86WV6	stimulator of interferon response cGAMP interactor 1
Malignant neoplasm of breast	PROSER2	Q86WR7	proline and serine rich 2

Malignant neoplasm of breast	IFNE	Q86WN2	interferon epsilon
Malignant neoplasm of breast	CHD1L	Q86WJ1	chromodomain helicase DNA binding protein 1 like
Malignant neoplasm of breast	NLRC5	Q86WI3	NLR family CARD domain containing 5
Malignant neoplasm of breast	RASSF3	Q86WH2	Ras association domain family member 3
Malignant neoplasm of breast	ANKRD46	Q86W74	ankyrin repeat domain 46
Malignant neoplasm of breast	PARG	Q86W56;Q86WA6	poly(ADP-ribose) glycohydrolase
Malignant neoplasm of breast	BPHL	Q86W56;Q86WA6	biphenyl hydrolase like
Malignant neoplasm of breast	NLRP8	Q86W28	NLR family pyrin domain containing 8
Malignant neoplasm of breast	NLRP14	Q86W24	NLR family pyrin domain containing 14
Malignant neoplasm of breast	CYP4Z1	Q86W10	cytochrome P450 family 4 subfamily Z member 1
Malignant neoplasm of breast	TSPYL5	Q86VY4	TSPY like 5
Malignant neoplasm of breast	SLC22A16	Q86VW1	solute carrier family 22 member 16
Malignant neoplasm of breast	HOOK3	Q86VS8	hook microtubule tethering protein 3
Malignant neoplasm of breast	TAX1BP1	Q86VP1	Tax1 binding protein 1
Malignant neoplasm of breast	ZNF410	Q86VK4	zinc finger protein 410
Malignant neoplasm of breast	LAPTM4B	Q86VI4	lysosomal protein transmembrane 4 beta
Malignant neoplasm of breast	IQGAP3	Q86VI3	IQ motif containing GTPase activating protein 3
Malignant neoplasm of breast	EXOC3L1	Q86VI1	exocyst complex component 3 like 1
Malignant neoplasm of breast	CD163	Q86VB7	CD163 molecule

Malignant neoplasm of breast	PIM3	Q86V86	Pim-3 proto-oncogene, serine/threonine kinase
Malignant neoplasm of breast	GPR180	Q86V85	G protein-coupled receptor 180
Malignant neoplasm of breast	VASH2	Q86V25	vasohibin 2
Malignant neoplasm of breast	ADIPOR2	Q86V24	adiponectin receptor 2
Malignant neoplasm of breast	CASZ1	Q86V15	castor zinc finger 1
Malignant neoplasm of breast	FAM83A	Q86UY5	family with sequence similarity 83 member A
Malignant neoplasm of breast	FERMT3	Q86UX7	fermitin family member 3
Malignant neoplasm of breast	ITIH5	Q86UX2	inter-alpha-trypsin inhibitor heavy chain 5
Malignant neoplasm of breast	HAPLN4	Q86UW8	hyaluronan and proteoglycan link protein 4
Malignant neoplasm of breast	N4BP2	Q86UW6	NEDD4 binding protein 2
Malignant neoplasm of breast	GGN	Q86UU5	gametogenetin
Malignant neoplasm of breast	PHLDB1	Q86UU1	pleckstrin homology like domain family B member 1
Malignant neoplasm of breast	BCL9L	Q86UU0	BCL9 like
Malignant neoplasm of breast	NLRX1	Q86UT6	NLR family member X1
Malignant neoplasm of breast	KTN1	Q86UP2	kinectin 1
Malignant neoplasm of breast	MAGI2	Q86UL8	membrane associated guanylate kinase, WW and PDZ domain containing 2
Malignant neoplasm of breast	SLCO6A1	Q86UG4	solute carrier organic anion transporter family member 6A1
Malignant neoplasm of breast	TSPAN33	Q86UF1	tetraspanin 33
Malignant neoplasm of breast	TLK2	Q86UE8	tousled like kinase 2

Malignant neoplasm of breast	MTDH	Q86UE4	metadherin
Malignant neoplasm of breast	ZNF546	Q86UE3	zinc finger protein 546
Malignant neoplasm of breast	RPAIN	Q86UA6	RPA interacting protein
Malignant neoplasm of breast	PBRM1	Q86U86	polybromo 1
Malignant neoplasm of breast	LDB1	Q86U70	LIM domain binding 1
Malignant neoplasm of breast	METTL3	Q86U44	methyltransferase like 3
Malignant neoplasm of breast	NOP9	Q86U38	NOP9 nucleolar protein
Malignant neoplasm of breast	ASPG	Q86U10	asparaginase
Malignant neoplasm of breast	PRUNE1	Q86TP1	prune exopolyphosphatase 1
Malignant neoplasm of breast	SYVN1	Q86TM6	synoviolin 1
Malignant neoplasm of breast	DDX53	Q86TM3	DEAD-box helicase 53
Malignant neoplasm of breast	ATG4D	Q86TL0	autophagy related 4D cysteine peptidase
Malignant neoplasm of breast	PEG10	Q86TG7	paternally expressed 10
Malignant neoplasm of breast	SRL	Q86TD4	sarcalumenin
Malignant neoplasm of breast	USP37	Q86T82	ubiquitin specific peptidase 37
Malignant neoplasm of breast	CLEC14A	Q86T13	C-type lectin domain containing 14A
Malignant neoplasm of breast	DHDDS	Q86SQ9	dehydrodolichyl diphosphate synthase subunit
Malignant neoplasm of breast	SCD5	Q86SK9	stearoyl-CoA desaturase 5
Malignant neoplasm of breast	AMIGO2	Q86SJ2	adhesion molecule with Ig like domain 2

Malignant neoplasm of breast	NEK8	Q86SG6	NIMA related kinase 8
Malignant neoplasm of breast	S100A7A	Q86SG5	S100 calcium binding protein A7A
Malignant neoplasm of breast	RALYL	Q86SE5	RALY RNA binding protein like
Malignant neoplasm of breast	GALNT5	Q7Z7M9	polypeptide N-acetylgalactosaminyltransferase 5
Malignant neoplasm of breast	SLFN11	Q7Z7L1	schlafen family member 11
Malignant neoplasm of breast	VPS13B	Q7Z7G8	vacuolar protein sorting 13 homolog B
Malignant neoplasm of breast	UBE2Q1	Q7Z7E8	ubiquitin conjugating enzyme E2 Q1
Malignant neoplasm of breast	VTCN1	Q7Z7D3	V-set domain containing T cell activation inhibitor 1
Malignant neoplasm of breast	TAF8	Q7Z7C8	TATA-box binding protein associated factor 8
Malignant neoplasm of breast	CTU1	Q7Z7A3	cytosolic thiouridylase subunit 1
Malignant neoplasm of breast	YTHDF3	Q7Z739	YTH N6-methyladenosine RNA binding protein 3
Malignant neoplasm of breast	HUWE1	Q7Z6Z7	HECT, UBA and WWE domain containing E3 ubiquitin protein ligase 1
Malignant neoplasm of breast	TFAP2D	Q7Z6R9	transcription factor AP-2 delta
Malignant neoplasm of breast	RABEPK	Q7Z6M1	Rab9 effector protein with kelch motifs
Malignant neoplasm of breast	PRRT2	Q7Z6L0	proline rich transmembrane protein 2
Malignant neoplasm of breast	ARPIN	Q7Z6K5	actin related protein 2/3 complex inhibitor
Malignant neoplasm of breast	NRARP	Q7Z6K4	NOTCH regulated ankyrin repeat protein
Malignant neoplasm of breast	RBBP6	Q7Z6E9	RB binding protein 6, ubiquitin ligase
Malignant neoplasm of breast	BTLA	Q7Z6A9	B and T lymphocyte associated

Malignant neoplasm of breast	SPRED1	Q7Z699	sprouty related EVH1 domain containing 1
Malignant neoplasm of breast	CEACAM19	Q7Z692	CEA cell adhesion molecule 19
Malignant neoplasm of breast	NET1	Q7Z628	neuroepithelial cell transforming 1
Malignant neoplasm of breast	CAMKMT	Q7Z624	calmodulin-lysine N-methyltransferase
Malignant neoplasm of breast	BCDIN3D	Q7Z5W3	BCDIN3 domain containing RNA methyltransferase
Malignant neoplasm of breast	WDR53	Q7Z5U6	WD repeat domain 53
Malignant neoplasm of breast	CPEB2	Q7Z5Q1	cytoplasmic polyadenylation element binding protein 2
Malignant neoplasm of breast	MUC19	Q7Z5P9	mucin 19, oligomeric
Malignant neoplasm of breast	HSD17B13	Q7Z5P4	hydroxysteroid 17-beta dehydrogenase 13
Malignant neoplasm of breast	ABHD12B	Q7Z5M8	abhydrolase domain containing 12B
Malignant neoplasm of breast	RAI1	Q7Z5J4	retinoic acid induced 1
Malignant neoplasm of breast	EMSY	Q7Z589	EMSY transcriptional repressor, BRCA2 interacting
Malignant neoplasm of breast	SPATA21	Q7Z572	spermatogenesis associated 21
Malignant neoplasm of breast	BRAP	Q7Z569	BRCA1 associated protein
Malignant neoplasm of breast	ZNF438	Q7Z4V0	zinc finger protein 438
Malignant neoplasm of breast	TRIM46	Q7Z4K8	tripartite motif containing 46
Malignant neoplasm of breast	TRMT11	Q7Z4G4	tRNA methyltransferase 11 homolog
Malignant neoplasm of breast	CYP2U1	Q7Z449	cytochrome P450 family 2 subfamily U member 1
Malignant neoplasm of breast	ERAS	Q7Z444	ES cell expressed Ras

Malignant neoplasm of breast	MAVS	Q7Z434	mitochondrial antiviral signaling protein
Malignant neoplasm of breast	TMC4	Q7Z404	transmembrane channel like 4
Malignant neoplasm of breast	PIWIL4	Q7Z3Z4	piwi like RNA-mediated gene silencing 4
Malignant neoplasm of breast	MIER3	Q7Z3K6	MIER family member 3
Malignant neoplasm of breast	TIPARP	Q7Z3E1	TCDD inducible poly(ADP-ribose) polymerase
Malignant neoplasm of breast	TRPM8	Q7Z2W7	transient receptor potential cation channel subfamily M member 8
Malignant neoplasm of breast	SLC36A1	Q7Z2H8	solute carrier family 36 member 1
Malignant neoplasm of breast	TMPRSS7	Q7RTY8	transmembrane serine protease 7
Malignant neoplasm of breast	PHF5A	Q7RTV0	PHD finger protein 5A
Malignant neoplasm of breast	DYM	Q7RTS9	dymeclin
Malignant neoplasm of breast	BHLHA15	Q7RTS1	basic helix-loop-helix family member a15
Malignant neoplasm of breast	NLRP9	Q7RTR0	NLR family pyrin domain containing 9
Malignant neoplasm of breast	MICAL3	Q7RTP6	microtubule associated monooxygenase, calponin and LIM domain containing 3
Malignant neoplasm of breast	CHST3	Q7LGC8	carbohydrate sulfotransferase 3
Malignant neoplasm of breast	RRM2B	Q7LG56	ribonucleotide reductase regulatory TP53 inducible subunit M2B
Malignant neoplasm of breast	CHST15	Q7LFX5	carbohydrate sulfotransferase 15
Malignant neoplasm of breast	CXXC5	Q7LFL8	CXXC finger protein 5
Malignant neoplasm of breast	RASGRP2	Q7LDG7	RAS guanyl releasing protein 2
Malignant neoplasm of breast	KDM3B	Q7LBC6	lysine demethylase 3B

Malignant neoplasm of breast	EEPD1	Q7L9B9	endonuclease/exonuclease/phosphatase family domain containing 1
Malignant neoplasm of breast	LINGO2	Q7L985	leucine rich repeat and Ig domain containing 2
Malignant neoplasm of breast	DHX32	Q7L7V1	DEAH-box helicase 32 (putative)
Malignant neoplasm of breast	MAEA	Q7L5Y9	macrophage erythroblast attacher
Malignant neoplasm of breast	COPS6	Q7L5N1	COP9 signalosome subunit 6
Malignant neoplasm of breast	FA2H	Q7L5A8	fatty acid 2-hydroxylase
Malignant neoplasm of breast	MCM10	Q7L590	minichromosome maintenance 10 replication initiation factor
Malignant neoplasm of breast	CYFIP1	Q7L576	cytoplasmic FMR1 interacting protein 1
Malignant neoplasm of breast	RTL10	Q7L3V2	retrotransposon Gag like 10
Malignant neoplasm of breast	EIF3M	Q7L2H7	eukaryotic translation initiation factor 3 subunit M
Malignant neoplasm of breast	ASRGL1	Q7L266	asparaginase and isoaspartyl peptidase 1
Malignant neoplasm of breast	CYB5R4	Q7L1T6	cytochrome b5 reductase 4
Malignant neoplasm of breast	CHST9	Q7L1S5	carbohydrate sulfotransferase 9
Malignant neoplasm of breast	MARK2	Q7KZI7	microtubule affinity regulating kinase 2
Malignant neoplasm of breast	SND1	Q7KZF4	staphylococcal nuclease and tudor domain containing 1
Malignant neoplasm of breast	HECW1	Q76N89	HECT, C2 and WW domain containing E3 ubiquitin protein ligase 1
Malignant neoplasm of breast	ASXL2	Q76L83	ASXL transcriptional regulator 2
Malignant neoplasm of breast	PDZD4	Q76G19	PDZ domain containing 4
Malignant neoplasm of breast	MIR17HG	Q75NE6	miR-17-92a-1 cluster host gene

Malignant neoplasm of breast	CBLL1	Q75N03	Cbl proto-oncogene like 1
Malignant neoplasm of breast	RPS27L	Q71UM5	ribosomal protein S27 like
Malignant neoplasm of breast	MED25	Q71SY5	mediator complex subunit 25
Malignant neoplasm of breast	TLCD3B	Q71RH2	TLC domain containing 3B
Malignant neoplasm of breast	CENPU	Q71F23	centromere protein U
Malignant neoplasm of breast	AHSA2P	Q719I0	activator of HSP90 ATPase homolog 2, pseudogene
Malignant neoplasm of breast	PREX2	Q70Z35	phosphatidylinositol-3,4,5-trisphosphate dependent Rac exchange factor 2
Malignant neoplasm of breast	ZNF365	Q70YC4;Q70YC5	zinc finger protein 365
Malignant neoplasm of breast	CHSY3	Q70JA7	chondroitin sulfate synthase 3
Malignant neoplasm of breast	USP43	Q70EL4	ubiquitin specific peptidase 43
Malignant neoplasm of breast	USP54	Q70EL1	ubiquitin specific peptidase 54
Malignant neoplasm of breast	USP51	Q70EK9	ubiquitin specific peptidase 51
Malignant neoplasm of breast	RAPH1	Q70E73	Ras association (RalGDS/AF-6) and pleckstrin homology domains 1
Malignant neoplasm of breast	XIRP1	Q702N8	xin actin binding repeat containing 1
Malignant neoplasm of breast	TMTC3	Q6ZXV5	transmembrane O-mannosyltransferase targeting cadherins 3
Malignant neoplasm of breast	STXBP4	Q6ZWJ1	syntaxin binding protein 4
Malignant neoplasm of breast	NEK10	Q6ZWH5	NIMA related kinase 10
Malignant neoplasm of breast	PAXIP1	Q6ZW49	PAX interacting protein 1
Malignant neoplasm of breast	HJV	Q6ZVN8	hemojuvelin BMP co-receptor

Malignant neoplasm of breast	GOLT1A	Q6ZVE7	golgi transport 1A
Malignant neoplasm of breast	PHLPP2	Q6ZVD8	PH domain and leucine rich repeat protein phosphatase 2
Malignant neoplasm of breast	TMEM26	Q6ZUK4	transmembrane protein 26
Malignant neoplasm of breast	RASSF6	Q6ZTQ3	Ras association domain family member 6
Malignant neoplasm of breast	TBC1D9	Q6ZT07	TBC1 domain family member 9
Malignant neoplasm of breast	TSHZ1	Q6ZSZ6	teashirt zinc finger homeobox 1
Malignant neoplasm of breast	MMS22L	Q6ZRQ5	MMS22 like, DNA repair protein
Malignant neoplasm of breast	C4orf50	Q6ZRC1	chromosome 4 open reading frame 50
Malignant neoplasm of breast	GADL1	Q6ZQY3	glutamate decarboxylase like 1
Malignant neoplasm of breast	C17orf97	Q6ZQX7	chromosome 17 open reading frame 97
Malignant neoplasm of breast	FGD5	Q6ZNL6	FYVE, RhoGEF and PH domain containing 5
Malignant neoplasm of breast	UNC5A	Q6ZN44	unc-5 netrin receptor A
Malignant neoplasm of breast	MACC1	Q6ZN28	MET transcriptional regulator MACC1
Malignant neoplasm of breast	LIN28B	Q6ZN17	lin-28 homolog B
Malignant neoplasm of breast	WDR88	Q6ZMY6	WD repeat domain 88
Malignant neoplasm of breast	SPOCD1	Q6ZMY3	SPOC domain containing 1
Malignant neoplasm of breast	KIF6	Q6ZMV9	kinesin family member 6
Malignant neoplasm of breast	KDM7A	Q6ZMT4	lysine demethylase 7A
Malignant neoplasm of breast	STAC2	Q6ZMT1	SH3 and cysteine rich domain 2

Malignant neoplasm of breast	IL34	Q6ZMJ4	interleukin 34
Malignant neoplasm of breast	SCARA5	Q6ZMJ2	scavenger receptor class A member 5
Malignant neoplasm of breast	CERS6	Q6ZMG9	ceramide synthase 6
Malignant neoplasm of breast	HSDL2	Q6YN16	hydroxysteroid dehydrogenase like 2
Malignant neoplasm of breast	CD109	Q6YHK3	CD109 molecule
Malignant neoplasm of breast	GIGYF2	Q6Y7W6	GRB10 interacting GYF protein 2
Malignant neoplasm of breast	RBMS3	Q6XE24	RNA binding motif single stranded interacting protein 3
Malignant neoplasm of breast	SOSTDC1	Q6X4U4	sclerostin domain containing 1
Malignant neoplasm of breast	RAB11FIP1	Q6WKZ4	RAB11 family interacting protein 1
Malignant neoplasm of breast	MPRIP	Q6WCQ1	myosin phosphatase Rho interacting protein
Malignant neoplasm of breast	MUC6	Q6W4X9	mucin 6, oligomeric mucus/gel-forming
Malignant neoplasm of breast	ATF7IP	Q6VMQ6	activating transcription factor 7 interacting protein
Malignant neoplasm of breast	FAT4	Q6V0I7	FAT atypical cadherin 4
Malignant neoplasm of breast	DLK2	Q6UY11	delta like non-canonical Notch ligand 2
Malignant neoplasm of breast	RSPO2	Q6UXX9	R-spondin 2
Malignant neoplasm of breast	GFRAL	Q6UXV0	GDNF family receptor alpha like
Malignant neoplasm of breast	SERPINA13P	Q6UXR4	serpin family A member 13, pseudogene
Malignant neoplasm of breast	NPNT	Q6UXI9	nephronectin
Malignant neoplasm of breast	PAMR1	Q6UXH9	peptidase domain containing associated with muscle regeneration 1

Malignant neoplasm of breast	CCBE1	Q6UXH8	collagen and calcium binding EGF domains 1
Malignant neoplasm of breast	KIAA1324	Q6UXG2	KIAA1324
Malignant neoplasm of breast	MAMDC4	Q6UXC1	MAM domain containing 4
Malignant neoplasm of breast	CLEC4G	Q6UXB4	C-type lectin domain family 4 member G
Malignant neoplasm of breast	CXCL17	Q6UXB2	C-X-C motif chemokine ligand 17
Malignant neoplasm of breast	FAM131A	Q6UXB0	family with sequence similarity 131 member A
Malignant neoplasm of breast	METTL7B	Q6UX53	methyltransferase like 7B
Malignant neoplasm of breast	OLFM4	Q6UX06	olfactomedin 4
Malignant neoplasm of breast	ABRAXAS1	Q6UWZ7	abraxas 1, BRCA1 A complex subunit
Malignant neoplasm of breast	ENHO	Q6UWT2	energy homeostasis associated
Malignant neoplasm of breast	DHRS11	Q6UWP2	dehydrogenase/reductase 11
Malignant neoplasm of breast	LYPD5	Q6UWN5	LY6/PLAUR domain containing 5
Malignant neoplasm of breast	LYPD4	Q6UWN0	LY6/PLAUR domain containing 4
Malignant neoplasm of breast	PRSS55	Q6UWB4	serine protease 55
Malignant neoplasm of breast	ATRAID	Q6UW56	all-trans retinoic acid induced differentiation factor
Malignant neoplasm of breast	KCNT2	Q6UVM3	potassium sodium-activated channel subfamily T member 2
Malignant neoplasm of breast	CSPG4	Q6UVK1	chondroitin sulfate proteoglycan 4
Malignant neoplasm of breast	ANKRD11	Q6UB99	ankyrin repeat domain 11
Malignant neoplasm of breast	ANKRD12	Q6UB98	ankyrin repeat domain 12

Malignant neoplasm of breast	OPN5	Q6U736	opsin 5
Malignant neoplasm of breast	CASC1	Q6TDU7	cancer susceptibility 1
Malignant neoplasm of breast	PAQR3	Q6TCH7	progestin and adipoQ receptor family member 3
Malignant neoplasm of breast	NHS	Q6T4R5	NHS actin remodeling regulator
Malignant neoplasm of breast	POTEE	Q6S8J3	POTE ankyrin domain family member E
Malignant neoplasm of breast	BRWD3	Q6RI45	bromodomain and WD repeat domain containing 3
Malignant neoplasm of breast	USP17L2	Q6R6M4	ubiquitin specific peptidase 17 like family member 2
Malignant neoplasm of breast	RICTOR	Q6R327	RPTOR independent companion of MTOR complex 2
Malignant neoplasm of breast	PAOX	Q6QHF9	polyamine oxidase
Malignant neoplasm of breast	TRAF7	Q6Q0C0	TNF receptor associated factor 7
Malignant neoplasm of breast	ATAD2	Q6PL18	ATPase family AAA domain containing 2
Malignant neoplasm of breast	DOK6	Q6PKX4	docking protein 6
Malignant neoplasm of breast	FOXR2	Q6PJQ5	forkhead box R2
Malignant neoplasm of breast	RHBDF2	Q6PJF5	rhomboid 5 homolog 2
Malignant neoplasm of breast	TRAT1	Q6PIZ9	T cell receptor associated transmembrane adaptor 1
Malignant neoplasm of breast	ARMH1	Q6PIY5	armadillo like helical domain containing 1
Malignant neoplasm of breast	KLHDC10	Q6PID8	kelch domain containing 10
Malignant neoplasm of breast	TMEM88	Q6PEY1	transmembrane protein 88
Malignant neoplasm of breast	CTR9	Q6PD62	CTR9 homolog, Paf1/RNA polymerase II complex component

Malignant neoplasm of breast	EMB	Q6PCB8	embigin
Malignant neoplasm of breast	SLC27A1	Q6PCB7	solute carrier family 27 member 1
Malignant neoplasm of breast	ALKBH5	Q6P6C2	alkB homolog 5, RNA demethylase
Malignant neoplasm of breast	PKN3	Q6P5Z2	protein kinase N3
Malignant neoplasm of breast	TNFAIP8L2	Q6P589	TNF alpha induced protein 8 like 2
Malignant neoplasm of breast	CNNM4	Q6P4Q7	cyclin and CBS domain divalent metal cation transport mediator 4
Malignant neoplasm of breast	ARHGAP11A	Q6P4F7	Rho GTPase activating protein 11A
Malignant neoplasm of breast	MTFR2	Q6P444	mitochondrial fission regulator 2
Malignant neoplasm of breast	DNAJC24	Q6P3W2	DnaJ heat shock protein family (Hsp40) member C24
Malignant neoplasm of breast	NEK5	Q6P3R8	NIMA related kinase 5
Malignant neoplasm of breast	PRPF8	Q6P2Q9	pre-mRNA processing factor 8
Malignant neoplasm of breast	PNCK	Q6P2M8	pregnancy up-regulated nonubiquitous CaM kinase
Malignant neoplasm of breast	EDC4	Q6P2E9	enhancer of mRNA decapping 4
Malignant neoplasm of breast	LETMD1	Q6P1Q0	LETM1 domain containing 1
Malignant neoplasm of breast	TATDN1	Q6P1N9	TatD DNase domain containing 1
Malignant neoplasm of breast	LLGL2	Q6P1M3	LLGL scribble cell polarity complex component 2
Malignant neoplasm of breast	SLC27A4	Q6P1M0	solute carrier family 27 member 4
Malignant neoplasm of breast	SLC48A1	Q6P1K1	solute carrier family 48 member 1
Malignant neoplasm of breast	PLB1	Q6P1J6	phospholipase B1

Malignant neoplasm of breast	CAVIN1	Q6NZI2	caveolae associated protein 1
Malignant neoplasm of breast	JMJD6	Q6NYC1	jumonji domain containing 6, arginine demethylase and lysine hydroxylase
Malignant neoplasm of breast	ESRP1	Q6NXG1	epithelial splicing regulatory protein 1
Malignant neoplasm of breast	RGMB	Q6NW40	repulsive guidance molecule BMP co-receptor b
Malignant neoplasm of breast	RINT1	Q6NUQ1	RAD50 interactor 1
Malignant neoplasm of breast	SLC25A24	Q6NUK1	solute carrier family 25 member 24
Malignant neoplasm of breast	GPAT2	Q6NUI2	glycerol-3-phosphate acyltransferase 2, mitochondrial
Malignant neoplasm of breast	RHBDD2	Q6NTF9	rhomboid domain containing 2
Malignant neoplasm of breast	FAM53A	Q6NSI3	family with sequence similarity 53 member A
Malignant neoplasm of breast	ALKBH2	Q6NS38	alkB homolog 2, alpha-ketoglutarate dependent dioxygenase
Malignant neoplasm of breast	NAA16	Q6N069	N-alpha-acetyltransferase 16, NatA auxiliary subunit
Malignant neoplasm of breast	TENM4	Q6N022	teneurin transmembrane protein 4
Malignant neoplasm of breast	TET2	Q6N021	tet methylcytosine dioxygenase 2
Malignant neoplasm of breast	PRR5L	Q6MZQ0	proline rich 5 like
Malignant neoplasm of breast	HCP5	Q6MZN7	HLA complex P5
Malignant neoplasm of breast	NIPBL	Q6KC79	NIPBL cohesin loading factor
Malignant neoplasm of breast	KRT80	Q6KB66	keratin 80
Malignant neoplasm of breast	PYHIN1	Q6K0P9	pyrin and HIN domain family member 1
Malignant neoplasm of breast	STYK1	Q6J9G0	serine/threonine/tyrosine kinase 1

Malignant neoplasm of breast	ANO7	Q6IWH7	anoctamin 7
Malignant neoplasm of breast	GRHL2	Q6ISB3	grainyhead like transcription factor 2
Malignant neoplasm of breast	TMEM132E	Q6IEE7	transmembrane protein 132E
Malignant neoplasm of breast	WBP2NL	Q6ICG8	WBP2 N-terminal like
Malignant neoplasm of breast	DESI1	Q6ICB0	desumoylating isopeptidase 1
Malignant neoplasm of breast	LAMTOR1	Q6IAA8	late endosomal/lysosomal adaptor, MAPK and MTOR activator 1
Malignant neoplasm of breast	SIGIRR	Q6IA17	single Ig and TIR domain containing
Malignant neoplasm of breast	SMYD5	Q6GMV2	SMYD family member 5
Malignant neoplasm of breast	CIAPIN1	Q6FI81	cytokine induced apoptosis inhibitor 1
Malignant neoplasm of breast	VASN	Q6EMK4	vasorin
Malignant neoplasm of breast	IQSEC1	Q6DN90	IQ motif and Sec7 domain ArfGEF 1
Malignant neoplasm of breast	PVRIG	Q6DKI7	PVR related immunoglobulin domain containing
Malignant neoplasm of breast	PLAG1	Q6DJT9	PLAG1 zinc finger
Malignant neoplasm of breast	TBC1D3	Q6DHY5;Q6IPX1;Q8IZP1	TBC1 domain family member 3
Malignant neoplasm of breast	UHRF1BP1	Q6BDS2	UHRF1 binding protein 1
Malignant neoplasm of breast	SCARA3	Q6AZY7	scavenger receptor class A member 3
Malignant neoplasm of breast	HEATR6	Q6AI08	HEAT repeat containing 6
Malignant neoplasm of breast	ANKRD34A	Q69YU3	ankyrin repeat domain 34A
Malignant neoplasm of breast	VIRMA	Q69YN4	vir like m6A methyltransferase associated

Malignant neoplasm of breast	ARHGAP17	Q68EM7	Rho GTPase activating protein 17
Malignant neoplasm of breast	ZFYVE26	Q68DK2	zinc finger FYVE-type containing 26
Malignant neoplasm of breast	FMN1	Q68DA7	formin 1
Malignant neoplasm of breast	NCR3LG1	Q68D85	natural killer cell cytotoxicity receptor 3 ligand 1
Malignant neoplasm of breast	TNS3	Q68CZ2	tensin 3
Malignant neoplasm of breast	MROH7	Q68CQ1	maestro heat like repeat family member 7
Malignant neoplasm of breast	MUC17	Q685J3	mucin 17, cell surface associated
Malignant neoplasm of breast	RUSC1-AS1	Q66K80	RUSC1 antisense RNA 1
Malignant neoplasm of breast	MAP1S	Q66K74	microtubule associated protein 1S
Malignant neoplasm of breast	TBC1D9B	Q66K14	TBC1 domain family member 9B
Malignant neoplasm of breast	CEP135	Q66GS9	centrosomal protein 135
Malignant neoplasm of breast	KANK2	Q63ZY3	KN motif and ankyrin repeat domains 2
Malignant neoplasm of breast	TSHZ3	Q63HK5	teashirt zinc finger homeobox 3
Malignant neoplasm of breast	ALS2CL	Q60I27	ALS2 C-terminal like
Malignant neoplasm of breast	CD276	Q5ZPR3	CD276 molecule
Malignant neoplasm of breast	ANO1	Q5XXA6	anoctamin 1
Malignant neoplasm of breast	FBXO31	Q5XUX0	F-box protein 31
Malignant neoplasm of breast	OTP	Q5XKR4	orthopedia homeobox
Malignant neoplasm of breast	TENT4A	Q5XG87	terminal nucleotidyltransferase 4A

Malignant neoplasm of breast	USPL1	Q5W0Q7	ubiquitin specific peptidase like 1
Malignant neoplasm of breast	PLPP4	Q5VZY2	phospholipid phosphatase 4
Malignant neoplasm of breast	MBNL2	Q5VZF2	muscleblind like splicing regulator 2
Malignant neoplasm of breast	JAKMIP3	Q5VZ66	Janus kinase and microtubule interacting protein 3
Malignant neoplasm of breast	TDRD10	Q5VZ19	tudor domain containing 10
Malignant neoplasm of breast	ECPAS	Q5VYK3	Ecm29 proteasome adaptor and scaffold
Malignant neoplasm of breast	SHOC1	Q5VXU9	shortage in chiasmata 1
Malignant neoplasm of breast	SUSD4	Q5VX71	sushi domain containing 4
Malignant neoplasm of breast	C1QL3	Q5VWW1	complement C1q like 3
Malignant neoplasm of breast	DAB2IP	Q5VWQ8	DAB2 interacting protein
Malignant neoplasm of breast	MLIP	Q5VWP3	muscular LMNA interacting protein
Malignant neoplasm of breast	IL23R	Q5VWK5	interleukin 23 receptor
Malignant neoplasm of breast	ATRNL1	Q5VV63	attractin like 1
Malignant neoplasm of breast	CDKAL1	Q5VV42	CDK5 regulatory subunit associated protein 1 like 1
Malignant neoplasm of breast	OTUD1	Q5VV17	OTU deubiquitinase 1
Malignant neoplasm of breast	FAM171A1	Q5VUB5	family with sequence similarity 171 member A1
Malignant neoplasm of breast	ZNF318	Q5VUA4	zinc finger protein 318
Malignant neoplasm of breast	HHAT	Q5VTY9	hedgehog acyltransferase
Malignant neoplasm of breast	RNF20	Q5VTR2	ring finger protein 20

Malignant neoplasm of breast	PRPF38B	Q5VTL8	pre-mRNA processing factor 38B
Malignant neoplasm of breast	KLHDC7A	Q5VTJ3	kelch domain containing 7A
Malignant neoplasm of breast	CDC42BPA	Q5VT25	CDC42 binding protein kinase alpha
Malignant neoplasm of breast	OBSCN	Q5VST9	obscurin, cytoskeletal calmodulin and titin-interacting RhoGEF
Malignant neoplasm of breast	RIF1	Q5UIP0	replication timing regulatory factor 1
Malignant neoplasm of breast	AGBL2	Q5U5Z8	ATP/GTP binding protein like 2
Malignant neoplasm of breast	CROCC	Q5TZA2	ciliary rootlet coiled-coil, rootletin
Malignant neoplasm of breast	LIN9	Q5TKA1	lin-9 DREAM MuvB core complex component
Malignant neoplasm of breast	CCDC181	Q5TID7	coiled-coil domain containing 181
Malignant neoplasm of breast	STPG1	Q5TH74	sperm tail PG-rich repeat containing 1
Malignant neoplasm of breast	ARFGEF3	Q5TH69	ARFGEF family member 3
Malignant neoplasm of breast	PXDC1	Q5TGL8	PX domain containing 1
Malignant neoplasm of breast	THEMIS2	Q5TEJ8	thymocyte selection associated family member 2
Malignant neoplasm of breast	FHL5	Q5TD97	four and a half LIM domains 5
Malignant neoplasm of breast	MAP3K21	Q5TCX8	mitogen-activated protein kinase kinase kinase 21
Malignant neoplasm of breast	MAGI3	Q5TCQ9	membrane associated guanylate kinase, WW and PDZ domain containing 3
Malignant neoplasm of breast	FRY	Q5TBA9	FRY microtubule binding protein
Malignant neoplasm of breast	DEPDC1	Q5TB30	DEP domain containing 1
Malignant neoplasm of breast	TUT4	Q5TAX3	terminal uridylyl transferase 4

Malignant neoplasm of breast	WLS	Q5T9L3	Wnt ligand secretion mediator
Malignant neoplasm of breast	ATAD3B	Q5T9A4	ATPase family AAA domain containing 3B
Malignant neoplasm of breast	PPP1R26	Q5T8A7	protein phosphatase 1 regulatory subunit 26
Malignant neoplasm of breast	LDLRAD1	Q5T700	low density lipoprotein receptor class A domain containing 1
Malignant neoplasm of breast	GPRC6A	Q5T6X5	G protein-coupled receptor class C group 6 member A
Malignant neoplasm of breast	CCNJ	Q5T5M9	cyclin J
Malignant neoplasm of breast	ARTN	Q5T4W7	artemin
Malignant neoplasm of breast	TMEM170B	Q5T4T1	transmembrane protein 170B
Malignant neoplasm of breast	UBR4	Q5T4S7	ubiquitin protein ligase E3 component n-recognin 4
Malignant neoplasm of breast	SFRP5	Q5T4F7	secreted frizzled related protein 5
Malignant neoplasm of breast	TMTC4	Q5T4D3	transmembrane O-mannosyltransferase targeting cadherins 4
Malignant neoplasm of breast	GJC2	Q5T442	gap junction protein gamma 2
Malignant neoplasm of breast	ABCC10	Q5T3U5	ATP binding cassette subfamily C member 10
Malignant neoplasm of breast	PDZK1	Q5T2W1	PDZ domain containing 1
Malignant neoplasm of breast	OTUD3	Q5T2D3	OTU deubiquitinase 3
Malignant neoplasm of breast	DDX59	Q5T1V6	DEAD-box helicase 59
Malignant neoplasm of breast	HIVEP3	Q5T1R4	HIVEP zinc finger 3
Malignant neoplasm of breast	THEM4	Q5T1C6	thioesterase superfamily member 4
Malignant neoplasm of breast	FAM83B	Q5T0W9	family with sequence similarity 83 member B

Malignant neoplasm of breast	MARCHF8	Q5T0T0	membrane associated ring-CH-type finger 8
Malignant neoplasm of breast	FNBP1L	Q5T0N5	formin binding protein 1 like
Malignant neoplasm of breast	CEP85L	Q5SZL2	centrosomal protein 85 like
Malignant neoplasm of breast	FRMPD1	Q5SYB0	FERM and PDZ domain containing 1
Malignant neoplasm of breast	CATSPERE	Q5SY80	catsper channel auxiliary subunit epsilon
Malignant neoplasm of breast	NOL9	Q5SY16	nucleolar protein 9
Malignant neoplasm of breast	PPP1R15B	Q5SWA1	protein phosphatase 1 regulatory subunit 15B
Malignant neoplasm of breast	DACT2	Q5SW24	dishevelled binding antagonist of beta catenin 2
Malignant neoplasm of breast	VARS2	Q5ST30	valyl-tRNA synthetase 2, mitochondrial
Malignant neoplasm of breast	MANEA	Q5SRI9	mannosidase endo-alpha
Malignant neoplasm of breast	LRRK2	Q5S007	leucine rich repeat kinase 2
Malignant neoplasm of breast	EXOSC6	Q5RKV6	exosome component 6
Malignant neoplasm of breast	BRMS1L	Q5PSV4	BRMS1 like transcriptional repressor
Malignant neoplasm of breast	FFAR4	Q5NUL3	free fatty acid receptor 4
Malignant neoplasm of breast	SPDYA	Q5MJ70	speedy/RINGO cell cycle regulator family member A
Malignant neoplasm of breast	ZNF569	Q5MCW4	zinc finger protein 569
Malignant neoplasm of breast	SPECC1	Q5M775	sperm antigen with calponin homology and coiled-coil domains 1
Malignant neoplasm of breast	EPHA10	Q5JZY3	EPH receptor A10
Malignant neoplasm of breast	MIIP	Q5JXC2	migration and invasion inhibitory protein

Malignant neoplasm of breast	DOP1A	Q5JWR5	DOP1 leucine zipper like protein A
Malignant neoplasm of breast	TOR1AIP1	Q5JTV8	torsin 1A interacting protein 1
Malignant neoplasm of breast	KLF17	Q5JT82	Kruppel like factor 17
Malignant neoplasm of breast	FGD3	Q5JSP0	FYVE, RhoGEF and PH domain containing 3
Malignant neoplasm of breast	DOCK11	Q5JSL3	dedicator of cytokinesis 11
Malignant neoplasm of breast	AKAP4	Q5JQC9	A-kinase anchoring protein 4
Malignant neoplasm of breast	CT47A12	Q5JQC4	cancer/testis antigen family 47 member A12
Malignant neoplasm of breast	CT47A11	Q5JQC4	cancer/testis antigen family 47 member A11
Malignant neoplasm of breast	CT47A7	Q5JQC4	cancer/testis antigen family 47 member A7
Malignant neoplasm of breast	CT47A10	Q5JQC4	cancer/testis antigen family 47 member A10
Malignant neoplasm of breast	CT47A9	Q5JQC4	cancer/testis antigen family 47 member A9
Malignant neoplasm of breast	CT47A8	Q5JQC4	cancer/testis antigen family 47 member A8
Malignant neoplasm of breast	CT47A6	Q5JQC4	cancer/testis antigen family 47 member A6
Malignant neoplasm of breast	CT47A5	Q5JQC4	cancer/testis antigen family 47 member A5
Malignant neoplasm of breast	CT47A4	Q5JQC4	cancer/testis antigen family 47 member A4
Malignant neoplasm of breast	CT47A3	Q5JQC4	cancer/testis antigen family 47 member A3
Malignant neoplasm of breast	CT47A2	Q5JQC4	cancer/testis antigen family 47 member A2
Malignant neoplasm of breast	CT47A1	Q5JQC4	cancer/testis antigen family 47 member A1
Malignant neoplasm of breast	CT45A1	Q5HYN5	cancer/testis antigen family 45 member A1

Malignant neoplasm of breast	FSCB	Q5H9T9	fibrous sheath CABYR binding protein
Malignant neoplasm of breast	TAF7L	Q5H9L4	TATA-box binding protein associated factor 7 like
Malignant neoplasm of breast	TFDP3	Q5H9I0	transcription factor Dp family member 3
Malignant neoplasm of breast	BCORL1	Q5H9F3	BCL6 corepressor like 1
Malignant neoplasm of breast	CT83	Q5H943	cancer/testis antigen 83
Malignant neoplasm of breast	HERC4	Q5GLZ8	HECT and RLD domain containing E3 ubiquitin protein ligase 4
Malignant neoplasm of breast	TNFAIP8L3	Q5GJ75	TNF alpha induced protein 8 like 3
Malignant neoplasm of breast	ARSI	Q5FYB1	arylsulfatase family member I
Malignant neoplasm of breast	DNAJC21	Q5F1R6	DnaJ heat shock protein family (Hsp40) member C21
Malignant neoplasm of breast	CARD16	Q5EG05	caspase recruitment domain family member 16
Malignant neoplasm of breast	CMPK2	Q5EBM0	cytidine/uridine monophosphate kinase 2
Malignant neoplasm of breast	MEST	Q5EB52	mesoderm specific transcript
Malignant neoplasm of breast	ZC3H12A	Q5D1E8	zinc finger CCCH-type containing 12A
Malignant neoplasm of breast	ZNF326	Q5BKZ1	zinc finger protein 326
Malignant neoplasm of breast	FAM133B	Q5BKY9	family with sequence similarity 133 member B
Malignant neoplasm of breast	TMEM97	Q5BJF2	transmembrane protein 97
Malignant neoplasm of breast	DCAF6	Q58WW2	DDB1 and CUL4 associated factor 6
Malignant neoplasm of breast	HSP90B2P	Q58FF3	heat shock protein 90 beta family member 2, pseudogene
Malignant neoplasm of breast	ACTBL2	Q562R1	actin beta like 2

Malignant neoplasm of breast	CYBRD1	Q53TN4	cytochrome b reductase 1
Malignant neoplasm of breast	SMUG1	Q53HV7	single-strand-selective monofunctional uracil-DNA glycosylase 1
Malignant neoplasm of breast	CDCA8	Q53HL2	cell division cycle associated 8
Malignant neoplasm of breast	EIPR1	Q53HC9	EARP complex and GARP complex interacting protein 1
Malignant neoplasm of breast	TRMT12	Q53H54	tRNA methyltransferase 12 homolog
Malignant neoplasm of breast	AGK	Q53H12	acylglycerol kinase
Malignant neoplasm of breast	KLHL22	Q53GT1	kelch like family member 22
Malignant neoplasm of breast	USP39	Q53GS9	ubiquitin specific peptidase 39
Malignant neoplasm of breast	PDLIM3	Q53GG5	PDZ and LIM domain 3
Malignant neoplasm of breast	ACSM3	Q53FZ2	acyl-CoA synthetase medium chain family member 3
Malignant neoplasm of breast	TP53I3	Q53FA7	tumor protein p53 inducible protein 3
Malignant neoplasm of breast	CEP55	Q53EZ4	centrosomal protein 55
Malignant neoplasm of breast	GPAT3	Q53EU6	glycerol-3-phosphate acyltransferase 3
Malignant neoplasm of breast	CRTC2	Q53ET0	CREB regulated transcription coactivator 2
Malignant neoplasm of breast	FNDC3B	Q53EP0	fibronectin type III domain containing 3B
Malignant neoplasm of breast	PDCD4	Q53EL6	programmed cell death 4
Malignant neoplasm of breast	ARHGAP29	Q52LW3	Rho GTPase activating protein 29
Malignant neoplasm of breast	ATP6AP1L	Q52LC2	ATPase H+ transporting accessory protein 1 like
Malignant neoplasm of breast	SLC39A12	Q504Y0	solute carrier family 39 member 12

Malignant neoplasm of breast	GREB1	Q4ZG55	growth regulating estrogen receptor binding 1
Malignant neoplasm of breast	AMOT	Q4VCS5	angiomotin
Malignant neoplasm of breast	BCL7A	Q4VC05	BAF chromatin remodeling complex subunit BCL7A
Malignant neoplasm of breast	ARID4B	Q4LE39	AT-rich interaction domain 4B
Malignant neoplasm of breast	IQCC	Q4KMZ1	IQ motif containing C
Malignant neoplasm of breast	LARP7	Q4G0J3	La ribonucleoprotein 7, transcriptional regulator
Malignant neoplasm of breast	MINDY4	Q4G0A6	MINDY lysine 48 deubiquitinase 4
Malignant neoplasm of breast	MAP9	Q49MG5	microtubule associated protein 9
Malignant neoplasm of breast	CRY2	Q49AN0	cryptochrome circadian regulator 2
Malignant neoplasm of breast	GLYR1	Q49A26	glyoxylate reductase 1 homolog
Malignant neoplasm of breast	LONRF3	Q496Y0	LON peptidase N-terminal domain and ring finger 3
Malignant neoplasm of breast	MMEL1	Q495T6	membrane metalloendopeptidase like 1
Malignant neoplasm of breast	ZNF404	Q494X3	zinc finger protein 404
Malignant neoplasm of breast	FBXL19-AS1	Q494R0	FBXL19 antisense RNA 1
Malignant neoplasm of breast	ZACN	Q401N2	zinc activated ion channel
Malignant neoplasm of breast	LGALSL	Q3ZCW2	galectin like
Malignant neoplasm of breast	TIMM50	Q3ZCQ8	translocase of inner mitochondrial membrane 50
Malignant neoplasm of breast	OTOGL	Q3ZCN5	otogelin like
Malignant neoplasm of breast	RABL6	Q3YEC7	RAB, member RAS oncogene family like 6

Malignant neoplasm of breast	CCDC88A	Q3V6T2	coiled-coil domain containing 88A
Malignant neoplasm of breast	SLC25A52	Q3SY17	solute carrier family 25 member 52
Malignant neoplasm of breast	ARL13B	Q3SXY8	ADP ribosylation factor like GTPase 13B
Malignant neoplasm of breast	KRTAP20-1	Q3LI63	keratin associated protein 20-1
Malignant neoplasm of breast	KRTAP21-1	Q3LI58	keratin associated protein 21-1
Malignant neoplasm of breast	GRAMD1B	Q3KR37	GRAM domain containing 1B
Malignant neoplasm of breast	PLEKHG6	Q3KR16	pleckstrin homology and RhoGEF domain containing G6
Malignant neoplasm of breast	INAVA	Q3KP66	innate immunity activator
Malignant neoplasm of breast	SLC10A6	Q3KNW5	solute carrier family 10 member 6
Malignant neoplasm of breast	NBPF1	Q3BBV0	NBPF member 1
Malignant neoplasm of breast	PLPPR5	Q32ZL2	phospholipid phosphatase related 5
Malignant neoplasm of breast	P3H1	Q32P28	prolyl 3-hydroxylase 1
Malignant neoplasm of breast	LRRFIP1	Q32MZ4	LRR binding FLII interacting protein 1
Malignant neoplasm of breast	HFE	Q30201	homeostatic iron regulator
Malignant neoplasm of breast	HLA-DRB5	Q30154	major histocompatibility complex, class II, DR beta 5
Malignant neoplasm of breast	CTU2	Q2VPK5	cytosolic thiouridylase subunit 2
Malignant neoplasm of breast	LRRC75B	Q2VPJ9	leucine rich repeat containing 75B
Malignant neoplasm of breast	PRICKLE4	Q2TBC4	prickle planar cell polarity protein 4
Malignant neoplasm of breast	HKDC1	Q2TB90	hexokinase domain containing 1

Malignant neoplasm of breast	PARP6	Q2NL67	poly(ADP-ribose) polymerase family member 6
Malignant neoplasm of breast	ERCC6L	Q2NKX8	ERCC excision repair 6 like, spindle assembly checkpoint helicase
Malignant neoplasm of breast	RSPO1	Q2MKA7	R-spondin 1
Malignant neoplasm of breast	SLC35G1	Q2M3R5	solute carrier family 35 member G1
Malignant neoplasm of breast	ABCB5	Q2M3G0	ATP binding cassette subfamily B member 5
Malignant neoplasm of breast	MPEG1	Q2M385	macrophage expressed 1
Malignant neoplasm of breast	ARHGAP31	Q2M1Z3	Rho GTPase activating protein 31
Malignant neoplasm of breast	ZNF423	Q2M1K9	zinc finger protein 423
Malignant neoplasm of breast	KIAA1109	Q2LD37	KIAA1109
Malignant neoplasm of breast	KIF26B	Q2KJY2	kinesin family member 26B
Malignant neoplasm of breast	RSPO4	Q2I0M5	R-spondin 4
Malignant neoplasm of breast	LRRC26	Q2I0M4	leucine rich repeat containing 26
Malignant neoplasm of breast	PDS5A	Q29RF7	PDS5 cohesin associated factor A
Malignant neoplasm of breast	MICA	Q29983	MHC class I polypeptide-related sequence A
Malignant neoplasm of breast	MICB	Q29980	MHC class I polypeptide-related sequence B
Malignant neoplasm of breast	CSPP1	Q1MSJ5	centrosome and spindle pole associated protein 1
Malignant neoplasm of breast	DUOXA1	Q1HG43	dual oxidase maturation factor 1
Malignant neoplasm of breast	DOK7	Q18PE1	docking protein 7
Malignant neoplasm of breast	LY6K	Q17RY6	lymphocyte antigen 6 family member K

Malignant neoplasm of breast	CPEB4	Q17RY0	cytoplasmic polyadenylation element binding protein 4
Malignant neoplasm of breast	GEN1	Q17RS7	GEN1 Holliday junction 5' flap endonuclease
Malignant neoplasm of breast	IMMT	Q16891	inner membrane mitochondrial protein
Malignant neoplasm of breast	TXNRD1	Q16881	thioredoxin reductase 1
Malignant neoplasm of breast	UGT8	Q16880	UDP glycosyltransferase 8
Malignant neoplasm of breast	CDO1	Q16878	cysteine dioxygenase type 1
Malignant neoplasm of breast	PFKFB4	Q16877	6-phosphofructo-2-kinase/fructose-2,6-biphosphatase 4
Malignant neoplasm of breast	PFKFB3	Q16875	6-phosphofructo-2-kinase/fructose-2,6-biphosphatase 3
Malignant neoplasm of breast	AOC3	Q16853	amine oxidase copper containing 3
Malignant neoplasm of breast	DDR2	Q16832	discoidin domain receptor tyrosine kinase 2
Malignant neoplasm of breast	UPP1	Q16831	uridine phosphorylase 1
Malignant neoplasm of breast	DUSP7	Q16829	dual specificity phosphatase 7
Malignant neoplasm of breast	DUSP6	Q16828	dual specificity phosphatase 6
Malignant neoplasm of breast	PTPRO	Q16827	protein tyrosine phosphatase receptor type O
Malignant neoplasm of breast	PPP1R3A	Q16821	protein phosphatase 1 regulatory subunit 3A
Malignant neoplasm of breast	RTN1	Q16799	reticulon 1
Malignant neoplasm of breast	ME3	Q16798	malic enzyme 3
Malignant neoplasm of breast	CA9	Q16790	carbonic anhydrase 9
Malignant neoplasm of breast	LAMA3	Q16787	laminin subunit alpha 3

Malignant neoplasm of breast	H2BC21	Q16778	H2B clustered histone 21
Malignant neoplasm of breast	H2AC20	Q16777	H2A clustered histone 20
Malignant neoplasm of breast	QPCT	Q16769	glutaminyl-peptide cyclotransferase
Malignant neoplasm of breast	UBE2S	Q16763	ubiquitin conjugating enzyme E2 S
Malignant neoplasm of breast	UGCG	Q16739	UDP-glucose ceramide glucosyltransferase
Malignant neoplasm of breast	ATP2B3	Q16720	ATPase plasma membrane Ca2+ transporting 3
Malignant neoplasm of breast	KYNU	Q16719	kynureninase
Malignant neoplasm of breast	DECR1	Q16698	2,4-dienoyl-CoA reductase 1
Malignant neoplasm of breast	DUSP5	Q16690	dual specificity phosphatase 5
Malignant neoplasm of breast	CYP1B1	Q16678	cytochrome P450 family 1 subfamily B member 1
Malignant neoplasm of breast	FOXD1	Q16676	forkhead box D1
Malignant neoplasm of breast	MIA	Q16674	MIA SH3 domain containing
Malignant neoplasm of breast	AMHR2	Q16671	anti-Mullerian hormone receptor type 2
Malignant neoplasm of breast	CDKN3	Q16667	cyclin dependent kinase inhibitor 3
Malignant neoplasm of breast	IFI16	Q16666	interferon gamma inducible protein 16
Malignant neoplasm of breast	HIF1A	Q16665	hypoxia inducible factor 1 subunit alpha
Malignant neoplasm of breast	MAPK6	Q16659	mitogen-activated protein kinase 6
Malignant neoplasm of breast	FSCN1	Q16658	fascin actin-bundling protein 1
Malignant neoplasm of breast	NRF1	Q16656	nuclear respiratory factor 1

Malignant neoplasm of breast	MLANA	Q16655	melan-A
Malignant neoplasm of breast	PDK4	Q16654	pyruvate dehydrogenase kinase 4
Malignant neoplasm of breast	PRSS8	Q16651	serine protease 8
Malignant neoplasm of breast	NFIL3	Q16649	nuclear factor, interleukin 3 regulated
Malignant neoplasm of breast	PTGIS	Q16647	prostaglandin I2 synthase
Malignant neoplasm of breast	DBN1	Q16643	drebrin 1
Malignant neoplasm of breast	SMN1	Q16637	survival of motor neuron 1, telomeric
Malignant neoplasm of breast	SMN2	Q16637	survival of motor neuron 2, centromeric
Malignant neoplasm of breast	TAZ	Q16635	tafazzin
Malignant neoplasm of breast	CPSF6	Q16630	cleavage and polyadenylation specific factor 6
Malignant neoplasm of breast	CCL14	Q16627	C-C motif chemokine ligand 14
Malignant neoplasm of breast	OCLN	Q16625	occludin
Malignant neoplasm of breast	STX1A	Q16623	syntaxin 1A
Malignant neoplasm of breast	NTRK2	Q16620	neurotrophic receptor tyrosine kinase 2
Malignant neoplasm of breast	AANAT	Q16613	aralkylamine N-acetyltransferase
Malignant neoplasm of breast	BAK1	Q16611	BCL2 antagonist/killer 1
Malignant neoplasm of breast	ECM1	Q16610	extracellular matrix protein 1
Malignant neoplasm of breast	CCNG2	Q16589	cyclin G2
Malignant neoplasm of breast	MAP3K11	Q16584	mitogen-activated protein kinase kinase kinase 11

Malignant neoplasm of breast	RBBP7	Q16576	RB binding protein 7, chromatin remodeling factor
Malignant neoplasm of breast	ACKR1	Q16570	atypical chemokine receptor 1 (Duffy blood group)
Malignant neoplasm of breast	CARTPT	Q16568	CART prepropeptide
Malignant neoplasm of breast	DPYSL2	Q16555	dihydropyrimidinase like 2
Malignant neoplasm of breast	LY6E	Q16553	lymphocyte antigen 6 family member E
Malignant neoplasm of breast	IL17A	Q16552	interleukin 17A
Malignant neoplasm of breast	PCSK7	Q16549	proprotein convertase subtilisin/kexin type 7
Malignant neoplasm of breast	BCL2A1	Q16548	BCL2 related protein A1
Malignant neoplasm of breast	MAPK14	Q16539	mitogen-activated protein kinase 14
Malignant neoplasm of breast	PPP2R5E	Q16537	protein phosphatase 2 regulatory subunit B'epsilon
Malignant neoplasm of breast	SNAPC1	Q16533	small nuclear RNA activating complex polypeptide 1
Malignant neoplasm of breast	CSRP2	Q16527	cysteine and glycine rich protein 2
Malignant neoplasm of breast	CRY1	Q16526	cryptochrome circadian regulator 1
Malignant neoplasm of breast	RPE65	Q16518	retinoid isomerohydrolase RPE65
Malignant neoplasm of breast	NNAT	Q16517	neuronatin
Malignant neoplasm of breast	EXT1	Q16394	exostosin glycosyltransferase 1
Malignant neoplasm of breast	SSX2	Q16385	SSX family member 2
Malignant neoplasm of breast	SSX2B	Q16385	SSX family member 2B
Malignant neoplasm of breast	INA	Q16352	internexin neuronal intermediate filament protein alpha

Malignant neoplasm of breast	PDCD2	Q16342	programmed cell death 2
Malignant neoplasm of breast	NTRK3	Q16288	neurotrophic receptor tyrosine kinase 3
Malignant neoplasm of breast	CNGA2	Q16280	cyclic nucleotide gated channel subunit alpha 2
Malignant neoplasm of breast	IGFBP7	Q16270	insulin like growth factor binding protein 7
Malignant neoplasm of breast	E2F4	Q16254	E2F transcription factor 4
Malignant neoplasm of breast	NFE2L2	Q16236	nuclear factor, erythroid 2 like 2
Malignant neoplasm of breast	ENOX2	Q16206	ecto-NOX disulfide-thiol exchanger 2
Malignant neoplasm of breast	CCDC6	Q16204	coiled-coil domain containing 6
Malignant neoplasm of breast	ADRM1	Q16186	adhesion regulating molecule 1
Malignant neoplasm of breast	SEPTIN7	Q16181	septin 7
Malignant neoplasm of breast	SNCB	Q16143	synuclein beta
Malignant neoplasm of breast	HSPB2	Q16082	heat shock protein family B (small) member 2
Malignant neoplasm of breast	ZYX	Q15942	zyxin
Malignant neoplasm of breast	ZIC1	Q15915	Zic family member 1
Malignant neoplasm of breast	ZFHX3	Q15911;Q6ZP98	zinc finger homeobox 3
Malignant neoplasm of breast	EZH2	Q15910	enhancer of zeste 2 polycomb repressive complex 2 subunit
Malignant neoplasm of breast	VPS72	Q15906	vacuolar protein sorting 72 homolog
Malignant neoplasm of breast	CACNA1E	Q15878	calcium voltage-gated channel subunit alpha1 E
Malignant neoplasm of breast	USF2	Q15853	upstream transcription factor 2, c-fos interacting

Malignant neoplasm of breast	SLC14A2	Q15849	solute carrier family 14 member 2
Malignant neoplasm of breast	ADIPOQ	Q15848	adiponectin, C1Q and collagen domain containing
Malignant neoplasm of breast	CLUL1	Q15846	clusterin like 1
Malignant neoplasm of breast	NEDD8	Q15843	NEDD8 ubiquitin like modifier
Malignant neoplasm of breast	STK11	Q15831	serine/threonine kinase 11
Malignant neoplasm of breast	CST6	Q15828	cystatin E/M
Malignant neoplasm of breast	UBE2V2	Q15819	ubiquitin conjugating enzyme E2 V2
Malignant neoplasm of breast	ITSN1	Q15811	intersectin 1
Malignant neoplasm of breast	SMAD1	Q15797	SMAD family member 1
Malignant neoplasm of breast	SMAD2	Q15796	SMAD family member 2
Malignant neoplasm of breast	NCOA1	Q15788	nuclear receptor coactivator 1
Malignant neoplasm of breast	CHI3L2	Q15782	chitinase 3 like 2
Malignant neoplasm of breast	MLF2	Q15773	myeloid leukemia factor 2
Malignant neoplasm of breast	CD226	Q15762	CD226 molecule
Malignant neoplasm of breast	NPY5R	Q15761	neuropeptide Y receptor Y5
Malignant neoplasm of breast	GPR19	Q15760	G protein-coupled receptor 19
Malignant neoplasm of breast	MAPK11	Q15759	mitogen-activated protein kinase 11
Malignant neoplasm of breast	SLC1A5	Q15758	solute carrier family 1 member 5
Malignant neoplasm of breast	MYLK	Q15746	myosin light chain kinase

Malignant neoplasm of breast	GPR68	Q15743	G protein-coupled receptor 68
Malignant neoplasm of breast	INPP5J	Q15735	inositol polyphosphate-5-phosphatase J
Malignant neoplasm of breast	KISS1	Q15726	KiSS-1 metastasis suppressor
Malignant neoplasm of breast	LTB4R	Q15722	leukotriene B4 receptor
Malignant neoplasm of breast	ELAVL1	Q15717	ELAV like RNA binding protein 1
Malignant neoplasm of breast	ALX1	Q15699	ALX homeobox 1
Malignant neoplasm of breast	MAPRE1	Q15691	microtubule associated protein RP/EB family member 1
Malignant neoplasm of breast	PTPN14	Q15678	protein tyrosine phosphatase non-receptor type 14
Malignant neoplasm of breast	TWIST1	Q15672	twist family bHLH transcription factor 1
Malignant neoplasm of breast	JMJD1C	Q15652	jumonji domain containing 1C
Malignant neoplasm of breast	TRIP4	Q15650	thyroid hormone receptor interactor 4
Malignant neoplasm of breast	MED1	Q15648	mediator complex subunit 1
Malignant neoplasm of breast	TRIP13	Q15645	thyroid hormone receptor interactor 13
Malignant neoplasm of breast	TRIP10	Q15642	thyroid hormone receptor interactor 10
Malignant neoplasm of breast	TARBP2	Q15633	TARBP2 subunit of RISC loading complex
Malignant neoplasm of breast	TRAM1	Q15629	translocation associated membrane protein 1
Malignant neoplasm of breast	TRADD	Q15628	TNFRSF1A associated via death domain
Malignant neoplasm of breast	SLC9A3R2	Q15599	SLC9A3 regulator 2
Malignant neoplasm of breast	NCOA2	Q15596	nuclear receptor coactivator 2

Malignant neoplasm of breast	TGFBI	Q15582	transforming growth factor beta induced
Malignant neoplasm of breast	TESK1	Q15569	testis associated actin remodelling kinase 1
Malignant neoplasm of breast	TEAD4	Q15561	TEA domain transcription factor 4
Malignant neoplasm of breast	TCEA2	Q15560	transcription elongation factor A2
Malignant neoplasm of breast	TERF2	Q15554	telomeric repeat binding factor 2
Malignant neoplasm of breast	MMD	Q15546	monocyte to macrophage differentiation associated
Malignant neoplasm of breast	TAF7	Q15545	TATA-box binding protein associated factor 7
Malignant neoplasm of breast	SURF1	Q15526	SURF1 cytochrome c oxidase assembly factor
Malignant neoplasm of breast	SPA17	Q15506	sperm autoantigenic protein 17
Malignant neoplasm of breast	RGN	Q15493	regucalcin
Malignant neoplasm of breast	SKIV2L	Q15477	Ski2 like RNA helicase
Malignant neoplasm of breast	SIX1	Q15475	SIX homeobox 1
Malignant neoplasm of breast	NR0B2	Q15466	nuclear receptor subfamily 0 group B member 2
Malignant neoplasm of breast	SHH	Q15465	sonic hedgehog signaling molecule
Malignant neoplasm of breast	PPP1R7	Q15435	protein phosphatase 1 regulatory subunit 7
Malignant neoplasm of breast	RBMS2	Q15434	RNA binding motif single stranded interacting protein 2
Malignant neoplasm of breast	SAFB	Q15424	scaffold attachment factor B
Malignant neoplasm of breast	RPS6KA1	Q15418	ribosomal protein S6 kinase A1
Malignant neoplasm of breast	RYR3	Q15413	ryanodine receptor 3

Malignant neoplasm of breast	RSU1	Q15404	Ras suppressor protein 1
Malignant neoplasm of breast	TLR1	Q15399	toll like receptor 1
Malignant neoplasm of breast	PUM3	Q15397	pumilio RNA binding family member 3
Malignant neoplasm of breast	SF3B3	Q15393	splicing factor 3b subunit 3
Malignant neoplasm of breast	DHCR24	Q15392	24-dehydrocholesterol reductase
Malignant neoplasm of breast	ANGPT1	Q15389	angiopoietin 1
Malignant neoplasm of breast	UBE3C	Q15386	ubiquitin protein ligase E3C
Malignant neoplasm of breast	RHEB	Q15382	Ras homolog, mTORC1 binding
Malignant neoplasm of breast	ELOC	Q15369	elongin C
Malignant neoplasm of breast	PCBP1	Q15365	poly(rC) binding protein 1
Malignant neoplasm of breast	TMED2	Q15363	transmembrane p24 trafficking protein 2
Malignant neoplasm of breast	TTF1	Q15361	transcription termination factor 1
Malignant neoplasm of breast	RPS6KA2	Q15349	ribosomal protein S6 kinase A2
Malignant neoplasm of breast	LLGL1	Q15334	LLGL scribble cell polarity complex component 1
Malignant neoplasm of breast	E2F5	Q15329	E2F transcription factor 5
Malignant neoplasm of breast	ZMYND11	Q15326	zinc finger MYND-type containing 11
Malignant neoplasm of breast	RALBP1	Q15311	ralA binding protein 1
Malignant neoplasm of breast	IRF4	Q15306	interferon regulatory factor 4
Malignant neoplasm of breast	ERBB4	Q15303	erb-b2 receptor tyrosine kinase 4

Malignant neoplasm of breast	RASA2	Q15283	RAS p21 protein activator 2
Malignant neoplasm of breast	PTPRK	Q15262	protein tyrosine phosphatase receptor type K
Malignant neoplasm of breast	PTPA	Q15257	protein phosphatase 2 phosphatase activator
Malignant neoplasm of breast	NONO	Q15233	non-POU domain containing octamer binding
Malignant neoplasm of breast	NECTIN1	Q15223	nectin cell adhesion molecule 1
Malignant neoplasm of breast	PTGES3	Q15185	prostaglandin E synthase 3
Malignant neoplasm of breast	PPA1	Q15181	inorganic pyrophosphatase 1
Malignant neoplasm of breast	PON2	Q15165	paraoxonase 2
Malignant neoplasm of breast	PLCB4	Q15147	phospholipase C beta 4
Malignant neoplasm of breast	PRKD1	Q15139	protein kinase D1
Malignant neoplasm of breast	CDK10	Q15131	cyclin dependent kinase 10
Malignant neoplasm of breast	EBP	Q15125	EBP cholestenol delta-isomerase
Malignant neoplasm of breast	PEA15	Q15121	proliferation and apoptosis adaptor protein 15
Malignant neoplasm of breast	PDK1	Q15118	pyruvate dehydrogenase kinase 1
Malignant neoplasm of breast	PDCD1	Q15116	programmed cell death 1
Malignant neoplasm of breast	AGER	Q15109	advanced glycosylation end-product specific receptor
Malignant neoplasm of breast	PDIA6	Q15084	protein disulfide isomerase family A member 6
Malignant neoplasm of breast	CDK5R1	Q15078	cyclin dependent kinase 5 regulatory subunit 1
Malignant neoplasm of breast	P2RY6	Q15077	pyrimidinergic receptor P2Y6

Malignant neoplasm of breast	OXA1L	Q15070	OXA1L mitochondrial inner membrane protein
Malignant neoplasm of breast	POSTN	Q15063	periostin
Malignant neoplasm of breast	BRD3	Q15059	bromodomain containing 3
Malignant neoplasm of breast	KIF14	Q15058	kinesin family member 14
Malignant neoplasm of breast	POLD3	Q15054	DNA polymerase delta 3, accessory subunit
Malignant neoplasm of breast	RRS1	Q15050	ribosome biogenesis regulator 1 homolog
Malignant neoplasm of breast	MLC1	Q15049	modulator of VRAC current 1
Malignant neoplasm of breast	SETDB1	Q15047	SET domain bifurcated histone lysine methyltransferase 1
Malignant neoplasm of breast	ARL6IP1	Q15041	ADP ribosylation factor like GTPase 6 interacting protein 1
Malignant neoplasm of breast	ACAP1	Q15027	ArfGAP with coiled-coil, ankyrin repeat and PH domains 1
Malignant neoplasm of breast	TNIP1	Q15025	TNFAIP3 interacting protein 1
Malignant neoplasm of breast	SUZ12	Q15022	SUZ12 polycomb repressive complex 2 subunit
Malignant neoplasm of breast	SART3	Q15020	spliceosome associated factor 3, U4/U6 recycling protein
Malignant neoplasm of breast	SEPTIN2	Q15019	septin 2
Malignant neoplasm of breast	WTAP	Q15007	WT1 associated protein
Malignant neoplasm of breast	PCLAF	Q15004	PCNA clamp associated factor
Malignant neoplasm of breast	HEPACAM	Q14CZ8	hepatic and glial cell adhesion molecule
Malignant neoplasm of breast	FASTKD3	Q14CZ7	FAST kinase domains 3
Malignant neoplasm of breast	NAA25	Q14CX7	N-alpha-acetyltransferase 25, NatB auxiliary subunit

Malignant neoplasm of breast	CLCA4	Q14CN2	chloride channel accessory 4
Malignant neoplasm of breast	TMEM132D	Q14C87	transmembrane protein 132D
Malignant neoplasm of breast	NR1D2	Q14995	nuclear receptor subfamily 1 group D member 2
Malignant neoplasm of breast	NR1I3	Q14994	nuclear receptor subfamily 1 group I member 3
Malignant neoplasm of breast	COL19A1	Q14993	collagen type XIX alpha 1 chain
Malignant neoplasm of breast	OPCML	Q14982	opioid binding protein/cell adhesion molecule like
Malignant neoplasm of breast	NUMA1	Q14980	nuclear mitotic apparatus protein 1
Malignant neoplasm of breast	GPNMB	Q14956	glycoprotein nmb
Malignant neoplasm of breast	KIR2DS1	Q14954	killer cell immunoglobulin like receptor, two Ig domains and short cytoplasmic tail 1
Malignant neoplasm of breast	NFATC4	Q14934	nuclear factor of activated T cells 4
Malignant neoplasm of breast	PTGR1	Q14914	prostaglandin reductase 1
Malignant neoplasm of breast	STARD3	Q14849	StAR related lipid transfer domain containing 3
Malignant neoplasm of breast	LASP1	Q14847	LIM and SH3 protein 1
Malignant neoplasm of breast	CHD4	Q14839	chromodomain helicase DNA binding protein 4
Malignant neoplasm of breast	GRM4	Q14833	glutamate metabotropic receptor 4
Malignant neoplasm of breast	KIF22	Q14807	kinesin family member 22
Malignant neoplasm of breast	FXYD3	Q14802	FXYD domain containing ion transport regulator 3
Malignant neoplasm of breast	CASP8	Q14790	caspase 8
Malignant neoplasm of breast	GOLGB1	Q14789	golgin B1

Malignant neoplasm of breast	CBX2	Q14781	chromobox 2
Malignant neoplasm of breast	ICAM4	Q14773	intercellular adhesion molecule 4 (Landsteiner-Wiener blood group)
Malignant neoplasm of breast	STAT4	Q14765	signal transducer and activator of transcription 4
Malignant neoplasm of breast	MVP	Q14764	major vault protein
Malignant neoplasm of breast	GNMT	Q14749	glycine N-methyltransferase
Malignant neoplasm of breast	LBR	Q14739	lamin B receptor
Malignant neoplasm of breast	MBTPS1	Q14703	membrane bound transcription factor peptidase, site 1
Malignant neoplasm of breast	RFTN1	Q14699	raftlin, lipid raft linker 1
Malignant neoplasm of breast	LPIN1	Q14693	lipin 1
Malignant neoplasm of breast	GINS1	Q14691	GINS complex subunit 1
Malignant neoplasm of breast	DIP2A	Q14689	disco interacting protein 2 homolog A
Malignant neoplasm of breast	GSE1	Q14687	Gse1 coiled-coil protein
Malignant neoplasm of breast	NCOA6	Q14686	nuclear receptor coactivator 6
Malignant neoplasm of breast	RRP1B	Q14684	ribosomal RNA processing 1B
Malignant neoplasm of breast	MELK	Q14680	maternal embryonic leucine zipper kinase
Malignant neoplasm of breast	KANK1	Q14678	KN motif and ankyrin repeat domains 1
Malignant neoplasm of breast	MDC1	Q14676	mediator of DNA damage checkpoint 1
Malignant neoplasm of breast	ESPL1	Q14674	extra spindle pole bodies like 1, separase
Malignant neoplasm of breast	KIAA0100	Q14667	KIAA0100

Malignant neoplasm of breast	IRF3	Q14653	interferon regulatory factor 3
Malignant neoplasm of breast	ITPR1	Q14643	inositol 1,4,5-trisphosphate receptor type 1
Malignant neoplasm of breast	IL13RA2	Q14627	interleukin 13 receptor subunit alpha 2
Malignant neoplasm of breast	ITIH4	Q14624	inter-alpha-trypsin inhibitor heavy chain 4
Malignant neoplasm of breast	NBR1	Q14596	NBR1 autophagy cargo receptor
Malignant neoplasm of breast	DSC3	Q14574	desmocollin 3
Malignant neoplasm of breast	ITPR3	Q14573	inositol 1,4,5-trisphosphate receptor type 3
Malignant neoplasm of breast	HSP90AA2P	Q14568	heat shock protein 90 alpha family class A member 2, pseudogene
Malignant neoplasm of breast	MCM6	Q14566	minichromosome maintenance complex component 6
Malignant neoplasm of breast	SEMA3A	Q14563	semaphorin 3A
Malignant neoplasm of breast	HNF4G	Q14541	hepatocyte nuclear factor 4 gamma
Malignant neoplasm of breast	SQLE	Q14534	squalene epoxidase
Malignant neoplasm of breast	KRT81	Q14533	keratin 81
Malignant neoplasm of breast	HIC1	Q14526	HIC ZBTB transcriptional repressor 1
Malignant neoplasm of breast	SCN5A	Q14524	sodium voltage-gated channel alpha subunit 5
Malignant neoplasm of breast	HABP2	Q14520	hyaluronan binding protein 2
Malignant neoplasm of breast	FAT1	Q14517	FAT atypical cadherin 1
Malignant neoplasm of breast	SPARCL1	Q14515	SPARC like 1
Malignant neoplasm of breast	FGFBP1	Q14512	fibroblast growth factor binding protein 1

Malignant neoplasm of breast	NEDD9	Q14511	neural precursor cell expressed, developmentally down-regulated 9
Malignant neoplasm of breast	RBM39	Q14498	RNA binding motif protein 39
Malignant neoplasm of breast	NFE2L1	Q14494	nuclear factor, erythroid 2 like 1
Malignant neoplasm of breast	SLBP	Q14493	stem-loop binding protein
Malignant neoplasm of breast	HES1	Q14469	hes family bHLH transcription factor 1
Malignant neoplasm of breast	BECN1	Q14457	beclin 1
Malignant neoplasm of breast	GRB7	Q14451	growth factor receptor bound protein 7
Malignant neoplasm of breast	GRB14	Q14449	growth factor receptor bound protein 14
Malignant neoplasm of breast	CAPRIN1	Q14444	cell cycle associated protein 1
Malignant neoplasm of breast	CSHL1	Q14406	chorionic somatomammotropin hormone like 1
Malignant neoplasm of breast	GAS6	Q14393	growth arrest specific 6
Malignant neoplasm of breast	LRRC32	Q14392	leucine rich repeat containing 32
Malignant neoplasm of breast	GNA13	Q14344	G protein subunit alpha 13
Malignant neoplasm of breast	FASTK	Q14296	Fas activated serine/threonine kinase
Malignant neoplasm of breast	PTK2B	Q14289	protein tyrosine kinase 2 beta
Malignant neoplasm of breast	ERV3-1	Q14264	endogenous retrovirus group 3 member 1, envelope
Malignant neoplasm of breast	TRIM25	Q14258	tripartite motif containing 25
Malignant neoplasm of breast	FLOT2	Q14254	flotillin 2
Malignant neoplasm of breast	CTTN	Q14247	cortactin

Malignant neoplasm of breast	SELPLG	Q14242	selectin P ligand
Malignant neoplasm of breast	EIF4A2	Q14240	eukaryotic translation initiation factor 4A2
Malignant neoplasm of breast	EIF2B1	Q14232	eukaryotic translation initiation factor 2B subunit alpha
Malignant neoplasm of breast	EBI3	Q14213	Epstein-Barr virus induced 3
Malignant neoplasm of breast	E2F2	Q14209	E2F transcription factor 2
Malignant neoplasm of breast	MRPL58	Q14197	mitochondrial ribosomal protein L58
Malignant neoplasm of breast	DPYSL3	Q14195	dihydropyrimidinase like 3
Malignant neoplasm of breast	CRMP1	Q14194	collapsin response mediator protein 1
Malignant neoplasm of breast	FHL2	Q14192	four and a half LIM domains 2
Malignant neoplasm of breast	WRN	Q14191	WRN RecQ like helicase
Malignant neoplasm of breast	SIM2	Q14190	SIM bHLH transcription factor 2
Malignant neoplasm of breast	TFDP1	Q14186	transcription factor Dp-1
Malignant neoplasm of breast	DOCK1	Q14185	dedicator of cytokinesis 1
Malignant neoplasm of breast	IKBKE	Q14164	inhibitor of nuclear factor kappa B kinase subunit epsilon
Malignant neoplasm of breast	SCRIB	Q14160	scribble planar cell polarity protein
Malignant neoplasm of breast	UBAP2L	Q14157	ubiquitin associated protein 2 like
Malignant neoplasm of breast	ARHGEF7	Q14155	Rho guanine nucleotide exchange factor 7
Malignant neoplasm of breast	EIF3A	Q14152	eukaryotic translation initiation factor 3 subunit A
Malignant neoplasm of breast	SAFB2	Q14151	scaffold attachment factor B2

Malignant neoplasm of breast	KEAP1	Q14145	kelch like ECH associated protein 1
Malignant neoplasm of breast	TRIM14	Q14142	tripartite motif containing 14
Malignant neoplasm of breast	SEPTIN6	Q14141	septin 6
Malignant neoplasm of breast	VGLL4	Q14135	vestigial like family member 4
Malignant neoplasm of breast	TRIM29	Q14134	tripartite motif containing 29
Malignant neoplasm of breast	VEZF1	Q14119	vascular endothelial zinc finger 1
Malignant neoplasm of breast	DAG1	Q14118	dystroglycan 1
Malignant neoplasm of breast	DPYS	Q14117	dihydropyrimidinase
Malignant neoplasm of breast	IL18	Q14116	interleukin 18
Malignant neoplasm of breast	LRP8	Q14114	LDL receptor related protein 8
Malignant neoplasm of breast	NID2	Q14112	nidogen 2
Malignant neoplasm of breast	CYLC2	Q14093	cylicin 2
Malignant neoplasm of breast	BAAT	Q14032	bile acid-CoA:amino acid N-acyltransferase
Malignant neoplasm of breast	CNGB1	Q14028	cyclic nucleotide gated channel subunit beta 1
Malignant neoplasm of breast	COTL1	Q14019	coactosin like F-actin binding protein 1
Malignant neoplasm of breast	CAMK1	Q14012	calcium/calmodulin dependent protein kinase I
Malignant neoplasm of breast	CIRBP	Q14011	cold inducible RNA binding protein
Malignant neoplasm of breast	IL16	Q14005	interleukin 16
Malignant neoplasm of breast	CEACAM7	Q14002	CEA cell adhesion molecule 7

Malignant neoplasm of breast	RASGRF1	Q13972	Ras protein specific guanine nucleotide releasing factor 1
Malignant neoplasm of breast	NFYC	Q13952	nuclear transcription factor Y subunit gamma
Malignant neoplasm of breast	CBFB	Q13951	core-binding factor subunit beta
Malignant neoplasm of breast	RUNX2	Q13950	RUNX family transcription factor 2
Malignant neoplasm of breast	BYSL	Q13895	bystin like
Malignant neoplasm of breast	GTF2H2	Q13888	general transcription factor IIH subunit 2
Malignant neoplasm of breast	KLF5	Q13887	Kruppel like factor 5
Malignant neoplasm of breast	KLF9	Q13886	Kruppel like factor 9
Malignant neoplasm of breast	PTK6	Q13882	protein tyrosine kinase 6
Malignant neoplasm of breast	BMPR2	Q13873	bone morphogenetic protein receptor type 2
Malignant neoplasm of breast	TNFAIP1	Q13829	TNF alpha induced protein 1
Malignant neoplasm of breast	ENPP2	Q13822	ectonucleotide pyrophosphatase/phosphodiesterase 2
Malignant neoplasm of breast	SPTAN1	Q13813	spectrin alpha, non-erythrocytic 1
Malignant neoplasm of breast	ITGA9	Q13797	integrin subunit alpha 9
Malignant neoplasm of breast	SHROOM2	Q13796	shroom family member 2
Malignant neoplasm of breast	ARFRP1	Q13795	ADP ribosylation factor related protein 1
Malignant neoplasm of breast	PMAIP1	Q13794	phorbol-12-myristate-13-acetate-induced protein 1
Malignant neoplasm of breast	NCOA4	Q13772	nuclear receptor coactivator 4
Malignant neoplasm of breast	THOC5	Q13769	THO complex 5

Malignant neoplasm of breast	RUNX3	Q13761	RUNX family transcription factor 3
Malignant neoplasm of breast	LAMC2	Q13753	laminin subunit gamma 2
Malignant neoplasm of breast	LAMB3	Q13751	laminin subunit beta 3
Malignant neoplasm of breast	ALCAM	Q13740	activated leukocyte cell adhesion molecule
Malignant neoplasm of breast	ACVR2B	Q13705	activin A receptor type 2B
Malignant neoplasm of breast	RAPSN	Q13702	receptor associated protein of the synapse
Malignant neoplasm of breast	AAMP	Q13685	angio associated migratory cell protein
Malignant neoplasm of breast	ITGA7	Q13683	integrin subunit alpha 7
Malignant neoplasm of breast	FHL3	Q13643	four and a half LIM domains 3
Malignant neoplasm of breast	FHL1	Q13642	four and a half LIM domains 1
Malignant neoplasm of breast	RAB31	Q13636	RAB31, member RAS oncogene family
Malignant neoplasm of breast	PTCH1	Q13635	patched 1
Malignant neoplasm of breast	TSTA3	Q13630	tissue specific transplantation antigen P35B
Malignant neoplasm of breast	DYRK1A	Q13627	dual specificity tyrosine phosphorylation regulated kinase 1A
Malignant neoplasm of breast	TP53BP2	Q13625	tumor protein p53 binding protein 2
Malignant neoplasm of breast	CUL4B	Q13620	cullin 4B
Malignant neoplasm of breast	CUL4A	Q13619	cullin 4A
Malignant neoplasm of breast	CUL3	Q13618	cullin 3
Malignant neoplasm of breast	CUL2	Q13617	cullin 2

Malignant neoplasm of breast	CUL1	Q13616	cullin 1
Malignant neoplasm of breast	MTMR3	Q13615	myotubularin related protein 3
Malignant neoplasm of breast	DNASE1L3	Q13609	deoxyribonuclease 1 like 3
Malignant neoplasm of breast	KRR1	Q13601	KRR1 small subunit processome component homolog
Malignant neoplasm of breast	TRA2A	Q13595	transformer 2 alpha homolog
Malignant neoplasm of breast	STIM1	Q13586	stromal interaction molecule 1
Malignant neoplasm of breast	GPR50	Q13585	G protein-coupled receptor 50
Malignant neoplasm of breast	SNW1	Q13573	SNW domain containing 1
Malignant neoplasm of breast	TDG	Q13569	thymine DNA glycosylase
Malignant neoplasm of breast	IRF5	Q13568	interferon regulatory factor 5
Malignant neoplasm of breast	PKD2	Q13563	polycystin 2, transient receptor potential cation channel
Malignant neoplasm of breast	NEUROD1	Q13562	neuronal differentiation 1
Malignant neoplasm of breast	CAMK2B	Q13554	calcium/calmodulin dependent protein kinase II beta
Malignant neoplasm of breast	HDAC1	Q13547	histone deacetylase 1
Malignant neoplasm of breast	RIPK1	Q13546	receptor interacting serine/threonine kinase 1
Malignant neoplasm of breast	EIF4EBP1	Q13541	eukaryotic translation initiation factor 4E binding protein 1
Malignant neoplasm of breast	ATR	Q13535	ATR serine/threonine kinase
Malignant neoplasm of breast	PIN1	Q13526	peptidylprolyl cis/trans isomerase, NIMA-interacting 1
Malignant neoplasm of breast	PRPF4B	Q13523	pre-mRNA processing factor 4B

Malignant neoplasm of breast	PPP1R1A	Q13522	protein phosphatase 1 regulatory inhibitor subunit 1A
Malignant neoplasm of breast	ASAH1	Q13510	N-acylsphingosine amidohydrolase 1
Malignant neoplasm of breast	TUBB3	Q13509	tubulin beta 3 class III
Malignant neoplasm of breast	TRPC3	Q13507	transient receptor potential cation channel subfamily C member 3
Malignant neoplasm of breast	MTX1	Q13505	metaxin 1
Malignant neoplasm of breast	SQSTM1	Q13501	sequestosome 1
Malignant neoplasm of breast	MTM1	Q13496	myotubularin 1
Malignant neoplasm of breast	GPM6B	Q13491	glycoprotein M6B
Malignant neoplasm of breast	BIRC2	Q13490	baculoviral IAP repeat containing 2
Malignant neoplasm of breast	BIRC3	Q13489	baculoviral IAP repeat containing 3
Malignant neoplasm of breast	SMAD4	Q13485	SMAD family member 4
Malignant neoplasm of breast	GAB1	Q13480	GRB2 associated binding protein 1
Malignant neoplasm of breast	IL18R1	Q13478	interleukin 18 receptor 1
Malignant neoplasm of breast	TOP3A	Q13472	DNA topoisomerase III alpha
Malignant neoplasm of breast	NFATC2	Q13469	nuclear factor of activated T cells 2
Malignant neoplasm of breast	FZD5	Q13467	frizzled class receptor 5
Malignant neoplasm of breast	ROCK1	Q13464	Rho associated coiled-coil containing protein kinase 1
Malignant neoplasm of breast	MYO9B	Q13459	myosin IXB
Malignant neoplasm of breast	TUSC3	Q13454	tumor suppressor candidate 3

Malignant neoplasm of breast	TMED1	Q13445	transmembrane p24 trafficking protein 1
Malignant neoplasm of breast	ADAM15	Q13444	ADAM metallopeptidase domain 15
Malignant neoplasm of breast	ADAM9	Q13443	ADAM metallopeptidase domain 9
Malignant neoplasm of breast	SLC39A6	Q13433	solute carrier family 39 member 6
Malignant neoplasm of breast	PPIG	Q13427	peptidylprolyl isomerase G
Malignant neoplasm of breast	XRCC4	Q13426	X-ray repair cross complementing 4
Malignant neoplasm of breast	SNTA1	Q13424	syntrophin alpha 1
Malignant neoplasm of breast	NNT	Q13423	nicotinamide nucleotide transhydrogenase
Malignant neoplasm of breast	MSLN	Q13421	mesothelin
Malignant neoplasm of breast	ILK	Q13418	integrin linked kinase
Malignant neoplasm of breast	PLD1	Q13393	phospholipase D1
Malignant neoplasm of breast	CTBP1	Q13363	C-terminal binding protein 1
Malignant neoplasm of breast	MFAP5	Q13361	microfibril associated protein 5
Malignant neoplasm of breast	PPIL2	Q13356	peptidylprolyl isomerase like 2
Malignant neoplasm of breast	ITGB3BP	Q13352	integrin subunit beta 3 binding protein
Malignant neoplasm of breast	MTA1	Q13330	metastasis associated 1
Malignant neoplasm of breast	SGCG	Q13326	sarcoglycan gamma
Malignant neoplasm of breast	IFIT5	Q13325	interferon induced protein with tetratricopeptide repeats 5
Malignant neoplasm of breast	BIK	Q13323	BCL2 interacting killer

Malignant neoplasm of breast	DMP1	Q13316	dentin matrix acidic phosphoprotein 1
Malignant neoplasm of breast	ATM	Q13315	ATM serine/threonine kinase
Malignant neoplasm of breast	SKP2	Q13309	S-phase kinase associated protein 2
Malignant neoplasm of breast	PTK7	Q13308	protein tyrosine kinase 7 (inactive)
Malignant neoplasm of breast	GPR17	Q13304	G protein-coupled receptor 17
Malignant neoplasm of breast	SCGB2A2	Q13296	secretoglobin family 2A member 2
Malignant neoplasm of breast	SLAMF1	Q13291	signaling lymphocytic activation molecule family member 1
Malignant neoplasm of breast	NMI	Q13287	N-myc and STAT interactor
Malignant neoplasm of breast	CLN3	Q13286	CLN3 lysosomal/endosomal transmembrane protein, battenin
Malignant neoplasm of breast	G3BP1	Q13283	G3BP stress granule assembly factor 1
Malignant neoplasm of breast	STX3	Q13277	syntaxin 3
Malignant neoplasm of breast	SEMA3F	Q13275	semaphorin 3F
Malignant neoplasm of breast	DHRS2	Q13268	dehydrogenase/reductase 2
Malignant neoplasm of breast	TRIM28	Q13263	tripartite motif containing 28
Malignant neoplasm of breast	IL15RA	Q13261	interleukin 15 receptor subunit alpha
Malignant neoplasm of breast	MAD2L1	Q13257	mitotic arrest deficient 2 like 1
Malignant neoplasm of breast	GRM1	Q13255	glutamate metabotropic receptor 1
Malignant neoplasm of breast	NOG	Q13253	noggin
Malignant neoplasm of breast	SRSF6	Q13247	serine and arginine rich splicing factor 6

Malignant neoplasm of breast	SRSF5	Q13243	serine and arginine rich splicing factor 5
Malignant neoplasm of breast	MAP3K1	Q13233	mitogen-activated protein kinase kinase kinase 1
Malignant neoplasm of breast	CHIT1	Q13231	chitinase 1
Malignant neoplasm of breast	SELENBP1	Q13228	selenium binding protein 1
Malignant neoplasm of breast	PAPPA	Q13219	pappalysin 1
Malignant neoplasm of breast	DNAJC3	Q13217	DnaJ heat shock protein family (Hsp40) member C3
Malignant neoplasm of breast	SEMA3B	Q13214	semaphorin 3B
Malignant neoplasm of breast	TBX2	Q13207	T-box transcription factor 2
Malignant neoplasm of breast	DDX10	Q13206	DEAD-box helicase 10
Malignant neoplasm of breast	DUSP8	Q13202	dual specificity phosphatase 8
Malignant neoplasm of breast	MMRN1	Q13201	multimerin 1
Malignant neoplasm of breast	PSMD2	Q13200	proteasome 26S subunit, non-ATPase 2
Malignant neoplasm of breast	STX5	Q13190	syntaxin 5
Malignant neoplasm of breast	STK3	Q13188	serine/threonine kinase 3
Malignant neoplasm of breast	CBX3	Q13185	chromobox 3
Malignant neoplasm of breast	SLC13A2	Q13183	solute carrier family 13 member 2
Malignant neoplasm of breast	PAK2	Q13177	p21 (RAC1) activated kinase 2
Malignant neoplasm of breast	MAPK7	Q13164	mitogen-activated protein kinase 7
Malignant neoplasm of breast	MAP2K5	Q13163	mitogen-activated protein kinase kinase 5

Malignant neoplasm of breast	PRDX4	Q13162	peroxiredoxin 4
Malignant neoplasm of breast	FADD	Q13158	Fas associated via death domain
Malignant neoplasm of breast	AIMP2	Q13155	aminoacyl tRNA synthetase complex interacting multifunctional protein 2
Malignant neoplasm of breast	PAK1	Q13153	p21 (RAC1) activated kinase 1
Malignant neoplasm of breast	TARDBP	Q13148	TAR DNA binding protein
Malignant neoplasm of breast	EIF2B5	Q13144	eukaryotic translation initiation factor 2B subunit epsilon
Malignant neoplasm of breast	PPFIA1	Q13136	PTPRF interacting protein alpha 1
Malignant neoplasm of breast	PRKAA1	Q13131	protein kinase AMP-activated catalytic subunit alpha 1
Malignant neoplasm of breast	REST	Q13127	RE1 silencing transcription factor
Malignant neoplasm of breast	MTAP	Q13126	methylthioadenosine phosphorylase
Malignant neoplasm of breast	KLF10	Q13118	Kruppel like factor 10
Malignant neoplasm of breast	DUSP4	Q13115	dual specificity phosphatase 4
Malignant neoplasm of breast	TRAF3	Q13114	TNF receptor associated factor 3
Malignant neoplasm of breast	PDZK1IP1	Q13113	PDZK1 interacting protein 1
Malignant neoplasm of breast	CHAF1B	Q13112	chromatin assembly factor 1 subunit B
Malignant neoplasm of breast	CHAF1A	Q13111	chromatin assembly factor 1 subunit A
Malignant neoplasm of breast	USP4	Q13107	ubiquitin specific peptidase 4
Malignant neoplasm of breast	PDIA2	Q13087	protein disulfide isomerase family A member 2
Malignant neoplasm of breast	ACACA	Q13085	acetyl-CoA carboxylase alpha

Malignant neoplasm of breast	MRPL28	Q13084	mitochondrial ribosomal protein L28
Malignant neoplasm of breast	TRAF1	Q13077	TNF receptor associated factor 1
Malignant neoplasm of breast	NAIP	Q13075	NLR family apoptosis inhibitory protein
Malignant neoplasm of breast	BAGE	Q13072	B melanoma antigen
Malignant neoplasm of breast	COASY	Q13057	Coenzyme A synthase
Malignant neoplasm of breast	TRIM32	Q13049	tripartite motif containing 32
Malignant neoplasm of breast	FLII	Q13045	FLII actin remodeling protein
Malignant neoplasm of breast	STK4	Q13043	serine/threonine kinase 4
Malignant neoplasm of breast	PRDM2	Q13029	PR/SET domain 2
Malignant neoplasm of breast	AKAP6	Q13023	A-kinase anchoring protein 6
Malignant neoplasm of breast	PLA2R1	Q13018	phospholipase A2 receptor 1
Malignant neoplasm of breast	ARHGAP5	Q13017	Rho GTPase activating protein 5
Malignant neoplasm of breast	MLLT11	Q13015	MLLT11 transcription factor 7 cofactor
Malignant neoplasm of breast	TIAM1	Q13009	TIAM Rac1 associated GEF 1
Malignant neoplasm of breast	IL24	Q13007	interleukin 24
Malignant neoplasm of breast	GRIK3	Q13003	glutamate ionotropic receptor kainate type subunit 3
Malignant neoplasm of breast	GRIK2	Q13002	glutamate ionotropic receptor kainate type subunit 2
Malignant neoplasm of breast	TSPAN31	Q12999	tetraspanin 31
Malignant neoplasm of breast	HSPB3	Q12988	heat shock protein family B (small) member 3

Malignant neoplasm of breast	BNIP3	Q12983	BCL2 interacting protein 3
Malignant neoplasm of breast	BNIP1	Q12981	BCL2 interacting protein 1
Malignant neoplasm of breast	ABR	Q12979	ABR activator of RhoGEF and GTPase
Malignant neoplasm of breast	PTP4A2	Q12974	protein tyrosine phosphatase 4A2
Malignant neoplasm of breast	PPP1R8	Q12972	protein phosphatase 1 regulatory subunit 8
Malignant neoplasm of breast	ANK3	Q12955	ankyrin 3
Malignant neoplasm of breast	FOXL1	Q12952	forkhead box L1
Malignant neoplasm of breast	FOXC1	Q12948	forkhead box C1
Malignant neoplasm of breast	FOXF2	Q12947	forkhead box F2
Malignant neoplasm of breast	FOXF1	Q12946	forkhead box F1
Malignant neoplasm of breast	TRAF2	Q12933	TNF receptor associated factor 2
Malignant neoplasm of breast	TRAP1	Q12931	TNF receptor associated protein 1
Malignant neoplasm of breast	EPS8	Q12929	epidermal growth factor receptor pathway substrate 8
Malignant neoplasm of breast	ELAVL2	Q12926	ELAV like RNA binding protein 2
Malignant neoplasm of breast	PTPN13	Q12923	protein tyrosine phosphatase non-receptor type 13
Malignant neoplasm of breast	PTPRJ	Q12913	protein tyrosine phosphatase receptor type J
Malignant neoplasm of breast	ILF3	Q12906	interleukin enhancer binding factor 3
Malignant neoplasm of breast	ILF2	Q12905	interleukin enhancer binding factor 2
Malignant neoplasm of breast	HYAL2	Q12891	hyaluronidase 2

Malignant neoplasm of breast	TP53BP1	Q12888	tumor protein p53 binding protein 1
Malignant neoplasm of breast	FAP	Q12884	fibroblast activation protein alpha
Malignant neoplasm of breast	DPYD	Q12882	dihydropyrimidine dehydrogenase
Malignant neoplasm of breast	CHD3	Q12873	chromodomain helicase DNA binding protein 3
Malignant neoplasm of breast	MERTK	Q12866	MER proto-oncogene, tyrosine kinase
Malignant neoplasm of breast	CDH17	Q12864	cadherin 17
Malignant neoplasm of breast	CNTN1	Q12860	contactin 1
Malignant neoplasm of breast	MAP3K12	Q12852	mitogen-activated protein kinase kinase kinase 12
Malignant neoplasm of breast	FSTL1	Q12841	follistatin like 1
Malignant neoplasm of breast	KIF5A	Q12840	kinesin family member 5A
Malignant neoplasm of breast	POU4F2	Q12837	POU class 4 homeobox 2
Malignant neoplasm of breast	CDC20	Q12834	cell division cycle 20
Malignant neoplasm of breast	RAB40B	Q12829	RAB40B, member RAS oncogene family
Malignant neoplasm of breast	SMARCB1	Q12824	SWI/SNF related, matrix associated, actin dependent regulator of chromatin, subfamily b, member 1
Malignant neoplasm of breast	TRO	Q12816	trophinin
Malignant neoplasm of breast	TROAP	Q12815	trophinin associated protein
Malignant neoplasm of breast	KCNH2	Q12809	potassium voltage-gated channel subfamily H member 2
Malignant neoplasm of breast	EFEMP1	Q12805	EGF containing fibulin extracellular matrix protein 1
Malignant neoplasm of breast	AKAP13	Q12802	A-kinase anchoring protein 13

Malignant neoplasm of breast	TFCP2	Q12800	transcription factor CP2
Malignant neoplasm of breast	CETN1	Q12798	centrin 1
Malignant neoplasm of breast	ASPH	Q12797	aspartate beta-hydroxylase
Malignant neoplasm of breast	HYAL1	Q12794	hyaluronidase 1
Malignant neoplasm of breast	TWF1	Q12792	twinfilin actin binding protein 1
Malignant neoplasm of breast	KCNMA1	Q12791	potassium calcium-activated channel subfamily M alpha 1
Malignant neoplasm of breast	FOXO1	Q12778	forkhead box O1
Malignant neoplasm of breast	ARHGEF5	Q12774	Rho guanine nucleotide exchange factor 5
Malignant neoplasm of breast	SREBF2	Q12772	sterol regulatory element binding transcription factor 2
Malignant neoplasm of breast	SCAP	Q12770	SREBF chaperone
Malignant neoplasm of breast	KIF1A	Q12756	kinesin family member 1A
Malignant neoplasm of breast	ST3GAL3	Q11203	ST3 beta-galactoside alpha-2,3-sialyltransferase 3
Malignant neoplasm of breast	ST3GAL1	Q11201	ST3 beta-galactoside alpha-2,3-sialyltransferase 1
Malignant neoplasm of breast	FUT2	Q10981	fucosyltransferase 2
Malignant neoplasm of breast	KLKP1	Q107X0	kallikrein pseudogene 1
Malignant neoplasm of breast	BST2	Q10589	bone marrow stromal cell antigen 2
Malignant neoplasm of breast	USP17L24	Q0WX57	ubiquitin specific peptidase 17 like family member 24
Malignant neoplasm of breast	USP17L25	Q0WX57	ubiquitin specific peptidase 17 like family member 25
Malignant neoplasm of breast	USP17L26	Q0WX57	ubiquitin specific peptidase 17 like family member 26

Malignant neoplasm of breast	USP17L27	Q0WX57	ubiquitin specific peptidase 17 like family member 27
Malignant neoplasm of breast	USP17L28	Q0WX57	ubiquitin specific peptidase 17 like family member 28
Malignant neoplasm of breast	USP17L29	Q0WX57	ubiquitin specific peptidase 17 like family member 29
Malignant neoplasm of breast	USP17L30	Q0WX57	ubiquitin specific peptidase 17 like family member 30
Malignant neoplasm of breast	HSPA14	Q0VDF9	heat shock protein family A (Hsp70) member 14
Malignant neoplasm of breast	MIR22HG	Q0VDD5	MIR22 host gene
Malignant neoplasm of breast	ZNF763	Q0D2J5	zinc finger protein 763
Malignant neoplasm of breast	ZNF415	Q09FC8	zinc finger protein 415
Malignant neoplasm of breast	AHNAK	Q09666	AHNAK nucleoprotein
Malignant neoplasm of breast	EP300	Q09472	E1A binding protein p300
Malignant neoplasm of breast	KCNA1	Q09470	potassium voltage-gated channel subfamily A member 1
Malignant neoplasm of breast	ABCC8	Q09428	ATP binding cassette subfamily C member 8
Malignant neoplasm of breast	MGAT5	Q09328	alpha-1,6-mannosylglycoprotein 6-beta-N-acetylglucosaminyltransferase
Malignant neoplasm of breast	MGAT3	Q09327	beta-1,4-mannosyl-glycoprotein 4-beta-N-acetylglucosaminyltransferase
Malignant neoplasm of breast	NSUN2	Q08J23	NOP2/Sun RNA methyltransferase 2
Malignant neoplasm of breast	VAC14	Q08AM6	VAC14 component of PIKFYVE complex
Malignant neoplasm of breast	SLFN5	Q08AF3	schlafen family member 5
Malignant neoplasm of breast	RBL2	Q08999	RB transcriptional corepressor like 2
Malignant neoplasm of breast	SSRP1	Q08945	structure specific recognition protein 1

Malignant neoplasm of breast	ITK	Q08881	IL2 inducible T cell kinase
Malignant neoplasm of breast	FGL1	Q08830	fibrinogen like 1
Malignant neoplasm of breast	PPID	Q08752	peptidylprolyl isomerase D
Malignant neoplasm of breast	CD47	Q08722	CD47 molecule
Malignant neoplasm of breast	SPOCK1	Q08629	SPARC (osteonectin), cwcv and kazal like domains proteoglycan 1
Malignant neoplasm of breast	DSC1	Q08554	desmocollin 1
Malignant neoplasm of breast	PDE4D	Q08499	phosphodiesterase 4D
Malignant neoplasm of breast	CYP4F3	Q08477	cytochrome P450 family 4 subfamily F member 3
Malignant neoplasm of breast	MFGE8	Q08431	milk fat globule-EGF factor 8 protein
Malignant neoplasm of breast	LOXL1	Q08397	lysyl oxidase like 1
Malignant neoplasm of breast	LGALS3BP	Q08380	galectin 3 binding protein
Malignant neoplasm of breast	GOLGA2	Q08379	golgin A2
Malignant neoplasm of breast	DDR1	Q08345	discoidin domain receptor tyrosine kinase 1
Malignant neoplasm of breast	CRYZ	Q08257	crystallin zeta
Malignant neoplasm of breast	DHX9	Q08211	DExH-box helicase 9
Malignant neoplasm of breast	PPP3CA	Q08209	protein phosphatase 3 catalytic subunit alpha
Malignant neoplasm of breast	FOXM1	Q08050	forkhead box M1
Malignant neoplasm of breast	CYP24A1	Q07973	cytochrome P450 family 24 subfamily A member 1
Malignant neoplasm of breast	ARHGAP1	Q07960	Rho GTPase activating protein 1

Malignant neoplasm of breast	SRSF1	Q07955	serine and arginine rich splicing factor 1
Malignant neoplasm of breast	LRP1	Q07954	LDL receptor related protein 1
Malignant neoplasm of breast	TNK2	Q07912	tyrosine kinase non receptor 2
Malignant neoplasm of breast	SOS1	Q07889	SOS Ras/Rac guanine nucleotide exchange factor 1
Malignant neoplasm of breast	PPARA	Q07869	peroxisome proliferator activated receptor alpha
Malignant neoplasm of breast	KLC1	Q07866	kinesin light chain 1
Malignant neoplasm of breast	POLE	Q07864	DNA polymerase epsilon, catalytic subunit
Malignant neoplasm of breast	SLC3A1	Q07837	solute carrier family 3 member 1
Malignant neoplasm of breast	MCL1	Q07820	MCL1 apoptosis regulator, BCL2 family member
Malignant neoplasm of breast	BCL2L1	Q07817	BCL2 like 1
Malignant neoplasm of breast	BAX	Q07812	BCL2 associated X, apoptosis regulator
Malignant neoplasm of breast	SCN1B	Q07699	sodium voltage-gated channel beta subunit 1
Malignant neoplasm of breast	DLX2	Q07687	distal-less homeobox 2
Malignant neoplasm of breast	KHDRBS1	Q07666	KH RNA binding domain containing, signal transduction associated 1
Malignant neoplasm of breast	TFF3	Q07654	trefoil factor 3
Malignant neoplasm of breast	DPT	Q07507	dermatopontin
Malignant neoplasm of breast	ZFP36L1	Q07352	ZFP36 ring finger protein like 1
Malignant neoplasm of breast	PDE4B	Q07343	phosphodiesterase 4B
Malignant neoplasm of breast	CXCL9	Q07325	C-X-C motif chemokine ligand 9

Malignant neoplasm of breast	TJP1	Q07157	tight junction protein 1
Malignant neoplasm of breast	CD69	Q07108	CD69 molecule
Malignant neoplasm of breast	ENPEP	Q07075	glutamyl aminopeptidase
Malignant neoplasm of breast	CKAP4	Q07065	cytoskeleton associated protein 4
Malignant neoplasm of breast	C1QBP	Q07021	complement C1q binding protein
Malignant neoplasm of breast	TNFRSF9	Q07011	TNF receptor superfamily member 9
Malignant neoplasm of breast	CDK18	Q07002	cyclin dependent kinase 18
Malignant neoplasm of breast	CHRND	Q07001	cholinergic receptor nicotinic delta subunit
Malignant neoplasm of breast	SOX4	Q06945	SRY-box transcription factor 4
Malignant neoplasm of breast	EGR3	Q06889	early growth response 3
Malignant neoplasm of breast	PRDX1	Q06830	peroxiredoxin 1
Malignant neoplasm of breast	FMOD	Q06828	fibromodulin
Malignant neoplasm of breast	FMR1	Q06787	FMRP translational regulator 1
Malignant neoplasm of breast	PAX8	Q06710	paired box 8
Malignant neoplasm of breast	LTB	Q06643	lymphotoxin beta
Malignant neoplasm of breast	RAD51	Q06609	RAD51 recombinase
Malignant neoplasm of breast	GABPA	Q06546	GA binding protein transcription factor subunit alpha
Malignant neoplasm of breast	SULT2A1	Q06520	sulfotransferase family 2A member 1
Malignant neoplasm of breast	RUNX1T1	Q06455	RUNX1 partner transcriptional co-repressor 1

Malignant neoplasm of breast	TYRO3	Q06418	TYRO3 protein tyrosine kinase
Malignant neoplasm of breast	POU5F1B	Q06416	POU class 5 homeobox 1B
Malignant neoplasm of breast	RBPJ	Q06330	recombination signal binding protein for immunoglobulin kappa J region
Malignant neoplasm of breast	GFPT1	Q06210	glutamine--fructose-6-phosphate transaminase 1
Malignant neoplasm of breast	PPAT	Q06203	phosphoribosyl pyrophosphate amidotransferase
Malignant neoplasm of breast	BTK	Q06187	Bruton tyrosine kinase
Malignant neoplasm of breast	REG3A	Q06141	regenerating family member 3 alpha
Malignant neoplasm of breast	PTPN11	Q06124	protein tyrosine phosphatase non-receptor type 11
Malignant neoplasm of breast	DUSP2	Q05923	dual specificity phosphatase 2
Malignant neoplasm of breast	RNASEL	Q05823	ribonuclease L
Malignant neoplasm of breast	CALD1	Q05682	caldesmon 1
Malignant neoplasm of breast	PRKCD	Q05655	protein kinase C delta
Malignant neoplasm of breast	EEF1A2	Q05639	eukaryotic translation elongation factor 1 alpha 2
Malignant neoplasm of breast	GRIN1	Q05586	glutamate ionotropic receptor NMDA type subunit 1
Malignant neoplasm of breast	PRKCZ	Q05513	protein kinase C zeta
Malignant neoplasm of breast	LIPE	Q05469	lipase E, hormone sensitive type
Malignant neoplasm of breast	PTK2	Q05397	protein tyrosine kinase 2
Malignant neoplasm of breast	GAD2	Q05329	glutamate decarboxylase 2
Malignant neoplasm of breast	PTPN12	Q05209	protein tyrosine phosphatase non-receptor type 12

Malignant neoplasm of breast	MXD1	Q05195	MAX dimerization protein 1
Malignant neoplasm of breast	DNM1	Q05193	dynamin 1
Malignant neoplasm of breast	UBE3A	Q05086	ubiquitin protein ligase E3A
Malignant neoplasm of breast	SRY	Q05066	sex determining region Y
Malignant neoplasm of breast	CSTF1	Q05048	cleavage stimulation factor subunit 1
Malignant neoplasm of breast	PLP2	Q04941	proteolipid protein 2
Malignant neoplasm of breast	MST1R	Q04912	macrophage stimulating 1 receptor
Malignant neoplasm of breast	AKR1C1	Q04828	aldo-keto reductase family 1 member C1
Malignant neoplasm of breast	ACVR1	Q04771	activin A receptor type 1
Malignant neoplasm of breast	GLO1	Q04760	glyoxalase I
Malignant neoplasm of breast	PRKCQ	Q04759	protein kinase C theta
Malignant neoplasm of breast	TLE4	Q04727	TLE family member 4, transcriptional corepressor
Malignant neoplasm of breast	TLE3	Q04726	TLE family member 3, transcriptional corepressor
Malignant neoplasm of breast	TLE1	Q04724	TLE family member 1, transcriptional corepressor
Malignant neoplasm of breast	NOTCH2	Q04721	notch receptor 2
Malignant neoplasm of breast	KRT17	Q04695	keratin 17
Malignant neoplasm of breast	OCA2	Q04671	OCA2 melanosomal transmembrane protein
Malignant neoplasm of breast	ATP7A	Q04656	ATPase copper transporting alpha
Malignant neoplasm of breast	EIF4G1	Q04637	eukaryotic translation initiation factor 4 gamma 1

Malignant neoplasm of breast	FOLH1	Q04609	folate hydrolase 1
Malignant neoplasm of breast	RELA	Q04206	RELA proto-oncogene, NF-kB subunit
Malignant neoplasm of breast	COL10A1	Q03692	collagen type X alpha 1 chain
Malignant neoplasm of breast	CFHR1	Q03591	complement factor H related 1
Malignant neoplasm of breast	TAP2	Q03519	transporter 2, ATP binding cassette subfamily B member
Malignant neoplasm of breast	TAP1	Q03518	transporter 1, ATP binding cassette subfamily B member
Malignant neoplasm of breast	PTH1R	Q03431	parathyroid hormone 1 receptor
Malignant neoplasm of breast	PLAUR	Q03405	plasminogen activator, urokinase receptor
Malignant neoplasm of breast	TFF2	Q03403	trefoil factor 2
Malignant neoplasm of breast	ROM1	Q03395	retinal outer segment membrane protein 1
Malignant neoplasm of breast	PPARD	Q03181	peroxisome proliferator activated receptor delta
Malignant neoplasm of breast	TNFAIP2	Q03169	TNF alpha induced protein 2
Malignant neoplasm of breast	TGFBR3	Q03167	transforming growth factor beta receptor 3
Malignant neoplasm of breast	KMT2A	Q03164	lysine methyltransferase 2A
Malignant neoplasm of breast	ACY1	Q03154	aminoacylase 1
Malignant neoplasm of breast	CAV1	Q03135	caveolin 1
Malignant neoplasm of breast	GNA12	Q03113	G protein subunit alpha 12
Malignant neoplasm of breast	MECOM	Q03112	MDS1 and EVI1 complex locus
Malignant neoplasm of breast	CREM	Q03060	cAMP responsive element modulator

Malignant neoplasm of breast	HHEX	Q03014	hematopoietically expressed homeobox
Malignant neoplasm of breast	GSTM4	Q03013	glutathione S-transferase mu 4
Malignant neoplasm of breast	DST	Q03001	dystonin
Malignant neoplasm of breast	PAX2	Q02962	paired box 2
Malignant neoplasm of breast	AKAP12	Q02952	A-kinase anchoring protein 12
Malignant neoplasm of breast	TOP2B	Q02880	DNA topoisomerase II beta
Malignant neoplasm of breast	GUCY2D	Q02846	guanylate cyclase 2D, retinal
Malignant neoplasm of breast	RASSF7	Q02833	Ras association domain family member 7
Malignant neoplasm of breast	MUC2	Q02817	mucin 2, oligomeric mucus/gel-forming
Malignant neoplasm of breast	PLOD1	Q02809	procollagen-lysine,2-oxoglutarate 5-dioxygenase 1
Malignant neoplasm of breast	FKBP4	Q02790	FKBP prolyl isomerase 4
Malignant neoplasm of breast	MAP3K10	Q02779	mitogen-activated protein kinase kinase kinase 10
Malignant neoplasm of breast	TEK	Q02763	TEK receptor tyrosine kinase
Malignant neoplasm of breast	MAP2K1	Q02750	mitogen-activated protein kinase kinase 1
Malignant neoplasm of breast	GHRHR	Q02643	growth hormone releasing hormone receptor
Malignant neoplasm of breast	IRF8	Q02556	interferon regulatory factor 8
Malignant neoplasm of breast	PAX5	Q02548	paired box 5
Malignant neoplasm of breast	MUC3A	Q02505	mucin 3A, cell surface associated
Malignant neoplasm of breast	DSC2	Q02487	desmocollin 2

Malignant neoplasm of breast	COL7A1	Q02388	collagen type VII alpha 1 chain
Malignant neoplasm of breast	ID2	Q02363	inhibitor of DNA binding 2
Malignant neoplasm of breast	BDH1	Q02338	3-hydroxybutyrate dehydrogenase 1
Malignant neoplasm of breast	CYP27A1	Q02318	cytochrome P450 family 27 subfamily A member 1
Malignant neoplasm of breast	NRG1	Q02297	neuregulin 1
Malignant neoplasm of breast	KIF23	Q02241	kinesin family member 23
Malignant neoplasm of breast	CENPE	Q02224	centromere protein E
Malignant neoplasm of breast	TNFRSF17	Q02223	TNF receptor superfamily member 17
Malignant neoplasm of breast	OGDH	Q02218	oxoglutarate dehydrogenase
Malignant neoplasm of breast	RHD	Q02161	Rh blood group D antigen
Malignant neoplasm of breast	PRKCE	Q02156	protein kinase C epsilon
Malignant neoplasm of breast	GUCY1B1	Q02153	guanylate cyclase 1 soluble subunit beta 1
Malignant neoplasm of breast	DHODH	Q02127	dihydroorotate dehydrogenase (quinone)
Malignant neoplasm of breast	RHAG	Q02094	Rh associated glycoprotein
Malignant neoplasm of breast	MEF2B	Q02080	myocyte enhancer factor 2B
Malignant neoplasm of breast	MEF2A	Q02078	myocyte enhancer factor 2A
Malignant neoplasm of breast	ROR2	Q01974	receptor tyrosine kinase like orphan receptor 2
Malignant neoplasm of breast	ROR1	Q01973	receptor tyrosine kinase like orphan receptor 1
Malignant neoplasm of breast	SLC6A3	Q01959	solute carrier family 6 member 3

Malignant neoplasm of breast	BNC1	Q01954	basonuclin 1
Malignant neoplasm of breast	POU5F1	Q01860	POU class 5 homeobox 1
Malignant neoplasm of breast	EWSR1	Q01844	EWS RNA binding protein 1
Malignant neoplasm of breast	XPC	Q01831	XPC complex subunit, DNA damage recognition and repair factor
Malignant neoplasm of breast	SATB1	Q01826	SATB homeobox 1
Malignant neoplasm of breast	ATP2B2	Q01814	ATPase plasma membrane Ca2+ transporting 2
Malignant neoplasm of breast	PFKP	Q01813	phosphofructokinase, platelet
Malignant neoplasm of breast	OTUD4	Q01804	OTU deubiquitinase 4
Malignant neoplasm of breast	MC1R	Q01726	melanocortin 1 receptor
Malignant neoplasm of breast	SLC7A5	Q01650	solute carrier family 7 member 5
Malignant neoplasm of breast	IFITM3	Q01628	interferon induced transmembrane protein 3
Malignant neoplasm of breast	KRT76	Q01546	keratin 76
Malignant neoplasm of breast	FLI1	Q01543	Fli-1 proto-oncogene, ETS transcription factor
Malignant neoplasm of breast	CAP1	Q01518	cyclase associated actin cytoskeleton regulatory protein 1
Malignant neoplasm of breast	ANK2	Q01484	ankyrin 2
Malignant neoplasm of breast	FABP5	Q01469	fatty acid binding protein 5
Malignant neoplasm of breast	PMP22	Q01453	peripheral myelin protein 22
Malignant neoplasm of breast	RELB	Q01201	RELB proto-oncogene, NF-kB subunit
Malignant neoplasm of breast	RUNX1	Q01196	RUNX family transcription factor 1

Malignant neoplasm of breast	FOXK2	Q01167	forkhead box K2
Malignant neoplasm of breast	CD83	Q01151	CD83 molecule
Malignant neoplasm of breast	SRSF2	Q01130	serine and arginine rich splicing factor 2
Malignant neoplasm of breast	SET	Q01105	SET nuclear proto-oncogene
Malignant neoplasm of breast	E2F1	Q01094	E2F transcription factor 1
Malignant neoplasm of breast	TIAL1	Q01085	TIA1 cytotoxic granule associated RNA binding protein like 1
Malignant neoplasm of breast	MDM2	Q00987	MDM2 proto-oncogene
Malignant neoplasm of breast	IRF9	Q00978	interferon regulatory factor 9
Malignant neoplasm of breast	HNRNPU	Q00839	heterogeneous nuclear ribonucleoprotein U
Malignant neoplasm of breast	SORD	Q00796	sorbitol dehydrogenase
Malignant neoplasm of breast	PLCB2	Q00722	phospholipase C beta 2
Malignant neoplasm of breast	NFKB2	Q00653	nuclear factor kappa B subunit 2
Malignant neoplasm of breast	HSF1	Q00613	heat shock transcription factor 1
Malignant neoplasm of breast	CLTC	Q00610	clathrin heavy chain
Malignant neoplasm of breast	NDP	Q00604	norrin cystine knot growth factor NDP
Malignant neoplasm of breast	FANCC	Q00597	FA complementation group C
Malignant neoplasm of breast	CDK16	Q00536	cyclin dependent kinase 16
Malignant neoplasm of breast	CDK5	Q00535	cyclin dependent kinase 5
Malignant neoplasm of breast	CDK6	Q00534	cyclin dependent kinase 6

Malignant neoplasm of breast	CDKL1	Q00532	cyclin dependent kinase like 1
Malignant neoplasm of breast	CDK3	Q00526	cyclin dependent kinase 3
Malignant neoplasm of breast	HDLBP	Q00341	high density lipoprotein binding protein
Malignant neoplasm of breast	SLC25A3	Q00325	solute carrier family 25 member 3
Malignant neoplasm of breast	MAT1A	Q00266	methionine adenosyltransferase 1A
Malignant neoplasm of breast	TFAM	Q00059	transcription factor A, mitochondrial
Malignant neoplasm of breast	HOXA4	Q00056	homeobox A4
Malignant neoplasm of breast	PPP2R2B	Q00005	protein phosphatase 2 regulatory subunit Bbeta
Malignant neoplasm of breast	CYCS	P99999	cytochrome c, somatic
Malignant neoplasm of breast	RBM3	P98179	RNA binding motif protein 3
Malignant neoplasm of breast	RBM10	P98175	RNA binding motif protein 10
Malignant neoplasm of breast	FGD1	P98174	FYVE, RhoGEF and PH domain containing 1
Malignant neoplasm of breast	XIAP	P98170	X-linked inhibitor of apoptosis
Malignant neoplasm of breast	LRP2	P98164	LDL receptor related protein 2
Malignant neoplasm of breast	PKD1	P98161	polycystin 1, transient receptor potential channel interacting
Malignant neoplasm of breast	HSPG2	P98160	heparan sulfate proteoglycan 2
Malignant neoplasm of breast	VLDLR	P98155	very low density lipoprotein receptor
Malignant neoplasm of breast	FBLN2	P98095	fibulin 2
Malignant neoplasm of breast	MUC5AC	P98088	mucin 5AC, oligomeric mucus/gel-forming

Malignant neoplasm of breast	DAB2	P98082	DAB adaptor protein 2
Malignant neoplasm of breast	SHC2	P98077	SHC adaptor protein 2
Malignant neoplasm of breast	TNFAIP6	P98066	TNF alpha induced protein 6
Malignant neoplasm of breast	PRR5	P85299	proline rich 5
Malignant neoplasm of breast	ARHGAP8	P85298	Rho GTPase activating protein 8
Malignant neoplasm of breast	FOXK1	P85037	forkhead box K1
Malignant neoplasm of breast	SRSF3	P84103	serine and arginine rich splicing factor 3
Malignant neoplasm of breast	ARF1	P84077	ADP ribosylation factor 1
Malignant neoplasm of breast	SMAD3	P84022	SMAD family member 3
Malignant neoplasm of breast	CBX1	P83916	chromobox 1
Malignant neoplasm of breast	RPL36A	P83881	ribosomal protein L36a
Malignant neoplasm of breast	RPL24	P83731	ribosomal protein L24
Malignant neoplasm of breast	LACTB	P83111	lactamase beta
Malignant neoplasm of breast	HTRA4	P83105	HtrA serine peptidase 4
Malignant neoplasm of breast	SARNP	P82979	SAP domain containing ribonucleoprotein
Malignant neoplasm of breast	HMGN5	P82970	high mobility group nucleosome binding domain 5
Malignant neoplasm of breast	MRPS22	P82650	mitochondrial ribosomal protein S22
Malignant neoplasm of breast	DCD	P81605	dermcidin
Malignant neoplasm of breast	FAM189B	P81408	family with sequence similarity 189 member B

Malignant neoplasm of breast	PRLH	P81277	prolactin releasing hormone
Malignant neoplasm of breast	GPSM2	P81274	G protein signaling modulator 2
Malignant neoplasm of breast	HAMP	P81172	hepcidin antimicrobial peptide
Malignant neoplasm of breast	SIM1	P81133	SIM bHLH transcription factor 1
Malignant neoplasm of breast	BASP1	P80723	brain abundant membrane attached signal protein 1
Malignant neoplasm of breast	S100A12	P80511	S100 calcium binding protein A12
Malignant neoplasm of breast	DLK1	P80370	delta like non-canonical Notch ligand 1
Malignant neoplasm of breast	HSD11B2	P80365	hydroxysteroid 11-beta dehydrogenase 2
Malignant neoplasm of breast	NUCB2	P80303	nucleobindin 2
Malignant neoplasm of breast	MT1X	P80297	metallothionein 1X
Malignant neoplasm of breast	MT1H	P80294	metallothionein 1H
Malignant neoplasm of breast	MAP3K9	P80192	mitogen-activated protein kinase kinase kinase 9
Malignant neoplasm of breast	LCN2	P80188	lipocalin 2
Malignant neoplasm of breast	GPLD1	P80108	glycosylphosphatidylinositol specific phospholipase D1
Malignant neoplasm of breast	CCL7	P80098	C-C motif chemokine ligand 7
Malignant neoplasm of breast	CCL8	P80075	C-C motif chemokine ligand 8
Malignant neoplasm of breast	HLA-DRB3	P79483	major histocompatibility complex, class II, DR beta 3
Malignant neoplasm of breast	ADARB1	P78563	adenosine deaminase RNA specific B1
Malignant neoplasm of breast	CCL20	P78556	C-C motif chemokine ligand 20

Malignant neoplasm of breast	ELF3	P78545	E74 like ETS transcription factor 3
Malignant neoplasm of breast	BTG2	P78543	BTG anti-proliferation factor 2
Malignant neoplasm of breast	ADAM17	P78536	ADAM metallopeptidase domain 17
Malignant neoplasm of breast	PRKDC	P78527	protein kinase, DNA-activated, catalytic subunit
Malignant neoplasm of breast	RELN	P78509	reelin
Malignant neoplasm of breast	JAG1	P78504	jagged canonical Notch ligand 1
Malignant neoplasm of breast	CX3CL1	P78423	C-X3-C motif chemokine ligand 1
Malignant neoplasm of breast	GSTO1	P78417	glutathione S-transferase omega 1
Malignant neoplasm of breast	IRX3	P78415	iroquois homeobox 3
Malignant neoplasm of breast	BTN3A2	P78410	butyrophilin subfamily 3 member A2
Malignant neoplasm of breast	RAE1	P78406	ribonucleic acid export 1
Malignant neoplasm of breast	CCNA1	P78396	cyclin A1
Malignant neoplasm of breast	PRAME	P78395	preferentially expressed antigen in melanoma
Malignant neoplasm of breast	SLC35A2	P78381	solute carrier family 35 member A2
Malignant neoplasm of breast	OLR1	P78380	oxidized low density lipoprotein receptor 1
Malignant neoplasm of breast	CCT2	P78371	chaperonin containing TCP1 subunit 2
Malignant neoplasm of breast	CLDN10	P78369	claudin 10
Malignant neoplasm of breast	ABCA4	P78363	ATP binding cassette subfamily A member 4
Malignant neoplasm of breast	CTAG1B	P78358	cancer/testis antigen 1B

Malignant neoplasm of breast	CTAG1A	P78358	cancer/testis antigen 1A
Malignant neoplasm of breast	GTF2I	P78347	general transcription factor IIi
Malignant neoplasm of breast	EIF4G2	P78344	eukaryotic translation initiation factor 4 gamma 2
Malignant neoplasm of breast	PITX1	P78337	paired like homeodomain 1
Malignant neoplasm of breast	ADAM8	P78325	ADAM metallopeptidase domain 8
Malignant neoplasm of breast	SIRPA	P78324	signal regulatory protein alpha
Malignant neoplasm of breast	IGBP1	P78318	immunoglobulin binding protein 1
Malignant neoplasm of breast	NOP14	P78316	NOP14 nucleolar protein
Malignant neoplasm of breast	CXADR	P78310	CXADR Ig-like cell adhesion molecule
Malignant neoplasm of breast	HBB	P68871	hemoglobin subunit beta
Malignant neoplasm of breast	CSNK2A1	P68400	casein kinase 2 alpha 1
Malignant neoplasm of breast	TUBB4B	P68371	tubulin beta 4B class IVb
Malignant neoplasm of breast	TUBA1B	P68363	tubulin alpha 1b
Malignant neoplasm of breast	ACTA1	P68133	actin alpha 1, skeletal muscle
Malignant neoplasm of breast	EEF1A1	P68104	eukaryotic translation elongation factor 1 alpha 1
Malignant neoplasm of breast	ACTC1	P68032	actin alpha cardiac muscle 1
Malignant neoplasm of breast	TPM4	P67936	tropomyosin 4
Malignant neoplasm of breast	CSNK2B	P67870	casein kinase 2 beta
Malignant neoplasm of breast	YBX1	P67809	Y-box binding protein 1

Malignant neoplasm of breast	PPP2CA	P67775	protein phosphatase 2 catalytic subunit alpha
Malignant neoplasm of breast	TNNC1	P63316	troponin C1, slow skeletal and cardiac type
Malignant neoplasm of breast	TMSB10	P63313	thymosin beta 10
Malignant neoplasm of breast	SELENOW	P63302	selenoprotein W
Malignant neoplasm of breast	UBE2I	P63279	ubiquitin conjugating enzyme E2 I
Malignant neoplasm of breast	ACTG2	P63267	actin gamma 2, smooth muscle
Malignant neoplasm of breast	ACTG1	P63261	actin gamma 1
Malignant neoplasm of breast	RACK1	P63244	receptor for activated C kinase 1
Malignant neoplasm of breast	DYNLL1	P63167	dynein light chain LC8-type 1
Malignant neoplasm of breast	SUMO1	P63165	small ubiquitin like modifier 1
Malignant neoplasm of breast	SNRPN	P63162;Q9Y675	small nuclear ribonucleoprotein polypeptide N
Malignant neoplasm of breast	PPP2R2A	P63151	protein phosphatase 2 regulatory subunit Balpha
Malignant neoplasm of breast	UBE2B	P63146	ubiquitin conjugating enzyme E2 B
Malignant neoplasm of breast	YWHAZ	P63104	tyrosine 3-monooxygenase/tryptophan 5-monooxygenase activation protein zeta
Malignant neoplasm of breast	PPP3R1	P63098	protein phosphatase 3 regulatory subunit B, alpha
Malignant neoplasm of breast	AP2B1	P63010	adaptor related protein complex 2 subunit beta 1
Malignant neoplasm of breast	RAC1	P63000	Rac family small GTPase 1
Malignant neoplasm of breast	TRA2B	P62995	transformer 2 beta homolog
Malignant neoplasm of breast	GRB2	P62993	growth factor receptor bound protein 2

Malignant neoplasm of breast	RPS27A	P62979	ribosomal protein S27a
Malignant neoplasm of breast	BLCAP	P62952	BLCAP apoptosis inducing factor
Malignant neoplasm of breast	FKBP1A	P62942	FKBP prolyl isomerase 1A
Malignant neoplasm of breast	PPIA	P62937	peptidylprolyl isomerase A
Malignant neoplasm of breast	RPL31	P62899	ribosomal protein L31
Malignant neoplasm of breast	RPL39	P62891	ribosomal protein L39
Malignant neoplasm of breast	GNB1	P62873	G protein subunit beta 1
Malignant neoplasm of breast	UBE2D2	P62837	ubiquitin conjugating enzyme E2 D2
Malignant neoplasm of breast	RAP1A	P62834	RAP1A, member of RAS oncogene family
Malignant neoplasm of breast	RPL23	P62829	ribosomal protein L23
Malignant neoplasm of breast	RAN	P62826	RAN, member RAS oncogene family
Malignant neoplasm of breast	RAB1A	P62820	RAB1A, member RAS oncogene family
Malignant neoplasm of breast	H4C1	P62805	H4 clustered histone 1
Malignant neoplasm of breast	H4-16	P62805	H4 histone 16
Malignant neoplasm of breast	H4C15	P62805	H4 clustered histone 15
Malignant neoplasm of breast	H4C9	P62805	H4 clustered histone 9
Malignant neoplasm of breast	H4C4	P62805	H4 clustered histone 4
Malignant neoplasm of breast	H4C6	P62805	H4 clustered histone 6
Malignant neoplasm of breast	H4C12	P62805	H4 clustered histone 12

Malignant neoplasm of breast	H4C11	P62805	H4 clustered histone 11
Malignant neoplasm of breast	H4C3	P62805	H4 clustered histone 3
Malignant neoplasm of breast	H4C8	P62805	H4 clustered histone 8
Malignant neoplasm of breast	H4C2	P62805	H4 clustered histone 2
Malignant neoplasm of breast	H4C5	P62805	H4 clustered histone 5
Malignant neoplasm of breast	H4C13	P62805	H4 clustered histone 13
Malignant neoplasm of breast	H4C14	P62805	H4 clustered histone 14
Malignant neoplasm of breast	RPS6	P62753	ribosomal protein S6
Malignant neoplasm of breast	RPL23A	P62750	ribosomal protein L23a
Malignant neoplasm of breast	RHOB	P62745	ras homolog family member B
Malignant neoplasm of breast	ACTA2	P62736	actin alpha 2, smooth muscle
Malignant neoplasm of breast	RPS4X	P62701	ribosomal protein S4 X-linked
Malignant neoplasm of breast	CNBP	P62633	CCHC-type zinc finger nucleic acid binding protein
Malignant neoplasm of breast	ESRRG	P62508	estrogen related receptor gamma
Malignant neoplasm of breast	ETF1	P62495	eukaryotic translation termination factor 1
Malignant neoplasm of breast	RAB11A	P62491	RAB11A, member RAS oncogene family
Malignant neoplasm of breast	RPL7A	P62424	ribosomal protein L7a
Malignant neoplasm of breast	TBPL1	P62380	TATA-box binding protein like 1
Malignant neoplasm of breast	ARF6	P62330	ADP ribosylation factor 6

Malignant neoplasm of breast	BTG1	P62324	BTG anti-proliferation factor 1
Malignant neoplasm of breast	SNRPD1	P62314	small nuclear ribonucleoprotein D1 polypeptide
Malignant neoplasm of breast	RPS14	P62263	ribosomal protein S14
Malignant neoplasm of breast	YWHAE	P62258	tyrosine 3-monooxygenase/tryptophan 5-monooxygenase activation protein epsilon
Malignant neoplasm of breast	RPS16	P62249	ribosomal protein S16
Malignant neoplasm of breast	RPS15A	P62244	ribosomal protein S15a
Malignant neoplasm of breast	RPS8	P62241	ribosomal protein S8
Malignant neoplasm of breast	NCS1	P62166	neuronal calcium sensor 1
Malignant neoplasm of breast	PPP1CA	P62136	protein phosphatase 1 catalytic subunit alpha
Malignant neoplasm of breast	RPS7	P62081	ribosomal protein S7
Malignant neoplasm of breast	RRAS2	P62070	RAS related 2
Malignant neoplasm of breast	HNRNPK	P61978	heterogeneous nuclear ribonucleoprotein K
Malignant neoplasm of breast	LMO4	P61968	LIM domain only 4
Malignant neoplasm of breast	WDR5	P61964	WD repeat domain 5
Malignant neoplasm of breast	UFM1	P61960	ubiquitin fold modifier 1
Malignant neoplasm of breast	NPC2	P61916	NPC intracellular cholesterol transporter 2
Malignant neoplasm of breast	TGFB2	P61812	transforming growth factor beta 2
Malignant neoplasm of breast	B2M	P61769	beta-2-microglobulin
Malignant neoplasm of breast	LYZ	P61626	lysozyme

Malignant neoplasm of breast	HSPE1	P61604	heat shock protein family E (Hsp10) member 1
Malignant neoplasm of breast	RHOA	P61586	ras homolog family member A
Malignant neoplasm of breast	ISL1	P61371	ISL LIM homeobox 1
Malignant neoplasm of breast	PSME3	P61289	proteasome activator subunit 3
Malignant neoplasm of breast	SST	P61278	somatostatin
Malignant neoplasm of breast	MAX	P61244	MYC associated factor X
Malignant neoplasm of breast	RAP2B	P61225	RAP2B, member of RAS oncogene family
Malignant neoplasm of breast	RAP1B	P61224	RAP1B, member of RAS oncogene family
Malignant neoplasm of breast	ABCE1	P61221	ATP binding cassette subfamily E member 1
Malignant neoplasm of breast	POLR2F	P61218	RNA polymerase II subunit F
Malignant neoplasm of breast	ARF3	P61204	ADP ribosylation factor 3
Malignant neoplasm of breast	COPS2	P61201	COP9 signalosome subunit 2
Malignant neoplasm of breast	ACTR2	P61160	actin related protein 2
Malignant neoplasm of breast	UBE2N	P61088	ubiquitin conjugating enzyme E2 N
Malignant neoplasm of breast	UBE2K	P61086	ubiquitin conjugating enzyme E2 K
Malignant neoplasm of breast	UBE2D3	P61077	ubiquitin conjugating enzyme E2 D3
Malignant neoplasm of breast	CXCR4	P61073	C-X-C motif chemokine receptor 4
Malignant neoplasm of breast	CKS1B	P61024	CDC28 protein kinase regulatory subunit 1B
Malignant neoplasm of breast	RAB5B	P61020	RAB5B, member RAS oncogene family

Malignant neoplasm of breast	RAB2A	P61019	RAB2A, member RAS oncogene family
Malignant neoplasm of breast	CDC42	P60953	cell division cycle 42
Malignant neoplasm of breast	S100A10	P60903	S100 calcium binding protein A10
Malignant neoplasm of breast	SEM1	P60896	SEM1 26S proteasome complex subunit
Malignant neoplasm of breast	PRPS1	P60891	phosphoribosyl pyrophosphate synthetase 1
Malignant neoplasm of breast	EIF4A1	P60842	eukaryotic translation initiation factor 4A1
Malignant neoplasm of breast	RAC3	P60763	Rac family small GTPase 3
Malignant neoplasm of breast	ACTB	P60709	actin beta
Malignant neoplasm of breast	IL2	P60568	interleukin 2
Malignant neoplasm of breast	PPP4C	P60510	protein phosphatase 4 catalytic subunit
Malignant neoplasm of breast	PTEN	P60484	phosphatase and tensin homolog
Malignant neoplasm of breast	KRTAP10-8	P60410	keratin associated protein 10-8
Malignant neoplasm of breast	EIF3E	P60228	eukaryotic translation initiation factor 3 subunit E
Malignant neoplasm of breast	TPI1	P60174	triosephosphate isomerase 1
Malignant neoplasm of breast	CD81	P60033	CD81 molecule
Malignant neoplasm of breast	ELOF1	P60002	elongation factor 1 homolog
Malignant neoplasm of breast	GPX6	P59796	glutathione peroxidase 6
Malignant neoplasm of breast	DEFA1	P59665	defensin alpha 1
Malignant neoplasm of breast	DEFA1B	P59665	defensin alpha 1B

Malignant neoplasm of breast	TAS2R38	P59533	taste 2 receptor member 38
Malignant neoplasm of breast	TPPP2	P59282	tubulin polymerization promoting protein family member 2
Malignant neoplasm of breast	LINC00310	P59036	long intergenic non-protein coding RNA 310
Malignant neoplasm of breast	RTP1	P59025	receptor transporter protein 1
Malignant neoplasm of breast	H2BC4	P58876;P62807	H2B clustered histone 4
Malignant neoplasm of breast	H2BC5	P58876;P62807	H2B clustered histone 5
Malignant neoplasm of breast	H2BC8	P58876;P62807	H2B clustered histone 8
Malignant neoplasm of breast	H2BC7	P58876;P62807	H2B clustered histone 7
Malignant neoplasm of breast	H2BC6	P58876;P62807	H2B clustered histone 6
Malignant neoplasm of breast	H2BC10	P58876;P62807	H2B clustered histone 10
Malignant neoplasm of breast	MTPN	P58546	myotrophin
Malignant neoplasm of breast	ADAMTS12	P58397	ADAM metallopeptidase with thrombospondin type 1 motif 12
Malignant neoplasm of breast	ANTXR2	P58335	ANTXR cell adhesion molecule 2
Malignant neoplasm of breast	ZNF121	P58317	zinc finger protein 121
Malignant neoplasm of breast	LOXL3	P58215	lysyl oxidase like 3
Malignant neoplasm of breast	INHBE	P58166	inhibin subunit beta E
Malignant neoplasm of breast	SESN2	P58004	sestrin 2
Malignant neoplasm of breast	FBXW4	P57775	F-box and WD repeat domain containing 4
Malignant neoplasm of breast	RGS8	P57771	regulator of G protein signaling 8

Malignant neoplasm of breast	CLDN2	P57739	claudin 2
Malignant neoplasm of breast	RAB25	P57735	RAB25, member RAS oncogene family
Malignant neoplasm of breast	TMPRSS3	P57727	transmembrane serine protease 3
Malignant neoplasm of breast	PCBP4	P57723	poly(rC) binding protein 4
Malignant neoplasm of breast	GEMIN4	P57678	gem nuclear organelle associated protein 4
Malignant neoplasm of breast	SYNJ2BP	P57105	synaptojanin 2 binding protein
Malignant neoplasm of breast	SLC8A3	P57103	solute carrier family 8 member A3
Malignant neoplasm of breast	MAP3K7CL	P57077	MAP3K7 C-terminal like
Malignant neoplasm of breast	SOX8	P57073	SRY-box transcription factor 8
Malignant neoplasm of breast	HUNK	P57058	hormonally up-regulated Neu-associated kinase
Malignant neoplasm of breast	SLC37A1	P57057	solute carrier family 37 member 1
Malignant neoplasm of breast	NRG3	P56975	neuregulin 3
Malignant neoplasm of breast	BCAR1	P56945	BCAR1 scaffold protein, Cas family member
Malignant neoplasm of breast	HSD17B7	P56937	hydroxysteroid 17-beta dehydrogenase 7
Malignant neoplasm of breast	CLDN18	P56856	claudin 18
Malignant neoplasm of breast	CLDN12	P56749	claudin 12
Malignant neoplasm of breast	CLDN6	P56747	claudin 6
Malignant neoplasm of breast	WNT7B	P56706	Wnt family member 7B
Malignant neoplasm of breast	WNT4	P56705	Wnt family member 4

Malignant neoplasm of breast	WNT3A	P56704	Wnt family member 3A
Malignant neoplasm of breast	WNT3	P56703	Wnt family member 3
Malignant neoplasm of breast	SOX10	P56693	SRY-box transcription factor 10
Malignant neoplasm of breast	PER3	P56645	period circadian regulator 3
Malignant neoplasm of breast	CTBP2	P56545	C-terminal binding protein 2
Malignant neoplasm of breast	EIF6	P56537	eukaryotic translation initiation factor 6
Malignant neoplasm of breast	HDAC4	P56524	histone deacetylase 4
Malignant neoplasm of breast	LGALS4	P56470	galectin 4
Malignant neoplasm of breast	AP1S2	P56377	adaptor related protein complex 1 subunit sigma 2
Malignant neoplasm of breast	TCL1A	P56279	T cell leukemia/lymphoma 1A
Malignant neoplasm of breast	MAZ	P56270	MYC associated zinc finger protein
Malignant neoplasm of breast	ITGA1	P56199	integrin subunit alpha 1
Malignant neoplasm of breast	TPTE	P56180	transmembrane phosphatase with tensin homology
Malignant neoplasm of breast	DLX5	P56178	distal-less homeobox 5
Malignant neoplasm of breast	GFRA1	P56159	GDNF family receptor alpha 1
Malignant neoplasm of breast	BID	P55957	BH3 interacting domain death agonist
Malignant neoplasm of breast	RAG2	P55895	recombination activating 2
Malignant neoplasm of breast	UCP2	P55851	uncoupling protein 2
Malignant neoplasm of breast	NPEPPS	P55786	aminopeptidase puromycin sensitive

Malignant neoplasm of breast	CCL18	P55774	C-C motif chemokine ligand 18
Malignant neoplasm of breast	PAX9	P55771	paired box 9
Malignant neoplasm of breast	PRMT2	P55345	protein arginine methyltransferase 2
Malignant neoplasm of breast	TPD52	P55327	tumor protein D52
Malignant neoplasm of breast	FOXA1	P55317	forkhead box A1
Malignant neoplasm of breast	FOXG1	P55316	forkhead box G1
Malignant neoplasm of breast	CDH15	P55291	cadherin 15
Malignant neoplasm of breast	CDH13	P55290	cadherin 13
Malignant neoplasm of breast	CDH11	P55287	cadherin 11
Malignant neoplasm of breast	CDH4	P55283	cadherin 4
Malignant neoplasm of breast	CDKN2D	P55273	cyclin dependent kinase inhibitor 2D
Malignant neoplasm of breast	ADAR	P55265	adenosine deaminase RNA specific
Malignant neoplasm of breast	CASP6	P55212	caspase 6
Malignant neoplasm of breast	CASP9	P55211	caspase 9
Malignant neoplasm of breast	CASP7	P55210	caspase 7
Malignant neoplasm of breast	NAP1L1	P55209	nucleosome assembly protein 1 like 1
Malignant neoplasm of breast	ELL	P55199	elongation factor for RNA polymerase II
Malignant neoplasm of breast	AFDN	P55196	afadin, adherens junction formation factor
Malignant neoplasm of breast	MTTP	P55157	microsomal triglyceride transfer protein

Malignant neoplasm of breast	GDF10	P55107	growth differentiation factor 10
Malignant neoplasm of breast	AQP4	P55087	aquaporin 4
Malignant neoplasm of breast	F2RL1	P55085	F2R like trypsin receptor 1
Malignant neoplasm of breast	HADHB	P55084	hydroxyacyl-CoA dehydrogenase trifunctional multienzyme complex subunit beta
Malignant neoplasm of breast	MFAP4	P55083	microfibril associated protein 4
Malignant neoplasm of breast	MFAP1	P55081	microfibril associated protein 1
Malignant neoplasm of breast	FGF8	P55075	fibroblast growth factor 8
Malignant neoplasm of breast	DIO3	P55073	iodothyronine deiodinase 3
Malignant neoplasm of breast	VCP	P55072	valosin containing protein
Malignant neoplasm of breast	AQP5	P55064	aquaporin 5
Malignant neoplasm of breast	TMBIM6	P55061	transmembrane BAX inhibitor motif containing 6
Malignant neoplasm of breast	CSE1L	P55060	chromosome segregation 1 like
Malignant neoplasm of breast	PLTP	P55058	phospholipid transfer protein
Malignant neoplasm of breast	APOC4	P55056	apolipoprotein C4
Malignant neoplasm of breast	NR1H2	P55055	nuclear receptor subfamily 1 group H member 2
Malignant neoplasm of breast	RRAD	P55042	RRAD, Ras related glycolysis inhibitor and calcium channel regulator
Malignant neoplasm of breast	GEM	P55040	GTP binding protein overexpressed in skeletal muscle
Malignant neoplasm of breast	PSMD4	P55036	proteasome 26S subunit, non-ATPase 4
Malignant neoplasm of breast	SLC12A3	P55017	solute carrier family 12 member 3

Malignant neoplasm of breast	EIF5	P55010	eukaryotic translation initiation factor 5
Malignant neoplasm of breast	AIF1	P55008	allograft inflammatory factor 1
Malignant neoplasm of breast	SLURP1	P55000	secreted LY6/PLAUR domain containing 1
Malignant neoplasm of breast	HMGCS2	P54868	3-hydroxy-3-methylglutaryl-CoA synthase 2
Malignant neoplasm of breast	UGT2B15	P54855	UDP glucuronosyltransferase family 2 member B15
Malignant neoplasm of breast	EMP3	P54852	epithelial membrane protein 3
Malignant neoplasm of breast	EMP2	P54851	epithelial membrane protein 2
Malignant neoplasm of breast	EMP1	P54849	epithelial membrane protein 1
Malignant neoplasm of breast	NRL	P54845	neural retina leucine zipper
Malignant neoplasm of breast	PTPN5	P54829	protein tyrosine phosphatase non-receptor type 5
Malignant neoplasm of breast	GAS1	P54826	growth arrest specific 1
Malignant neoplasm of breast	PRRX1	P54821	paired related homeobox 1
Malignant neoplasm of breast	ARSF	P54793	arylsulfatase F
Malignant neoplasm of breast	DVL1P1	P54792	dishevelled segment polarity protein 1 pseudogene 1
Malignant neoplasm of breast	EPHA4	P54764	EPH receptor A4
Malignant neoplasm of breast	EPHB1	P54762	EPH receptor B1
Malignant neoplasm of breast	EPHB4	P54760	EPH receptor B4
Malignant neoplasm of breast	EPHA5	P54756	EPH receptor A5
Malignant neoplasm of breast	EPHB3	P54753	EPH receptor B3

Malignant neoplasm of breast	RAD23B	P54727	RAD23 homolog B, nucleotide excision repair protein
Malignant neoplasm of breast	BCAT1	P54687	branched chain amino acid transaminase 1
Malignant neoplasm of breast	HSPA2	P54652	heat shock protein family A (Hsp70) member 2
Malignant neoplasm of breast	PRKAA2	P54646	protein kinase AMP-activated catalytic subunit alpha 2
Malignant neoplasm of breast	USP14	P54578	ubiquitin specific peptidase 14
Malignant neoplasm of breast	YARS1	P54577	tyrosyl-tRNA synthetase 1
Malignant neoplasm of breast	OAZ1	P54368	ornithine decarboxylase antizyme 1
Malignant neoplasm of breast	PNLIPRP1	P54315	pancreatic lipase related protein 1
Malignant neoplasm of breast	MYOM2	P54296	myomesin 2
Malignant neoplasm of breast	PMS2	P54278	PMS1 homolog 2, mismatch repair system component
Malignant neoplasm of breast	TERF1	P54274	telomeric repeat binding factor 1
Malignant neoplasm of breast	ATN1	P54259	atrophin 1
Malignant neoplasm of breast	HAP1	P54257	huntingtin associated protein 1
Malignant neoplasm of breast	ATXN1	P54253	ataxin 1
Malignant neoplasm of breast	ATXN3	P54252	ataxin 3
Malignant neoplasm of breast	SLC18A1	P54219	solute carrier family 18 member A1
Malignant neoplasm of breast	RARS1	P54136	arginyl-tRNA synthetase 1
Malignant neoplasm of breast	BLM	P54132	BLM RecQ like helicase
Malignant neoplasm of breast	POLG	P54098	DNA polymerase gamma, catalytic subunit

Malignant neoplasm of breast	SUB1	P53999	SUB1 regulator of transcription
Malignant neoplasm of breast	IST1	P53990	IST1 factor associated with ESCRT-III
Malignant neoplasm of breast	SLC16A1	P53985	solute carrier family 16 member 1
Malignant neoplasm of breast	PLAAT3	P53816	phospholipase A and acyltransferase 3
Malignant neoplasm of breast	TTC3	P53804	tetratricopeptide repeat domain 3
Malignant neoplasm of breast	PTTG1IP	P53801	PTTG1 interacting protein
Malignant neoplasm of breast	MAPK10	P53779	mitogen-activated protein kinase 10
Malignant neoplasm of breast	MAPK12	P53778	mitogen-activated protein kinase 12
Malignant neoplasm of breast	LIMK2	P53671	LIM domain kinase 2
Malignant neoplasm of breast	LIMK1	P53667	LIM domain kinase 1
Malignant neoplasm of breast	MVD	P53602	mevalonate diphosphate decarboxylase
Malignant neoplasm of breast	FOSB	P53539	FosB proto-oncogene, AP-1 transcription factor subunit
Malignant neoplasm of breast	ACLY	P53396	ATP citrate lyase
Malignant neoplasm of breast	DAPK1	P53355	death associated protein kinase 1
Malignant neoplasm of breast	PLK1	P53350	polo like kinase 1
Malignant neoplasm of breast	PPP5C	P53041	protein phosphatase 5 catalytic subunit
Malignant neoplasm of breast	SLC25A1	P53007	solute carrier family 25 member 1
Malignant neoplasm of breast	LBX1	P52954	ladybird homeobox 1
Malignant neoplasm of breast	GBX2	P52951	gastrulation brain homeobox 2

Malignant neoplasm of breast	NUP98	P52948	nucleoporin 98 and 96 precursor
Malignant neoplasm of breast	PDX1	P52945	pancreatic and duodenal homeobox 1
Malignant neoplasm of breast	CRIP2	P52943	cysteine rich protein 2
Malignant neoplasm of breast	HMGA2	P52926	high mobility group AT-hook 2
Malignant neoplasm of breast	AKR1C2	P52895	aldo-keto reductase family 1 member C2
Malignant neoplasm of breast	THOP1	P52888	thimet oligopeptidase 1
Malignant neoplasm of breast	NDST1	P52848	N-deacetylase and N-sulfotransferase 1
Malignant neoplasm of breast	STC1	P52823	stanniocalcin 1
Malignant neoplasm of breast	EFNA5	P52803	ephrin A5
Malignant neoplasm of breast	EFNB2	P52799	ephrin B2
Malignant neoplasm of breast	EFNA3	P52797	ephrin A3
Malignant neoplasm of breast	HK3	P52790	hexokinase 3
Malignant neoplasm of breast	HK2	P52789	hexokinase 2
Malignant neoplasm of breast	SMS	P52788	spermine synthase
Malignant neoplasm of breast	RBM5	P52756	RNA binding motif protein 5
Malignant neoplasm of breast	ZNF143	P52747	zinc finger protein 143
Malignant neoplasm of breast	ZNF131	P52739	zinc finger protein 131
Malignant neoplasm of breast	VAV2	P52735	vav guanine nucleotide exchange factor 2
Malignant neoplasm of breast	KIF11	P52732	kinesin family member 11

Malignant neoplasm of breast	MSH6	P52701	mutS homolog 6
Malignant neoplasm of breast	GTF2A1	P52655	general transcription factor IIA subunit 1
Malignant neoplasm of breast	STAT2	P52630	signal transducer and activator of transcription 2
Malignant neoplasm of breast	HNRNPF	P52597	heterogeneous nuclear ribonucleoprotein F
Malignant neoplasm of breast	AGFG1	P52594	ArfGAP with FG repeats 1
Malignant neoplasm of breast	SLC7A2	P52569	solute carrier family 7 member 2
Malignant neoplasm of breast	ARHGDIB	P52566	Rho GDP dissociation inhibitor beta
Malignant neoplasm of breast	ARHGDIA	P52565	Rho GDP dissociation inhibitor alpha
Malignant neoplasm of breast	DGKE	P52429	diacylglycerol kinase epsilon
Malignant neoplasm of breast	JAK3	P52333	Janus kinase 3
Malignant neoplasm of breast	RAP1GDS1	P52306	Rap1 GTPase-GDP dissociation stimulator 1
Malignant neoplasm of breast	NCBP2	P52298	nuclear cap binding protein subunit 2
Malignant neoplasm of breast	KPNA2	P52292	karyopherin subunit alpha 2
Malignant neoplasm of breast	HNRNPM	P52272	heterogeneous nuclear ribonucleoprotein M
Malignant neoplasm of breast	PGD	P52209	phosphogluconate dehydrogenase
Malignant neoplasm of breast	FUT6	P51993	fucosyltransferase 6
Malignant neoplasm of breast	NDUFA8	P51970	NADH:ubiquinone oxidoreductase subunit A8
Malignant neoplasm of breast	CCNG1	P51959	cyclin G1
Malignant neoplasm of breast	NEK3	P51956	NIMA related kinase 3

Malignant neoplasm of breast	NEK2	P51955	NIMA related kinase 2
Malignant neoplasm of breast	MNAT1	P51948	MNAT1 component of CDK activating kinase
Malignant neoplasm of breast	CCNH	P51946	cyclin H
Malignant neoplasm of breast	LUM	P51884	lumican
Malignant neoplasm of breast	CASP5	P51878	caspase 5
Malignant neoplasm of breast	CDR1	P51861	cerebellar degeneration related protein 1
Malignant neoplasm of breast	HDGF	P51858	heparin binding growth factor
Malignant neoplasm of breast	TKTL1	P51854	transketolase like 1
Malignant neoplasm of breast	NR0B1	P51843	nuclear receptor subfamily 0 group B member 1
Malignant neoplasm of breast	GUCY2F	P51841	guanylate cyclase 2F, retinal
Malignant neoplasm of breast	AFF3	P51826	AF4/FMR2 family member 3
Malignant neoplasm of breast	AFF1	P51825	AF4/FMR2 family member 1
Malignant neoplasm of breast	RPS6KA3	P51812	ribosomal protein S6 kinase A3
Malignant neoplasm of breast	XK	P51811	X-linked Kx blood group
Malignant neoplasm of breast	VAMP7	P51809	vesicle associated membrane protein 7
Malignant neoplasm of breast	PLXNA3	P51805	plexin A3
Malignant neoplasm of breast	CLCN3	P51790	chloride voltage-gated channel 3
Malignant neoplasm of breast	USP11	P51784	ubiquitin specific peptidase 11
Malignant neoplasm of breast	STAT5B	P51692	signal transducer and activator of transcription 5B

Malignant neoplasm of breast	ARSD	P51689	arylsulfatase D
Malignant neoplasm of breast	CCR9	P51686	C-C motif chemokine receptor 9
Malignant neoplasm of breast	CCR6	P51684	C-C motif chemokine receptor 6
Malignant neoplasm of breast	CCR5	P51681	C-C motif chemokine receptor 5 (gene/pseudogene)
Malignant neoplasm of breast	CCR4	P51679	C-C motif chemokine receptor 4
Malignant neoplasm of breast	CCR3	P51677	C-C motif chemokine receptor 3
Malignant neoplasm of breast	CCL11	P51671	C-C motif chemokine ligand 11
Malignant neoplasm of breast	PSMD7	P51665	proteasome 26S subunit, non-ATPase 7
Malignant neoplasm of breast	HSD17B4	P51659	hydroxysteroid 17-beta dehydrogenase 4
Malignant neoplasm of breast	GPC3	P51654	glypican 3
Malignant neoplasm of breast	CAV2	P51636	caveolin 2
Malignant neoplasm of breast	IRAK1	P51617	interleukin 1 receptor associated kinase 1
Malignant neoplasm of breast	MECP2	P51608	methyl-CpG binding protein 2
Malignant neoplasm of breast	RENBP	P51606	renin binding protein
Malignant neoplasm of breast	CYP2J2	P51589	cytochrome P450 family 2 subfamily J member 2
Malignant neoplasm of breast	BRCA2	P51587	BRCA2 DNA repair associated
Malignant neoplasm of breast	BCAP31	P51572	B cell receptor associated protein 31
Malignant neoplasm of breast	SMARCA4	P51532	SWI/SNF related, matrix associated, actin dependent regulator of chromatin, subfamily a, member 4
Malignant neoplasm of breast	DNA2	P51530	DNA replication helicase/nuclease 2

Malignant neoplasm of breast	NOVA1	P51513	NOVA alternative splicing regulator 1
Malignant neoplasm of breast	RORC	P51449	RAR related orphan receptor C
Malignant neoplasm of breast	DAP3	P51398	death associated protein 3
Malignant neoplasm of breast	DAP	P51397	death associated protein
Malignant neoplasm of breast	PLCD1	P51178	phospholipase C delta 1
Malignant neoplasm of breast	SCNN1B	P51168	sodium channel epithelial 1 subunit beta
Malignant neoplasm of breast	RAB27A	P51159	RAB27A, member RAS oncogene family
Malignant neoplasm of breast	RAB9A	P51151	RAB9A, member RAS oncogene family
Malignant neoplasm of breast	GZMM	P51124	granzyme M
Malignant neoplasm of breast	FXR1	P51114	FMR1 autosomal homolog 1
Malignant neoplasm of breast	CCT4	P50991	chaperonin containing TCP1 subunit 4
Malignant neoplasm of breast	PPT1	P50897	palmitoyl-protein thioesterase 1
Malignant neoplasm of breast	BCAM	P50895	basal cell adhesion molecule (Lutheran blood group)
Malignant neoplasm of breast	RNF144A	P50876	ring finger protein 144A
Malignant neoplasm of breast	LRBA	P50851	LPS responsive beige-like anchor protein
Malignant neoplasm of breast	CDK9	P50750	cyclin dependent kinase 9
Malignant neoplasm of breast	RASSF2	P50749	Ras association domain family member 2
Malignant neoplasm of breast	HLCS	P50747	holocarboxylase synthetase
Malignant neoplasm of breast	TOB1	P50616	transducer of ERBB2, 1

Malignant neoplasm of breast	CDK7	P50613	cyclin dependent kinase 7
Malignant neoplasm of breast	TUB	P50607	TUB bipartite transcription factor
Malignant neoplasm of breast	TNFSF10	P50591	TNF superfamily member 10
Malignant neoplasm of breast	NUDT2	P50583	nudix hydrolase 2
Malignant neoplasm of breast	DNM2	P50570	dynamin 2
Malignant neoplasm of breast	ASCL1	P50553	achaete-scute family bHLH transcription factor 1
Malignant neoplasm of breast	VASP	P50552	vasodilator stimulated phosphoprotein
Malignant neoplasm of breast	ETV1	P50549	ETS variant transcription factor 1
Malignant neoplasm of breast	MXI1	P50539	MAX interactor 1, dimerization protein
Malignant neoplasm of breast	ST13	P50502	ST13 Hsp70 interacting protein
Malignant neoplasm of breast	PDLIM4	P50479	PDZ and LIM domain 4
Malignant neoplasm of breast	CSRP3	P50461	cysteine and glycine rich protein 3
Malignant neoplasm of breast	LHX2	P50458	LIM homeobox 2
Malignant neoplasm of breast	SERPINH1	P50454	serpin family H member 1
Malignant neoplasm of breast	SERPINB9	P50453	serpin family B member 9
Malignant neoplasm of breast	SLC26A2	P50443	solute carrier family 26 member 2
Malignant neoplasm of breast	CPT1A	P50416	carnitine palmitoyltransferase 1A
Malignant neoplasm of breast	EMD	P50402	emerin
Malignant neoplasm of breast	MMP14	P50281	matrix metallopeptidase 14

Malignant neoplasm of breast	CRIP1	P50238	cysteine rich protein 1
Malignant neoplasm of breast	SULT1A2	P50226	sulfotransferase family 1A member 2
Malignant neoplasm of breast	SULT1A1	P50225	sulfotransferase family 1A member 1
Malignant neoplasm of breast	MEOX1	P50221	mesenchyme homeobox 1
Malignant neoplasm of breast	MNX1	P50219	motor neuron and pancreas homeobox 1
Malignant neoplasm of breast	GNAQ	P50148	G protein subunit alpha q
Malignant neoplasm of breast	HNMT	P50135	histamine N-methyltransferase
Malignant neoplasm of breast	RBP2	P50120	retinol binding protein 2
Malignant neoplasm of breast	AGTR2	P50052	angiotensin II receptor type 2
Malignant neoplasm of breast	MRE11	P49959	MRE11 homolog, double strand break repair nuclease
Malignant neoplasm of breast	CDKN1C	P49918	cyclin dependent kinase inhibitor 1C
Malignant neoplasm of breast	LIG4	P49917	DNA ligase 4
Malignant neoplasm of breast	LIG3	P49916	DNA ligase 3
Malignant neoplasm of breast	CAMP	P49913	cathelicidin antimicrobial peptide
Malignant neoplasm of breast	SELENOP	P49908	selenoprotein P
Malignant neoplasm of breast	NT5C2	P49902	5'-nucleotidase, cytosolic II
Malignant neoplasm of breast	SULT1E1	P49888	sulfotransferase family 1E member 1
Malignant neoplasm of breast	KLK7	P49862	kallikrein related peptidase 7
Malignant neoplasm of breast	GSK3B	P49841	glycogen synthase kinase 3 beta

Malignant neoplasm of breast	TSC2	P49815	TSC complex subunit 2
Malignant neoplasm of breast	PSEN2	P49810	presenilin 2
Malignant neoplasm of breast	RGS4	P49798	regulator of G protein signaling 4
Malignant neoplasm of breast	RGS3	P49796	regulator of G protein signaling 3
Malignant neoplasm of breast	FHIT	P49789	fragile histidine triad diadenosine triphosphatase
Malignant neoplasm of breast	RARRES1	P49788	retinoic acid receptor responder 1
Malignant neoplasm of breast	FLT3LG	P49771	fms related receptor tyrosine kinase 3 ligand
Malignant neoplasm of breast	EIF2B2	P49770	eukaryotic translation initiation factor 2B subunit beta
Malignant neoplasm of breast	PSEN1	P49768	presenilin 1
Malignant neoplasm of breast	VEGFC	P49767	vascular endothelial growth factor C
Malignant neoplasm of breast	VEGFB	P49765	vascular endothelial growth factor B
Malignant neoplasm of breast	PGF	P49763	placental growth factor
Malignant neoplasm of breast	CLK2	P49760	CDC like kinase 2
Malignant neoplasm of breast	RGS6	P49758	regulator of G protein signaling 6
Malignant neoplasm of breast	NUMB	P49757	NUMB endocytic adaptor protein
Malignant neoplasm of breast	TMED10	P49755	transmembrane p24 trafficking protein 10
Malignant neoplasm of breast	COMP	P49747	cartilage oligomeric matrix protein
Malignant neoplasm of breast	THBS3	P49746	thrombospondin 3
Malignant neoplasm of breast	MCM2	P49736	minichromosome maintenance complex component 2

Malignant neoplasm of breast	CEBPD	P49716	CCAAT enhancer binding protein delta
Malignant neoplasm of breast	CEBPA	P49715	CCAAT enhancer binding protein alpha
Malignant neoplasm of breast	CTCF	P49711	CCCTC-binding factor
Malignant neoplasm of breast	CXCR3	P49682	C-X-C motif chemokine receptor 3
Malignant neoplasm of breast	STAR	P49675	steroidogenic acute regulatory protein
Malignant neoplasm of breast	CSNK1E	P49674	casein kinase 1 epsilon
Malignant neoplasm of breast	TPTEP2-CSNK1E	P49674	TPTEP2-CSNK1E readthrough
Malignant neoplasm of breast	PRIM2	P49643	DNA primase subunit 2
Malignant neoplasm of breast	PRIM1	P49642	DNA primase subunit 1
Malignant neoplasm of breast	HOXA1	P49639	homeobox A1
Malignant neoplasm of breast	DGKG	P49619	diacylglycerol kinase gamma
Malignant neoplasm of breast	PPM1F	P49593	protein phosphatase, Mg2+/Mn2+ dependent 1F
Malignant neoplasm of breast	SARS1	P49591	seryl-tRNA synthetase 1
Malignant neoplasm of breast	AARS1	P49588	alanyl-tRNA synthetase 1
Malignant neoplasm of breast	CENPF	P49454	centromere protein F
Malignant neoplasm of breast	CENPA	P49450	centromere protein A
Malignant neoplasm of breast	ALDH7A1	P49419	aldehyde dehydrogenase 7 family member A1
Malignant neoplasm of breast	AMPH	P49418	amphiphysin
Malignant neoplasm of breast	ARRB1	P49407	arrestin beta 1

Malignant neoplasm of breast	MRPL19	P49406	mitochondrial ribosomal protein L19
Malignant neoplasm of breast	CCT3	P49368	chaperonin containing TCP1 subunit 3
Malignant neoplasm of breast	DHPS	P49366	deoxyhypusine synthase
Malignant neoplasm of breast	CDK8	P49336	cyclin dependent kinase 8
Malignant neoplasm of breast	FASN	P49327	fatty acid synthase
Malignant neoplasm of breast	FMO5	P49326	flavin containing dimethylaniline monoxygenase 5
Malignant neoplasm of breast	MTNR1B	P49286	melatonin receptor 1B
Malignant neoplasm of breast	SLC11A2	P49281	solute carrier family 11 member 2
Malignant neoplasm of breast	SLC11A1	P49279	solute carrier family 11 member 1
Malignant neoplasm of breast	CX3CR1	P49238	C-X3-C motif chemokine receptor 1
Malignant neoplasm of breast	NR2C2	P49116	nuclear receptor subfamily 2 group C member 2
Malignant neoplasm of breast	CAMLG	P49069	calcium modulating ligand
Malignant neoplasm of breast	PXN	P49023	paxillin
Malignant neoplasm of breast	HCAR3	P49019	hydroxycarboxylic acid receptor 3
Malignant neoplasm of breast	MARCKSL1	P49006	MARCKS like 1
Malignant neoplasm of breast	TRPC1	P48995	transient receptor potential cation channel subfamily C member 1
Malignant neoplasm of breast	ADGRE5	P48960	adhesion G protein-coupled receptor E5
Malignant neoplasm of breast	TDO2	P48775	tryptophan 2,3-dioxygenase
Malignant neoplasm of breast	CCN3	P48745	cellular communication network factor 3

Malignant neoplasm of breast	PIK3CG	P48736	phosphatidylinositol-4,5-bisphosphate 3-kinase catalytic subunit gamma
Malignant neoplasm of breast	IDH2	P48735	isocitrate dehydrogenase (NADP(+)) 2
Malignant neoplasm of breast	CSNK1D	P48730	casein kinase 1 delta
Malignant neoplasm of breast	NES	P48681	nestin
Malignant neoplasm of breast	NMU	P48645	neuromedin U
Malignant neoplasm of breast	CCT5	P48643	chaperonin containing TCP1 subunit 5
Malignant neoplasm of breast	PRRC2A	P48634	proline rich coiled-coil 2A
Malignant neoplasm of breast	PSMD8	P48556	proteasome 26S subunit, non-ATPase 8
Malignant neoplasm of breast	NRIP1	P48552	nuclear receptor interacting protein 1
Malignant neoplasm of breast	KCNJ3	P48549	potassium inwardly rectifying channel subfamily J member 3
Malignant neoplasm of breast	PCP4	P48539	Purkinje cell protein 4
Malignant neoplasm of breast	CD151	P48509	CD151 molecule (Raph blood group)
Malignant neoplasm of breast	GCLC	P48506	glutamate-cysteine ligase catalytic subunit
Malignant neoplasm of breast	LSS	P48449	lanosterol synthase
Malignant neoplasm of breast	RXRG	P48443	retinoid X receptor gamma
Malignant neoplasm of breast	SOX9	P48436	SRY-box transcription factor 9
Malignant neoplasm of breast	SOX2	P48431	SRY-box transcription factor 2
Malignant neoplasm of breast	RFX2	P48378	regulatory factor X2
Malignant neoplasm of breast	LEPR	P48357	leptin receptor

Malignant neoplasm of breast	TFPI2	P48307	tissue factor pathway inhibitor 2
Malignant neoplasm of breast	GABRA4	P48169	gamma-aminobutyric acid type A receptor subunit alpha4
Malignant neoplasm of breast	GJA8	P48165	gap junction protein alpha 8
Malignant neoplasm of breast	PREP	P48147	prolyl endopeptidase
Malignant neoplasm of breast	NPBWR1	P48145	neuropeptides B and W receptor 1
Malignant neoplasm of breast	CXCL12	P48061	C-X-C motif chemokine ligand 12
Malignant neoplasm of breast	LIMS1	P48059	LIM zinc finger domain containing 1
Malignant neoplasm of breast	KCNJ1	P48048	potassium inwardly rectifying channel subfamily J member 1
Malignant neoplasm of breast	MTNR1A	P48039	melatonin receptor 1A
Malignant neoplasm of breast	SLC6A8	P48029	solute carrier family 6 member 8
Malignant neoplasm of breast	FASLG	P48023	Fas ligand
Malignant neoplasm of breast	XCL1	P47992	X-C motif chemokine ligand 1
Malignant neoplasm of breast	XDH	P47989	xanthine dehydrogenase
Malignant neoplasm of breast	UQCRFS1	P47985	ubiquinol-cytochrome c reductase, Rieske iron-sulfur polypeptide 1
Malignant neoplasm of breast	ZFP36L2	P47974	ZFP36 ring finger protein like 2
Malignant neoplasm of breast	LGALS7	P47929	galectin 7
Malignant neoplasm of breast	LGALS7B	P47929	galectin 7B
Malignant neoplasm of breast	ID4	P47928	inhibitor of DNA binding 4, HLH protein
Malignant neoplasm of breast	RPL29	P47914	ribosomal protein L29

Malignant neoplasm of breast	ALDH1A3	P47895	aldehyde dehydrogenase 1 family member A3
Malignant neoplasm of breast	RAP1GAP	P47736	RAP1 GTPase activating protein
Malignant neoplasm of breast	PLA2G4A	P47712	phospholipase A2 group IVA
Malignant neoplasm of breast	CSN1S1	P47710	casein alpha s1
Malignant neoplasm of breast	IQGAP1	P46940	IQ motif containing GTPase activating protein 1
Malignant neoplasm of breast	UTRN	P46939	utrophin
Malignant neoplasm of breast	YAP1	P46937	Yes associated protein 1
Malignant neoplasm of breast	NEDD4	P46934	NEDD4 E3 ubiquitin protein ligase
Malignant neoplasm of breast	RPS9	P46781	ribosomal protein S9
Malignant neoplasm of breast	RPL5	P46777	ribosomal protein L5
Malignant neoplasm of breast	BRCC3	P46736	BRCA1/BRCA2-containing complex subunit 3
Malignant neoplasm of breast	MAP2K3	P46734	mitogen-activated protein kinase kinase 3
Malignant neoplasm of breast	SLCO1A2	P46721	solute carrier organic anion transporter family member 1A2
Malignant neoplasm of breast	BDKRB1	P46663	bradykinin receptor B1
Malignant neoplasm of breast	NOTCH1	P46531	notch receptor 1
Malignant neoplasm of breast	CDKN1B	P46527	cyclin dependent kinase inhibitor 1B
Malignant neoplasm of breast	GSTM5	P46439	glutathione S-transferase mu 5
Malignant neoplasm of breast	CRKL	P46109	CRK like proto-oncogene, adaptor protein
Malignant neoplasm of breast	CRK	P46108	CRK proto-oncogene, adaptor protein

Malignant neoplasm of breast	ATRX	P46100	ATRX chromatin remodeler
Malignant neoplasm of breast	XCR1	P46094	X-C motif chemokine receptor 1
Malignant neoplasm of breast	CCR10	P46092	C-C motif chemokine receptor 10
Malignant neoplasm of breast	NOP2	P46087	NOP2 nucleolar protein
Malignant neoplasm of breast	RECQL	P46063	RecQ like helicase
Malignant neoplasm of breast	MKI67	P46013	marker of proliferation Ki-67
Malignant neoplasm of breast	MAP2K4	P45985	mitogen-activated protein kinase kinase 4
Malignant neoplasm of breast	MAPK9	P45984	mitogen-activated protein kinase 9
Malignant neoplasm of breast	MAPK8	P45983	mitogen-activated protein kinase 8
Malignant neoplasm of breast	CBX5	P45973	chromobox 5
Malignant neoplasm of breast	ACADSB	P45954	acyl-CoA dehydrogenase short/branched chain
Malignant neoplasm of breast	ABCG1	P45844	ATP binding cassette subfamily G member 1
Malignant neoplasm of breast	MMP13	P45452	matrix metallopeptidase 13
Malignant neoplasm of breast	ASPA	P45381	aspartoacylase
Malignant neoplasm of breast	TNNT3	P45378	troponin T3, fast skeletal type
Malignant neoplasm of breast	NKX2-1	P43699	NK2 homeobox 1
Malignant neoplasm of breast	GATA4	P43694	GATA binding protein 4
Malignant neoplasm of breast	CHRNA4	P43681	cholinergic receptor nicotinic alpha 4 subunit
Malignant neoplasm of breast	LPAR6	P43657	lysophosphatidic acid receptor 6

Malignant neoplasm of breast	AFM	P43652	afamin
Malignant neoplasm of breast	KIR2DS2	P43631	killer cell immunoglobulin like receptor, two Ig domains and short cytoplasmic tail 2
Malignant neoplasm of breast	KIR3DL2	P43630	killer cell immunoglobulin like receptor, three Ig domains and long cytoplasmic tail 2
Malignant neoplasm of breast	KIR3DL1	P43629	killer cell immunoglobulin like receptor, three Ig domains and long cytoplasmic tail 1
Malignant neoplasm of breast	KIR2DL3	P43628	killer cell immunoglobulin like receptor, two Ig domains and long cytoplasmic tail 3
Malignant neoplasm of breast	NAMPT	P43490	nicotinamide phosphoribosyltransferase
Malignant neoplasm of breast	RANBP1	P43487	RAN binding protein 1
Malignant neoplasm of breast	SYK	P43405	spleen associated tyrosine kinase
Malignant neoplasm of breast	ZAP70	P43403	zeta chain of T cell receptor associated protein kinase 70
Malignant neoplasm of breast	PTPN9	P43378	protein tyrosine phosphatase non-receptor type 9
Malignant neoplasm of breast	MAGEA12	P43365	MAGE family member A12
Malignant neoplasm of breast	MAGEA5	P43359	MAGE family member A5
Malignant neoplasm of breast	MAGEA4	P43358	MAGE family member A4
Malignant neoplasm of breast	MAGEA2B	P43356	MAGE family member A2B
Malignant neoplasm of breast	MAGEA2	P43356	MAGE family member A2
Malignant neoplasm of breast	MAGEA1	P43355	MAGE family member A1
Malignant neoplasm of breast	NR4A2	P43354	nuclear receptor subfamily 4 group A member 2
Malignant neoplasm of breast	RAD52	P43351	RAD52 homolog, DNA repair protein
Malignant neoplasm of breast	SSR1	P43307	signal sequence receptor subunit 1

Malignant neoplasm of breast	ETV4	P43268	ETS variant transcription factor 4
Malignant neoplasm of breast	BTD	P43251	biotinidase
Malignant neoplasm of breast	MSH2	P43246	mutS homolog 2
Malignant neoplasm of breast	CTSK	P43235	cathepsin K
Malignant neoplasm of breast	CTSO	P43234	cathepsin O
Malignant neoplasm of breast	GLP1R	P43220	glucagon like peptide 1 receptor
Malignant neoplasm of breast	CRAT	P43155	carnitine O-acetyltransferase
Malignant neoplasm of breast	DCC	P43146	DCC netrin 1 receptor
Malignant neoplasm of breast	MCAM	P43121	melanoma cell adhesion molecule
Malignant neoplasm of breast	PTGER2	P43116	prostaglandin E receptor 2
Malignant neoplasm of breast	PTGER3	P43115	prostaglandin E receptor 3
Malignant neoplasm of breast	PTGFR	P43088	prostaglandin F receptor
Malignant neoplasm of breast	GDF5	P43026	growth differentiation factor 5
Malignant neoplasm of breast	SLC1A2	P43004	solute carrier family 1 member 2
Malignant neoplasm of breast	MTHFR	P42898	methylenetetrahydrofolate reductase
Malignant neoplasm of breast	ECE1	P42892	endothelin converting enzyme 1
Malignant neoplasm of breast	HTT	P42858	huntingtin
Malignant neoplasm of breast	CXCL5	P42830	C-X-C motif chemokine ligand 5
Malignant neoplasm of breast	PRCP	P42785	prolylcarboxypeptidase

Malignant neoplasm of breast	CDKN2C	P42773	cyclin dependent kinase inhibitor 2C
Malignant neoplasm of breast	CDKN2B	P42772	cyclin dependent kinase inhibitor 2B
Malignant neoplasm of breast	CDKN2A	P42771;Q8N726	cyclin dependent kinase inhibitor 2A
Malignant neoplasm of breast	WAS	P42768	WASP actin nucleation promoting factor
Malignant neoplasm of breast	LRPPRC	P42704	leucine rich pentatricopeptide repeat containing
Malignant neoplasm of breast	LIFR	P42702	LIF receptor subunit alpha
Malignant neoplasm of breast	FRK	P42685	fyn related Src family tyrosine kinase
Malignant neoplasm of breast	ABL2	P42684	ABL proto-oncogene 2, non-receptor tyrosine kinase
Malignant neoplasm of breast	MATK	P42679	megakaryocyte-associated tyrosine kinase
Malignant neoplasm of breast	RPS27	P42677	ribosomal protein S27
Malignant neoplasm of breast	CASP2	P42575	caspase 2
Malignant neoplasm of breast	CASP3	P42574	caspase 3
Malignant neoplasm of breast	EPS15	P42566	epidermal growth factor receptor pathway substrate 15
Malignant neoplasm of breast	MTOR	P42345	mechanistic target of rapamycin kinase
Malignant neoplasm of breast	PIK3CB	P42338	phosphatidylinositol-4,5-bisphosphate 3-kinase catalytic subunit beta
Malignant neoplasm of breast	PIK3CA	P42336	phosphatidylinositol-4,5-bisphosphate 3-kinase catalytic subunit alpha
Malignant neoplasm of breast	AKR1C3	P42330	aldo-keto reductase family 1 member C3
Malignant neoplasm of breast	STAT5A	P42229	signal transducer and activator of transcription 5A
Malignant neoplasm of breast	STAT6	P42226	signal transducer and activator of transcription 6

Malignant neoplasm of breast	STAT1	P42224	signal transducer and activator of transcription 1
Malignant neoplasm of breast	TMPO	P42166;P42167	thymopoietin
Malignant neoplasm of breast	ASIP	P42127	agouti signaling protein
Malignant neoplasm of breast	CD86	P42081	CD86 molecule
Malignant neoplasm of breast	ACTR1B	P42025	actin related protein 1B
Malignant neoplasm of breast	ELK3	P41970	ETS transcription factor ELK3
Malignant neoplasm of breast	CCR2	P41597	C-C motif chemokine receptor 2
Malignant neoplasm of breast	VIPR2	P41587	vasoactive intestinal peptide receptor 2
Malignant neoplasm of breast	ADCYAP1R1	P41586	ADCYAP receptor type I
Malignant neoplasm of breast	EIF1	P41567	eukaryotic translation initiation factor 1
Malignant neoplasm of breast	SLC19A1	P41440	solute carrier family 19 member 1
Malignant neoplasm of breast	MAP3K8	P41279	mitogen-activated protein kinase kinase kinase 8
Malignant neoplasm of breast	TNFSF9	P41273	TNF superfamily member 9
Malignant neoplasm of breast	IARS1	P41252	isoleucyl-tRNA synthetase 1
Malignant neoplasm of breast	GARS1	P41250	glycyl-tRNA synthetase 1
Malignant neoplasm of breast	CSK	P41240	C-terminal Src kinase
Malignant neoplasm of breast	PPP1R2	P41236	protein phosphatase 1 regulatory inhibitor subunit 2
Malignant neoplasm of breast	HNF4A	P41235	hepatocyte nuclear factor 4 alpha
Malignant neoplasm of breast	P2RY2	P41231	purinergic receptor P2Y2

Malignant neoplasm of breast	KDM5C	P41229	lysine demethylase 5C
Malignant neoplasm of breast	NAA10	P41227	N-alpha-acetyltransferase 10, NatA catalytic subunit
Malignant neoplasm of breast	SOX3	P41225	SRY-box transcription factor 3
Malignant neoplasm of breast	PTGDS	P41222	prostaglandin D2 synthase
Malignant neoplasm of breast	WNT5A	P41221	Wnt family member 5A
Malignant neoplasm of breast	RGS2	P41220	regulator of G protein signaling 2
Malignant neoplasm of breast	CD200	P41217	CD200 molecule
Malignant neoplasm of breast	ETV6	P41212	ETS variant transcription factor 6
Malignant neoplasm of breast	BCL6	P41182	BCL6 transcription repressor
Malignant neoplasm of breast	CASR	P41180	calcium sensing receptor
Malignant neoplasm of breast	ETV3	P41162	ETS variant transcription factor 3
Malignant neoplasm of breast	ETV5	P41161	ETS variant transcription factor 5
Malignant neoplasm of breast	LEP	P41159	leptin
Malignant neoplasm of breast	ID1	P41134	inhibitor of DNA binding 1, HLH protein
Malignant neoplasm of breast	EIF2S3	P41091	eukaryotic translation initiation factor 2 subunit gamma
Malignant neoplasm of breast	CCNF	P41002	cyclin F
Malignant neoplasm of breast	PMEL	P40967	premelanosome protein
Malignant neoplasm of breast	HADHA	P40939	hydroxyacyl-CoA dehydrogenase trifunctional multienzyme complex subunit alpha
Malignant neoplasm of breast	RFC3	P40938	replication factor C subunit 3

Malignant neoplasm of breast	IL15	P40933	interleukin 15
Malignant neoplasm of breast	SLC26A3	P40879	solute carrier family 26 member 3
Malignant neoplasm of breast	USP8	P40818	ubiquitin specific peptidase 8
Malignant neoplasm of breast	STAT3	P40763	signal transducer and activator of transcription 3
Malignant neoplasm of breast	MLH1	P40692	mutL homolog 1
Malignant neoplasm of breast	PBX1	P40424	PBX homeobox 1
Malignant neoplasm of breast	VHL	P40337	von Hippel-Lindau tumor suppressor
Malignant neoplasm of breast	CTRL	P40313	chymotrypsin like
Malignant neoplasm of breast	IFI27	P40305	interferon alpha inducible protein 27
Malignant neoplasm of breast	NNMT	P40261	nicotinamide N-methyltransferase
Malignant neoplasm of breast	CCT6A	P40227	chaperonin containing TCP1 subunit 6A
Malignant neoplasm of breast	THPO	P40225	thrombopoietin
Malignant neoplasm of breast	CEACAM6	P40199	CEA cell adhesion molecule 6
Malignant neoplasm of breast	CEACAM3	P40198	CEA cell adhesion molecule 3
Malignant neoplasm of breast	IL6ST	P40189	interleukin 6 signal transducer
Malignant neoplasm of breast	CAP2	P40123	cyclase associated actin cytoskeleton regulatory protein 2
Malignant neoplasm of breast	CAPG	P40121	capping actin protein, gelsolin like
Malignant neoplasm of breast	GDNF	P39905	glial cell derived neurotrophic factor
Malignant neoplasm of breast	MMP12	P39900	matrix metallopeptidase 12

Malignant neoplasm of breast	CUX1	P39880;Q13948	cut like homeobox 1
Malignant neoplasm of breast	FEN1	P39748	flap structure-specific endonuclease 1
Malignant neoplasm of breast	ANP32A	P39687	acidic nuclear phosphoprotein 32 family member A
Malignant neoplasm of breast	COL18A1	P39060	collagen type XVIII alpha 1 chain
Malignant neoplasm of breast	COL15A1	P39059	collagen type XV alpha 1 chain
Malignant neoplasm of breast	RPS19	P39019	ribosomal protein S19
Malignant neoplasm of breast	CDKN1A	P38936	cyclin dependent kinase inhibitor 1A
Malignant neoplasm of breast	IGHMBP2	P38935	immunoglobulin mu DNA binding protein 2
Malignant neoplasm of breast	HSPA9	P38646	heat shock protein family A (Hsp70) member 9
Malignant neoplasm of breast	SPAM1	P38567	sperm adhesion molecule 1
Malignant neoplasm of breast	IFNGR2	P38484	interferon gamma receptor 2
Malignant neoplasm of breast	COIL	P38432	coilin
Malignant neoplasm of breast	BRCA1	P38398	BRCA1 DNA repair associated
Malignant neoplasm of breast	SNCA	P37840	synuclein alpha
Malignant neoplasm of breast	TALDO1	P37837	transaldolase 1
Malignant neoplasm of breast	TAGLN2	P37802	transgelin 2
Malignant neoplasm of breast	ZEB1	P37275	zinc finger E-box binding homeobox 1
Malignant neoplasm of breast	PPARG	P37231	peroxisome proliferator activated receptor gamma
Malignant neoplasm of breast	NUP62	P37198	nucleoporin 62

Malignant neoplasm of breast	TGFBR2	P37173	transforming growth factor beta receptor 2
Malignant neoplasm of breast	SCNN1A	P37088	sodium channel epithelial 1 subunit alpha
Malignant neoplasm of breast	HSD17B2	P37059	hydroxysteroid 17-beta dehydrogenase 2
Malignant neoplasm of breast	ACVRL1	P37023	activin A receptor like type 1
Malignant neoplasm of breast	GPX4	P36969	glutathione peroxidase 4
Malignant neoplasm of breast	SREBF1	P36956	sterol regulatory element binding transcription factor 1
Malignant neoplasm of breast	SERPINF1	P36955	serpin family F member 1
Malignant neoplasm of breast	SERPINB5	P36952	serpin family B member 5
Malignant neoplasm of breast	LTBR	P36941	lymphotoxin beta receptor
Malignant neoplasm of breast	TGFBR1	P36897	transforming growth factor beta receptor 1
Malignant neoplasm of breast	BMPR1A	P36894	bone morphogenetic protein receptor type 1A
Malignant neoplasm of breast	FLT3	P36888	fms related receptor tyrosine kinase 3
Malignant neoplasm of breast	LONP1	P36776	lon peptidase 1, mitochondrial
Malignant neoplasm of breast	NUDT1	P36639	nudix hydrolase 1
Malignant neoplasm of breast	ARR3	P36575	arrestin 3
Malignant neoplasm of breast	CPOX	P36551	coproporphyrinogen oxidase
Malignant neoplasm of breast	TRIM23	P36406	tripartite motif containing 23
Malignant neoplasm of breast	ARL2	P36404	ADP ribosylation factor like GTPase 2
Malignant neoplasm of breast	TCF7	P36402	transcription factor 7

Malignant neoplasm of breast	CHI3L1	P36222	chitinase 3 like 1
Malignant neoplasm of breast	SLC16A2	P36021	solute carrier family 16 member 2
Malignant neoplasm of breast	KDR	P35968	kinase insert domain receptor
Malignant neoplasm of breast	FLT4	P35916	fms related receptor tyrosine kinase 4
Malignant neoplasm of breast	KRT2	P35908	keratin 2
Malignant neoplasm of breast	KRT20	P35900	keratin 20
Malignant neoplasm of breast	AHR	P35869	aryl hydrocarbon receptor
Malignant neoplasm of breast	CHKA	P35790	choline kinase alpha
Malignant neoplasm of breast	MYH11	P35749	myosin heavy chain 11
Malignant neoplasm of breast	SOX11	P35716	SRY-box transcription factor 11
Malignant neoplasm of breast	SOX18	P35713	SRY-box transcription factor 18
Malignant neoplasm of breast	SOX5	P35711	SRY-box transcription factor 5
Malignant neoplasm of breast	ATP7B	P35670	ATPase copper transporting beta
Malignant neoplasm of breast	DEK	P35659	DEK proto-oncogene
Malignant neoplasm of breast	NUP214	P35658	nucleoporin 214
Malignant neoplasm of breast	DDIT3	P35638	DNA damage inducible transcript 3
Malignant neoplasm of breast	FUS	P35637	FUS RNA binding protein
Malignant neoplasm of breast	GRK3	P35626	G protein-coupled receptor kinase 3
Malignant neoplasm of breast	TIMP3	P35625	TIMP metallopeptidase inhibitor 3

Malignant neoplasm of breast	BSG	P35613	basigin (Ok blood group)
Malignant neoplasm of breast	SOAT1	P35610	sterol O-acyltransferase 1
Malignant neoplasm of breast	TIE1	P35590	tyrosine kinase with immunoglobulin like and EGF like domains 1
Malignant neoplasm of breast	MYH10	P35580	myosin heavy chain 10
Malignant neoplasm of breast	MYH9	P35579	myosin heavy chain 9
Malignant neoplasm of breast	G6PC	P35575	glucose-6-phosphatase catalytic subunit
Malignant neoplasm of breast	IRS1	P35568	insulin receptor substrate 1
Malignant neoplasm of breast	FBN2	P35556	fibrillin 2
Malignant neoplasm of breast	FBN1	P35555	fibrillin 1
Malignant neoplasm of breast	MSX2	P35548	msh homeobox 2
Malignant neoplasm of breast	CLCN1	P35523	chloride voltage-gated channel 1
Malignant neoplasm of breast	IDUA	P35475	alpha-L-iduronidase
Malignant neoplasm of breast	DRD3	P35462	dopamine receptor D3
Malignant neoplasm of breast	HOXD13	P35453	homeobox D13
Malignant neoplasm of breast	THBS4	P35443	thrombospondin 4
Malignant neoplasm of breast	THBS2	P35442	thrombospondin 2
Malignant neoplasm of breast	PTGER4	P35408	prostaglandin E receptor 4
Malignant neoplasm of breast	RORA	P35398	RAR related orphan receptor A
Malignant neoplasm of breast	OPRM1	P35372	opioid receptor mu 1

Malignant neoplasm of breast	HRH1	P35367	histamine receptor H1
Malignant neoplasm of breast	PTGS2	P35354	prostaglandin-endoperoxide synthase 2
Malignant neoplasm of breast	ADRA1A	P35348	adrenoceptor alpha 1A
Malignant neoplasm of breast	SSTR5	P35346	somatostatin receptor 5
Malignant neoplasm of breast	SPRR2A	P35326	small proline rich protein 2A
Malignant neoplasm of breast	SPRR1A	P35321	small proline rich protein 1A
Malignant neoplasm of breast	ADM	P35318	adrenomedullin
Malignant neoplasm of breast	RFC1	P35251	replication factor C subunit 1
Malignant neoplasm of breast	RFC2	P35250	replication factor C subunit 2
Malignant neoplasm of breast	RFC4	P35249	replication factor C subunit 4
Malignant neoplasm of breast	SFTPD	P35247	surfactant protein D
Malignant neoplasm of breast	NF2	P35240	neurofibromin 2
Malignant neoplasm of breast	SERPINB6	P35237	serpin family B member 6
Malignant neoplasm of breast	PHB	P35232	prohibitin
Malignant neoplasm of breast	NOS2	P35228	nitric oxide synthase 2
Malignant neoplasm of breast	PCGF2	P35227	polycomb group ring finger 2
Malignant neoplasm of breast	COMMD3-BMI1	P35226	COMMD3-BMI1 readthrough
Malignant neoplasm of breast	BMI1	P35226	BMI1 proto-oncogene, polycomb ring finger
Malignant neoplasm of breast	IL13	P35225	interleukin 13

Malignant neoplasm of breast	CTNNB1	P35222	catenin beta 1
Malignant neoplasm of breast	CTNNA1	P35221	catenin alpha 1
Malignant neoplasm of breast	CA5A	P35218	carbonic anhydrase 5A
Malignant neoplasm of breast	USP6	P35125	ubiquitin specific peptidase 6
Malignant neoplasm of breast	PFN2	P35080	profilin 2
Malignant neoplasm of breast	BTC	P35070	betacellulin
Malignant neoplasm of breast	GPC1	P35052	glypican 1
Malignant neoplasm of breast	PRSS3	P35030	serine protease 3
Malignant neoplasm of breast	CRHR1	P34998	corticotropin releasing hormone receptor 1
Malignant neoplasm of breast	CNR2	P34972	cannabinoid receptor 2
Malignant neoplasm of breast	GRK5	P34947	G protein-coupled receptor kinase 5
Malignant neoplasm of breast	HSPA4	P34932	heat shock protein family A (Hsp70) member 4
Malignant neoplasm of breast	EVI2B	P34910	ecotropic viral integration site 2B
Malignant neoplasm of breast	GABRA3	P34903	gamma-aminobutyric acid type A receptor subunit alpha3
Malignant neoplasm of breast	SHMT2	P34897	serine hydroxymethyltransferase 2
Malignant neoplasm of breast	SHMT1	P34896	serine hydroxymethyltransferase 1
Malignant neoplasm of breast	BMP8B	P34820	bone morphogenetic protein 8b
Malignant neoplasm of breast	CD68	P34810	CD68 molecule
Malignant neoplasm of breast	SDC2	P34741	syndecan 2

Malignant neoplasm of breast	NTF4	P34130	neurotrophin 4
Malignant neoplasm of breast	GALNS	P34059	galactosamine (N-acetyl)-6-sulfatase
Malignant neoplasm of breast	MCM7	P33993	minichromosome maintenance complex component 7
Malignant neoplasm of breast	MCM5	P33992	minichromosome maintenance complex component 5
Malignant neoplasm of breast	TTK	P33981	TTK protein kinase
Malignant neoplasm of breast	MAN1A1	P33908	mannosidase alpha class 1A member 1
Malignant neoplasm of breast	CD80	P33681	CD80 molecule
Malignant neoplasm of breast	CKS2	P33552	CDC28 protein kinase regulatory subunit 2
Malignant neoplasm of breast	ABCC1	P33527	ATP binding cassette subfamily C member 1
Malignant neoplasm of breast	GUCY1A2	P33402	guanylate cyclase 1 soluble subunit alpha 2
Malignant neoplasm of breast	CYP2C19	P33261	cytochrome P450 family 2 subfamily C member 19
Malignant neoplasm of breast	CYP2C18	P33260	cytochrome P450 family 2 subfamily C member 18
Malignant neoplasm of breast	LSP1	P33241	lymphocyte specific protein 1
Malignant neoplasm of breast	KIF5B	P33176	kinesin family member 5B
Malignant neoplasm of breast	CDH5	P33151	cadherin 5
Malignant neoplasm of breast	ACSL1	P33121	acyl-CoA synthetase long chain family member 1
Malignant neoplasm of breast	CD70	P32970	CD70 molecule
Malignant neoplasm of breast	CTH	P32929	cystathionine gamma-lyase
Malignant neoplasm of breast	DSG3	P32926	desmoglein 3

Malignant neoplasm of breast	GTF2H1	P32780	general transcription factor IIH subunit 1
Malignant neoplasm of breast	HPD	P32754	4-hydroxyphenylpyruvate dioxygenase
Malignant neoplasm of breast	ELF1	P32519	E74 like ETS transcription factor 1
Malignant neoplasm of breast	GBP2	P32456	guanylate binding protein 2
Malignant neoplasm of breast	GBP1	P32455	guanylate binding protein 1
Malignant neoplasm of breast	SLC8A1	P32418	solute carrier family 8 member A1
Malignant neoplasm of breast	PYCR1	P32322	pyrroline-5-carboxylate reductase 1
Malignant neoplasm of breast	DCTD	P32321	dCMP deaminase
Malignant neoplasm of breast	CDA	P32320	cytidine deaminase
Malignant neoplasm of breast	FOXN2	P32314	forkhead box N2
Malignant neoplasm of breast	CXCR5	P32302	C-X-C motif chemokine receptor 5
Malignant neoplasm of breast	GRK4	P32298	G protein-coupled receptor kinase 4
Malignant neoplasm of breast	CCR7	P32248	C-C motif chemokine receptor 7
Malignant neoplasm of breast	BRS3	P32247	bombesin receptor subtype 3
Malignant neoplasm of breast	MC4R	P32245	melanocortin 4 receptor
Malignant neoplasm of breast	OTX2	P32243	orthodenticle homeobox 2
Malignant neoplasm of breast	OTX1	P32242	orthodenticle homeobox 1
Malignant neoplasm of breast	VIPR1	P32241	vasoactive intestinal peptide receptor 1
Malignant neoplasm of breast	CCKBR	P32239	cholecystokinin B receptor

Malignant neoplasm of breast	ARRB2	P32121	arrestin beta 2
Malignant neoplasm of breast	PRDX2	P32119	peroxiredoxin 2
Malignant neoplasm of breast	L1CAM	P32004	L1 cell adhesion molecule
Malignant neoplasm of breast	CEACAM8	P31997	CEA cell adhesion molecule 8
Malignant neoplasm of breast	S100A11	P31949	S100 calcium binding protein A11
Malignant neoplasm of breast	STIP1	P31948	stress induced phosphoprotein 1
Malignant neoplasm of breast	SFN	P31947	stratifin
Malignant neoplasm of breast	CASP14	P31944	caspase 14
Malignant neoplasm of breast	APOBEC3A	P31941	apolipoprotein B mRNA editing enzyme catalytic subunit 3A
Malignant neoplasm of breast	APOBEC3A_B	P31941	APOBEC3A and APOBEC3B deletion hybrid
Malignant neoplasm of breast	ATIC	P31939	5-aminoimidazole-4-carboxamide ribonucleotide formyltransferase/IMP cyclohydrolase
Malignant neoplasm of breast	AKT2	P31751	AKT serine/threonine kinase 2
Malignant neoplasm of breast	AKT1	P31749	AKT serine/threonine kinase 1
Malignant neoplasm of breast	SLC6A4	P31645	solute carrier family 6 member 4
Malignant neoplasm of breast	SLC6A6	P31641	solute carrier family 6 member 6
Malignant neoplasm of breast	SLC5A2	P31639	solute carrier family 5 member 2
Malignant neoplasm of breast	HIVEP2	P31629	HIVEP zinc finger 2
Malignant neoplasm of breast	SDC4	P31431	syndecan 4
Malignant neoplasm of breast	SSTR4	P31391	somatostatin receptor 4

Malignant neoplasm of breast	FGF9	P31371	fibroblast growth factor 9
Malignant neoplasm of breast	RRM2	P31350	ribonucleotide reductase regulatory subunit M2
Malignant neoplasm of breast	HOXD11	P31277	homeobox D11
Malignant neoplasm of breast	HOXC8	P31273	homeobox C8
Malignant neoplasm of breast	HOXA11	P31270	homeobox A11
Malignant neoplasm of breast	HOXA9	P31269	homeobox A9
Malignant neoplasm of breast	HOXA7	P31268	homeobox A7
Malignant neoplasm of breast	HOXA6	P31267	homeobox A6
Malignant neoplasm of breast	HOXA10	P31260	homeobox A10
Malignant neoplasm of breast	HOXD3	P31249	homeobox D3
Malignant neoplasm of breast	SRD5A2	P31213	steroid 5 alpha-reductase 2
Malignant neoplasm of breast	S100A7	P31151	S100 calcium binding protein A7
Malignant neoplasm of breast	CORO1A	P31146	coronin 1A
Malignant neoplasm of breast	SDHA	P31040	succinate dehydrogenase complex flavoprotein subunit A
Malignant neoplasm of breast	LCN1	P31025	lipocalin 1
Malignant neoplasm of breast	NTS	P30990	neurotensin
Malignant neoplasm of breast	NTSR1	P30989	neurotensin receptor 1
Malignant neoplasm of breast	CALCR	P30988	calcitonin receptor
Malignant neoplasm of breast	GNRHR	P30968	gonadotropin releasing hormone receptor

Malignant neoplasm of breast	SSTR2	P30874	somatostatin receptor 2
Malignant neoplasm of breast	SSTR1	P30872	somatostatin receptor 1
Malignant neoplasm of breast	ALDH3A1	P30838	aldehyde dehydrogenase 3 family member A1
Malignant neoplasm of breast	ALDH1B1	P30837	aldehyde dehydrogenase 1 family member B1
Malignant neoplasm of breast	SLC7A1	P30825	solute carrier family 7 member 1
Malignant neoplasm of breast	SERPINB1	P30740	serpin family B member 1
Malignant neoplasm of breast	GSTT1	P30711	glutathione S-transferase theta 1
Malignant neoplasm of breast	SRI	P30626	sorcin
Malignant neoplasm of breast	CLIP1	P30622	CAP-Gly domain containing linker protein 1
Malignant neoplasm of breast	PKLR	P30613	pyruvate kinase L/R
Malignant neoplasm of breast	ADSL	P30566	adenylosuccinate lyase
Malignant neoplasm of breast	OXTR	P30559	oxytocin receptor
Malignant neoplasm of breast	AGTR1	P30556	angiotensin II receptor type 1
Malignant neoplasm of breast	GRPR	P30550	gastrin releasing peptide receptor
Malignant neoplasm of breast	ADORA1	P30542	adenosine A1 receptor
Malignant neoplasm of breast	TSPO	P30536	translocator protein
Malignant neoplasm of breast	CHRNA5	P30532	cholinergic receptor nicotinic alpha 5 subunit
Malignant neoplasm of breast	AXL	P30530	AXL receptor tyrosine kinase
Malignant neoplasm of breast	AVPR2	P30518	arginine vasopressin receptor 2

Malignant neoplasm of breast	NMT1	P30419	N-myristoyltransferase 1
Malignant neoplasm of breast	TM4SF1	P30408	transmembrane 4 L six family member 1
Malignant neoplasm of breast	PPIF	P30405	peptidylprolyl isomerase F
Malignant neoplasm of breast	CDC25C	P30307	cell division cycle 25C
Malignant neoplasm of breast	CDC25B	P30305	cell division cycle 25B
Malignant neoplasm of breast	CDC25A	P30304	cell division cycle 25A
Malignant neoplasm of breast	MIP	P30301	major intrinsic protein of lens fiber
Malignant neoplasm of breast	WEE1	P30291	WEE1 G2 checkpoint kinase
Malignant neoplasm of breast	CCND3	P30281	cyclin D3
Malignant neoplasm of breast	CCND2	P30279	cyclin D2
Malignant neoplasm of breast	CDC27	P30260	cell division cycle 27
Malignant neoplasm of breast	CD6	P30203	CD6 molecule
Malignant neoplasm of breast	PPP2R1B	P30154	protein phosphatase 2 scaffold subunit Abeta
Malignant neoplasm of breast	PPP2R1A	P30153	protein phosphatase 2 scaffold subunit Aalpha
Malignant neoplasm of breast	PDIA3	P30101	protein disulfide isomerase family A member 3
Malignant neoplasm of breast	PEBP1	P30086	phosphatidylethanolamine binding protein 1
Malignant neoplasm of breast	CMPK1	P30085	cytidine/uridine monophosphate kinase 1
Malignant neoplasm of breast	ECHS1	P30084	enoyl-CoA hydratase, short chain 1
Malignant neoplasm of breast	PRDX3	P30048	peroxiredoxin 3

Malignant neoplasm of breast	DDT	P30046	D-dopachrome tautomerase
Malignant neoplasm of breast	PRDX5	P30044	peroxiredoxin 5
Malignant neoplasm of breast	PRDX6	P30041	peroxiredoxin 6
Malignant neoplasm of breast	ERP29	P30040	endoplasmic reticulum protein 29
Malignant neoplasm of breast	PBLD	P30039	phenazine biosynthesis like protein domain containing
Malignant neoplasm of breast	GNA11	P29992	G protein subunit alpha 11
Malignant neoplasm of breast	AQP1	P29972	aquaporin 1 (Colton blood group)
Malignant neoplasm of breast	MARCKS	P29966	myristoylated alanine rich protein kinase C substrate
Malignant neoplasm of breast	CD40LG	P29965	CD40 ligand
Malignant neoplasm of breast	CRABP1	P29762	cellular retinoic acid binding protein 1
Malignant neoplasm of breast	TYK2	P29597	tyrosine kinase 2
Malignant neoplasm of breast	PML	P29590	promyelocytic leukemia
Malignant neoplasm of breast	NOS1	P29475	nitric oxide synthase 1
Malignant neoplasm of breast	NOS3	P29474	nitric oxide synthase 3
Malignant neoplasm of breast	CASP1	P29466	caspase 1
Malignant neoplasm of breast	IL12B	P29460	interleukin 12B
Malignant neoplasm of breast	TKT	P29401	transketolase
Malignant neoplasm of breast	COL4A5	P29400	collagen type IV alpha 5 chain
Malignant neoplasm of breast	KDM5A	P29375	lysine demethylase 5A

Malignant neoplasm of breast	ARID4A	P29374	AT-rich interaction domain 4A
Malignant neoplasm of breast	CRABP2	P29373	cellular retinoic acid binding protein 2
Malignant neoplasm of breast	MPG	P29372	N-methylpurine DNA glycosylase
Malignant neoplasm of breast	SHC1	P29353	SHC adaptor protein 1
Malignant neoplasm of breast	PTPN6	P29350	protein tyrosine phosphatase non-receptor type 6
Malignant neoplasm of breast	EPHB2	P29323	EPH receptor B2
Malignant neoplasm of breast	EPHA3	P29320	EPH receptor A3
Malignant neoplasm of breast	EPHA2	P29317	EPH receptor A2
Malignant neoplasm of breast	CCN2	P29279	cellular communication network factor 2
Malignant neoplasm of breast	ADORA2B	P29275	adenosine A2b receptor
Malignant neoplasm of breast	PCSK6	P29122	proprotein convertase subtilisin/kexin type 6
Malignant neoplasm of breast	PCSK1	P29120	proprotein convertase subtilisin/kexin type 1
Malignant neoplasm of breast	S100A2	P29034	S100 calcium binding protein A2
Malignant neoplasm of breast	GJB2	P29033	gap junction protein beta 2
Malignant neoplasm of breast	TNFRSF8	P28908	TNF receptor superfamily member 8
Malignant neoplasm of breast	CD38	P28907	CD38 molecule
Malignant neoplasm of breast	CD34	P28906	CD34 molecule
Malignant neoplasm of breast	HSD11B1	P28845	hydroxysteroid 11-beta dehydrogenase 1
Malignant neoplasm of breast	LAP3	P28838	leucine aminopeptidase 3

Malignant neoplasm of breast	PTPRM	P28827	protein tyrosine phosphatase receptor type M
Malignant neoplasm of breast	GRN	P28799	granulin precursor
Malignant neoplasm of breast	RBL1	P28749	RB transcriptional corepressor like 1
Malignant neoplasm of breast	ERCC5	P28715	ERCC excision repair 5, endonuclease
Malignant neoplasm of breast	RXRB	P28702	retinoid X receptor beta
Malignant neoplasm of breast	MZF1	P28698	myeloid zinc finger 1
Malignant neoplasm of breast	DUSP1	P28562	dual specificity phosphatase 1
Malignant neoplasm of breast	MAPK1	P28482	mitogen-activated protein kinase 1
Malignant neoplasm of breast	SMARCA1	P28370	SWI/SNF related, matrix associated, actin dependent regulator of chromatin, subfamily a, member 1
Malignant neoplasm of breast	MSX1	P28360	msh homeobox 1
Malignant neoplasm of breast	HOXD10	P28358	homeobox D10
Malignant neoplasm of breast	TEAD1	P28347	TEA domain transcription factor 1
Malignant neoplasm of breast	POLD1	P28340	DNA polymerase delta 1, catalytic subunit
Malignant neoplasm of breast	NMBR	P28336	neuromedin B receptor
Malignant neoplasm of breast	ELK4	P28324	ETS transcription factor ELK4
Malignant neoplasm of breast	LOX	P28300	lysyl oxidase
Malignant neoplasm of breast	TMOD1	P28289	tropomodulin 1
Malignant neoplasm of breast	HTR2A	P28223	5-hydroxytryptamine receptor 2A
Malignant neoplasm of breast	HTR1B	P28222	5-hydroxytryptamine receptor 1B

Malignant neoplasm of breast	GSTM2	P28161	glutathione S-transferase mu 2
Malignant neoplasm of breast	PSMB5	P28074	proteasome 20S subunit beta 5
Malignant neoplasm of breast	PSMB4	P28070	proteasome 20S subunit beta 4
Malignant neoplasm of breast	POU1F1	P28069	POU class 1 homeobox 1
Malignant neoplasm of breast	PSMB9	P28065	proteasome 20S subunit beta 9
Malignant neoplasm of breast	PSMB8	P28062	proteasome 20S subunit beta 8
Malignant neoplasm of breast	PIK3R1	P27986	phosphoinositide-3-kinase regulatory subunit 1
Malignant neoplasm of breast	CFP	P27918	complement factor properdin
Malignant neoplasm of breast	CANX	P27824	calnexin
Malignant neoplasm of breast	PDE4A	P27815	phosphodiesterase 4A
Malignant neoplasm of breast	CALR	P27797	calreticulin
Malignant neoplasm of breast	CAD	P27708	carbamoyl-phosphate synthetase 2, aspartate transcarbamylase, and dihydroorotase
Malignant neoplasm of breast	DCK	P27707	deoxycytidine kinase
Malignant neoplasm of breast	CD82	P27701	CD82 molecule
Malignant neoplasm of breast	APEX1	P27695	apurinic/apyrimidinic endodeoxyribonuclease 1
Malignant neoplasm of breast	RPA1	P27694	replication protein A1
Malignant neoplasm of breast	RPL10	P27635	ribosomal protein L10
Malignant neoplasm of breast	ARNT	P27540	aryl hydrocarbon receptor nuclear translocator
Malignant neoplasm of breast	GDF1	P27539	growth differentiation factor 1

Malignant neoplasm of breast	DPP4	P27487	dipeptidyl peptidase 4
Malignant neoplasm of breast	CALML3	P27482	calmodulin like 3
Malignant neoplasm of breast	G0S2	P27469	G0/G1 switch 2
Malignant neoplasm of breast	MAPK3	P27361	mitogen-activated protein kinase 3
Malignant neoplasm of breast	ANXA13	P27216	annexin A13
Malignant neoplasm of breast	PON1	P27169	paraoxonase 1
Malignant neoplasm of breast	AK4	P27144	adenylate kinase 4
Malignant neoplasm of breast	ACVR2A	P27037	activin A receptor type 2A
Malignant neoplasm of breast	MST1	P26927	macrophage stimulating 1
Malignant neoplasm of breast	CD27	P26842	CD27 molecule
Malignant neoplasm of breast	KLRC4-KLRK1	P26718	KLRC4-KLRK1 readthrough
Malignant neoplasm of breast	KLRK1	P26718	killer cell lectin like receptor K1
Malignant neoplasm of breast	ZFP36	P26651	ZFP36 ring finger protein
Malignant neoplasm of breast	PTBP1	P26599	polypyrimidine tract binding protein 1
Malignant neoplasm of breast	HMGB2	P26583	high mobility group box 2
Malignant neoplasm of breast	MGAT1	P26572	alpha-1,3-mannosyl-glycoprotein 2-beta-N-acetylglucosaminyltransferase
Malignant neoplasm of breast	S100A4	P26447	S100 calcium binding protein A4
Malignant neoplasm of breast	RPL13	P26373	ribosomal protein L13
Malignant neoplasm of breast	PAX6	P26367	paired box 6

Malignant neoplasm of breast	DNMT1	P26358	DNA methyltransferase 1
Malignant neoplasm of breast	PTPN3	P26045	protein tyrosine phosphatase non-receptor type 3
Malignant neoplasm of breast	MSN	P26038	moesin
Malignant neoplasm of breast	PTX3	P26022	pentraxin 3
Malignant neoplasm of breast	NFKBIA	P25963	NFKB inhibitor alpha
Malignant neoplasm of breast	CD40	P25942	CD40 molecule
Malignant neoplasm of breast	LMO2	P25791	LIM domain only 2
Malignant neoplasm of breast	CTSS	P25774	cathepsin S
Malignant neoplasm of breast	MT3	P25713	metallothionein 3
Malignant neoplasm of breast	DNAJB1	P25685	DnaJ heat shock protein family (Hsp40) member B1
Malignant neoplasm of breast	YY1	P25490	YY1 transcription factor
Malignant neoplasm of breast	FAS	P25445	Fas cell surface death receptor
Malignant neoplasm of breast	BRD2	P25440	bromodomain containing 2
Malignant neoplasm of breast	MPST	P25325	mercaptopyruvate sulfurtransferase
Malignant neoplasm of breast	AZGP1	P25311	alpha-2-glycoprotein 1, zinc-binding
Malignant neoplasm of breast	MPZ	P25189	myelin protein zero
Malignant neoplasm of breast	F2R	P25116	coagulation factor II thrombin receptor
Malignant neoplasm of breast	ACKR3	P25106	atypical chemokine receptor 3
Malignant neoplasm of breast	TACR1	P25103	tachykinin receptor 1

Malignant neoplasm of breast	EDNRA	P25101	endothelin receptor type A
Malignant neoplasm of breast	ADRA1D	P25100	adrenoceptor alpha 1D
Malignant neoplasm of breast	GRK2	P25098	G protein-coupled receptor kinase 2
Malignant neoplasm of breast	CD24	P25063	CD24 molecule
Malignant neoplasm of breast	APC	P25054	APC regulator of WNT signaling pathway
Malignant neoplasm of breast	CXCR2	P25025	C-X-C motif chemokine receptor 2
Malignant neoplasm of breast	CXCR1	P25024	C-X-C motif chemokine receptor 1
Malignant neoplasm of breast	CDK2	P24941	cyclin dependent kinase 2
Malignant neoplasm of breast	CCNE1	P24864	cyclin E1
Malignant neoplasm of breast	CCNC	P24863	cyclin C
Malignant neoplasm of breast	DNASE1	P24855	deoxyribonuclease 1
Malignant neoplasm of breast	TNC	P24821	tenascin C
Malignant neoplasm of breast	PRKCH	P24723	protein kinase C eta
Malignant neoplasm of breast	ACP1	P24666	acid phosphatase 1
Malignant neoplasm of breast	IGFBP5	P24593	insulin like growth factor binding protein 5
Malignant neoplasm of breast	TBXAS1	P24557	thromboxane A synthase 1
Malignant neoplasm of breast	EEF1B2	P24534	eukaryotic translation elongation factor 1 beta 2
Malignant neoplasm of breast	EDNRB	P24530	endothelin receptor type B
Malignant neoplasm of breast	GADD45A	P24522	growth arrest and DNA damage inducible alpha

Malignant neoplasm of breast	NR2F2	P24468	nuclear receptor subfamily 2 group F member 2
Malignant neoplasm of breast	CYP3A7-CYP3A51P	P24462	CYP3A7-CYP3A51P readthrough
Malignant neoplasm of breast	CYP3A7	P24462	cytochrome P450 family 3 subfamily A member 7
Malignant neoplasm of breast	IL4R	P24394	interleukin 4 receptor
Malignant neoplasm of breast	CHM	P24386	CHM Rab escort protein
Malignant neoplasm of breast	CCND1	P24385	cyclin D1
Malignant neoplasm of breast	MMP11	P24347	matrix metallopeptidase 11
Malignant neoplasm of breast	GPT	P24298	glutamic--pyruvic transaminase
Malignant neoplasm of breast	LAMA2	P24043	laminin subunit alpha 2
Malignant neoplasm of breast	IL32	P24001	interleukin 32
Malignant neoplasm of breast	SLC6A2	P23975	solute carrier family 6 member 2
Malignant neoplasm of breast	CMA1	P23946	chymase 1
Malignant neoplasm of breast	FSHR	P23945	follicle stimulating hormone receptor
Malignant neoplasm of breast	RRM1	P23921	ribonucleotide reductase catalytic subunit M1
Malignant neoplasm of breast	CPT2	P23786	carnitine palmitoyltransferase 2
Malignant neoplasm of breast	GATA3	P23771	GATA binding protein 3
Malignant neoplasm of breast	GATA2	P23769	GATA binding protein 2
Malignant neoplasm of breast	VAMP1	P23763	vesicle associated membrane protein 1
Malignant neoplasm of breast	DGKA	P23743	diacylglycerol kinase alpha

Malignant neoplasm of breast	ITPKA	P23677	inositol-trisphosphate 3-kinase A
Malignant neoplasm of breast	ATP2B4	P23634	ATPase plasma membrane Ca2+ transporting 4
Malignant neoplasm of breast	EIF4B	P23588	eukaryotic translation initiation factor 4B
Malignant neoplasm of breast	BDNF	P23560	brain derived neurotrophic factor
Malignant neoplasm of breast	CFL1	P23528	cofilin 1
Malignant neoplasm of breast	AHCY	P23526	adenosylhomocysteinase
Malignant neoplasm of breast	NFYA	P23511	nuclear transcription factor Y subunit alpha
Malignant neoplasm of breast	TNFSF4	P23510	TNF superfamily member 4
Malignant neoplasm of breast	MCC	P23508	MCC regulator of WNT signaling pathway
Malignant neoplasm of breast	PTPRG	P23470	protein tyrosine phosphatase receptor type G
Malignant neoplasm of breast	PTPRD	P23468	protein tyrosine phosphatase receptor type D
Malignant neoplasm of breast	JAK1	P23458	Janus kinase 1
Malignant neoplasm of breast	RPS6KB1	P23443	ribosomal protein S6 kinase B1
Malignant neoplasm of breast	GCSH	P23434	glycine cleavage system protein H
Malignant neoplasm of breast	WARS1	P23381	tryptophanyl-tRNA synthetase 1
Malignant neoplasm of breast	S100A1	P23297	S100 calcium binding protein A1
Malignant neoplasm of breast	PPIB	P23284	peptidylprolyl isomerase B
Malignant neoplasm of breast	TUBG1	P23258	tubulin gamma 1
Malignant neoplasm of breast	ITGA6	P23229	integrin subunit alpha 6

Malignant neoplasm of breast	PTGS1	P23219	prostaglandin-endoperoxide synthase 1
Malignant neoplasm of breast	TCEA1	P23193	transcription elongation factor A1
Malignant neoplasm of breast	FBLN1	P23142	fibulin 1
Malignant neoplasm of breast	AMPD1	P23109	adenosine monophosphate deaminase 1
Malignant neoplasm of breast	XPA	P23025	XPA, DNA damage recognition and repair factor
Malignant neoplasm of breast	MRC1	P22897	mannose receptor C-type 1
Malignant neoplasm of breast	MMP8	P22894	matrix metallopeptidase 8
Malignant neoplasm of breast	LHCGR	P22888	luteinizing hormone/choriogonadotropin receptor
Malignant neoplasm of breast	FECH	P22830	ferrochelatase
Malignant neoplasm of breast	AADAC	P22760	arylacetamide deacetylase
Malignant neoplasm of breast	GNLY	P22749	granulysin
Malignant neoplasm of breast	NR4A1	P22736	nuclear receptor subfamily 4 group A member 1
Malignant neoplasm of breast	SLC2A5	P22732	solute carrier family 2 member 5
Malignant neoplasm of breast	IGFBP4	P22692	insulin like growth factor binding protein 4
Malignant neoplasm of breast	CBL	P22681	Cbl proto-oncogene
Malignant neoplasm of breast	RFX1	P22670	regulatory factor X1
Malignant neoplasm of breast	HNRNPA2B1	P22626	heterogeneous nuclear ribonucleoprotein A2/B1
Malignant neoplasm of breast	PRKACG	P22612	protein kinase cAMP-activated catalytic subunit gamma
Malignant neoplasm of breast	FGFR3	P22607	fibroblast growth factor receptor 3

Malignant neoplasm of breast	FDXR	P22570	ferredoxin reductase
Malignant neoplasm of breast	GAL	P22466	galanin and GMAP prepropeptide
Malignant neoplasm of breast	KCNA5	P22460	potassium voltage-gated channel subfamily A member 5
Malignant neoplasm of breast	FGFR4	P22455	fibroblast growth factor receptor 4
Malignant neoplasm of breast	USF1	P22415	upstream transcription factor 1
Malignant neoplasm of breast	ENPP1	P22413	ectonucleotide pyrophosphatase/phosphodiesterase 1
Malignant neoplasm of breast	NME2	P22392	NME/NM23 nucleoside diphosphate kinase 2
Malignant neoplasm of breast	NME1-NME2	P22392	NME1-NME2 readthrough
Malignant neoplasm of breast	CCL1	P22362	C-C motif chemokine ligand 1
Malignant neoplasm of breast	GPX3	P22352	glutathione peroxidase 3
Malignant neoplasm of breast	UGT1A4	P22310	UDP glucuronosyltransferase family 1 member A4
Malignant neoplasm of breast	UGT1A1	P22309	UDP glucuronosyltransferase family 1 member A1
Malignant neoplasm of breast	SCP2	P22307	sterol carrier protein 2
Malignant neoplasm of breast	IDS	P22304	iduronate 2-sulfatase
Malignant neoplasm of breast	ACHE	P22303	acetylcholinesterase (Cartwright blood group)
Malignant neoplasm of breast	IL10	P22301	interleukin 10
Malignant neoplasm of breast	PAICS	P22234	phosphoribosylaminoimidazole carboxylase and phosphoribosylaminoimidazolesuccinocarboxamide synthase
Malignant neoplasm of breast	CDH3	P22223	cadherin 3
Malignant neoplasm of breast	FBL	P22087	fibrillarin

Malignant neoplasm of breast	FUT4	P22083	fucosyltransferase 4
Malignant neoplasm of breast	LPO	P22079	lactoperoxidase
Malignant neoplasm of breast	MMUT	P22033	methylmalonyl-CoA mutase
Malignant neoplasm of breast	BMP6	P22004	bone morphogenetic protein 6
Malignant neoplasm of breast	BMP5	P22003	bone morphogenetic protein 5
Malignant neoplasm of breast	TGM2	P21980	transglutaminase 2
Malignant neoplasm of breast	COMT	P21964	catechol-O-methyltransferase
Malignant neoplasm of breast	MATN1	P21941	matrilin 1
Malignant neoplasm of breast	CD9	P21926	CD9 molecule
Malignant neoplasm of breast	DRD5	P21918	dopamine receptor D5
Malignant neoplasm of breast	DRD4	P21917	dopamine receptor D4
Malignant neoplasm of breast	SDHB	P21912	succinate dehydrogenase complex iron sulfur subunit B
Malignant neoplasm of breast	ERBB3	P21860	erb-b2 receptor tyrosine kinase 3
Malignant neoplasm of breast	IBSP	P21815	integrin binding sialoprotein
Malignant neoplasm of breast	BGN	P21810	biglycan
Malignant neoplasm of breast	FGFR2	P21802	fibroblast growth factor receptor 2
Malignant neoplasm of breast	VDAC1	P21796	voltage dependent anion channel 1
Malignant neoplasm of breast	FGF7	P21781	fibroblast growth factor 7
Malignant neoplasm of breast	MSR1	P21757	macrophage scavenger receptor 1

Malignant neoplasm of breast	MDK	P21741	midkine
Malignant neoplasm of breast	TBXA2R	P21731	thromboxane A2 receptor
Malignant neoplasm of breast	C5AR1	P21730	complement C5a receptor 1
Malignant neoplasm of breast	DRD1	P21728	dopamine receptor D1
Malignant neoplasm of breast	EPHA1	P21709	EPH receptor A1
Malignant neoplasm of breast	GPD1	P21695	glycerol-3-phosphate dehydrogenase 1
Malignant neoplasm of breast	SAT1	P21673	spermidine/spermine N1-acetyltransferase 1
Malignant neoplasm of breast	NT5E	P21589	5'-nucleotidase ecto
Malignant neoplasm of breast	KITLG	P21583	KIT ligand
Malignant neoplasm of breast	TNFAIP3	P21580	TNF alpha induced protein 3
Malignant neoplasm of breast	SYT1	P21579	synaptotagmin 1
Malignant neoplasm of breast	CNR1	P21554	cannabinoid receptor 1
Malignant neoplasm of breast	FPR1	P21462	formyl peptide receptor 1
Malignant neoplasm of breast	S1PR1	P21453	sphingosine-1-phosphate receptor 1
Malignant neoplasm of breast	ABCB4	P21439	ATP binding cassette subfamily B member 4
Malignant neoplasm of breast	MAOA	P21397	monoamine oxidase A
Malignant neoplasm of breast	NF1	P21359	neurofibromin 1
Malignant neoplasm of breast	FLNA	P21333	filamin A
Malignant neoplasm of breast	ATP6V1C1	P21283	ATPase H+ transporting V1 subunit C1

Malignant neoplasm of breast	GSTM3	P21266	glutathione S-transferase mu 3
Malignant neoplasm of breast	PTN	P21246	pleiotrophin
Malignant neoplasm of breast	FUT3	P21217	fucosyltransferase 3 (Lewis blood group)
Malignant neoplasm of breast	MAL	P21145	mal, T cell differentiation protein
Malignant neoplasm of breast	CDK11B	P21127	cyclin dependent kinase 11B
Malignant neoplasm of breast	PDC	P20941	phosducin
Malignant neoplasm of breast	RASA1	P20936	RAS p21 protein activator 1
Malignant neoplasm of breast	FLG	P20930	filaggrin
Malignant neoplasm of breast	COL5A1	P20908	collagen type V alpha 1 chain
Malignant neoplasm of breast	CYP2A7	P20853	cytochrome P450 family 2 subfamily A member 7
Malignant neoplasm of breast	IMPDH1	P20839	inosine monophosphate dehydrogenase 1
Malignant neoplasm of breast	EFNA1	P20827	ephrin A1
Malignant neoplasm of breast	HNF1A	P20823	HNF1 homeobox A
Malignant neoplasm of breast	CYP3A5	P20815	cytochrome P450 family 3 subfamily A member 5
Malignant neoplasm of breast	CYP2B6	P20813	cytochrome P450 family 2 subfamily B member 6
Malignant neoplasm of breast	CAST	P20810	calpastatin
Malignant neoplasm of breast	IL11	P20809	interleukin 11
Malignant neoplasm of breast	OGN	P20774	osteoglycin
Malignant neoplasm of breast	BCL3	P20749	BCL3 transcription coactivator

Malignant neoplasm of breast	HOXA5	P20719	homeobox A5
Malignant neoplasm of breast	DDC	P20711	dopa decarboxylase
Malignant neoplasm of breast	ITGAX	P20702	integrin subunit alpha X
Malignant neoplasm of breast	LMNB1	P20700	lamin B1
Malignant neoplasm of breast	COX5A	P20674	cytochrome c oxidase subunit 5A
Malignant neoplasm of breast	MSH3	P20585	mutS homolog 3
Malignant neoplasm of breast	TRH	P20396	thyrotropin releasing hormone
Malignant neoplasm of breast	NR1D1	P20393	nuclear receptor subfamily 1 group D member 1
Malignant neoplasm of breast	TAC1	P20366	tachykinin precursor 1
Malignant neoplasm of breast	RAB5A	P20339	RAB5A, member RAS oncogene family
Malignant neoplasm of breast	RAB3A	P20336	RAB3A, member RAS oncogene family
Malignant neoplasm of breast	TNFRSF1B	P20333	TNF receptor superfamily member 1B
Malignant neoplasm of breast	ALOX5AP	P20292	arachidonate 5-lipoxygenase activating protein
Malignant neoplasm of breast	BTF3	P20290	basic transcription factor 3
Malignant neoplasm of breast	CD22	P20273	CD22 molecule
Malignant neoplasm of breast	CCNA2	P20248	cyclin A2
Malignant neoplasm of breast	TBP	P20226	TATA-box binding protein
Malignant neoplasm of breast	AZU1	P20160	azurocidin 1
Malignant neoplasm of breast	KLK2	P20151	kallikrein related peptidase 2

Malignant neoplasm of breast	PGC	P20142	progastricsin
Malignant neoplasm of breast	CD33	P20138	CD33 molecule
Malignant neoplasm of breast	SDS	P20132	serine dehydratase
Malignant neoplasm of breast	ANXA7	P20073	annexin A7
Malignant neoplasm of breast	TCN1	P20061	transcobalamin 1
Malignant neoplasm of breast	EIF2S2	P20042	eukaryotic translation initiation factor 2 subunit beta
Malignant neoplasm of breast	ATP2B1	P20020	ATPase plasma membrane Ca2+ transporting 1
Malignant neoplasm of breast	TYMP	P19971	thymidine phosphorylase
Malignant neoplasm of breast	PI3	P19957	peptidase inhibitor 3
Malignant neoplasm of breast	FST	P19883	follistatin
Malignant neoplasm of breast	CXCL3	P19876	C-X-C motif chemokine ligand 3
Malignant neoplasm of breast	CXCL2	P19875	C-X-C motif chemokine ligand 2
Malignant neoplasm of breast	NFKB1	P19838	nuclear factor kappa B subunit 1
Malignant neoplasm of breast	CEL	P19835	carboxyl ester lipase
Malignant neoplasm of breast	ITIH2	P19823	inter-alpha-trypsin inhibitor heavy chain 2
Malignant neoplasm of breast	AOC1	P19801	amine oxidase copper containing 1
Malignant neoplasm of breast	RXRA	P19793	retinoid X receptor alpha
Malignant neoplasm of breast	CSNK2A2	P19784	casein kinase 2 alpha 2
Malignant neoplasm of breast	SLC9A1	P19634	solute carrier family 9 member A1

Malignant neoplasm of breast	EN2	P19622	engrailed homeobox 2
Malignant neoplasm of breast	WT1	P19544	WT1 transcription factor
Malignant neoplasm of breast	TFE3	P19532	transcription factor binding to IGHM enhancer 3
Malignant neoplasm of breast	FUT1	P19526	fucosyltransferase 1 (H blood group)
Malignant neoplasm of breast	EIF2AK2	P19525	eukaryotic translation initiation factor 2 alpha kinase 2
Malignant neoplasm of breast	TFEB	P19484	transcription factor EB
Malignant neoplasm of breast	TRIM21	P19474	tripartite motif containing 21
Malignant neoplasm of breast	ERCC3	P19447	ERCC excision repair 3, TFIIH core complex helicase subunit
Malignant neoplasm of breast	TNFRSF1A	P19438	TNF receptor superfamily member 1A
Malignant neoplasm of breast	ELK1	P19419	ETS transcription factor ELK1
Malignant neoplasm of breast	HK1	P19367	hexokinase 1
Malignant neoplasm of breast	NCL	P19338	nucleolin
Malignant neoplasm of breast	VCAM1	P19320	vascular cell adhesion molecule 1
Malignant neoplasm of breast	EPOR	P19235	erythropoietin receptor
Malignant neoplasm of breast	UGT1A6	P19224	UDP glucuronosyltransferase family 1 member A6
Malignant neoplasm of breast	PLCG1	P19174	phospholipase C gamma 1
Malignant neoplasm of breast	HDC	P19113	histidine decarboxylase
Malignant neoplasm of breast	TSPAN8	P19075	tetraspanin 8
Malignant neoplasm of breast	CDH2	P19022	cadherin 2

Malignant neoplasm of breast	PAM	P19021	peptidylglycine alpha-amidating monooxygenase
Malignant neoplasm of breast	XRCC1	P18887	X-ray repair cross complementing 1
Malignant neoplasm of breast	LIG1	P18858	DNA ligase 1
Malignant neoplasm of breast	ATF6	P18850	activating transcription factor 6
Malignant neoplasm of breast	ATF4	P18848	activating transcription factor 4
Malignant neoplasm of breast	ATF3	P18847	activating transcription factor 3
Malignant neoplasm of breast	SDC1	P18827	syndecan 1
Malignant neoplasm of breast	ADRA2C	P18825	adrenoceptor alpha 2C
Malignant neoplasm of breast	RCC1	P18754	regulator of chromosome condensation 1
Malignant neoplasm of breast	PGAM1	P18669	phosphoglycerate mutase 1
Malignant neoplasm of breast	LAG3	P18627	lymphocyte activating 3
Malignant neoplasm of breast	RPL17	P18621	ribosomal protein L17
Malignant neoplasm of breast	ITGB6	P18564	integrin subunit beta 6
Malignant neoplasm of breast	IL1RN	P18510	interleukin 1 receptor antagonist
Malignant neoplasm of breast	NAT1	P18440	N-acetyltransferase 1
Malignant neoplasm of breast	PTPRA	P18433	protein tyrosine phosphatase receptor type A
Malignant neoplasm of breast	LBP	P18428	lipopolysaccharide binding protein
Malignant neoplasm of breast	SRD5A1	P18405	steroid 5 alpha-reductase 1
Malignant neoplasm of breast	GPX2	P18283	glutathione peroxidase 2

Malignant neoplasm of breast	VCL	P18206	vinculin
Malignant neoplasm of breast	EGR1	P18146	early growth response 1
Malignant neoplasm of breast	ADRA2B	P18089	adrenoceptor alpha 2B
Malignant neoplasm of breast	ARF4	P18085	ADP ribosylation factor 4
Malignant neoplasm of breast	ITGB5	P18084	integrin subunit beta 5
Malignant neoplasm of breast	BMP7	P18075	bone morphogenetic protein 7
Malignant neoplasm of breast	ERCC2	P18074	ERCC excision repair 2, TFIIH core complex helicase subunit
Malignant neoplasm of breast	IGFBP2	P18065	insulin like growth factor binding protein 2
Malignant neoplasm of breast	ALOX12	P18054	arachidonate 12-lipoxygenase, 12S type
Malignant neoplasm of breast	PTPN1	P18031	protein tyrosine phosphatase non-receptor type 1
Malignant neoplasm of breast	TCP1	P17987	t-complex 1
Malignant neoplasm of breast	PSMC3	P17980	proteasome 26S subunit, ATPase 3
Malignant neoplasm of breast	FLT1	P17948	fms related receptor tyrosine kinase 1
Malignant neoplasm of breast	IGFBP3	P17936	insulin like growth factor binding protein 3
Malignant neoplasm of breast	LGALS3	P17931	galectin 3
Malignant neoplasm of breast	CR1	P17927	complement C3b/C4b receptor 1 (Knops blood group)
Malignant neoplasm of breast	GM2A	P17900	GM2 ganglioside activator
Malignant neoplasm of breast	XBP1	P17861	X-box binding protein 1
Malignant neoplasm of breast	DDX5	P17844	DEAD-box helicase 5

Malignant neoplasm of breast	ENG	P17813	endoglin
Malignant neoplasm of breast	TPH1	P17752	tryptophan hydroxylase 1
Malignant neoplasm of breast	TAT	P17735	tyrosine aminotransferase
Malignant neoplasm of breast	AMD1	P17707	adenosylmethionine decarboxylase 1
Malignant neoplasm of breast	PTPN2	P17706	protein tyrosine phosphatase non-receptor type 2
Malignant neoplasm of breast	HLA-G	P17693	major histocompatibility complex, class I, G
Malignant neoplasm of breast	CEBPB	P17676	CCAAT enhancer binding protein beta
Malignant neoplasm of breast	DES	P17661	desmin
Malignant neoplasm of breast	CAPN2	P17655	calpain 2
Malignant neoplasm of breast	TYRP1	P17643	tyrosinase related protein 1
Malignant neoplasm of breast	PRKACA	P17612	protein kinase cAMP-activated catalytic subunit alpha
Malignant neoplasm of breast	SYN1	P17600	synapsin I
Malignant neoplasm of breast	TAL1	P17542	TAL bHLH transcription factor 1, erythroid differentiation factor
Malignant neoplasm of breast	JUND	P17535	JunD proto-oncogene, AP-1 transcription factor subunit
Malignant neoplasm of breast	AKR1C4	P17516	aldo-keto reductase family 1 member C4
Malignant neoplasm of breast	HOXB4	P17483	homeobox B4
Malignant neoplasm of breast	HOXB9	P17482	homeobox B9
Malignant neoplasm of breast	HOXB8	P17481	homeobox B8
Malignant neoplasm of breast	GJA1	P17302	gap junction protein alpha 1

Malignant neoplasm of breast	ITGA2	P17301	integrin subunit alpha 2
Malignant neoplasm of breast	JUNB	P17275	JunB proto-oncogene, AP-1 transcription factor subunit
Malignant neoplasm of breast	PRKCA	P17252	protein kinase C alpha
Malignant neoplasm of breast	IFNAR1	P17181	interferon alpha and beta receptor subunit 1
Malignant neoplasm of breast	GOT1	P17174	glutamic-oxaloacetic transaminase 1
Malignant neoplasm of breast	HMGA1	P17096	high mobility group AT-hook 1
Malignant neoplasm of breast	RHOQ	P17081	ras homolog family member Q
Malignant neoplasm of breast	ZNF32	P17041	zinc finger protein 32
Malignant neoplasm of breast	ZNF25	P17030	zinc finger protein 25
Malignant neoplasm of breast	ZNF24	P17028	zinc finger protein 24
Malignant neoplasm of breast	ZNF23	P17027	zinc finger protein 23
Malignant neoplasm of breast	ZNF22	P17026	zinc finger protein 22
Malignant neoplasm of breast	ZFX	P17010	zinc finger protein X-linked
Malignant neoplasm of breast	YBX3	P16989	Y-box binding protein 3
Malignant neoplasm of breast	STMN1	P16949	stathmin 1
Malignant neoplasm of breast	FAH	P16930	fumarylacetoacetate hydrolase
Malignant neoplasm of breast	PLCG2	P16885	phospholipase C gamma 2
Malignant neoplasm of breast	IL7R	P16871	interleukin 7 receptor
Malignant neoplasm of breast	NPPB	P16860	natriuretic peptide B

Malignant neoplasm of breast	CD36	P16671	CD36 molecule
Malignant neoplasm of breast	UGT2B7	P16662	UDP glucuronosyltransferase family 2 member B7
Malignant neoplasm of breast	ATP2A2	P16615	ATPase sarcoplasmic/endoplasmic reticulum Ca2+ transporting 2
Malignant neoplasm of breast	SELE	P16581	selectin E
Malignant neoplasm of breast	CRISP2	P16562	cysteine rich secretory protein 2
Malignant neoplasm of breast	GNB3	P16520	G protein subunit beta 3
Malignant neoplasm of breast	TSHR	P16473	thyroid stimulating hormone receptor
Malignant neoplasm of breast	PRLR	P16471	prolactin receptor
Malignant neoplasm of breast	MGMT	P16455	O-6-methylguanine-DNA methyltransferase
Malignant neoplasm of breast	DPEP1	P16444	dipeptidase 1
Malignant neoplasm of breast	ABO	P16442	ABO, alpha 1-3-N-acetylgalactosaminyltransferase and alpha 1-3-galactosyltransferase
Malignant neoplasm of breast	POR	P16435	cytochrome p450 oxidoreductase
Malignant neoplasm of breast	EPCAM	P16422	epithelial cell adhesion molecule
Malignant neoplasm of breast	CTLA4	P16410	cytotoxic T-lymphocyte associated protein 4
Malignant neoplasm of breast	H1-2	P16403	H1.2 linker histone, cluster member
Malignant neoplasm of breast	PECAM1	P16284	platelet and endothelial cell adhesion molecule 1
Malignant neoplasm of breast	GLB1	P16278	galactosidase beta 1
Malignant neoplasm of breast	SLC25A16	P16260	solute carrier family 25 member 16
Malignant neoplasm of breast	PDGFRA	P16234	platelet derived growth factor receptor alpha

Malignant neoplasm of breast	CREB1	P16220	cAMP responsive element binding protein 1
Malignant neoplasm of breast	ANK1	P16157	ankyrin 1
Malignant neoplasm of breast	CBR1	P16152	carbonyl reductase 1
Malignant neoplasm of breast	SPN	P16150	sialophorin
Malignant neoplasm of breast	ITGB4	P16144	integrin subunit beta 4
Malignant neoplasm of breast	SELP	P16109	selectin P
Malignant neoplasm of breast	H2AX	P16104	H2A.X variant histone
Malignant neoplasm of breast	NQO2	P16083	N-ribosyldihydronicotinamide:quinone reductase 2
Malignant neoplasm of breast	CD44	P16070	CD44 molecule (Indian blood group)
Malignant neoplasm of breast	NPR1	P16066	natriuretic peptide receptor 1
Malignant neoplasm of breast	ALOX15	P16050	arachidonate 15-lipoxygenase
Malignant neoplasm of breast	TIMP2	P16035	TIMP metallopeptidase inhibitor 2
Malignant neoplasm of breast	GATA1	P15976	GATA binding protein 1
Malignant neoplasm of breast	MUC1	P15941	mucin 1, cell surface associated
Malignant neoplasm of breast	RPA2	P15927	replication protein A2
Malignant neoplasm of breast	TCF3	P15923	transcription factor 3
Malignant neoplasm of breast	ST6GAL1	P15907	ST6 beta-galactoside alpha-2,6-sialyltransferase 1
Malignant neoplasm of breast	TCF4	P15884	transcription factor 4
Malignant neoplasm of breast	HIVEP1	P15822	HIVEP zinc finger 1

Malignant neoplasm of breast	CD1D	P15813	CD1d molecule
Malignant neoplasm of breast	PHKG2	P15735	phosphorylase kinase catalytic subunit gamma 2
Malignant neoplasm of breast	VEGFA	P15692	vascular endothelial growth factor A
Malignant neoplasm of breast	ZNF44	P15621	zinc finger protein 44
Malignant neoplasm of breast	NQO1	P15559	NAD(P)H quinone dehydrogenase 1
Malignant neoplasm of breast	CYP11B1	P15538	cytochrome P450 family 11 subfamily B member 1
Malignant neoplasm of breast	NME1	P15531	NME/NM23 nucleoside diphosphate kinase 1
Malignant neoplasm of breast	CD46	P15529	CD46 molecule
Malignant neoplasm of breast	HTN3	P15516	histatin 3
Malignant neoplasm of breast	AREG	P15514	amphiregulin
Malignant neoplasm of breast	ELN	P15502	elastin
Malignant neoplasm of breast	VAV1	P15498	vav guanine nucleotide exchange factor 1
Malignant neoplasm of breast	HPGD	P15428	15-hydroxyprostaglandin dehydrogenase
Malignant neoplasm of breast	GYPE	P15421	glycophorin E (MNS blood group)
Malignant neoplasm of breast	FOSL2	P15408	FOS like 2, AP-1 transcription factor subunit
Malignant neoplasm of breast	FOSL1	P15407	FOS like 1, AP-1 transcription factor subunit
Malignant neoplasm of breast	UCHL3	P15374	ubiquitin C-terminal hydrolase L3
Malignant neoplasm of breast	ATF2	P15336	activating transcription factor 2
Malignant neoplasm of breast	FOLR1	P15328	folate receptor alpha

Malignant neoplasm of breast	EZR	P15311	ezrin
Malignant neoplasm of breast	ACP3	P15309	acid phosphatase 3
Malignant neoplasm of breast	B4GALT1	P15291	beta-1,4-galactosyltransferase 1
Malignant neoplasm of breast	ARSA	P15289	arylsulfatase A
Malignant neoplasm of breast	IFNGR1	P15260	interferon gamma receptor 1
Malignant neoplasm of breast	IL9	P15248	interleukin 9
Malignant neoplasm of breast	MYOD1	P15172	myogenic differentiation 1
Malignant neoplasm of breast	GSPT1	P15170	G1 to S phase transition 1
Malignant neoplasm of breast	RAC2	P15153	Rac family small GTPase 2
Malignant neoplasm of breast	PVR	P15151	PVR cell adhesion molecule
Malignant neoplasm of breast	ANPEP	P15144	alanyl aminopeptidase, membrane
Malignant neoplasm of breast	AKR1B1	P15121	aldo-keto reductase family 1 member B
Malignant neoplasm of breast	GLUL	P15104	glutamate-ammonia ligase
Malignant neoplasm of breast	FABP4	P15090	fatty acid binding protein 4
Malignant neoplasm of breast	CPB1	P15086	carboxypeptidase B1
Malignant neoplasm of breast	CPA1	P15085	carboxypeptidase A1
Malignant neoplasm of breast	BRAF	P15056	B-Raf proto-oncogene, serine/threonine kinase
Malignant neoplasm of breast	ETS2	P15036	ETS proto-oncogene 2, transcription factor
Malignant neoplasm of breast	LIF	P15018	LIF interleukin 6 family cytokine

Malignant neoplasm of breast	JUP	P14923	junction plakoglobin
Malignant neoplasm of breast	ETS1	P14921	ETS proto-oncogene 1, transcription factor
Malignant neoplasm of breast	IDO1	P14902	indoleamine 2,3-dioxygenase 1
Malignant neoplasm of breast	HNRNPL	P14866	heterogeneous nuclear ribonucleoprotein L
Malignant neoplasm of breast	POU2F1	P14859	POU class 2 homeobox 1
Malignant neoplasm of breast	IL2RB	P14784	interleukin 2 receptor subunit beta
Malignant neoplasm of breast	MMP9	P14780	matrix metallopeptidase 9
Malignant neoplasm of breast	IL1R1	P14778	interleukin 1 receptor type 1
Malignant neoplasm of breast	TYR	P14679	tyrosinase
Malignant neoplasm of breast	SLC2A4	P14672	solute carrier family 2 member 4
Malignant neoplasm of breast	HOXB1	P14653	homeobox B1
Malignant neoplasm of breast	HOXB2	P14652	homeobox B2
Malignant neoplasm of breast	HOXB3	P14651	homeobox B3
Malignant neoplasm of breast	CCNB1	P14635	cyclin B1
Malignant neoplasm of breast	HSP90B1	P14625	heat shock protein 90 beta family member 1
Malignant neoplasm of breast	ACYP2	P14621	acylphosphatase 2
Malignant neoplasm of breast	PKM	P14618	pyruvate kinase M1/2
Malignant neoplasm of breast	INSRR	P14616	insulin receptor related receptor
Malignant neoplasm of breast	PLA2G2A	P14555	phospholipase A2 group IIA

Malignant neoplasm of breast	AKR1A1	P14550	aldo-keto reductase family 1 member A1
Malignant neoplasm of breast	NID1	P14543	nidogen 1
Malignant neoplasm of breast	DRD2	P14416	dopamine receptor D2
Malignant neoplasm of breast	ATP1B2	P14415	ATPase Na+/K+ transporting subunit beta 2
Malignant neoplasm of breast	CPM	P14384	carboxypeptidase M
Malignant neoplasm of breast	HCLS1	P14317	hematopoietic cell-specific Lyn substrate 1
Malignant neoplasm of breast	PRKCSH	P14314	protein kinase C substrate 80K-H
Malignant neoplasm of breast	HGF	P14210	hepatocyte growth factor
Malignant neoplasm of breast	CD99	P14209	CD99 molecule (Xg blood group)
Malignant neoplasm of breast	FOLR2	P14207	folate receptor beta
Malignant neoplasm of breast	MIF	P14174	macrophage migration inhibitory factor
Malignant neoplasm of breast	EDN3	P14138	endothelin 3
Malignant neoplasm of breast	GFAP	P14136	glial fibrillary acidic protein
Malignant neoplasm of breast	HSD17B1	P14061	hydroxysteroid 17-beta dehydrogenase 1
Malignant neoplasm of breast	HSD3B1	P14060	hydroxy-delta-5-steroid dehydrogenase, 3 beta- and steroid delta-isomerase 1
Malignant neoplasm of breast	MTHFD2	P13995	methylenetetrahydrofolate dehydrogenase (NADP+ dependent) 2, methenyltetrahydrofolate cyclohydrolase
Malignant neoplasm of breast	CD59	P13987	CD59 molecule (CD59 blood group)
Malignant neoplasm of breast	ADRB3	P13945	adrenoceptor beta 3
Malignant neoplasm of breast	COL11A2	P13942	collagen type XI alpha 2 chain

Malignant neoplasm of breast	SLC5A1	P13866	solute carrier family 5 member 1
Malignant neoplasm of breast	TNNT1	P13805	troponin T1, slow skeletal type
Malignant neoplasm of breast	ETFA	P13804	electron transfer flavoprotein subunit alpha
Malignant neoplasm of breast	PLS3	P13797	plastin 3
Malignant neoplasm of breast	LCP1	P13796	lymphocyte cytosolic protein 1
Malignant neoplasm of breast	HLA-DOB	P13765	major histocompatibility complex, class II, DO beta
Malignant neoplasm of breast	HLA-E	P13747	major histocompatibility complex, class I, E
Malignant neoplasm of breast	PRG2	P13727	proteoglycan 2, pro eosinophil major basic protein
Malignant neoplasm of breast	F3	P13726	coagulation factor III, tissue factor
Malignant neoplasm of breast	OSM	P13725	oncostatin M
Malignant neoplasm of breast	ALAD	P13716	aminolevulinate dehydratase
Malignant neoplasm of breast	TPT1	P13693	tumor protein, translationally-controlled 1
Malignant neoplasm of breast	CEACAM1	P13688	CEA cell adhesion molecule 1
Malignant neoplasm of breast	ACP5	P13686	acid phosphatase 5, tartrate resistant
Malignant neoplasm of breast	ZNF35	P13682	zinc finger protein 35
Malignant neoplasm of breast	P4HA1	P13674	prolyl 4-hydroxylase subunit alpha 1
Malignant neoplasm of breast	KRT5	P13647	keratin 5
Malignant neoplasm of breast	KRT13	P13646	keratin 13
Malignant neoplasm of breast	MT1G	P13640	metallothionein 1G

Malignant neoplasm of breast	EEF2	P13639	eukaryotic translation elongation factor 2
Malignant neoplasm of breast	RARG	P13631	retinoic acid receptor gamma
Malignant neoplasm of breast	ITGA4	P13612	integrin subunit alpha 4
Malignant neoplasm of breast	VCAN	P13611	versican
Malignant neoplasm of breast	NCAM1	P13591	neural cell adhesion molecule 1
Malignant neoplasm of breast	CYP4B1	P13584	cytochrome P450 family 4 subfamily B member 1
Malignant neoplasm of breast	CFTR	P13569	CF transmembrane conductance regulator
Malignant neoplasm of breast	MYH6	P13533	myosin heavy chain 6
Malignant neoplasm of breast	CCL5	P13501	C-C motif chemokine ligand 5
Malignant neoplasm of breast	CCL2	P13500	C-C motif chemokine ligand 2
Malignant neoplasm of breast	CYBA	P13498	cytochrome b-245 alpha chain
Malignant neoplasm of breast	BMP1	P13497	bone morphogenetic protein 1
Malignant neoplasm of breast	RNH1	P13489	ribonuclease/angiogenin inhibitor 1
Malignant neoplasm of breast	LAMP2	P13473	lysosomal associated membrane protein 2
Malignant neoplasm of breast	TDGF1	P13385	teratocarcinoma-derived growth factor 1
Malignant neoplasm of breast	IFI30	P13284	IFI30 lysosomal thiol reductase
Malignant neoplasm of breast	CCL4	P13236;Q8NHW4	C-C motif chemokine ligand 4
Malignant neoplasm of breast	CCL4L1	P13236;Q8NHW4	C-C motif chemokine ligand 4 like 1
Malignant neoplasm of breast	CCL4L2	P13236;Q8NHW4	C-C motif chemokine ligand 4 like 2

Malignant neoplasm of breast	IL7	P13232	interleukin 7
Malignant neoplasm of breast	IFITM1	P13164	interferon induced transmembrane protein 1
Malignant neoplasm of breast	COX4I1	P13073	cytochrome c oxidase subunit 4I1
Malignant neoplasm of breast	XRCC5	P13010	X-ray repair cross complementing 5
Malignant neoplasm of breast	XRCC6	P12956	X-ray repair cross complementing 6
Malignant neoplasm of breast	SRC	P12931	SRC proto-oncogene, non-receptor tyrosine kinase
Malignant neoplasm of breast	MYH1	P12882	myosin heavy chain 1
Malignant neoplasm of breast	MLN	P12872	motilin
Malignant neoplasm of breast	CDH1	P12830	cadherin 1
Malignant neoplasm of breast	MYL4	P12829	myosin light chain 4
Malignant neoplasm of breast	ACE	P12821	angiotensin I converting enzyme
Malignant neoplasm of breast	ACTN1	P12814	actinin alpha 1
Malignant neoplasm of breast	SKI	P12755	SKI proto-oncogene
Malignant neoplasm of breast	RNASE3	P12724	ribonuclease A family member 3
Malignant neoplasm of breast	BMP4	P12644	bone morphogenetic protein 4
Malignant neoplasm of breast	BMP2	P12643	bone morphogenetic protein 2
Malignant neoplasm of breast	MYCL	P12524	MYCL proto-oncogene, bHLH transcription factor
Malignant neoplasm of breast	ANXA3	P12429	annexin A3
Malignant neoplasm of breast	FCER1A	P12319	Fc fragment of IgE receptor Ia

Malignant neoplasm of breast	FCGR2A	P12318	Fc fragment of IgG receptor IIa
Malignant neoplasm of breast	CKB	P12277	creatine kinase B
Malignant neoplasm of breast	PIP	P12273	prolactin induced protein
Malignant neoplasm of breast	PTHLH	P12272	parathyroid hormone like hormone
Malignant neoplasm of breast	IMPDH2	P12268	inosine monophosphate dehydrogenase 2
Malignant neoplasm of breast	F5	P12259	coagulation factor V
Malignant neoplasm of breast	SLC25A6	P12236	solute carrier family 25 member 6
Malignant neoplasm of breast	SLC25A4	P12235	solute carrier family 25 member 4
Malignant neoplasm of breast	COL11A1	P12107	collagen type XI alpha 1 chain
Malignant neoplasm of breast	HARS1	P12081	histidyl-tRNA synthetase 1
Malignant neoplasm of breast	NEFH	P12036	neurofilament heavy
Malignant neoplasm of breast	FGF5	P12034	fibroblast growth factor 5
Malignant neoplasm of breast	PCNA	P12004	proliferating cell nuclear antigen
Malignant neoplasm of breast	PABPC1	P11940	poly(A) binding protein cytoplasmic 1
Malignant neoplasm of breast	ODC1	P11926	ornithine decarboxylase 1
Malignant neoplasm of breast	MS4A1	P11836	membrane spanning 4-domains A1
Malignant neoplasm of breast	SRF	P11831	serum response factor
Malignant neoplasm of breast	CDK4	P11802	cyclin dependent kinase 4
Malignant neoplasm of breast	ADH5	P11766	alcohol dehydrogenase 5 (class III), chi polypeptide

Malignant neoplasm of breast	IGF2R	P11717	insulin like growth factor 2 receptor
Malignant neoplasm of breast	CYP2C9	P11712	cytochrome P450 family 2 subfamily C member 9
Malignant neoplasm of breast	SFTPC	P11686	surfactant protein C
Malignant neoplasm of breast	SCGB1A1	P11684	secretoglobin family 1A member 1
Malignant neoplasm of breast	EPX	P11678	eosinophil peroxidase
Malignant neoplasm of breast	CETP	P11597	cholesteryl ester transfer protein
Malignant neoplasm of breast	MTHFD1	P11586	methylenetetrahydrofolate dehydrogenase, cyclohydrolase and formyltetrahydrofolate synthetase 1
Malignant neoplasm of breast	DMD	P11532	dystrophin
Malignant neoplasm of breast	CYP19A1	P11511	cytochrome P450 family 19 subfamily A member 1
Malignant neoplasm of breast	CYP2A6	P11509	cytochrome P450 family 2 subfamily A member 6
Malignant neoplasm of breast	PC	P11498	pyruvate carboxylase
Malignant neoplasm of breast	FGF3	P11487	fibroblast growth factor 3
Malignant neoplasm of breast	ESRRA	P11474	estrogen related receptor alpha
Malignant neoplasm of breast	VDR	P11473	vitamin D receptor
Malignant neoplasm of breast	PSG2	P11465	pregnancy specific beta-1-glycoprotein 2
Malignant neoplasm of breast	G6PD	P11413	glucose-6-phosphate dehydrogenase
Malignant neoplasm of breast	TOP2A	P11388	DNA topoisomerase II alpha
Malignant neoplasm of breast	TOP1	P11387	DNA topoisomerase I
Malignant neoplasm of breast	FGFR1	P11362	fibroblast growth factor receptor 1

Malignant neoplasm of breast	ACADM	P11310	acyl-CoA dehydrogenase medium chain
Malignant neoplasm of breast	PIM1	P11309	Pim-1 proto-oncogene, serine/threonine kinase
Malignant neoplasm of breast	ERG	P11308	ETS transcription factor ERG
Malignant neoplasm of breast	LAMP1	P11279	lysosomal associated membrane protein 1
Malignant neoplasm of breast	BCR	P11274	BCR activator of RhoGEF and GTPase
Malignant neoplasm of breast	NAT2	P11245	N-acetyltransferase 2
Malignant neoplasm of breast	MBL2	P11226	mannose binding lectin 2
Malignant neoplasm of breast	PYGM	P11217	glycogen phosphorylase, muscle associated
Malignant neoplasm of breast	PYGB	P11216	glycogen phosphorylase B
Malignant neoplasm of breast	ITGAM	P11215	integrin subunit alpha M
Malignant neoplasm of breast	DBT	P11182	dihydrolipoamide branched chain transacylase E2
Malignant neoplasm of breast	PDHB	P11177	pyruvate dehydrogenase E1 subunit beta
Malignant neoplasm of breast	UMPS	P11172	uridine monophosphate synthetase
Malignant neoplasm of breast	EPB41	P11171	erythrocyte membrane protein band 4.1
Malignant neoplasm of breast	SLC2A3	P11169	solute carrier family 2 member 3
Malignant neoplasm of breast	SLC2A2	P11168	solute carrier family 2 member 2
Malignant neoplasm of breast	SLC2A1	P11166	solute carrier family 2 member 1
Malignant neoplasm of breast	HSPA8	P11142	heat shock protein family A (Hsp70) member 8
Malignant neoplasm of breast	MAP2	P11137	microtubule associated protein 2

Malignant neoplasm of breast	PNMT	P11086	phenylethanolamine N-methyltransferase
Malignant neoplasm of breast	HSPA5	P11021	heat shock protein family A (Hsp70) member 5
Malignant neoplasm of breast	IAPP	P10997	islet amyloid polypeptide
Malignant neoplasm of breast	IRF1	P10914	interferon regulatory factor 1
Malignant neoplasm of breast	GHR	P10912	growth hormone receptor
Malignant neoplasm of breast	CLU	P10909	clusterin
Malignant neoplasm of breast	THRB	P10828	thyroid hormone receptor beta
Malignant neoplasm of breast	THRA	P10827	thyroid hormone receptor alpha
Malignant neoplasm of breast	RARB	P10826	retinoic acid receptor beta
Malignant neoplasm of breast	HSPD1	P10809	heat shock protein family D (Hsp60) member 1
Malignant neoplasm of breast	CD28	P10747	CD28 molecule
Malignant neoplasm of breast	KIT	P10721	KIT proto-oncogene, receptor tyrosine kinase
Malignant neoplasm of breast	TFPI	P10646	tissue factor pathway inhibitor
Malignant neoplasm of breast	CHGA	P10645	chromogranin A
Malignant neoplasm of breast	PRKAR1A	P10644	protein kinase cAMP-dependent type I regulatory subunit alpha
Malignant neoplasm of breast	MAPT	P10636	microtubule associated protein tau
Malignant neoplasm of breast	CYP2D6	P10635	cytochrome P450 family 2 subfamily D member 6
Malignant neoplasm of breast	CYP2C8	P10632	cytochrome P450 family 2 subfamily C member 8
Malignant neoplasm of breast	COX5B	P10606	cytochrome c oxidase subunit 5B

Malignant neoplasm of breast	TGFB3	P10600	transforming growth factor beta 3
Malignant neoplasm of breast	TXN	P10599	thioredoxin
Malignant neoplasm of breast	NR2F1	P10589	nuclear receptor subfamily 2 group F member 1
Malignant neoplasm of breast	NR2F6	P10588	nuclear receptor subfamily 2 group F member 6
Malignant neoplasm of breast	PTPRF	P10586	protein tyrosine phosphatase receptor type F
Malignant neoplasm of breast	DLAT	P10515	dihydrolipoamide S-acetyltransferase
Malignant neoplasm of breast	SPP1	P10451	secreted phosphoprotein 1
Malignant neoplasm of breast	BCL2	P10415	BCL2 apoptosis regulator
Malignant neoplasm of breast	ARAF	P10398	A-Raf proto-oncogene, serine/threonine kinase
Malignant neoplasm of breast	ACR	P10323	acrosin
Malignant neoplasm of breast	HLA-C	P10321	major histocompatibility complex, class I, C
Malignant neoplasm of breast	RRAS	P10301	RAS related
Malignant neoplasm of breast	RARA	P10276	retinoic acid receptor alpha
Malignant neoplasm of breast	AR	P10275	androgen receptor
Malignant neoplasm of breast	MYBL2	P10244	MYB proto-oncogene like 2
Malignant neoplasm of breast	MYB	P10242	MYB proto-oncogene, transcription factor
Malignant neoplasm of breast	COX8A	P10176	cytochrome c oxidase subunit 8A
Malignant neoplasm of breast	PRB4	P10163	proline rich protein BstNI subfamily 4
Malignant neoplasm of breast	RO60	P10155	Ro60, Y RNA binding protein

Malignant neoplasm of breast	CCL3	P10147	C-C motif chemokine ligand 3
Malignant neoplasm of breast	CXCL8	P10145	C-X-C motif chemokine ligand 8
Malignant neoplasm of breast	GZMB	P10144	granzyme B
Malignant neoplasm of breast	SRGN	P10124	serglycin
Malignant neoplasm of breast	RAP2A	P10114	RAP2A, member of RAS oncogene family
Malignant neoplasm of breast	CALCB	P10092	calcitonin related polypeptide beta
Malignant neoplasm of breast	PYY	P10082	peptide YY
Malignant neoplasm of breast	GLI3	P10071	GLI family zinc finger 3
Malignant neoplasm of breast	GLI2	P10070	GLI family zinc finger 2
Malignant neoplasm of breast	TRA	P0DSE1	T cell receptor alpha locus
Malignant neoplasm of breast	NPY4R2	P0DQD5;P50391	neuropeptide Y receptor Y4-2
Malignant neoplasm of breast	NPY4R	P0DQD5;P50391	neuropeptide Y receptor Y4
Malignant neoplasm of breast	ERCC6	P0DP91;Q03468	ERCC excision repair 6, chromatin remodeling factor
Malignant neoplasm of breast	CALM1	P0DP23;P0DP24;P0DP25	calmodulin 1
Malignant neoplasm of breast	CALM2	P0DP23;P0DP24;P0DP25	calmodulin 2
Malignant neoplasm of breast	CALM3	P0DP23;P0DP24;P0DP25	calmodulin 3
Malignant neoplasm of breast	CGB3	P0DN86	chorionic gonadotropin subunit beta 3
Malignant neoplasm of breast	CGB5	P0DN86	chorionic gonadotropin subunit beta 5
Malignant neoplasm of breast	CGB8	P0DN86	chorionic gonadotropin subunit beta 8

Malignant neoplasm of breast	CBS	P0DN79;P35520	cystathionine beta-synthase
Malignant neoplasm of breast	SMIM10L2A	P0DMW4;P0DMW5	small integral membrane protein 10 like 2A
Malignant neoplasm of breast	SMIM10L2B	P0DMW4;P0DMW5	small integral membrane protein 10 like 2B
Malignant neoplasm of breast	HSPA1B	P0DMV8;P0DMV9	heat shock protein family A (Hsp70) member 1B
Malignant neoplasm of breast	HSPA1A	P0DMV8;P0DMV9	heat shock protein family A (Hsp70) member 1A
Malignant neoplasm of breast	ATXN8OS	P0DMR3	ATXN8 opposite strand lncRNA
Malignant neoplasm of breast	CSH1	P0DML2;P0DML3	chorionic somatomammotropin hormone 1
Malignant neoplasm of breast	CSH2	P0DML2;P0DML3	chorionic somatomammotropin hormone 2
Malignant neoplasm of breast	RAB34	P0DI83;Q9BZG1	RAB34, member RAS oncogene family
Malignant neoplasm of breast	TSPY10	P0CV98;P0CW01;Q01534	testis specific protein Y-linked 10
Malignant neoplasm of breast	TSPY1	P0CV98;P0CW01;Q01534	testis specific protein Y-linked 1
Malignant neoplasm of breast	TSPY3	P0CV98;P0CW01;Q01534	testis specific protein Y-linked 3
Malignant neoplasm of breast	UBC	P0CG48	ubiquitin C
Malignant neoplasm of breast	JMJD7	P0C870	jumonji domain containing 7
Malignant neoplasm of breast	DANCR	P0C864	differentiation antagonizing non-protein coding RNA
Malignant neoplasm of breast	ASAH2B	P0C7U1	N-acylsphingosine amidohydrolase 2B
Malignant neoplasm of breast	RBMY1D	P0C7P1;P0DJD3	RNA binding motif protein Y-linked family 1 member D
Malignant neoplasm of breast	RBMY1A1	P0C7P1;P0DJD3	RNA binding motif protein Y-linked family 1 member A1
Malignant neoplasm of breast	ZGLP1	P0C6A0	zinc finger GATA like protein 1

Malignant neoplasm of breast	CT62	P0C5K7	cancer/testis associated 62
Malignant neoplasm of breast	H2AZ1	P0C0S5	H2A.Z variant histone 1
Malignant neoplasm of breast	NPS	P0C0P6	neuropeptide S
Malignant neoplasm of breast	NUDT17	P0C025	nudix hydrolase 17
Malignant neoplasm of breast	UCHL1	P09936	ubiquitin C-terminal hydrolase L1
Malignant neoplasm of breast	ALPI	P09923	alkaline phosphatase, intestinal
Malignant neoplasm of breast	CSF3	P09919	colony stimulating factor 3
Malignant neoplasm of breast	ALOX5	P09917	arachidonate 5-lipoxygenase
Malignant neoplasm of breast	IFI6	P09912	interferon alpha inducible protein 6
Malignant neoplasm of breast	PARP1	P09874	poly(ADP-ribose) polymerase 1
Malignant neoplasm of breast	FGR	P09769	FGR proto-oncogene, Src family tyrosine kinase
Malignant neoplasm of breast	TACSTD2	P09758	tumor associated calcium signal transducer 2
Malignant neoplasm of breast	GIP	P09681	gastric inhibitory polypeptide
Malignant neoplasm of breast	COX6C	P09669	cytochrome c oxidase subunit 6C
Malignant neoplasm of breast	HNRNPA1	P09651	heterogeneous nuclear ribonucleoprotein A1
Malignant neoplasm of breast	HOXC6	P09630	homeobox C6
Malignant neoplasm of breast	HOXB7	P09629	homeobox B7
Malignant neoplasm of breast	DLD	P09622	dihydrolipoamide dehydrogenase
Malignant neoplasm of breast	PDGFRB	P09619	platelet derived growth factor receptor beta

Malignant neoplasm of breast	CSF1	P09603	colony stimulating factor 1
Malignant neoplasm of breast	HMOX1	P09601	heme oxygenase 1
Malignant neoplasm of breast	WNT2	P09544	Wnt family member 2
Malignant neoplasm of breast	CNP	P09543	2',3'-cyclic nucleotide 3' phosphodiesterase
Malignant neoplasm of breast	INHBB	P09529	inhibin subunit beta B
Malignant neoplasm of breast	ANXA4	P09525	annexin A4
Malignant neoplasm of breast	CLTA	P09496	clathrin light chain A
Malignant neoplasm of breast	TPM1	P09493	tropomyosin 1
Malignant neoplasm of breast	GSTM1	P09488	glutathione S-transferase mu 1
Malignant neoplasm of breast	SPARC	P09486	secreted protein acidic and cysteine rich
Malignant neoplasm of breast	FBP1	P09467	fructose-bisphosphatase 1
Malignant neoplasm of breast	PAEP	P09466	progestagen associated endometrial protein
Malignant neoplasm of breast	RBP1	P09455	retinol binding protein 1
Malignant neoplasm of breast	TNP1	P09430	transition protein 1
Malignant neoplasm of breast	HMGB1	P09429	high mobility group box 1
Malignant neoplasm of breast	LGALS1	P09382	galectin 1
Malignant neoplasm of breast	CXCL1	P09341	C-X-C motif chemokine ligand 1
Malignant neoplasm of breast	MMP10	P09238	matrix metallopeptidase 10
Malignant neoplasm of breast	MMP7	P09237	matrix metallopeptidase 7

Malignant neoplasm of breast	GSTP1	P09211	glutathione S-transferase pi 1
Malignant neoplasm of breast	GSTA2	P09210	glutathione S-transferase alpha 2
Malignant neoplasm of breast	ENO2	P09104	enolase 2
Malignant neoplasm of breast	HOXB5	P09067	homeobox B5
Malignant neoplasm of breast	FGF2	P09038	fibroblast growth factor 2
Malignant neoplasm of breast	CD63	P08962	CD63 molecule
Malignant neoplasm of breast	ROS1	P08922	ROS proto-oncogene 1, receptor tyrosine kinase
Malignant neoplasm of breast	ADRA2A	P08913	adrenoceptor alpha 2A
Malignant neoplasm of breast	IL6R	P08887	interleukin 6 receptor
Malignant neoplasm of breast	RPSA	P08865	ribosomal protein SA
Malignant neoplasm of breast	STS	P08842	steroid sulfatase
Malignant neoplasm of breast	IGFBP1	P08833	insulin like growth factor binding protein 1
Malignant neoplasm of breast	ANXA5	P08758	annexin A5
Malignant neoplasm of breast	KRT7	P08729	keratin 7
Malignant neoplasm of breast	KRT19	P08727	keratin 19
Malignant neoplasm of breast	F7	P08709	coagulation factor VII
Malignant neoplasm of breast	IL3	P08700	interleukin 3
Malignant neoplasm of breast	CYP3A4	P08684	cytochrome P450 family 3 subfamily A member 4
Malignant neoplasm of breast	VIM	P08670	vimentin

Malignant neoplasm of breast	NFIC	P08651	nuclear factor I C
Malignant neoplasm of breast	ITGA5	P08648	integrin subunit alpha 5
Malignant neoplasm of breast	FCGR3A	P08637	Fc fragment of IgG receptor IIIa
Malignant neoplasm of breast	FGF4	P08620	fibroblast growth factor 4
Malignant neoplasm of breast	CFH	P08603	complement factor H
Malignant neoplasm of breast	ADRB1	P08588	adrenoceptor beta 1
Malignant neoplasm of breast	MET	P08581	MET proto-oncogene, receptor tyrosine kinase
Malignant neoplasm of breast	PTPRC	P08575	protein tyrosine phosphatase receptor type C
Malignant neoplasm of breast	CYC1	P08574	cytochrome c1
Malignant neoplasm of breast	COL4A2	P08572	collagen type IV alpha 2 chain
Malignant neoplasm of breast	CD14	P08571	CD14 molecule
Malignant neoplasm of breast	PLEK	P08567	pleckstrin
Malignant neoplasm of breast	LPA	P08519	lipoprotein(a)
Malignant neoplasm of breast	ITGA2B	P08514	integrin subunit alpha 2b
Malignant neoplasm of breast	MGP	P08493	matrix Gla protein
Malignant neoplasm of breast	MME	P08473	membrane metalloendopeptidase
Malignant neoplasm of breast	HMBS	P08397	hydroxymethylbilane synthase
Malignant neoplasm of breast	CTSG	P08311	cathepsin G
Malignant neoplasm of breast	SOD3	P08294	superoxide dismutase 3

Malignant neoplasm of breast	GSTA1	P08263	glutathione S-transferase alpha 1
Malignant neoplasm of breast	MMP3	P08254	matrix metallopeptidase 3
Malignant neoplasm of breast	MMP2	P08253	matrix metallopeptidase 2
Malignant neoplasm of breast	SYP	P08247	synaptophysin
Malignant neoplasm of breast	ELANE	P08246	elastase, neutrophil expressed
Malignant neoplasm of breast	ASNS	P08243	asparagine synthetase (glutamine-hydrolyzing)
Malignant neoplasm of breast	HSP90AB1	P08238	heat shock protein 90 alpha family class B member 1
Malignant neoplasm of breast	PFKM	P08237	phosphofructokinase, muscle
Malignant neoplasm of breast	GUSB	P08236	glucuronidase beta
Malignant neoplasm of breast	SLC3A2	P08195	solute carrier family 3 member 2
Malignant neoplasm of breast	ABCB1	P08183	ATP binding cassette subfamily B member 1
Malignant neoplasm of breast	CD55	P08174	CD55 molecule (Cromer blood group)
Malignant neoplasm of breast	GLI1	P08151	GLI family zinc finger 1
Malignant neoplasm of breast	NGFR	P08138	nerve growth factor receptor
Malignant neoplasm of breast	RHOC	P08134	ras homolog family member C
Malignant neoplasm of breast	ANXA6	P08133	annexin A6
Malignant neoplasm of breast	MSMB	P08118	microseminoprotein beta
Malignant neoplasm of breast	IGF1R	P08069	insulin like growth factor 1 receptor
Malignant neoplasm of breast	SP1	P08047	Sp1 transcription factor

Malignant neoplasm of breast	THBS1	P07996	thrombospondin 1
Malignant neoplasm of breast	ERCC1	P07992	ERCC excision repair 1, endonuclease non-catalytic subunit
Malignant neoplasm of breast	FH	P07954	fumarate hydratase
Malignant neoplasm of breast	TPM2	P07951	tropomyosin 2
Malignant neoplasm of breast	RET	P07949	ret proto-oncogene
Malignant neoplasm of breast	LYN	P07948	LYN proto-oncogene, Src family tyrosine kinase
Malignant neoplasm of breast	YES1	P07947	YES proto-oncogene 1, Src family tyrosine kinase
Malignant neoplasm of breast	UMOD	P07911	uromodulin
Malignant neoplasm of breast	HNRNPC	P07910	heterogeneous nuclear ribonucleoprotein C
Malignant neoplasm of breast	HSP90AA1	P07900	heat shock protein 90 alpha family class A member 1
Malignant neoplasm of breast	LDHC	P07864	lactate dehydrogenase C
Malignant neoplasm of breast	CTSB	P07858	cathepsin B
Malignant neoplasm of breast	EPRS1	P07814	glutamyl-prolyl-tRNA synthetase 1
Malignant neoplasm of breast	CD3E	P07766	CD3e molecule
Malignant neoplasm of breast	APRT	P07741	adenine phosphoribosyltransferase
Malignant neoplasm of breast	PFN1	P07737	profilin 1
Malignant neoplasm of breast	CTSL	P07711	cathepsin L
Malignant neoplasm of breast	PSAP	P07602	prosaposin
Malignant neoplasm of breast	DCN	P07585	decorin

Malignant neoplasm of breast	ADRB2	P07550	adrenoceptor beta 2
Malignant neoplasm of breast	GRP	P07492	gastrin releasing peptide
Malignant neoplasm of breast	MT1B	P07438	metallothionein 1B
Malignant neoplasm of breast	CAPN1	P07384	calpain 1
Malignant neoplasm of breast	ANXA2	P07355	annexin A2
Malignant neoplasm of breast	CTSD	P07339	cathepsin D
Malignant neoplasm of breast	CSF1R	P07333	colony stimulating factor 1 receptor
Malignant neoplasm of breast	FES	P07332	FES proto-oncogene, tyrosine kinase
Malignant neoplasm of breast	H1-0	P07305	H1.0 linker histone
Malignant neoplasm of breast	KLK3	P07288	kallikrein related peptidase 3
Malignant neoplasm of breast	P4HB	P07237	prolyl 4-hydroxylase subunit beta
Malignant neoplasm of breast	PROS1	P07225	protein S
Malignant neoplasm of breast	THBD	P07204	thrombomodulin
Malignant neoplasm of breast	GPX1	P07203	glutathione peroxidase 1
Malignant neoplasm of breast	TPO	P07202	thyroid peroxidase
Malignant neoplasm of breast	NEFM	P07197	neurofilament medium
Malignant neoplasm of breast	NEFL	P07196	neurofilament light
Malignant neoplasm of breast	LDHB	P07195	lactate dehydrogenase B
Malignant neoplasm of breast	EPHX1	P07099	epoxide hydrolase 1

Malignant neoplasm of breast	SERPINE2	P07093	serpin family E member 2
Malignant neoplasm of breast	KLK1	P06870	kallikrein 1
Malignant neoplasm of breast	LPL	P06858	lipoprotein lipase
Malignant neoplasm of breast	CRH	P06850	corticotropin releasing hormone
Malignant neoplasm of breast	ITGAV	P06756	integrin subunit alpha V
Malignant neoplasm of breast	TPM3	P06753	tropomyosin 3
Malignant neoplasm of breast	NPM1	P06748	nucleophosmin 1
Malignant neoplasm of breast	POLB	P06746	DNA polymerase beta
Malignant neoplasm of breast	GPI	P06744	glucose-6-phosphate isomerase
Malignant neoplasm of breast	ENO1	P06733	enolase 1
Malignant neoplasm of breast	CEACAM5	P06731	CEA cell adhesion molecule 5
Malignant neoplasm of breast	EIF4E	P06730	eukaryotic translation initiation factor 4E
Malignant neoplasm of breast	CD2	P06729	CD2 molecule
Malignant neoplasm of breast	APOA4	P06727	apolipoprotein A4
Malignant neoplasm of breast	S100A6	P06703	S100 calcium binding protein A6
Malignant neoplasm of breast	S100A9	P06702	S100 calcium binding protein A9
Malignant neoplasm of breast	CDK1	P06493	cyclin dependent kinase 1
Malignant neoplasm of breast	PTMA	P06454	prothymosin alpha
Malignant neoplasm of breast	PGR	P06401	progesterone receptor

Malignant neoplasm of breast	RB1	P06400	RB transcriptional corepressor 1
Malignant neoplasm of breast	GSN	P06396	gelsolin
Malignant neoplasm of breast	HLA-DOA	P06340	major histocompatibility complex, class II, DO alpha
Malignant neoplasm of breast	CCK	P06307	cholecystokinin
Malignant neoplasm of breast	GLA	P06280	galactosidase alpha
Malignant neoplasm of breast	BCHE	P06276	butyrylcholinesterase
Malignant neoplasm of breast	FYN	P06241	FYN proto-oncogene, Src family tyrosine kinase
Malignant neoplasm of breast	INSR	P06213	insulin receptor
Malignant neoplasm of breast	UGT2B4	P06133	UDP glucuronosyltransferase family 2 member B4
Malignant neoplasm of breast	CD1A	P06126	CD1a molecule
Malignant neoplasm of breast	GYPB	P06028	glycophorin B (MNS blood group)
Malignant neoplasm of breast	HPN	P05981	hepsin
Malignant neoplasm of breast	KRT8	P05787	keratin 8
Malignant neoplasm of breast	KRT18	P05783	keratin 18
Malignant neoplasm of breast	PRKCB	P05771	protein kinase C beta
Malignant neoplasm of breast	ITGB1	P05556	integrin subunit beta 1
Malignant neoplasm of breast	TFAP2A	P05549	transcription factor AP-2 alpha
Malignant neoplasm of breast	REG1A	P05451	regenerating family member 1 alpha
Malignant neoplasm of breast	FABP3	P05413	fatty acid binding protein 3

Malignant neoplasm of breast	JUN	P05412	Jun proto-oncogene, AP-1 transcription factor subunit
Malignant neoplasm of breast	RPLP2	P05387	ribosomal protein lateral stalk subunit P2
Malignant neoplasm of breast	ICAM1	P05362	intercellular adhesion molecule 1
Malignant neoplasm of breast	EDN1	P05305	endothelin 1
Malignant neoplasm of breast	IL6	P05231	interleukin 6
Malignant neoplasm of breast	FGF1	P05230	fibroblast growth factor 1
Malignant neoplasm of breast	HMGN2	P05204	high mobility group nucleosomal binding domain 2
Malignant neoplasm of breast	EIF2S1	P05198	eukaryotic translation initiation factor 2 subunit alpha
Malignant neoplasm of breast	ALPP	P05187	alkaline phosphatase, placental
Malignant neoplasm of breast	ALPL	P05186	alkaline phosphatase, biomineralization associated
Malignant neoplasm of breast	CYP2E1	P05181	cytochrome P450 family 2 subfamily E member 1
Malignant neoplasm of breast	CYP1A2	P05177	cytochrome P450 family 1 subfamily A member 2
Malignant neoplasm of breast	MPO	P05164	myeloperoxidase
Malignant neoplasm of breast	LGALS2	P05162	galectin 2
Malignant neoplasm of breast	ISG15	P05161	ISG15 ubiquitin like modifier
Malignant neoplasm of breast	CFI	P05156	complement factor I
Malignant neoplasm of breast	SERPINA5	P05154	serpin family A member 5
Malignant neoplasm of breast	SLC25A5	P05141	solute carrier family 25 member 5
Malignant neoplasm of breast	SERPINE1	P05121	serpin family E member 1

Malignant neoplasm of breast	SERPINB2	P05120	serpin family B member 2
Malignant neoplasm of breast	IL5	P05113	interleukin 5
Malignant neoplasm of breast	IL4	P05112	interleukin 4
Malignant neoplasm of breast	S100A8	P05109	S100 calcium binding protein A8
Malignant neoplasm of breast	CYP11A1	P05108	cytochrome P450 family 11 subfamily A member 1
Malignant neoplasm of breast	ITGB2	P05107	integrin subunit beta 2
Malignant neoplasm of breast	ITGB3	P05106	integrin subunit beta 3
Malignant neoplasm of breast	CYP17A1	P05093	cytochrome P450 family 17 subfamily A member 1
Malignant neoplasm of breast	ALDH2	P05091	aldehyde dehydrogenase 2 family member
Malignant neoplasm of breast	APOD	P05090	apolipoprotein D
Malignant neoplasm of breast	APP	P05067	amyloid beta precursor protein
Malignant neoplasm of breast	CHGB	P05060	chromogranin B
Malignant neoplasm of breast	ATP1B1	P05026	ATPase Na+/K+ transporting subunit beta 1
Malignant neoplasm of breast	IGF1	P05019	insulin like growth factor 1
Malignant neoplasm of breast	IFNW1	P05000	interferon omega 1
Malignant neoplasm of breast	GNAI2	P04899	G protein subunit alpha i2
Malignant neoplasm of breast	RPN2	P04844	ribophorin II
Malignant neoplasm of breast	TYMS	P04818	thymidylate synthetase
Malignant neoplasm of breast	CYP1A1	P04798	cytochrome P450 family 1 subfamily A member 1

Malignant neoplasm of breast	HSPB1	P04792	heat shock protein family B (small) member 1
Malignant neoplasm of breast	MT1F	P04733	metallothionein 1F
Malignant neoplasm of breast	MT1E	P04732	metallothionein 1E
Malignant neoplasm of breast	MT1A	P04731	metallothionein 1A
Malignant neoplasm of breast	TP53	P04637	tumor protein p53
Malignant neoplasm of breast	CAPNS1	P04632	calpain small subunit 1
Malignant neoplasm of breast	NTRK1	P04629	neurotrophic receptor tyrosine kinase 1
Malignant neoplasm of breast	WNT1	P04628	Wnt family member 1
Malignant neoplasm of breast	ERBB2	P04626	erb-b2 receptor tyrosine kinase 2
Malignant neoplasm of breast	PRM1	P04553	protamine 1
Malignant neoplasm of breast	HLA-A	P04439	major histocompatibility complex, class I, A
Malignant neoplasm of breast	ASL	P04424	argininosuccinate lyase
Malignant neoplasm of breast	GAPDH	P04406	glyceraldehyde-3-phosphate dehydrogenase
Malignant neoplasm of breast	SHBG	P04278	sex hormone binding globulin
Malignant neoplasm of breast	VWF	P04275	von Willebrand factor
Malignant neoplasm of breast	S100B	P04271	S100 calcium binding protein B
Malignant neoplasm of breast	KRT1	P04264	keratin 1
Malignant neoplasm of breast	CD3D	P04234	CD3d molecule
Malignant neoplasm of breast	CD74	P04233	CD74 molecule

Malignant neoplasm of breast	THY1	P04216	Thy-1 cell surface antigen
Malignant neoplasm of breast	MAS1	P04201	MAS1 proto-oncogene, G protein-coupled receptor
Malignant neoplasm of breast	HRG	P04196	histidine rich glycoprotein
Malignant neoplasm of breast	TK1	P04183	thymidine kinase 1
Malignant neoplasm of breast	SOD2	P04179	superoxide dismutase 2
Malignant neoplasm of breast	TFF1	P04155	trefoil factor 1
Malignant neoplasm of breast	NR3C1	P04150	nuclear receptor subfamily 3 group C member 1
Malignant neoplasm of breast	CSF2	P04141	colony stimulating factor 2
Malignant neoplasm of breast	APOB	P04114	apolipoprotein B
Malignant neoplasm of breast	PDGFA	P04085	platelet derived growth factor subunit A
Malignant neoplasm of breast	ANXA1	P04083	annexin A1
Malignant neoplasm of breast	ALDOA	P04075	aldolase, fructose-bisphosphate A
Malignant neoplasm of breast	PROC	P04070	protein C, inactivator of coagulation factors Va and VIIIa
Malignant neoplasm of breast	FUCA1	P04066	alpha-L-fucosidase 1
Malignant neoplasm of breast	PLA2G1B	P04054	phospholipase A2 group IB
Malignant neoplasm of breast	RAF1	P04049	Raf-1 proto-oncogene, serine/threonine kinase
Malignant neoplasm of breast	CAT	P04040	catalase
Malignant neoplasm of breast	HMGCR	P04035	3-hydroxy-3-methylglutaryl-CoA reductase
Malignant neoplasm of breast	VTN	P04004	vitronectin

Malignant neoplasm of breast	C4BPA	P04003	complement component 4 binding protein alpha
Malignant neoplasm of breast	OPN1LW	P04000	opsin 1, long wave sensitive
Malignant neoplasm of breast	SLPI	P03973	secretory leukocyte peptidase inhibitor
Malignant neoplasm of breast	AMH	P03971	anti-Mullerian hormone
Malignant neoplasm of breast	MMP1	P03956	matrix metallopeptidase 1
Malignant neoplasm of breast	F11	P03951	coagulation factor XI
Malignant neoplasm of breast	ANG	P03950	angiogenin
Malignant neoplasm of breast	ATP8	P03928	ATP synthase F0 subunit 8
Malignant neoplasm of breast	ND5	P03915	NADH dehydrogenase, subunit 5 (complex I)
Malignant neoplasm of breast	ND4	P03905	NADH dehydrogenase, subunit 4 (complex I)
Malignant neoplasm of breast	ND3	P03897	NADH dehydrogenase, subunit 3 (complex I)
Malignant neoplasm of breast	ND2	P03891	MTND2
Malignant neoplasm of breast	ESR1	P03372	estrogen receptor 1
Malignant neoplasm of breast	BGLAP	P02818	bone gamma-carboxyglutamate protein
Malignant neoplasm of breast	SMR3B	P02814	submaxillary gland androgen regulated protein 3B
Malignant neoplasm of breast	PRB2	P02812	proline rich protein BstNI subfamily 2
Malignant neoplasm of breast	MT2A	P02795	metallothionein 2A
Malignant neoplasm of breast	FTH1	P02794	ferritin heavy chain 1
Malignant neoplasm of breast	FTL	P02792	ferritin light chain

Malignant neoplasm of breast	HPX	P02790	hemopexin
Malignant neoplasm of breast	LTF	P02788	lactotransferrin
Malignant neoplasm of breast	TF	P02787	transferrin
Malignant neoplasm of breast	TFRC	P02786	transferrin receptor
Malignant neoplasm of breast	CXCL10	P02778	C-X-C motif chemokine ligand 10
Malignant neoplasm of breast	PF4	P02776	platelet factor 4
Malignant neoplasm of breast	PPBP	P02775	pro-platelet basic protein
Malignant neoplasm of breast	GC	P02774	GC vitamin D binding protein
Malignant neoplasm of breast	AFP	P02771	alpha fetoprotein
Malignant neoplasm of breast	ALB	P02768	albumin
Malignant neoplasm of breast	TTR	P02766	transthyretin
Malignant neoplasm of breast	AHSG	P02765	alpha 2-HS glycoprotein
Malignant neoplasm of breast	AMBP	P02760	alpha-1-microglobulin/bikunin precursor
Malignant neoplasm of breast	FN1	P02751	fibronectin 1
Malignant neoplasm of breast	LRG1	P02750	leucine rich alpha-2-glycoprotein 1
Malignant neoplasm of breast	C1QB	P02746	complement C1q B chain
Malignant neoplasm of breast	C1QA	P02745	complement C1q A chain
Malignant neoplasm of breast	APCS	P02743	amyloid P component, serum
Malignant neoplasm of breast	CRP	P02741	C-reactive protein

Malignant neoplasm of breast	SLC4A1	P02730	solute carrier family 4 member 1 (Diego blood group)
Malignant neoplasm of breast	GYPA	P02724	glycophorin A (MNS blood group)
Malignant neoplasm of breast	FGA	P02671	fibrinogen alpha chain
Malignant neoplasm of breast	APOC3	P02656	apolipoprotein C3
Malignant neoplasm of breast	APOE	P02649	apolipoprotein E
Malignant neoplasm of breast	APOA1	P02647	apolipoprotein A1
Malignant neoplasm of breast	LMNA	P02545	lamin A/C
Malignant neoplasm of breast	KRT6A	P02538	keratin 6A
Malignant neoplasm of breast	KRT14	P02533	keratin 14
Malignant neoplasm of breast	CRYAB	P02511	crystallin alpha B
Malignant neoplasm of breast	COL4A1	P02462	collagen type IV alpha 1 chain
Malignant neoplasm of breast	COL1A1	P02452	collagen type I alpha 1 chain
Malignant neoplasm of breast	MB	P02144	myoglobin
Malignant neoplasm of breast	HLA-DQB1	P01920	major histocompatibility complex, class II, DQ beta 1
Malignant neoplasm of breast	HLA-DRB1	P01911	major histocompatibility complex, class II, DR beta 1
Malignant neoplasm of breast	HLA-DQA1	P01909	major histocompatibility complex, class II, DQ alpha 1
Malignant neoplasm of breast	HLA-DRA	P01903	major histocompatibility complex, class II, DR alpha
Malignant neoplasm of breast	HLA-B	P01889	major histocompatibility complex, class I, B
Malignant neoplasm of breast	IGHG3	P01860	immunoglobulin heavy constant gamma 3 (G3m marker)

Malignant neoplasm of breast	IGKC	P01834	immunoglobulin kappa constant
Malignant neoplasm of breast	CD8A	P01732	CD8a molecule
Malignant neoplasm of breast	IL2RA	P01589	interleukin 2 receptor subunit alpha
Malignant neoplasm of breast	EPO	P01588	erythropoietin
Malignant neoplasm of breast	IL1B	P01584	interleukin 1 beta
Malignant neoplasm of breast	IL1A	P01583	interleukin 1 alpha
Malignant neoplasm of breast	IFNG	P01579	interferon gamma
Malignant neoplasm of breast	IFNB1	P01574	interferon beta 1
Malignant neoplasm of breast	IFNA2	P01563	interferon alpha 2
Malignant neoplasm of breast	IFNA1	P01562	interferon alpha 1
Malignant neoplasm of breast	IFNA13	P01562	interferon alpha 13
Malignant neoplasm of breast	TNF	P01375	tumor necrosis factor
Malignant neoplasm of breast	LTA	P01374	lymphotoxin alpha
Malignant neoplasm of breast	GAST	P01350	gastrin
Malignant neoplasm of breast	IGF2	P01344	insulin like growth factor 2
Malignant neoplasm of breast	INS	P01308	insulin
Malignant neoplasm of breast	NPY	P01303	neuropeptide Y
Malignant neoplasm of breast	GHRH	P01286	growth hormone releasing hormone
Malignant neoplasm of breast	VIP	P01282	vasoactive intestinal peptide

Malignant neoplasm of breast	GCG	P01275	glucagon
Malignant neoplasm of breast	PTH	P01270	parathyroid hormone
Malignant neoplasm of breast	CALCA	P01258;P06881	calcitonin related polypeptide alpha
Malignant neoplasm of breast	GH1	P01241	growth hormone 1
Malignant neoplasm of breast	PRL	P01236	prolactin
Malignant neoplasm of breast	LHB	P01229	luteinizing hormone subunit beta
Malignant neoplasm of breast	CGA	P01215	glycoprotein hormones, alpha polypeptide
Malignant neoplasm of breast	PENK	P01210	proenkephalin
Malignant neoplasm of breast	POMC	P01189	proopiomelanocortin
Malignant neoplasm of breast	AVP	P01185	arginine vasopressin
Malignant neoplasm of breast	OXT	P01178	oxytocin/neurophysin I prepropeptide
Malignant neoplasm of breast	NPPA	P01160	natriuretic peptide A
Malignant neoplasm of breast	GNRH1	P01148	gonadotropin releasing hormone 1
Malignant neoplasm of breast	NGF	P01138	nerve growth factor
Malignant neoplasm of breast	TGFB1	P01137	transforming growth factor beta 1
Malignant neoplasm of breast	TGFA	P01135	transforming growth factor alpha
Malignant neoplasm of breast	EGF	P01133	epidermal growth factor
Malignant neoplasm of breast	LDLR	P01130	low density lipoprotein receptor
Malignant neoplasm of breast	PDGFB	P01127	platelet derived growth factor subunit B

Malignant neoplasm of breast	KRAS	P01116	KRAS proto-oncogene, GTPase
Malignant neoplasm of breast	HRAS	P01112	HRas proto-oncogene, GTPase
Malignant neoplasm of breast	NRAS	P01111	NRAS proto-oncogene, GTPase
Malignant neoplasm of breast	MYC	P01106	MYC proto-oncogene, bHLH transcription factor
Malignant neoplasm of breast	FOS	P01100	Fos proto-oncogene, AP-1 transcription factor subunit
Malignant neoplasm of breast	KNG1	P01042	kininogen 1
Malignant neoplasm of breast	CSTA	P01040	cystatin A
Malignant neoplasm of breast	CST1	P01037	cystatin SN
Malignant neoplasm of breast	CST4	P01036	cystatin S
Malignant neoplasm of breast	CST3	P01034	cystatin C
Malignant neoplasm of breast	TIMP1	P01033	TIMP metallopeptidase inhibitor 1
Malignant neoplasm of breast	C5	P01031	complement C5
Malignant neoplasm of breast	C3	P01024	complement C3
Malignant neoplasm of breast	AGT	P01019	angiotensinogen
Malignant neoplasm of breast	SERPINA3	P01011	serpin family A member 3
Malignant neoplasm of breast	SERPINA1	P01009	serpin family A member 1
Malignant neoplasm of breast	SPINK1	P00995	serine peptidase inhibitor Kazal type 1
Malignant neoplasm of breast	ASS1	P00966	argininosuccinate synthase 1
Malignant neoplasm of breast	CA2	P00918	carbonic anhydrase 2

Malignant neoplasm of breast	CA1	P00915	carbonic anhydrase 1
Malignant neoplasm of breast	ATP6	P00846	ATP synthase F0 subunit 6
Malignant neoplasm of breast	ADA	P00813	adenosine deaminase
Malignant neoplasm of breast	REN	P00797	renin
Malignant neoplasm of breast	CFB	P00751	complement factor B
Malignant neoplasm of breast	PLAT	P00750	plasminogen activator, tissue type
Malignant neoplasm of breast	PLAU	P00749	plasminogen activator, urokinase
Malignant neoplasm of breast	PLG	P00747	plasminogen
Malignant neoplasm of breast	F10	P00742	coagulation factor X
Malignant neoplasm of breast	F9	P00740	coagulation factor IX
Malignant neoplasm of breast	HPR	P00739	haptoglobin-related protein
Malignant neoplasm of breast	HP	P00738	haptoglobin
Malignant neoplasm of breast	F2	P00734	coagulation factor II, thrombin
Malignant neoplasm of breast	LALBA	P00709	lactalbumin alpha
Malignant neoplasm of breast	PGK1	P00558	phosphoglycerate kinase 1
Malignant neoplasm of breast	MOS	P00540	MOS proto-oncogene, serine/threonine kinase
Malignant neoplasm of breast	EGFR	P00533	epidermal growth factor receptor
Malignant neoplasm of breast	ABL1	P00519	ABL proto-oncogene 1, non-receptor tyrosine kinase
Malignant neoplasm of breast	GOT2	P00505	glutamic-oxaloacetic transaminase 2

Malignant neoplasm of breast	HPRT1	P00492	hypoxanthine phosphoribosyltransferase 1
Malignant neoplasm of breast	PNP	P00491	purine nucleoside phosphorylase
Malignant neoplasm of breast	F8	P00451	coagulation factor VIII
Malignant neoplasm of breast	CP	P00450	ceruloplasmin
Malignant neoplasm of breast	SOD1	P00441	superoxide dismutase 1
Malignant neoplasm of breast	PAH	P00439	phenylalanine hydroxylase
Malignant neoplasm of breast	COX3	P00414	cytochrome c oxidase III
Malignant neoplasm of breast	COX2	P00403	cytochrome c oxidase subunit II
Malignant neoplasm of breast	COX1	P00395	cytochrome c oxidase subunit I
Malignant neoplasm of breast	GSR	P00390	glutathione-disulfide reductase
Malignant neoplasm of breast	CYB5R3	P00387	cytochrome b5 reductase 3
Malignant neoplasm of breast	DHFR	P00374	dihydrofolate reductase
Malignant neoplasm of breast	GLUD1	P00367	glutamate dehydrogenase 1
Malignant neoplasm of breast	ALDH1A1	P00352	aldehyde dehydrogenase 1 family member A1
Malignant neoplasm of breast	LDHA	P00338	lactate dehydrogenase A
Malignant neoplasm of breast	ADH1C	P00326	alcohol dehydrogenase 1C (class I), gamma polypeptide
Malignant neoplasm of breast	ADH1B	P00325	alcohol dehydrogenase 1B (class I), beta polypeptide
Malignant neoplasm of breast	CYB5A	P00167	cytochrome b5 type A
Malignant neoplasm of breast	CYTB	P00156	cytochrome b

Malignant neoplasm of breast	NSD2	O96028	nuclear receptor binding SET domain protein 2
Malignant neoplasm of breast	B3GALT4	O96024	beta-1,3-galactosyltransferase 4
Malignant neoplasm of breast	CCNE2	O96020	cyclin E2
Malignant neoplasm of breast	APBA3	O96018	amyloid beta precursor protein binding family A member 3
Malignant neoplasm of breast	CHEK2	O96017	checkpoint kinase 2
Malignant neoplasm of breast	WNT11	O96014	Wnt family member 11
Malignant neoplasm of breast	PAK4	O96013	p21 (RAC1) activated kinase 4
Malignant neoplasm of breast	BCL10	O95999	BCL10 immune signaling adaptor
Malignant neoplasm of breast	IL18BP	O95998	interleukin 18 binding protein
Malignant neoplasm of breast	PTTG1	O95997	PTTG1 regulator of sister chromatid separation, securin
Malignant neoplasm of breast	APC2	O95996	APC regulator of WNT signaling pathway 2
Malignant neoplasm of breast	GAS8	O95995	growth arrest specific 8
Malignant neoplasm of breast	AGR2	O95994	anterior gradient 2, protein disulphide isomerase family member
Malignant neoplasm of breast	TCL1B	O95988	T cell leukemia/lymphoma 1B
Malignant neoplasm of breast	RECK	O95980	reversion inducing cysteine rich protein with kazal motifs
Malignant neoplasm of breast	S1PR4	O95977	sphingosine-1-phosphate receptor 4
Malignant neoplasm of breast	CD160	O95971	CD160 molecule
Malignant neoplasm of breast	SCGB1D2	O95969	secretoglobin family 1D member 2
Malignant neoplasm of breast	EFEMP2	O95967	EGF containing fibulin extracellular matrix protein 2

Malignant neoplasm of breast	ITGBL1	O95965	integrin subunit beta like 1
Malignant neoplasm of breast	NCR2	O95944	natural cytotoxicity triggering receptor 2
Malignant neoplasm of breast	EOMES	O95936	eomesodermin
Malignant neoplasm of breast	CBX7	O95931	chromobox 7
Malignant neoplasm of breast	SYF2	O95926	SYF2 pre-mRNA splicing factor
Malignant neoplasm of breast	ECD	O95905	ecdysoneless cell cycle regulator
Malignant neoplasm of breast	FADS2	O95864	fatty acid desaturase 2
Malignant neoplasm of breast	SNAI1	O95863	snail family transcriptional repressor 1
Malignant neoplasm of breast	TSPAN12	O95859	tetraspanin 12
Malignant neoplasm of breast	TSPAN13	O95857	tetraspanin 13
Malignant neoplasm of breast	ANGPTL1	O95841	angiopoietin like 1
Malignant neoplasm of breast	LATS1	O95835	large tumor suppressor kinase 1
Malignant neoplasm of breast	CLIC3	O95833	chloride intracellular channel 3
Malignant neoplasm of breast	CLDN1	O95832	claudin 1
Malignant neoplasm of breast	AIFM1	O95831	apoptosis inducing factor mitochondria associated 1
Malignant neoplasm of breast	MAP4K4	O95819	mitogen-activated protein kinase kinase kinase kinase 4
Malignant neoplasm of breast	BAG3	O95817	BAG cochaperone 3
Malignant neoplasm of breast	CAVIN2	O95810	caveolae associated protein 2
Malignant neoplasm of breast	TTC4	O95801	tetratricopeptide repeat domain 4

Malignant neoplasm of breast	DDX58	O95786	DExD/H-box helicase 58
Malignant neoplasm of breast	AP2A1	O95782	adaptor related protein complex 2 subunit alpha 1
Malignant neoplasm of breast	IL33	O95760	interleukin 33
Malignant neoplasm of breast	PTBP3	O95758	polypyrimidine tract binding protein 3
Malignant neoplasm of breast	SEMA4F	O95754	ssemaphorin 4F
Malignant neoplasm of breast	FGF19	O95750	fibroblast growth factor 19
Malignant neoplasm of breast	ESRRB	O95718	estrogen related receptor beta
Malignant neoplasm of breast	RAB3D	O95716	RAB3D, member RAS oncogene family
Malignant neoplasm of breast	CXCL14	O95715	C-X-C motif chemokine ligand 14
Malignant neoplasm of breast	HERC2	O95714	HECT and RLD domain containing E3 ubiquitin protein ligase 2
Malignant neoplasm of breast	APBB3	O95704	amyloid beta precursor protein binding family B member 3
Malignant neoplasm of breast	BRD1	O95696	bromodomain containing 1
Malignant neoplasm of breast	DIRAS3	O95661	DIRAS family GTPase 3
Malignant neoplasm of breast	NFATC1	O95644	nuclear factor of activated T cells 1
Malignant neoplasm of breast	CPSF4	O95639	cleavage and polyadenylation specific factor 4
Malignant neoplasm of breast	FSTL3	O95633	follistatin like 3
Malignant neoplasm of breast	NTN1	O95631	netrin 1
Malignant neoplasm of breast	PCNT	O95613	pericentrin
Malignant neoplasm of breast	KLF8	O95600	Kruppel like factor 8

Malignant neoplasm of breast	SLC2A10	O95528	solute carrier family 2 member 10
Malignant neoplasm of breast	CBX6	O95503	chromobox 6
Malignant neoplasm of breast	ADGRL2	O95490	adhesion G protein-coupled receptor L2
Malignant neoplasm of breast	H6PD	O95479	hexose-6-phosphate dehydrogenase/glucose 1-dehydrogenase
Malignant neoplasm of breast	ABCA1	O95477	ATP binding cassette subfamily A member 1
Malignant neoplasm of breast	SIX6	O95475	SIX homeobox 6
Malignant neoplasm of breast	CLDN7	O95471	claudin 7
Malignant neoplasm of breast	SGPL1	O95470	sphingosine-1-phosphate lyase 1
Malignant neoplasm of breast	GNAS	O95467;P63092;P84996;Q5JWF2	GNAS complex locus
Malignant neoplasm of breast	PSMG1	O95456	proteasome assembly chaperone 1
Malignant neoplasm of breast	PARN	O95453	poly(A)-specific ribonuclease
Malignant neoplasm of breast	GJB6	O95452	gap junction protein beta 6
Malignant neoplasm of breast	ADAMTS2	O95450	ADAM metallopeptidase with thrombospondin type 1 motif 2
Malignant neoplasm of breast	SLC34A2	O95436	solute carrier family 34 member 2
Malignant neoplasm of breast	AHSA1	O95433	activator of HSP90 ATPase activity 1
Malignant neoplasm of breast	BRI3	O95415	brain protein I3
Malignant neoplasm of breast	TNFRSF6B	O95407	TNF receptor superfamily member 6b
Malignant neoplasm of breast	ZFYVE9	O95405	zinc finger FYVE-type containing 9
Malignant neoplasm of breast	UTS2	O95399	urotensin 2

Malignant neoplasm of breast	RAPGEF3	O95398	Rap guanine nucleotide exchange factor 3
Malignant neoplasm of breast	PGM3	O95394	phosphoglucomutase 3
Malignant neoplasm of breast	BMP10	O95393	bone morphogenetic protein 10
Malignant neoplasm of breast	GDF11	O95390	growth differentiation factor 11
Malignant neoplasm of breast	CCN6	O95389	cellular communication network factor 6
Malignant neoplasm of breast	CCN4	O95388	cellular communication network factor 4
Malignant neoplasm of breast	MAP3K6	O95382	mitogen-activated protein kinase kinase kinase 6
Malignant neoplasm of breast	TNFAIP8	O95379	TNF alpha induced protein 8
Malignant neoplasm of breast	ZBTB7A	O95365	zinc finger and BTB domain containing 7A
Malignant neoplasm of breast	TRIM16	O95361	tripartite motif containing 16
Malignant neoplasm of breast	TACC2	O95359	transforming acidic coiled-coil containing protein 2
Malignant neoplasm of breast	ATG7	O95352	autophagy related 7
Malignant neoplasm of breast	SIX3	O95343	SIX homeobox 3
Malignant neoplasm of breast	ABCB11	O95342	ATP binding cassette subfamily B member 11
Malignant neoplasm of breast	PGLS	O95336	6-phosphogluconolactonase
Malignant neoplasm of breast	CELF2	O95319	CUGBP Elav-like family member 2
Malignant neoplasm of breast	RASAL1	O95294	RAS protein activator like 1
Malignant neoplasm of breast	KCNK5	O95279	potassium two pore domain channel subfamily K member 5
Malignant neoplasm of breast	TNKS	O95271	tankyrase

Malignant neoplasm of breast	RASGRP1	O95267	RAS guanyl releasing protein 1
Malignant neoplasm of breast	KCNH1	O95259	potassium voltage-gated channel subfamily H member 1
Malignant neoplasm of breast	GADD45G	O95257	growth arrest and DNA damage inducible gamma
Malignant neoplasm of breast	KAT7	O95251	lysine acetyltransferase 7
Malignant neoplasm of breast	GOSR1	O95249	golgi SNAP receptor complex member 1
Malignant neoplasm of breast	MBD4	O95243	methyl-CpG binding domain 4, DNA glycosylase
Malignant neoplasm of breast	KIF4A	O95239	kinesin family member 4A
Malignant neoplasm of breast	SPDEF	O95238	SAM pointed domain containing ETS transcription factor
Malignant neoplasm of breast	KIF20A	O95235	kinesin family member 20A
Malignant neoplasm of breast	ZWINT	O95229	ZW10 interacting kinetochore protein
Malignant neoplasm of breast	PCDH8	O95206	protocadherin 8
Malignant neoplasm of breast	UNC5C	O95185	unc-5 netrin receptor C
Malignant neoplasm of breast	CACNA1H	O95180	calcium voltage-gated channel subunit alpha1 H
Malignant neoplasm of breast	GAS8-AS1	O95177	GAS8 antisense RNA 1
Malignant neoplasm of breast	NDUFA3	O95167	NADH:ubiquinone oxidoreductase subunit A3
Malignant neoplasm of breast	GABARAP	O95166	GABA type A receptor-associated protein
Malignant neoplasm of breast	UBL3	O95164	ubiquitin like 3
Malignant neoplasm of breast	ELP1	O95163	elongator complex protein 1
Malignant neoplasm of breast	TNFSF15	O95150	TNF superfamily member 15

Malignant neoplasm of breast	MFN2	O95140	mitofusin 2
Malignant neoplasm of breast	S1PR2	O95136	sphingosine-1-phosphate receptor 2
Malignant neoplasm of breast	PRSS23	O95084	serine protease 23
Malignant neoplasm of breast	ALX3	O95076	ALX homeobox 3
Malignant neoplasm of breast	RAD54B	O95073;Q9Y620	RAD54 homolog B
Malignant neoplasm of breast	FSBP	O95073;Q9Y620	fibrinogen silencer binding protein
Malignant neoplasm of breast	UBR5	O95071	ubiquitin protein ligase E3 component n-recognin 5
Malignant neoplasm of breast	CCNB2	O95067	cyclin B2
Malignant neoplasm of breast	RPP14	O95059	ribonuclease P/MRP subunit p14
Malignant neoplasm of breast	HEXIM1	O94992	HEXIM P-TEFb complex subunit 1
Malignant neoplasm of breast	FAM13A	O94988	family with sequence similarity 13 member A
Malignant neoplasm of breast	AP2A2	O94973	adaptor related protein complex 2 subunit alpha 2
Malignant neoplasm of breast	TRIM37	O94972	tripartite motif containing 37
Malignant neoplasm of breast	SLCO2B1	O94956	solute carrier organic anion transporter family member 2B1
Malignant neoplasm of breast	KDM4B	O94953	lysine demethylase 4B
Malignant neoplasm of breast	GLS	O94925	glutaminase
Malignant neoplasm of breast	GLCE	O94923	glucuronic acid epimerase
Malignant neoplasm of breast	CDK14	O94921	cyclin dependent kinase 14
Malignant neoplasm of breast	NFAT5	O94916	nuclear factor of activated T cells 5

Malignant neoplasm of breast	DKK1	O94907	dickkopf WNT signaling pathway inhibitor 1
Malignant neoplasm of breast	ERLIN2	O94905	ER lipid raft associated 2
Malignant neoplasm of breast	SUN1	O94901	Sad1 and UNC84 domain containing 1
Malignant neoplasm of breast	TOX	O94900	thymocyte selection associated high mobility group box
Malignant neoplasm of breast	ZNF432	O94892	zinc finger protein 432
Malignant neoplasm of breast	SASH1	O94885	SAM and SH3 domain containing 1
Malignant neoplasm of breast	MICAL2	O94851	microtubule associated monooxygenase, calponin and LIM domain containing 2
Malignant neoplasm of breast	RHOBTB1	O94844	Rho related BTB domain containing 1
Malignant neoplasm of breast	TOX4	O94842	TOX high mobility group box family member 4
Malignant neoplasm of breast	MYO1D	O94832	myosin ID
Malignant neoplasm of breast	DDHD2	O94830	DDHD domain containing 2
Malignant neoplasm of breast	IPO13	O94829	importin 13
Malignant neoplasm of breast	KBTBD11	O94819	kelch repeat and BTB domain containing 11
Malignant neoplasm of breast	ATG12	O94817	autophagy related 12
Malignant neoplasm of breast	SLIT2	O94813	slit guidance ligand 2
Malignant neoplasm of breast	RGS11	O94810	regulator of G protein signaling 11
Malignant neoplasm of breast	PRKD3	O94806	protein kinase D3
Malignant neoplasm of breast	ALDH1A2	O94788	aldehyde dehydrogenase 1 family member A2
Malignant neoplasm of breast	USP1	O94782	ubiquitin specific peptidase 1

Malignant neoplasm of breast	AQP8	O94778	aquaporin 8
Malignant neoplasm of breast	MTA2	O94776	metastasis associated 1 family member 2
Malignant neoplasm of breast	RECQL5	O94762	RecQ like helicase 5
Malignant neoplasm of breast	RECQL4	O94761	RecQ like helicase 4
Malignant neoplasm of breast	DDAH1	O94760	dimethylarginine dimethylaminohydrolase 1
Malignant neoplasm of breast	TRPM2	O94759	transient receptor potential cation channel subfamily M member 2
Malignant neoplasm of breast	JTB	O76095	jumping translocation breakpoint
Malignant neoplasm of breast	FGF18	O76093	fibroblast growth factor 18
Malignant neoplasm of breast	BEST1	O76090	bestrophin 1
Malignant neoplasm of breast	SLC22A5	O76082	solute carrier family 22 member 5
Malignant neoplasm of breast	RGS20	O76081	regulator of G protein signaling 20
Malignant neoplasm of breast	CCN5	O76076	cellular communication network factor 5
Malignant neoplasm of breast	DFFB	O76075	DNA fragmentation factor subunit beta
Malignant neoplasm of breast	PDE5A	O76074	phosphodiesterase 5A
Malignant neoplasm of breast	SNCG	O76070	synuclein gamma
Malignant neoplasm of breast	RNF8	O76064	ring finger protein 8
Malignant neoplasm of breast	TM7SF2	O76062	transmembrane 7 superfamily member 2
Malignant neoplasm of breast	STC2	O76061	stanniocalcin 2
Malignant neoplasm of breast	SEC14L2	O76054	SEC14 like lipid binding 2

Malignant neoplasm of breast	NEURL1	O76050	neuralized E3 ubiquitin protein ligase 1
Malignant neoplasm of breast	GLRX3	O76003	glutaredoxin 3
Malignant neoplasm of breast	CPD	O75976	carboxypeptidase D
Malignant neoplasm of breast	C1QL1	O75973	complement C1q like 1
Malignant neoplasm of breast	AKAP3	O75969	A-kinase anchoring protein 3
Malignant neoplasm of breast	CDK2AP2	O75956	cyclin dependent kinase 2 associated protein 2
Malignant neoplasm of breast	FLOT1	O75955	flotillin 1
Malignant neoplasm of breast	RAD17	O75943	RAD17 checkpoint clamp loader component
Malignant neoplasm of breast	BCAS2	O75934	BCAS2 pre-mRNA processing factor
Malignant neoplasm of breast	PIAS1	O75925	protein inhibitor of activated STAT 1
Malignant neoplasm of breast	DYSF	O75923	dysferlin
Malignant neoplasm of breast	ARL6IP5	O75915	ADP ribosylation factor like GTPase 6 interacting protein 5
Malignant neoplasm of breast	RASSF9	O75901	Ras association domain family member 9
Malignant neoplasm of breast	MMP23B	O75900	matrix metallopeptidase 23B
Malignant neoplasm of breast	TUSC2	O75896	tumor suppressor 2, mitochondrial calcium regulator
Malignant neoplasm of breast	ALDH1L1	O75891	aldehyde dehydrogenase 1 family member L1
Malignant neoplasm of breast	TNFSF13	O75888	TNF superfamily member 13
Malignant neoplasm of breast	STAM2	O75886	signal transducing adaptor molecule 2
Malignant neoplasm of breast	CYP7B1	O75881	cytochrome P450 family 7 subfamily B member 1

Malignant neoplasm of breast	IDH1	O75874	isocitrate dehydrogenase (NADP(+)) 1
Malignant neoplasm of breast	ZMPSTE24	O75844	zinc metallopeptidase STE24
Malignant neoplasm of breast	UPK1B	O75841	uroplakin 1B
Malignant neoplasm of breast	CIB2	O75838	calcium and integrin binding family member 2
Malignant neoplasm of breast	PSMD10	O75832	proteasome 26S subunit, non-ATPase 10
Malignant neoplasm of breast	CNMD	O75829	chondromodulin
Malignant neoplasm of breast	CBR3	O75828	carbonyl reductase 3
Malignant neoplasm of breast	EIF3G	O75821	eukaryotic translation initiation factor 3 subunit G
Malignant neoplasm of breast	BCAR3	O75815	BCAR3 adaptor protein, NSP family member
Malignant neoplasm of breast	ZMYND10	O75800	zinc finger MYND-type containing 10
Malignant neoplasm of breast	UGT2B17	O75795	UDP glucuronosyltransferase family 2 member B17
Malignant neoplasm of breast	GRAP2	O75791	GRB2 related adaptor protein 2
Malignant neoplasm of breast	ATP6AP2	O75787	ATPase H+ transporting accessory protein 2
Malignant neoplasm of breast	RAD51D	O75771	RAD51 paralog D
Malignant neoplasm of breast	TCEA3	O75764	transcription elongation factor A3
Malignant neoplasm of breast	SLC22A3	O75751	solute carrier family 22 member 3
Malignant neoplasm of breast	WDHD1	O75717	WD repeat and HMG-box DNA binding protein 1
Malignant neoplasm of breast	UTP20	O75691	UTP20 small subunit processome component
Malignant neoplasm of breast	PPM1B	O75688	protein phosphatase, Mg2+/Mn2+ dependent 1B

Malignant neoplasm of breast	RPS6KA4	O75676	ribosomal protein S6 kinase A4
Malignant neoplasm of breast	REM1	O75628	RRAD and GEM like GTPase 1
Malignant neoplasm of breast	PRDM1	O75626	PR/SET domain 1
Malignant neoplasm of breast	ERAL1	O75616	Era like 12S mitochondrial rRNA chaperone 1
Malignant neoplasm of breast	USP2	O75604	ubiquitin specific peptidase 2
Malignant neoplasm of breast	GCM2	O75603	glial cells missing transcription factor 2
Malignant neoplasm of breast	SPAG6	O75602	sperm associated antigen 6
Malignant neoplasm of breast	CLEC3A	O75596	C-type lectin domain family 3 member A
Malignant neoplasm of breast	RPS6KA5	O75582	ribosomal protein S6 kinase A5
Malignant neoplasm of breast	LRP6	O75581	LDL receptor related protein 6
Malignant neoplasm of breast	CRCP	O75575	CGRP receptor component
Malignant neoplasm of breast	PRKRA	O75569	protein activator of interferon induced protein kinase EIF2AK2
Malignant neoplasm of breast	SCGB2A1	O75556	secretoglobin family 2A member 1
Malignant neoplasm of breast	WBP4	O75554	WW domain binding protein 4
Malignant neoplasm of breast	DAB1	O75553	DAB adaptor protein 1
Malignant neoplasm of breast	SF3B1	O75533	splicing factor 3b subunit 1
Malignant neoplasm of breast	EED	O75530	embryonic ectoderm development
Malignant neoplasm of breast	TADA3	O75528	transcriptional adaptor 3
Malignant neoplasm of breast	KHDRBS3	O75525	KH RNA binding domain containing, signal transduction associated 3

Malignant neoplasm of breast	GMNN	O75496	geminin DNA replication inhibitor
Malignant neoplasm of breast	CA11	O75493	carbonic anhydrase 11
Malignant neoplasm of breast	NDUFS3	O75489	NADH:ubiquinone oxidoreductase core subunit S3
Malignant neoplasm of breast	PSIP1	O75475	PC4 and SFRS1 interacting protein 1
Malignant neoplasm of breast	LGR5	O75473	leucine rich repeat containing G protein-coupled receptor 5
Malignant neoplasm of breast	NR1I2	O75469	nuclear receptor subfamily 1 group I member 2
Malignant neoplasm of breast	E2F6	O75461	E2F transcription factor 6
Malignant neoplasm of breast	ERN1	O75460	endoplasmic reticulum to nucleus signaling 1
Malignant neoplasm of breast	USH2A	O75445	usherin
Malignant neoplasm of breast	TECTA	O75443	tectorin alpha
Malignant neoplasm of breast	GIGYF1	O75420	GRB10 interacting GYF protein 1
Malignant neoplasm of breast	POLQ	O75417	DNA polymerase theta
Malignant neoplasm of breast	TACC1	O75410	transforming acidic coiled-coil containing protein 1
Malignant neoplasm of breast	ULK1	O75385	unc-51 like autophagy activating kinase 1
Malignant neoplasm of breast	TRIM3	O75382	tripartite motif containing 3
Malignant neoplasm of breast	PEX14	O75381	peroxisomal biogenesis factor 14
Malignant neoplasm of breast	NCOR1	O75376	nuclear receptor corepressor 1
Malignant neoplasm of breast	FLNB	O75369	filamin B
Malignant neoplasm of breast	MACROH2A1	O75367	macroH2A.1 histone

Malignant neoplasm of breast	PTP4A3	O75365	protein tyrosine phosphatase 4A3
Malignant neoplasm of breast	BCAS1	O75363	breast carcinoma amplified sequence 1
Malignant neoplasm of breast	ZNF217	O75362	zinc finger protein 217
Malignant neoplasm of breast	ENTPD5	O75356	ectonucleoside triphosphate diphosphohydrolase 5 (inactive)
Malignant neoplasm of breast	ENTPD3	O75355	ectonucleoside triphosphate diphosphohydrolase 3
Malignant neoplasm of breast	VPS4B	O75351	vacuolar protein sorting 4 homolog B
Malignant neoplasm of breast	PDCD6	O75340	programmed cell death 6
Malignant neoplasm of breast	HMMR	O75330	hyaluronan mediated motility receptor
Malignant neoplasm of breast	SEMA7A	O75326	semaphorin 7A (John Milton Hagen blood group)
Malignant neoplasm of breast	ZPR1	O75312	ZPR1 zinc finger
Malignant neoplasm of breast	NDUFS7	O75251	NADH:ubiquinone oxidoreductase core subunit S7
Malignant neoplasm of breast	GGCT	O75223	gamma-glutamylcyclotransferase
Malignant neoplasm of breast	LRP5	O75197	LDL receptor related protein 5
Malignant neoplasm of breast	DNAJB6	O75190	DnaJ heat shock protein family (Hsp40) member B6
Malignant neoplasm of breast	ATP2C2	O75185	ATPase secretory pathway Ca2+ transporting 2
Malignant neoplasm of breast	SIN3B	O75182	SIN3 transcription regulator family member B
Malignant neoplasm of breast	CNOT3	O75175	CCR4-NOT transcription complex subunit 3
Malignant neoplasm of breast	ADAMTS4	O75173	ADAM metallopeptidase with thrombospondin type 1 motif 4
Malignant neoplasm of breast	DNAJC13	O75165	DnaJ heat shock protein family (Hsp40) member C13

Malignant neoplasm of breast	KDM4A	O75164	lysine demethylase 4A
Malignant neoplasm of breast	SOCS5	O75159	suppressor of cytokine signaling 5
Malignant neoplasm of breast	RAB11FIP3	O75154	RAB11 family interacting protein 3
Malignant neoplasm of breast	ZC3H11A	O75152	zinc finger CCCH-type containing 11A
Malignant neoplasm of breast	PHF2	O75151	PHD finger protein 2
Malignant neoplasm of breast	CPNE3	O75131	copine 3
Malignant neoplasm of breast	ASTN2	O75129	astrotactin 2
Malignant neoplasm of breast	ROCK2	O75116	Rho associated coiled-coil containing protein kinase 2
Malignant neoplasm of breast	SLIT3	O75094	slit guidance ligand 3
Malignant neoplasm of breast	FZD7	O75084	frizzled class receptor 7
Malignant neoplasm of breast	WDR1	O75083	WD repeat domain 1
Malignant neoplasm of breast	CBFA2T3	O75081	CBFA2/RUNX1 partner transcriptional co-repressor 3
Malignant neoplasm of breast	ADAM11	O75078	ADAM metallopeptidase domain 11
Malignant neoplasm of breast	ADAM23	O75077	ADAM metallopeptidase domain 23
Malignant neoplasm of breast	FKTN	O75072	fukutin
Malignant neoplasm of breast	NOS1AP	O75052	nitric oxide synthase 1 adaptor protein
Malignant neoplasm of breast	PLXNA2	O75051	plexin A2
Malignant neoplasm of breast	SRGAP2	O75044	SLIT-ROBO Rho GTPase activating protein 2
Malignant neoplasm of breast	ABCB7	O75027	ATP binding cassette subfamily B member 7

Malignant neoplasm of breast	FCGR3B	O75015	Fc fragment of IgG receptor IIIb
Malignant neoplasm of breast	NOL3	O60936	nucleolar protein 3
Malignant neoplasm of breast	NBN	O60934	nibrin
Malignant neoplasm of breast	RNASEH1	O60930	ribonuclease H1
Malignant neoplasm of breast	HUS1	O60921	HUS1 checkpoint clamp component
Malignant neoplasm of breast	TBL1X	O60907	transducin beta like 1 X-linked
Malignant neoplasm of breast	SMPD2	O60906	sphingomyelin phosphodiesterase 2
Malignant neoplasm of breast	SHOX2	O60902	short stature homeobox 2
Malignant neoplasm of breast	BRD4	O60885	bromodomain containing 4
Malignant neoplasm of breast	SH2D1A	O60880	SH2 domain containing 1A
Malignant neoplasm of breast	KIN	O60870	Kin17 DNA and RNA binding protein
Malignant neoplasm of breast	GAS7	O60861	growth arrest specific 7
Malignant neoplasm of breast	TRIM13	O60858	tripartite motif containing 13
Malignant neoplasm of breast	EIF5B	O60841	eukaryotic translation initiation factor 5B
Malignant neoplasm of breast	DKC1	O60832	dyskerin pseudouridine synthase 1
Malignant neoplasm of breast	PFKFB2	O60825	6-phosphofructo-2-kinase/fructose-2,6-biphosphatase 2
Malignant neoplasm of breast	H2BC12	O60814	H2B clustered histone 12
Malignant neoplasm of breast	SLC19A2	O60779	solute carrier family 19 member 2
Malignant neoplasm of breast	USO1	O60763	USO1 vesicle transport factor

Malignant neoplasm of breast	HPGDS	O60760	hematopoietic prostaglandin D synthase
Malignant neoplasm of breast	HCN1	O60741	hyperpolarization activated cyclic nucleotide gated potassium channel 1
Malignant neoplasm of breast	PLA2G6	O60733	phospholipase A2 group VI
Malignant neoplasm of breast	CTNND1	O60716	catenin delta 1
Malignant neoplasm of breast	LPXN	O60711	leupaxin
Malignant neoplasm of breast	UGDH	O60701	UDP-glucose 6-dehydrogenase
Malignant neoplasm of breast	MSC	O60682	musculin
Malignant neoplasm of breast	MAFK	O60675	MAF bZIP transcription factor K
Malignant neoplasm of breast	JAK2	O60674	Janus kinase 2
Malignant neoplasm of breast	REV3L	O60673	REV3 like, DNA directed polymerase zeta catalytic subunit
Malignant neoplasm of breast	RAD1	O60671	RAD1 checkpoint DNA exonuclease
Malignant neoplasm of breast	SLC16A7	O60669	solute carrier family 16 member 7
Malignant neoplasm of breast	PDE8A	O60658	phosphodiesterase 8A
Malignant neoplasm of breast	UGT1A9	O60656	UDP glucuronosyltransferase family 1 member A9
Malignant neoplasm of breast	TSPAN1	O60635	tetraspanin 1
Malignant neoplasm of breast	SELENOF	O60613	selenoprotein F
Malignant neoplasm of breast	TLR2	O60603	toll like receptor 2
Malignant neoplasm of breast	TLR5	O60602	toll like receptor 5
Malignant neoplasm of breast	BUB1B	O60566	BUB1 mitotic checkpoint serine/threonine kinase B

Malignant neoplasm of breast	GREM1	O60565	gremlin 1, DAN family BMP antagonist
Malignant neoplasm of breast	OGA	O60502	O-GlcNAcase
Malignant neoplasm of breast	DOK2	O60496	docking protein 2
Malignant neoplasm of breast	CUBN	O60494	cubilin
Malignant neoplasm of breast	ACSL4	O60488	acyl-CoA synthetase long chain family member 4
Malignant neoplasm of breast	MPZL2	O60487	myelin protein zero like 2
Malignant neoplasm of breast	BRINP1	O60477	BMP/retinoic acid inducible neural specific 1
Malignant neoplasm of breast	NRP2	O60462	neuropilin 2
Malignant neoplasm of breast	LY75-CD302	O60449	LY75-CD302 readthrough
Malignant neoplasm of breast	LY75	O60449	lymphocyte antigen 75
Malignant neoplasm of breast	GSDME	O60443	gasdermin E
Malignant neoplasm of breast	FADS1	O60427	fatty acid desaturase 1
Malignant neoplasm of breast	GRIN3B	O60391	glutamate ionotropic receptor NMDA type subunit 3B
Malignant neoplasm of breast	HBP1	O60381	HMG-box transcription factor 1
Malignant neoplasm of breast	NUPR1	O60356	nuclear protein 1, transcriptional regulator
Malignant neoplasm of breast	FZD6	O60353	frizzled class receptor 6
Malignant neoplasm of breast	KDM1A	O60341	lysine demethylase 1A
Malignant neoplasm of breast	KIF1B	O60333	kinesin family member 1B
Malignant neoplasm of breast	PIP5K1C	O60331	phosphatidylinositol-4-phosphate 5-kinase type 1 gamma

Malignant neoplasm of breast	MCM3AP	O60318	minichromosome maintenance complex component 3 associated protein
Malignant neoplasm of breast	ZEB2	O60315	zinc finger E-box binding homeobox 2
Malignant neoplasm of breast	MGRN1	O60291	mahogunin ring finger 1
Malignant neoplasm of breast	NUAK1	O60285	NUAK family kinase 1
Malignant neoplasm of breast	ST18	O60284	ST18 C2H2C-type zinc finger transcription factor
Malignant neoplasm of breast	SPAG9	O60271	sperm associated antigen 9
Malignant neoplasm of breast	SMARCA5	O60264	SWI/SNF related, matrix associated, actin dependent regulator of chromatin, subfamily a, member 5
Malignant neoplasm of breast	PRKN	O60260	parkin RBR E3 ubiquitin protein ligase
Malignant neoplasm of breast	KLK8	O60259	kallikrein related peptidase 8
Malignant neoplasm of breast	FGF17	O60258	fibroblast growth factor 17
Malignant neoplasm of breast	PCDH7	O60245	protocadherin 7
Malignant neoplasm of breast	MED14	O60244	mediator complex subunit 14
Malignant neoplasm of breast	BNIP3L	O60238	BCL2 interacting protein 3 like
Malignant neoplasm of breast	PPP1R12B	O60237	protein phosphatase 1 regulatory subunit 12B
Malignant neoplasm of breast	TMPRSS11D	O60235	transmembrane serine protease 11D
Malignant neoplasm of breast	ZNRD2	O60232	zinc ribbon domain containing 2
Malignant neoplasm of breast	KALRN	O60229	kalirin RhoGEF kinase
Malignant neoplasm of breast	TIMM8A	O60220	translocase of inner mitochondrial membrane 8A
Malignant neoplasm of breast	AKR1B10	O60218	aldo-keto reductase family 1 member B10

Malignant neoplasm of breast	RAD21	O60216	RAD21 cohesin complex component
Malignant neoplasm of breast	ORC4	O43929	origin recognition complex subunit 4
Malignant neoplasm of breast	CXCL13	O43927	C-X-C motif chemokine ligand 13
Malignant neoplasm of breast	EFNA2	O43921	ephrin A2
Malignant neoplasm of breast	AIRE	O43918	autoimmune regulator
Malignant neoplasm of breast	VEGFD	O43915	vascular endothelial growth factor D
Malignant neoplasm of breast	EXTL3	O43909	exostosin like glycosyltransferase 3
Malignant neoplasm of breast	PRICKLE3	O43900	prickle planar cell polarity protein 3
Malignant neoplasm of breast	TLL1	O43897	tolloid like 1
Malignant neoplasm of breast	CREB3	O43889	cAMP responsive element binding protein 3
Malignant neoplasm of breast	CD5L	O43866	CD5 molecule like
Malignant neoplasm of breast	EDIL3	O43854	EGF like repeats and discoidin domains 3
Malignant neoplasm of breast	CALU	O43852	calumenin
Malignant neoplasm of breast	NRDC	O43847	nardilysin convertase
Malignant neoplasm of breast	ANGPTL7	O43827	angiopoietin like 7
Malignant neoplasm of breast	AKAP8	O43823	A-kinase anchoring protein 8
Malignant neoplasm of breast	SCO2	O43819	synthesis of cytochrome C oxidase 2
Malignant neoplasm of breast	RRP9	O43818	ribosomal RNA processing 9, U3 small nucleolar RNA binding protein
Malignant neoplasm of breast	LANCL1	O43813	LanC like 1

Malignant neoplasm of breast	SSNA1	O43805	SS nuclear autoantigen 1
Malignant neoplasm of breast	SPOP	O43791	speckle type BTB/POZ protein
Malignant neoplasm of breast	SLC25A20	O43772	solute carrier family 25 member 20
Malignant neoplasm of breast	ENSA	O43768	endosulfine alpha
Malignant neoplasm of breast	SGTA	O43765	small glutamine rich tetratricopeptide repeat containing alpha
Malignant neoplasm of breast	CHP2	O43745	calcineurin like EF-hand protein 2
Malignant neoplasm of breast	ITM2A	O43736	integral membrane protein 2A
Malignant neoplasm of breast	TRIAP1	O43715	TP53 regulated inhibitor of apoptosis 1
Malignant neoplasm of breast	GSTZ1	O43708	glutathione S-transferase zeta 1
Malignant neoplasm of breast	ACTN4	O43707	actinin alpha 4
Malignant neoplasm of breast	BUB3	O43684	BUB3 mitotic checkpoint protein
Malignant neoplasm of breast	BUB1	O43683	BUB1 mitotic checkpoint serine/threonine kinase
Malignant neoplasm of breast	TCF21	O43680	transcription factor 21
Malignant neoplasm of breast	NDUFA2	O43678	NADH:ubiquinone oxidoreductase subunit A2
Malignant neoplasm of breast	NDUFB3	O43676	NADH:ubiquinone oxidoreductase subunit B3
Malignant neoplasm of breast	PRC1	O43663	protein regulator of cytokinesis 1
Malignant neoplasm of breast	PSCA	O43653	prostate stem cell antigen
Malignant neoplasm of breast	FOXS1	O43638	forkhead box S1
Malignant neoplasm of breast	SNAI2	O43623	snail family transcriptional repressor 2

Malignant neoplasm of breast	TIMM44	O43615	translocase of inner mitochondrial membrane 44
Malignant neoplasm of breast	SPRY1	O43609	sprouty RTK signaling antagonist 1
Malignant neoplasm of breast	DCX	O43602	doublecortin
Malignant neoplasm of breast	SPRY2	O43597	sprouty RTK signaling antagonist 2
Malignant neoplasm of breast	XPOT	O43592	exportin for tRNA
Malignant neoplasm of breast	DENR	O43583	density regulated re-initiation and release factor
Malignant neoplasm of breast	AKAP10	O43572	A-kinase anchoring protein 10
Malignant neoplasm of breast	CA12	O43570	carbonic anhydrase 12
Malignant neoplasm of breast	XRCC2	O43543	X-ray repair cross complementing 2
Malignant neoplasm of breast	XRCC3	O43542	X-ray repair cross complementing 3
Malignant neoplasm of breast	SMAD6	O43541	SMAD family member 6
Malignant neoplasm of breast	FOXO3	O43524	forkhead box O3
Malignant neoplasm of breast	BCL2L11	O43521	BCL2 like 11
Malignant neoplasm of breast	ATP8B1	O43520	ATPase phospholipid transporting 8B1
Malignant neoplasm of breast	WIPF1	O43516	WAS/WASL interacting protein family member 1
Malignant neoplasm of breast	MED7	O43513	mediator complex subunit 7
Malignant neoplasm of breast	SLC26A4	O43511	solute carrier family 26 member 4
Malignant neoplasm of breast	TNFSF12-TNFSF13	O43508	TNFSF12-TNFSF13 readthrough
Malignant neoplasm of breast	TNFSF12	O43508	TNF superfamily member 12

Malignant neoplasm of breast	B4GAT1	O43505	beta-1,4-glucuronyltransferase 1
Malignant neoplasm of breast	LAMTOR5	O43504	late endosomal/lysosomal adaptor, MAPK and MTOR activator 5
Malignant neoplasm of breast	RAD51C	O43502	RAD51 paralog C
Malignant neoplasm of breast	CACNA1G	O43497	calcium voltage-gated channel subunit alpha1 G
Malignant neoplasm of breast	EPB41L2	O43491	erythrocyte membrane protein band 4.1 like 2
Malignant neoplasm of breast	PROM1	O43490	prominin 1
Malignant neoplasm of breast	OIP5	O43482	Opa interacting protein 5
Malignant neoplasm of breast	KLF4	O43474	Kruppel like factor 4
Malignant neoplasm of breast	HTRA2	O43464	HtrA serine peptidase 2
Malignant neoplasm of breast	SUV39H1	O43463	suppressor of variegation 3-9 homolog 1
Malignant neoplasm of breast	MGAM	O43451	maltase-glucoamylase
Malignant neoplasm of breast	CBFA2T2	O43439	CBFA2/RUNX1 partner transcriptional co-repressor 2
Malignant neoplasm of breast	TBX1	O43435	T-box transcription factor 1
Malignant neoplasm of breast	GRID2	O43424	glutamate ionotropic receptor delta type subunit 2
Malignant neoplasm of breast	TPD52L2	O43399	TPD52 like 2
Malignant neoplasm of breast	PRPF3	O43395	pre-mRNA processing factor 3
Malignant neoplasm of breast	HNRNPR	O43390	heterogeneous nuclear ribonucleoprotein R
Malignant neoplasm of breast	WDR62	O43379	WD repeat domain 62
Malignant neoplasm of breast	HOXA3	O43365	homeobox A3

Malignant neoplasm of breast	RIPK2	O43353	receptor interacting serine/threonine kinase 2
Malignant neoplasm of breast	MSI1	O43347	musashi RNA binding protein 1
Malignant neoplasm of breast	EEF1E1	O43324	eukaryotic translation elongation factor 1 epsilon 1
Malignant neoplasm of breast	MAP3K7	O43318	mitogen-activated protein kinase kinase kinase 7
Malignant neoplasm of breast	AQP9	O43315	aquaporin 9
Malignant neoplasm of breast	MTSS1	O43312	MTSS I-BAR domain containing 1
Malignant neoplasm of breast	CTIF	O43310	cap binding complex dependent translation initiation factor
Malignant neoplasm of breast	SRGAP3	O43295	SLIT-ROBO Rho GTPase activating protein 3
Malignant neoplasm of breast	DAPK3	O43293	death associated protein kinase 3
Malignant neoplasm of breast	GPAA1	O43292	glycosylphosphatidylinositol anchor attachment 1
Malignant neoplasm of breast	SPINT2	O43291	serine peptidase inhibitor, Kunitz type 2
Malignant neoplasm of breast	SART1	O43290	spliceosome associated factor 1, recruiter of U4/U6.U5 tri-snRNP
Malignant neoplasm of breast	MAP3K13	O43283	mitogen-activated protein kinase kinase kinase 13
Malignant neoplasm of breast	SPINT1	O43278	serine peptidase inhibitor, Kunitz type 1
Malignant neoplasm of breast	PRODH	O43272	proline dehydrogenase 1
Malignant neoplasm of breast	DLEU1	O43261	deleted in lymphocytic leukemia 1
Malignant neoplasm of breast	ZNHIT1	O43257	zinc finger HIT-type containing 1
Malignant neoplasm of breast	SIAH2	O43255	siah E3 ubiquitin protein ligase 2
Malignant neoplasm of breast	RBFOX2	O43251	RNA binding fox-1 homolog 2

Malignant neoplasm of breast	HOXC11	O43248	homeobox C11
Malignant neoplasm of breast	PSMD3	O43242	proteasome 26S subunit, non-ATPase 3
Malignant neoplasm of breast	KLK10	O43240	kallikrein related peptidase 10
Malignant neoplasm of breast	SEPTIN4	O43236;Q8NEP4	septin 4
Malignant neoplasm of breast	GPR39	O43194	G protein-coupled receptor 39
Malignant neoplasm of breast	CRX	O43186	cone-rod homeobox
Malignant neoplasm of breast	ADAM12	O43184	ADAM metallopeptidase domain 12
Malignant neoplasm of breast	NDUFS4	O43181	NADH:ubiquinone oxidoreductase subunit S4
Malignant neoplasm of breast	PHGDH	O43175	phosphoglycerate dehydrogenase
Malignant neoplasm of breast	CYP26A1	O43174	cytochrome P450 family 26 subfamily A member 1
Malignant neoplasm of breast	PRPF4	O43172	pre-mRNA processing factor 4
Malignant neoplasm of breast	ZBTB24	O43167	zinc finger and BTB domain containing 24
Malignant neoplasm of breast	SIPA1L1	O43166	signal induced proliferation associated 1 like 1
Malignant neoplasm of breast	PLXNB1	O43157	plexin B1
Malignant neoplasm of breast	FLRT2	O43155	fibronectin leucine rich transmembrane protein 2
Malignant neoplasm of breast	TET3	O43151	tet methylcytosine dioxygenase 3
Malignant neoplasm of breast	RNMT	O43148	RNA guanine-7 methyltransferase
Malignant neoplasm of breast	KCNN4	O15554	potassium calcium-activated channel subfamily N member 4
Malignant neoplasm of breast	MEFV	O15553	MEFV innate immuity regulator, pyrin

Malignant neoplasm of breast	CLDN3	O15551	claudin 3
Malignant neoplasm of breast	KDM6A	O15550	lysine demethylase 6A
Malignant neoplasm of breast	FABP7	O15540	fatty acid binding protein 7
Malignant neoplasm of breast	PER1	O15534	period circadian regulator 1
Malignant neoplasm of breast	PDPK1	O15530	3-phosphoinositide dependent protein kinase 1
Malignant neoplasm of breast	GPR42	O15529	G protein-coupled receptor 42 (gene/pseudogene)
Malignant neoplasm of breast	CYP27B1	O15528	cytochrome P450 family 27 subfamily B member 1
Malignant neoplasm of breast	OGG1	O15527	8-oxoguanine DNA glycosylase
Malignant neoplasm of breast	SOCS1	O15524	suppressor of cytokine signaling 1
Malignant neoplasm of breast	FGF10	O15520	fibroblast growth factor 10
Malignant neoplasm of breast	CFLAR	O15519	CASP8 and FADD like apoptosis regulator
Malignant neoplasm of breast	CLOCK	O15516	clock circadian regulator
Malignant neoplasm of breast	YKT6	O15498	YKT6 v-SNARE homolog
Malignant neoplasm of breast	PLA2G10	O15496	phospholipase A2 group X
Malignant neoplasm of breast	CAPN5	O15484	calpain 5
Malignant neoplasm of breast	CCL16	O15467	C-C motif chemokine ligand 16
Malignant neoplasm of breast	P4HA2	O15460	prolyl 4-hydroxylase subunit alpha 2
Malignant neoplasm of breast	MSH4	O15457	mutS homolog 4
Malignant neoplasm of breast	TLR3	O15455	toll like receptor 3

Malignant neoplasm of breast	CD3EAP	O15446	CD3e molecule associated protein
Malignant neoplasm of breast	CCL25	O15444	C-C motif chemokine ligand 25
Malignant neoplasm of breast	ABCC5	O15440	ATP binding cassette subfamily C member 5
Malignant neoplasm of breast	ABCC4	O15439	ATP binding cassette subfamily C member 4
Malignant neoplasm of breast	ABCC3	O15438	ATP binding cassette subfamily C member 3
Malignant neoplasm of breast	SLC16A3	O15427	solute carrier family 16 member 3
Malignant neoplasm of breast	FOXP2	O15409	forkhead box P2
Malignant neoplasm of breast	TOX3	O15405	TOX high mobility group box family member 3
Malignant neoplasm of breast	SLC16A6	O15403	solute carrier family 16 member 6
Malignant neoplasm of breast	GRIN2D	O15399	glutamate ionotropic receptor NMDA type subunit 2D
Malignant neoplasm of breast	NCAM2	O15394	neural cell adhesion molecule 2
Malignant neoplasm of breast	BIRC5	O15392	baculoviral IAP repeat containing 5
Malignant neoplasm of breast	YY2	O15391	YY2 transcription factor
Malignant neoplasm of breast	BCAT2	O15382	branched chain amino acid transaminase 2
Malignant neoplasm of breast	HDAC3	O15379	histone deacetylase 3
Malignant neoplasm of breast	SLC16A4	O15374	solute carrier family 16 member 4
Malignant neoplasm of breast	EIF3H	O15372	eukaryotic translation initiation factor 3 subunit H
Malignant neoplasm of breast	EIF3D	O15371	eukaryotic translation initiation factor 3 subunit D
Malignant neoplasm of breast	SOX12	O15370	SRY-box transcription factor 12

Malignant neoplasm of breast	FANCA	O15360	FA complementation group A
Malignant neoplasm of breast	INPPL1	O15357	inositol polyphosphate phosphatase like 1
Malignant neoplasm of breast	TP73	O15350	tumor protein p73
Malignant neoplasm of breast	HMGB3	O15347	high mobility group box 3
Malignant neoplasm of breast	INPP4B	O15327	inositol polyphosphate-4-phosphatase type II B
Malignant neoplasm of breast	RAD51B	O15315	RAD51 paralog B
Malignant neoplasm of breast	GRM6	O15303	glutamate metabotropic receptor 6
Malignant neoplasm of breast	PPM1D	O15297	protein phosphatase, Mg2+/Mn2+ dependent 1D
Malignant neoplasm of breast	OGT	O15294	O-linked N-acetylglucosamine (GlcNAc) transferase
Malignant neoplasm of breast	TCAP	O15273	titin-cap
Malignant neoplasm of breast	SPTLC1	O15269	serine palmitoyltransferase long chain base subunit 1
Malignant neoplasm of breast	ATXN7	O15265	ataxin 7
Malignant neoplasm of breast	MAPK13	O15264	mitogen-activated protein kinase 13
Malignant neoplasm of breast	SLC22A1	O15245	solute carrier family 22 member 1
Malignant neoplasm of breast	LEPROT	O15243	leptin receptor overlapping transcript
Malignant neoplasm of breast	VGF	O15240	VGF nerve growth factor inducible
Malignant neoplasm of breast	CASC3	O15234	CASC3 exon junction complex subunit
Malignant neoplasm of breast	LAMA5	O15230	laminin subunit alpha 5
Malignant neoplasm of breast	UBD	O15205	ubiquitin D

Malignant neoplasm of breast	EPHB6	O15197	EPH receptor B6
Malignant neoplasm of breast	AXIN1	O15169	axin 1
Malignant neoplasm of breast	TRIM24	O15164	tripartite motif containing 24
Malignant neoplasm of breast	MDM4	O15151	MDM4 regulator of p53
Malignant neoplasm of breast	ARPC2	O15144	actin related protein 2/3 complex subunit 2
Malignant neoplasm of breast	ARPC1B	O15143	actin related protein 2/3 complex subunit 1B
Malignant neoplasm of breast	KPNA5	O15131	karyopherin subunit alpha 5
Malignant neoplasm of breast	SCAMP1	O15126	secretory carrier membrane protein 1
Malignant neoplasm of breast	ANGPT2	O15123	angiopoietin 2
Malignant neoplasm of breast	TBX3	O15119	T-box transcription factor 3
Malignant neoplasm of breast	FYB1	O15117	FYN binding protein 1
Malignant neoplasm of breast	LSM1	O15116	LSM1 homolog, mRNA degradation associated
Malignant neoplasm of breast	CHUK	O15111	component of inhibitor of nuclear factor kappa B kinase complex
Malignant neoplasm of breast	SMAD7	O15105	SMAD family member 7
Malignant neoplasm of breast	ARHGEF11	O15085	Rho guanine nucleotide exchange factor 11
Malignant neoplasm of breast	DCLK1	O15075	doublecortin like kinase 1
Malignant neoplasm of breast	ADAMTS3	O15072	ADAM metallopeptidase with thrombospondin type 1 motif 3
Malignant neoplasm of breast	MCF2L	O15068	MCF.2 cell line derived transforming sequence like
Malignant neoplasm of breast	SYNM	O15061	synemin

Malignant neoplasm of breast	SYNJ2	O15056	synaptojanin 2
Malignant neoplasm of breast	PER2	O15055	period circadian regulator 2
Malignant neoplasm of breast	KDM6B	O15054	lysine demethylase 6B
Malignant neoplasm of breast	SETD1A	O15047	SET domain containing 1A, histone lysine methyltransferase
Malignant neoplasm of breast	SEC16A	O15027	SEC16 homolog A, endoplasmic reticulum export factor
Malignant neoplasm of breast	ZNF646	O15015	zinc finger protein 646
Malignant neoplasm of breast	PPP1R2C	O14990	PPP1R2C family member C
Malignant neoplasm of breast	XPO1	O14980	exportin 1
Malignant neoplasm of breast	HNRNPDL	O14979	heterogeneous nuclear ribonucleoprotein D like
Malignant neoplasm of breast	AZIN1	O14977	antizyme inhibitor 1
Malignant neoplasm of breast	SLC27A2	O14975	solute carrier family 27 member 2
Malignant neoplasm of breast	CLGN	O14967	calmegin
Malignant neoplasm of breast	AURKA	O14965	aurora kinase A
Malignant neoplasm of breast	HGS	O14964	hepatocyte growth factor-regulated tyrosine kinase substrate
Malignant neoplasm of breast	EREG	O14944	epiregulin
Malignant neoplasm of breast	PLD2	O14939	phospholipase D2
Malignant neoplasm of breast	UBE2L6	O14933	ubiquitin conjugating enzyme E2 L6
Malignant neoplasm of breast	HAT1	O14929	histone acetyltransferase 1
Malignant neoplasm of breast	IKBKB	O14920	inhibitor of nuclear factor kappa B kinase subunit beta

Malignant neoplasm of breast	PCDH17	O14917	protocadherin 17
Malignant neoplasm of breast	GIPC1	O14908	GIPC PDZ domain containing family member 1
Malignant neoplasm of breast	TAX1BP3	O14907	Tax1 binding protein 3
Malignant neoplasm of breast	WNT9A	O14904	Wnt family member 9A
Malignant neoplasm of breast	KLF11	O14901	Kruppel like factor 11
Malignant neoplasm of breast	IRF6	O14896	interferon regulatory factor 6
Malignant neoplasm of breast	GEMIN2	O14893	gem nuclear organelle associated protein 2
Malignant neoplasm of breast	IFIT3	O14879	interferon induced protein with tetratricopeptide repeats 3
Malignant neoplasm of breast	BACH1	O14867	BTB domain and CNC homolog 1
Malignant neoplasm of breast	AIM2	O14862	absent in melanoma 2
Malignant neoplasm of breast	FFAR1	O14842	free fatty acid receptor 1
Malignant neoplasm of breast	RASGRF2	O14827	Ras protein specific guanine nucleotide releasing factor 2
Malignant neoplasm of breast	TSPAN4	O14817	tetraspanin 4
Malignant neoplasm of breast	TAAR5	O14804	trace amine associated receptor 5
Malignant neoplasm of breast	TNFRSF10C	O14798	TNF receptor superfamily member 10c
Malignant neoplasm of breast	MSTN	O14793	myostatin
Malignant neoplasm of breast	APOL1	O14791	apolipoprotein L1
Malignant neoplasm of breast	TNFSF11	O14788	TNF superfamily member 11
Malignant neoplasm of breast	NRP1	O14786	neuropilin 1

Malignant neoplasm of breast	KIF3C	O14782	kinesin family member 3C
Malignant neoplasm of breast	NDC80	O14777	NDC80 kinetochore complex component
Malignant neoplasm of breast	TNFRSF10B	O14763	TNF receptor superfamily member 10b
Malignant neoplasm of breast	CHEK1	O14757	checkpoint kinase 1
Malignant neoplasm of breast	HSD17B6	O14756	hydroxysteroid 17-beta dehydrogenase 6
Malignant neoplasm of breast	TERT	O14746	telomerase reverse transcriptase
Malignant neoplasm of breast	SLC9A3R1	O14745	SLC9A3 regulator 1
Malignant neoplasm of breast	PRMT5	O14744	protein arginine methyltransferase 5
Malignant neoplasm of breast	PDCD5	O14737	programmed cell death 5
Malignant neoplasm of breast	MAP2K7	O14733	mitogen-activated protein kinase kinase 7
Malignant neoplasm of breast	APAF1	O14727	apoptotic peptidase activating factor 1
Malignant neoplasm of breast	TRDMT1	O14717	tRNA aspartic acid methyltransferase 1
Malignant neoplasm of breast	ZNF197	O14709	zinc finger protein 197
Malignant neoplasm of breast	KMT2D	O14686	lysine methyltransferase 2D
Malignant neoplasm of breast	PTGES	O14684	prostaglandin E synthase
Malignant neoplasm of breast	TP53I11	O14683	tumor protein p53 inducible protein 11
Malignant neoplasm of breast	EI24	O14681	EI24 autophagy associated transmembrane protein
Malignant neoplasm of breast	ADAM10	O14672	ADAM metallopeptidase domain 10
Malignant neoplasm of breast	PRRG1	O14668	proline rich and Gla domain 1

Malignant neoplasm of breast	IRS4	O14654	insulin receptor substrate 4
Malignant neoplasm of breast	CHD1	O14646	chromodomain helicase DNA binding protein 1
Malignant neoplasm of breast	DNALI1	O14645	dynein axonemal light intermediate chain 1
Malignant neoplasm of breast	DVL1	O14640	dishevelled segment polarity protein 1
Malignant neoplasm of breast	CCS	O14618	copper chaperone for superoxide dismutase
Malignant neoplasm of breast	BPY2B	O14599	basic charge Y-linked 2B
Malignant neoplasm of breast	BPY2C	O14599	basic charge Y-linked 2C
Malignant neoplasm of breast	BPY2	O14599	basic charge Y-linked 2
Malignant neoplasm of breast	CIT	O14578	citron rho-interacting serine/threonine kinase
Malignant neoplasm of breast	COX7A2L	O14548	cytochrome c oxidase subunit 7A2 like
Malignant neoplasm of breast	SOCS3	O14543	suppressor of cytokine signaling 3
Malignant neoplasm of breast	NEMP1	O14524	nuclear envelope integral membrane protein 1
Malignant neoplasm of breast	AQP7	O14520	aquaporin 7
Malignant neoplasm of breast	CDK2AP1	O14519	cyclin dependent kinase 2 associated protein 1
Malignant neoplasm of breast	SOCS7	O14512	suppressor of cytokine signaling 7
Malignant neoplasm of breast	NRG2	O14511	neuregulin 2
Malignant neoplasm of breast	SOCS2	O14508	suppressor of cytokine signaling 2
Malignant neoplasm of breast	BHLHE40	O14503	basic helix-loop-helix family member e40
Malignant neoplasm of breast	ARID1A	O14497	AT-rich interaction domain 1A

Malignant neoplasm of breast	PLPP1	O14494	phospholipid phosphatase 1
Malignant neoplasm of breast	CLDN4	O14493	claudin 4
Malignant neoplasm of breast	DLGAP1	O14490	DLG associated protein 1
Malignant neoplasm of breast	SCD	O00767	stearoyl-CoA desaturase
Malignant neoplasm of breast	UBE2C	O00762	ubiquitin conjugating enzyme E2 C
Malignant neoplasm of breast	WNT7A	O00755	Wnt family member 7A
Malignant neoplasm of breast	PIK3C2B	O00750	phosphatidylinositol-4-phosphate 3-kinase catalytic subunit type 2 beta
Malignant neoplasm of breast	CES2	O00748	carboxylesterase 2
Malignant neoplasm of breast	WNT10B	O00744	Wnt family member 10B
Malignant neoplasm of breast	E2F3	O00716	E2F transcription factor 3
Malignant neoplasm of breast	NFIB	O00712	nuclear factor I B
Malignant neoplasm of breast	SLN	O00631	sarcolipin
Malignant neoplasm of breast	CCL22	O00626	C-C motif chemokine ligand 22
Malignant neoplasm of breast	PIR	O00625	pirin
Malignant neoplasm of breast	CCN1	O00622	cellular communication network factor 1
Malignant neoplasm of breast	PODXL	O00592	podocalyxin like
Malignant neoplasm of breast	GABRP	O00591	gamma-aminobutyric acid type A receptor subunit pi
Malignant neoplasm of breast	ACKR2	O00590	atypical chemokine receptor 2
Malignant neoplasm of breast	CCL21	O00585	C-C motif chemokine ligand 21

Malignant neoplasm of breast	CXCR6	O00574	C-X-C motif chemokine receptor 6
Malignant neoplasm of breast	DDX3X	O00571	DEAD-box helicase 3 X-linked
Malignant neoplasm of breast	MPHOSPH10	O00566	M-phase phosphoprotein 10
Malignant neoplasm of breast	PITPNM1	O00562	phosphatidylinositol transfer protein membrane associated 1
Malignant neoplasm of breast	SDCBP	O00560	syndecan binding protein
Malignant neoplasm of breast	EBAG9	O00559	estrogen receptor binding site associated antigen 9
Malignant neoplasm of breast	DLL1	O00548	delta like canonical Notch ligand 1
Malignant neoplasm of breast	PES1	O00541	pescadillo ribosomal biogenesis factor 1
Malignant neoplasm of breast	VWA5A	O00534	von Willebrand factor A domain containing 5A
Malignant neoplasm of breast	CHL1	O00533	cell adhesion molecule L1 like
Malignant neoplasm of breast	KRIT1	O00522	KRIT1 ankyrin repeat containing
Malignant neoplasm of breast	FAAH	O00519	fatty acid amide hydrolase
Malignant neoplasm of breast	LAD1	O00515	ladinin 1
Malignant neoplasm of breast	BCL9	O00512	BCL9 transcription coactivator
Malignant neoplasm of breast	STK25	O00506	serine/threonine kinase 25
Malignant neoplasm of breast	CLDN5	O00501	claudin 5
Malignant neoplasm of breast	BIN1	O00499	bridging integrator 1
Malignant neoplasm of breast	PSMD14	O00487	proteasome 26S subunit, non-ATPase 14
Malignant neoplasm of breast	NR5A2	O00482	nuclear receptor subfamily 5 group A member 2

Malignant neoplasm of breast	BTN3A3	O00478	butyrophilin subfamily 3 member A3
Malignant neoplasm of breast	MEIS1	O00470	Meis homeobox 1
Malignant neoplasm of breast	PLOD2	O00469	procollagen-lysine,2-oxoglutarate 5-dioxygenase 2
Malignant neoplasm of breast	GOLIM4	O00461	golgi integral membrane protein 4
Malignant neoplasm of breast	PIK3R2	O00459	phosphoinositide-3-kinase regulatory subunit 2
Malignant neoplasm of breast	PLK4	O00444	polo like kinase 4
Malignant neoplasm of breast	PIK3C2A	O00443	phosphatidylinositol-4-phosphate 3-kinase catalytic subunit type 2 alpha
Malignant neoplasm of breast	RTCA	O00442	RNA 3'-terminal phosphate cyclase
Malignant neoplasm of breast	DNM1L	O00429	dynamin 1 like
Malignant neoplasm of breast	IGF2BP3	O00425	insulin like growth factor 2 mRNA binding protein 3
Malignant neoplasm of breast	CCRL2	O00421	C-C motif chemokine receptor like 2
Malignant neoplasm of breast	EEF2K	O00418	eukaryotic elongation factor 2 kinase
Malignant neoplasm of breast	FOXN3	O00409	forkhead box N3
Malignant neoplasm of breast	PDE2A	O00408	phosphodiesterase 2A
Malignant neoplasm of breast	WASL	O00401	WASP like actin nucleation promoting factor
Malignant neoplasm of breast	SLC33A1	O00400	solute carrier family 33 member 1
Malignant neoplasm of breast	DCTN6	O00399	dynactin subunit 6
Malignant neoplasm of breast	QSOX1	O00391	quiescin sulfhydryl oxidase 1
Malignant neoplasm of breast	FOXE1	O00358	forkhead box E1

Malignant neoplasm of breast	SULT1C2	O00338	sulfotransferase family 1C member 2
Malignant neoplasm of breast	SLC28A1	O00337	solute carrier family 28 member 1
Malignant neoplasm of breast	PDHX	O00330	pyruvate dehydrogenase complex component X
Malignant neoplasm of breast	PIK3CD	O00329	phosphatidylinositol-4,5-bisphosphate 3-kinase catalytic subunit delta
Malignant neoplasm of breast	ARNTL	O00327	aryl hydrocarbon receptor nuclear translocator like
Malignant neoplasm of breast	CDC7	O00311	cell division cycle 7
Malignant neoplasm of breast	TNFRSF11B	O00300	TNF receptor superfamily member 11b
Malignant neoplasm of breast	CLIC1	O00299	chloride intracellular channel 1
Malignant neoplasm of breast	HIP1	O00291	huntingtin interacting protein 1
Malignant neoplasm of breast	DFFA	O00273	DNA fragmentation factor subunit alpha
Malignant neoplasm of breast	PGRMC1	O00264	progesterone receptor membrane component 1
Malignant neoplasm of breast	CBX4	O00257	chromobox 4
Malignant neoplasm of breast	MEN1	O00255	menin 1
Malignant neoplasm of breast	F2RL2	O00254	coagulation factor II thrombin receptor like 2
Malignant neoplasm of breast	ATOX1	O00244	antioxidant 1 copper chaperone
Malignant neoplasm of breast	BMPR1B	O00238	bone morphogenetic protein receptor type 1B
Malignant neoplasm of breast	PSMD9	O00233	proteasome 26S subunit, non-ATPase 9
Malignant neoplasm of breast	CORT	O00230	cortistatin
Malignant neoplasm of breast	TNFRSF10A	O00220	TNF receptor superfamily member 10a

Malignant neoplasm of breast	LGALS8	O00214	galectin 8
Malignant neoplasm of breast	APBB1	O00213	amyloid beta precursor protein binding family B member 1
Malignant neoplasm of breast	RHOD	O00212	ras homolog family member D
Malignant neoplasm of breast	TLR4	O00206	toll like receptor 4
Malignant neoplasm of breast	AP3B1	O00203	adaptor related protein complex 3 subunit beta 1
Malignant neoplasm of breast	RAB27B	O00194	RAB27B, member RAS oncogene family
Malignant neoplasm of breast	LGALS9	O00182	galectin 9
Malignant neoplasm of breast	KCNK1	O00180	potassium two pore domain channel subfamily K member 1
Malignant neoplasm of breast	EYA2	O00167	EYA transcriptional coactivator and phosphatase 2
Malignant neoplasm of breast	HAX1	O00165	HCLS1 associated protein X-1
Malignant neoplasm of breast	MYO1C	O00159	myosin IC
Malignant neoplasm of breast	ACOT7	O00154	acyl-CoA thioesterase 7
Malignant neoplasm of breast	PDLIM1	O00151	PDZ and LIM domain 1
Malignant neoplasm of breast	SGK1	O00141	serum/glucocorticoid regulated kinase 1
Malignant neoplasm of breast	KIF2A	O00139	kinesin family member 2A
Malignant neoplasm of breast	SMIM22	K7EJ46	small integral membrane protein 22
Malignant neoplasm of breast	TEX46	H3BTG2	testis expressed 46
Malignant neoplasm of breast	UBE2Q2L	H0YL09	ubiquitin conjugating enzyme E2 Q2 like
Malignant neoplasm of breast	PRNP	F7VJQ1;P04156	prion protein

Malignant neoplasm of breast	PRSS46P	E5RG02	serine protease 46, pseudogene
Malignant neoplasm of breast	PRAC2	D3DTV9	PRAC2 small nuclear protein
Malignant neoplasm of breast	KLLN	B2CW77	killin, p53 regulated DNA replication inhibitor
Malignant neoplasm of breast	HACD1	B0YJ81	3-hydroxyacyl-CoA dehydratase 1
Malignant neoplasm of breast	AHRR	A9YTQ3	aryl-hydrocarbon receptor repressor
Malignant neoplasm of breast	KPNA7	A9QM74	karyopherin subunit alpha 7
Malignant neoplasm of breast	FOXO6	A8MYZ6	forkhead box O6
Malignant neoplasm of breast	PHF20L1	A8MW92	PHD finger protein 20 like 1
Malignant neoplasm of breast	HEPACAM2	A8MVW5	HEPACAM family member 2
Malignant neoplasm of breast	SYCE1L	A8MT33	synaptonemal complex central element protein 1 like
Malignant neoplasm of breast	CLCA1	A8K7I4	chloride channel accessory 1
Malignant neoplasm of breast	A2ML1	A8K2U0	alpha-2-macroglobulin like 1
Malignant neoplasm of breast	LINC00473	A8K010	long intergenic non-protein coding RNA 473
Malignant neoplasm of breast	ANKRD30BL	A7E2S9	ankyrin repeat domain 30B like
Malignant neoplasm of breast	SRRM3	A6NNA2	serine/arginine repetitive matrix 3
Malignant neoplasm of breast	LRRC37A	A6NMS7	leucine rich repeat containing 37A
Malignant neoplasm of breast	IFITM10	A6NMD0	interferon induced transmembrane protein 10
Malignant neoplasm of breast	LRRC37A2	A6NM11	leucine rich repeat containing 37 member A2
Malignant neoplasm of breast	TTC36	A6NLP5	tetratricopeptide repeat domain 36

Malignant neoplasm of breast	RTL1	A6NKG5	retrotransposon Gag like 1
Malignant neoplasm of breast	RASSF10	A6NK89	Ras association domain family member 10
Malignant neoplasm of breast	POTEM	A6NI47	POTE ankyrin domain family member M
Malignant neoplasm of breast	PCARE	A6NGG8	photoreceptor cilium actin regulator
Malignant neoplasm of breast	PLEKHD1	A6NEE1	pleckstrin homology and coiled-coil domain containing D1
Malignant neoplasm of breast	RCCD1	A6NED2	RCC1 domain containing 1
Malignant neoplasm of breast	TMEM8B	A6NDV4	transmembrane protein 8B
Malignant neoplasm of breast	PGP	A6NDG6	phosphoglycolate phosphatase
Malignant neoplasm of breast	TMEM189	A5PLL7;Q13404	transmembrane protein 189
Malignant neoplasm of breast	TMEM189-UBE2V1	A5PLL7;Q13404	TMEM189-UBE2V1 readthrough
Malignant neoplasm of breast	UBE2V1	A5PLL7;Q13404	ubiquitin conjugating enzyme E2 V1
Malignant neoplasm of breast	RGSL1	A5PLK6	regulator of G protein signaling like 1
Malignant neoplasm of breast	POTEF	A5A3E0	POTE ankyrin domain family member F
Malignant neoplasm of breast	CPED1	A4D0V7	cadherin like and PC-esterase domain containing 1
Malignant neoplasm of breast	SBNO1	A3KN83	strawberry notch homolog 1
Malignant neoplasm of breast	TMEM131L	A2VDJ0	transmembrane 131 like
Malignant neoplasm of breast	HEATR9	A2RTY3	HEAT repeat containing 9
Malignant neoplasm of breast	HFM1	A2PYH4	helicase for meiosis 1
Malignant neoplasm of breast	PXDNL	A1KZ92	peroxidasin like

Malignant neoplasm of breast	ODAM	A1E959	odontogenic, ameloblast associated
Malignant neoplasm of breast	ARHGAP10	A1A4S6	Rho GTPase activating protein 10
Malignant neoplasm of breast	MED19	A0JLT2	mediator complex subunit 19
Malignant neoplasm of breast	UBA6	A0AVT1	ubiquitin like modifier activating enzyme 6
Malignant neoplasm of breast	E2F8	A0AVK6	E2F transcription factor 8
Malignant neoplasm of breast	SLC12A8	A0AV02	solute carrier family 12 member 8
Malignant neoplasm of breast	PERCC1	A0A1W2PR82	proline and glutamate rich with coiled coil 1
Malignant neoplasm of breast	C2orf92	A0A1B0GVN3	chromosome 2 open reading frame 92
Malignant neoplasm of breast	C10orf143	A0A1B0GUT2	chromosome 10 open reading frame 143
Malignant neoplasm of breast	CRYAA	A0A140G945;P02489	crystallin alpha A
Malignant neoplasm of breast	SIK1	A0A0B4J2F2;P57059	salt inducible kinase 1
Malignant neoplasm of breast	GATD3B	A0A0B4J2D5;P0DPI2	glutamine amidotransferase like class 1 domain containing 3B
Malignant neoplasm of breast	GATD3A	A0A0B4J2D5;P0DPI2	glutamine amidotransferase like class 1 domain containing 3A
Malignant neoplasm of breast	CYP2D7	A0A087X1C5	cytochrome P450 family 2 subfamily D member 7 (gene/pseudogene)

Table S5

Venn intersection between active ingredients predicted targets and targets of DisGeNET database
**Common targets uniprot ids**
Q9HBH9
P19793
P21980
P04150
P53779
P07949
P09917
P09874
O14757
O00329
P53985
P10415
P11413
P10275
P06756
P32245
Q01959
P11217
Q06124
Q9BZL6
O75116
O15379
Q16548
Q8TDU6
P51449
P25103
P51812
P33527
P62136
Q9Y2K7
Q9Y2I1

P53667
P13866
P45983
Q92731
Q9Y5N1
P05231
O15303
P00519
P68400
Q92843
P04818
P28838
Q13003
P05121
P04035
O60885
Q9Y256
P34972
O00408
P14902
P28482
P49286
P50750
P02766
P15085
P18031
P56545
P52333
P17861
P07858
P30304
O43174
Q9H4B4
P08253

P04439
P25774
P08238
P04049
P06401
O00444
P15538
Q96RI1
P42345
Q9P1W9
P00749
Q13304
P11388
P42336
Q7KZI7
O14965
Q13002
P24666
Q8NFI3
Q8TD08
O00141
O60674
P04629
P60033
P06280
P48147
P10828
P08263
P11712
P42330
P29350
P35398
P49841
P78536

P30556
P28845
P23219
Q14994
P20839
O94953
P23458
Q15722
P24530
P00915
P08581
P08473
Q9Y2R2
O00750
O14746
P05093
P19021
Q9GZT9
P16050
O15382
Q07820
P12268
P20142
P19634
P50416
P17252
P29466
P53350
Q15303
Q08499
P18405
P16662
P62508
O14684

P43490
Q8NER1
P42338
Q14833
Q16739
P27487
P22303
Q7Z2W7
P15104
P05413
P11940
P08254
P10827
P22736
O15530
P00742
P07384
P07333
P11926
P17948
P31213
P35354
O43570
P31645
P49760
P32239
P48039
P03372
Q00535
P00533
Q07869
P43116
P04626
Q16769

P00734
P00918
Q9HC97
P22607
Q13822
P23786
P16278
P29474
P04066
P15090
P24941
P51955
P43378
P15056
O00767
Q8TDS5
P11387
P11509
P43088
Q9ULA0
Q9Y2T6
Q9NYY3
P25098
Q9H3R0
Q16539
P43004
P35408
Q9UPP1
Q8N1Q1
P11473
P42858
Q96IY4
Q01650
P06746

Q96GD4
P80365
P29475
P15121
P52895
Q8TBX8
P45984
O14842
P06241
P37231
P53396
P36897
Q9H6Z9
Q9Y478
Q06187
P07900
Q99720
P23975
P18054
O60218
Q99523
Q9BYF1
P12821
P16581
P21397
Q13163
Q02750
P00797
P28223
O15264
O96017
P05186
P11511
Q04609

Q13526
P37023
P10145
Q12809
Q16790
O43451
P41229
P47712
P54760
P08246
P35218
Q8IXJ6
P21554
P14174
P30542
P48736
P10586
P00747
Q13464
P41279
P35228
P17706
P24557
P14555
P08311
P06276
P43115
Q13255
P09467
P35968
Q9BUB5
P30305
P21731
Q15125

Q03181
Q5NUL3
P05362
P41235
O00519
Q8TDS4
P11802
P09211
P25024
P04054
Q01469
Q92793
P98170
P23946
P14550
P48449
P04278
P25101
P24723
P07099
Q9Y6L6
P27361
P45452
P33261
P49682
Q13188
O00748
Q5S007
Q00987

Table S6

The top 10 ranked string PPI NETWORK Target genes			
**Rank**	**uniprot**	**Score**	**gene id**
1	P05231	134	IL6
2	P00533	117	EGFR
3	P10415	107	BCL2
4	P37231	104	PPARG
5	P07900	103	HSP90AA1
6	P03372	99	ESR1
7	P27361	97	MAPK3
8	P35354	95	PTGS2
9	P08238	86	HSP90AB1
10	P04626	80	ERBB2

Table S7

GoTerm	Enrichment score	Subgroup
Enzyme linked receptor protein signaling pathway	71.07176189	BP
Transmembrane receptor protein tyrosine kinase signaling pathway	35.71267637	BP
Neurotransmitter metabolism	35.71267637	BP
Regulation of blood pressure	23.84799984	BP
Cellular morphogenesis during differentiation	23.84799984	BP
Anti-apoptosis	8.90339785	BP
Phosphoinositide 3-kinase complex	57.29305933	CC
Endomembrane system	22.98583817	CC
Cyclin-dependent protein kinase holoenzyme complex	16.42782443	CC
Sarcolemma	14.37691002	CC
Membrane raft	7.971069904	CC
Mitochondrial outer membrane	7.919516302	CC
Centriole	7.835170767	CC
Spindle microtubule	7.182511811	CC
Ligand-dependent nuclear receptor activity	18.29168542	MF
Kinase activity	15.27036451	MF
Protein tyrosine phosphatase activity	14.23787179	MF
Transmembrane receptor protein tyrosine kinase activity	13.97149211	MF
Lipid kinase activity	12.20122413	MF
Protein-tyrosine kinase activity	11.23822301	MF
Protein serine/threonine kinase activity	6.61383306	MF
Peptidase activity	6.13841016	MF

Table S8

KEGG pathway enrichment analysis of results for breast cancer treatment of SpE			
**Pathway**	**Fold Enrichment**	**Enrichment FDR (P-value)**	**Pathway Genes**
EGFR tyrosine kinase inhibitor resistance	20.90992478	1.02E-19	79
Endocrine resistance	19.12707857	1.28E-20	95
AGE-RAGE signaling pathway in diabetic complications	18.99666667	1.99E-21	100
Prostate cancer	18.73270581	1.80E-20	97
Progesterone-mediated oocyte maturation	17.34478261	5.80E-19	100
FoxO signaling pathway	17.02323266	1.70E-23	131
HIF-1 signaling pathway	15.9126446	3.33E-18	109
Insulin resistance	15.29522276	4.11E-17	108
Neurotrophin signaling pathway	15.2695165	1.24E-18	119
Insulin signaling pathway	13.26330266	2.14E-17	137
Hepatitis B	11.79917184	4.41E-17	161
MicroRNAs in cancer	11.79917184	4.41E-17	161
Lipid and atherosclerosis	11.57862657	1.99E-21	214
Proteoglycans in cancer	11.44870139	4.80E-20	202
Chemical carcinogenesis	11.32001766	2.82E-19	197
Focal adhesion	10.73724638	4.21E-18	200
Pathways in cancer	9.661963358	1.99E-40	530
PI3K-Akt signaling pathway	8.632727422	7.42E-22	354
MAPK signaling pathway	8.427979888	6.60E-18	294
Metabolic pathways	3.461708569	1.56E-17	1527

Table S9

The top 5 compounds ranked by Degree method		
**Rank**	**Name**	**Score**
1	442879	80
2	5312775	78
3	36689743	69
4	46178008	39
5	7991	26

Table S10

Docking Rank	Proposed compound (bioactives)	Subclass	Pubchem ID (CID)	EGFR
1	Hinokinin	Lignan	442879	-6.2373
2	Hydroxylinolenic acid II	Fatty acid	5312775	-6.1899
3	Swainsonine	Alkaloid	51683	-6.0959
4	chromequinolide	Quinone	162870052	-5.7314
5	N-valeryl-valine	Amino acid conjugated with fatty acid	36689743	-5.2791
6	Dihydrocinammic acid	Dihydrocinammic acid	107	-5.0666
7	Peyssonoic acid B	Sesquiterpene hydroquinones	46178008	-4.8753
8	*p*-Dihydrocoumaric acid	Dihydrocinammic acid	129846263	-4.5789
9	Valeric acid	Fatty acid	7991	-4.0252

Docking Rank	Proposed compound (bioactives)	Subclass	Pubchem ID (CID)	PI3K
1	Swainsonine	Alkaloid	51683	-5.1383
2	*p*-Dihydrocoumaric acid	Dihydrocinammic acid	129846263	-4.8623
3	N-valeryl-valine	Amino acid conjugated with fatty acid	36689743	-4.8375
4	chromequinolide	Quinone	162870052	-4.8012
5	Peyssonoic acid B	Sesquiterpene hydroquinones	46178008	-4.7989
6	Dihydrocinammic acid	Dihydrocinammic acid	107	-4.7735
7	Hinokinin	Lignan	442879	-4.728
8	Hydroxylinolenic acid II	Fatty acid	5312775	-4.7154
9	Valeric acid	Fatty acid	7991	-0.3687

Docking Rank	Proposed compound (bioactives)	Subclass	Pubchem ID (CID)	ERK (MAPK)
1	Hydroxylinolenic acid II	Fatty acid	5312775	-4.9854
2	Peyssonoic acid B	Sesquiterpene hydroquinones	46178008	-4.8738
3	*p*-Dihydrocoumaric acid	Dihydrocinammic acid	129846263	-4.7872
4	Dihydrocinammic acid	Dihydrocinammic acid	107	-4.7865
5	Swainsonine	Alkaloid	51683	-4.7838

6	**N-valeryl-valine**	**Amino acid conjugated with fatty acid**	**36689743**	-4.7617
7	**Hinokinin**	**Lignan**	**442879**	-4.6367
8	**Valeric acid**	**Fatty acid**	**7991**	-3.9005
9	**chromequinolide**	**Quinone**	**162870052**	-3.3318

Supp Figure S2. PPI network

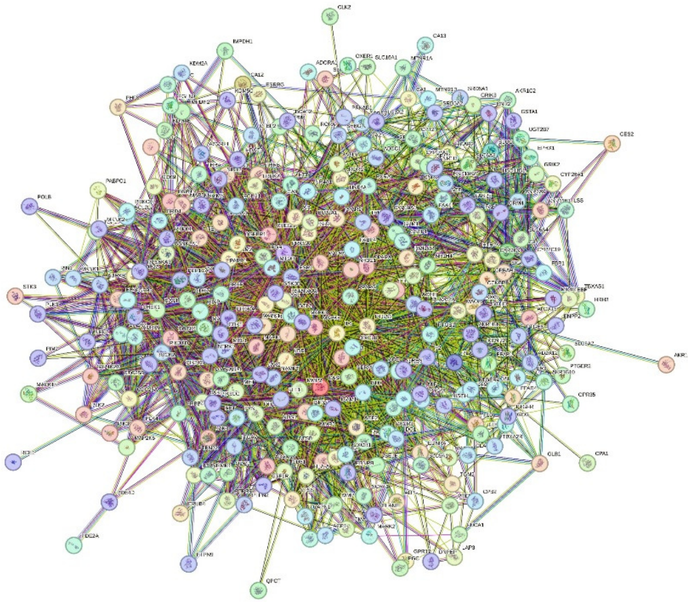

Supp **Figure S3.** KEGG, PI3K-Akt signal pathway

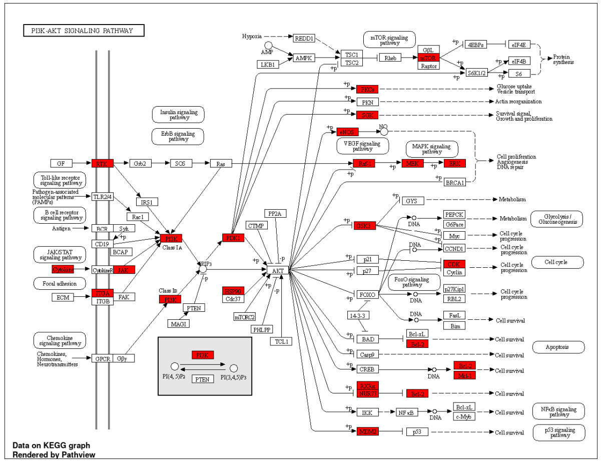

Supplement: Supplementary file 1 [file marinedrugs-22-00328-s001.zip › marinedrugs-3096105-supplementary.pdf]
